# Exploring the patterns of multisectoral approach in fighting COVID-19 Pandemic in SNNPR, Ethiopia: A qualitative case study approach

**DOI:** 10.1371/journal.pone.0263667

**Published:** 2022-02-25

**Authors:** Abraham A. Ali, Akmel M. Usman, Fekadeselassie B. Badebo, Solomon H. Tilahun

**Affiliations:** Policy Study and Research Institute, SNNPR, Hawassa, Ethiopia; Curtin University, AUSTRALIA

## Abstract

**Background:**

Since COVID-19 has been declared as a global pandemic, South Nations, Nationalities People’s Region, Ethiopia, has been responding by establishing strong structure. The response against the disease requires consolidated efforts, However, there is paucity of information about the patterns of multisectoral collaboration actions in the region. Therefore, this study was conducted with the aim of exploring the patterns of multisectoral collaboration against the COVID-19 using a qualitative case study design. Data were collected by key informant interview and document review and analyzed using a content analysis framework focused on case study data analysis.

**Result:**

The study has indicated a unique perspective in establishing functional multisectoral approach with strong courage and motivation of most involved stakeholder. Almost all stakeholders have shown clear understanding about the multidimensional burdens the disease could cause and the necessities for joint efforts to tackle the multidimensional problems. The study further elucidated that despite the encouraging beginning, the eventual slowdown undertakings in the joint actions. This included failure to meet regularly to monitor and evaluate as stated earlier and societies’ reluctances despite consistent information dissemination.

**Conclusion:**

A firm understanding about multidimensional impacts of COVID-19 by all stakeholders was revealed in the region. Remarkable achievements were observed at the early stage of disease prevention and control responses with encouraging multisectoral approach as guided by the convergence model. However, several setbacks were observed in the process of multisectoral approach intervention as indicated by the conceptual framework. Hence, to ensure sustainable MSA, revitalization of the initial commitment of all stakeholders and strengthening MSA considering end-to end approach of the convergence frame is crucial; subsequent quantitative study recommended to establish wider determinants on success of MSA.

## Introduction

Multisectoral collaboration is an approach that involves various expertise and capacities to solve multidimensions problems beyond the competence of single sector or institution [1,2]. Multisectoral approach (MSA) refers to “deliberate collaboration among various stakeholder groups (e.g., government, civil society, and private sector) and sectors (e.g., health, education, environment and economy), jointly achieve a policy outcome” [3,4].

Multisectoral collaboration is one of the necessary conditions for the effective management of global population’s socio-economic development arena. The benefits of multisectoral collaboration are obvious and established ones, despite the challenges exhibited in handling the processes [5,6].

Health related matters are among the numerous challenges that had been encountered humankind and necessitated the collaborative efforts of various stakeholders in the history [7–9]. In relation to this, the establishment of World Health Organization (WHO) represents the demand for collective efforts to improving global health outcomes [10,11]. The WHO mandates imply that the attainment of these mandates require the efforts of multiple sectors, organizations, or institutions. Hence, numerous efforts have been taken since its establishment, among which the promulgation of the primary health care (PHC),1978, Alma -Ata, the former USSR, was a paradigm shift in bringing efforts together and revealing functional multisectoral approach and principles [12,13].

Many epidemics and pandemics had occurred and have been occurring since the establishment of WHO. To mention a few, recently global health programs have been grappling with: Asian flu, HIV/AIDS, H1N1 Swine flu, West Africa Ebola and Zika, which have caused millions of illnesses and deaths globally. The lessons from prevention and control of epidemics and pandemics can be remarkable directives to guide interventions for the present and future incidents [14–16].

The outbreak of COVID-19 is one of the world’s grave pandemics that crossed national boarders within a very short period [17,18]. Moreover, it has been the causes for the infection of more than thirty- two million people around the world and has been reason for the death of close to million lives until September 24, 2020. The WHO, as the global leading organization is responsible for health affairs urged the UN member nations and its allies to institute strong multisectoral collaboration across all stages of interventions. It took the primary initiative to function closely with the COVID-19 origin nation, China and further played pivotal role in involving many other nations [19].

Evidence clearly revealed that those nations registered better response to the management of COVID-19 pandemic were practiced well-coordinated and cooperative multisectoral collaboration. For example, China aggressively intervened and could limit the progression of the disease and controlled it within short time [20–24].

The government of Ethiopia commenced responding to COVID-19 pandemic considering as a national priority by establishing a ministerial committee, chaired by the prime minister of the federal government. Similarly, several sub ministerial committees were established to support efforts of crisis management. Moreover, the Ethiopian parliament declared a state of emergency to fight the disease and, several rules, protocols, and regulations were established are currently in action [25,26].

The Ministry of Health of the Federal government of Ethiopia (FMOH) carried out all the necessary preparations to tackle COVID-19 pandemic. As the result, inter alia, a national comprehensive COVID-19 management document was developed and distributed to all concerned stakeholders [27].

Regional governments and city administrations are engaged in fighting the disease in collaboration with the federal government. Moreover, civic organizations, multi-lateral and bilateral organizations, NGOs and GOs, universities and research institutions, religious institutions and the public at large; deeply involved in the fighting of the disease and by the succinct technical guidance of the minister of health, all stakeholders involved in running the prevention and control of COVID-19.

Similarly, the South Nations, Nationalities and peoples Region (SNNPR) government has established taskforces in the response to COVID-19 pandemic. On the other hand, the nature of the disease is not yet fully understood, and no one can precisely predict the end of the crises. Therefore, the efforts undertaken shall be geared towards the magnitude and complexity of the problem. Hence, it is important to document and present the patterns of multisectoral approach to tackle the problem comprehensively and stretched manner in region. Any gaps in the MSA should be identified and corrective actions should be sought to ensure sustainable responses. The study was conducted to explore the patterns of collaborative efforts of the multiple actors who engaged in prevention and control of the COVID-19 pandemic and identify any gaps in implementing the MSA in the desired manner.

The significance of the study is to learn lessons from the process of implementation and fill the gaps observed in the undertaking. At the same time, the findings of the study would help to sustainably handle multisectoral collaborative actions by providing proper recommendations and the experience will be a springboard for similar actions the future.

## Materials and methods

### 2.1. Study area

The study was conducted in South Nations, Nationalities and People’s Region (SNNPR) before the declaration of the Sidama as an independent state. The region is one of the nine regional administrations, and two city administrations make up the federal republic government of Ethiopia [28]. The capital of the region, Hawassa, is located 275 KM south of Addis Ababa, on the main highway to Moyale–Kenya. SNNPR is administratively divided into 18 zones, one city administration and seven special woredas. The region is well known for its ethnic, cultural, linguistic, and geographical diversities. It is thus, hosts for 56 ethnic diversities which is more than the three-fourth of the national diversities. Based on the population projection of 2007, the regional population is estimated to be 22 million [29]. The region is one of the densely populated regions of the country.

In terms of health facilities and health services delivery the region is has covered 90 percent regarding the primary health services (health posts). There are 79 hospitals, 729 health centers and 3961 health posts. and 769 private pharmacies and drug shops and 2 diagnostic centers in the region.

Regarding the preparedness of the regional government to prevent and control COVID-19 pandemic, the region established and prepared 19 treatment centers, 02 test centers, 34 quarantine, 57 isolation centers. The region further organized 6 surveillance teams in different parts and, there are four Rapid Response Team (RRT) and 2 laboratories having 30 staff members [30].

### 2.2. Conceptual framework

The study was guided by the convergence model for the exploration of the patterns of the multisectoral approach. The **’Convergence’ Model**: Convergence is an approach to problem solving that cut across disciplinary boundaries [31–33]. Convergence can be characterized as the extent to which sectors, stakeholders, and administrative levels have been organized to work together with respect to a response across sectors for a certain issue: from line functioning continuing (sectors work separately with little communication, interaction or common strategic plan on issue), networking (maintaining sectoral responsibilities while recognizing and exchanging information on shared interest), cooperation/coordination(maintaining sectoral responsibilities while joining together to reach shared goals on certain issues using formal or informal structures, agreements, and links), collaboration (maintaining sectoral responsibilities while sharing some resources or personnel to facilitate joint strategic planning and action) to integration (full convergence with sectors sharing structures, resources, and merged remits) [34,35].

**Fig 1:**
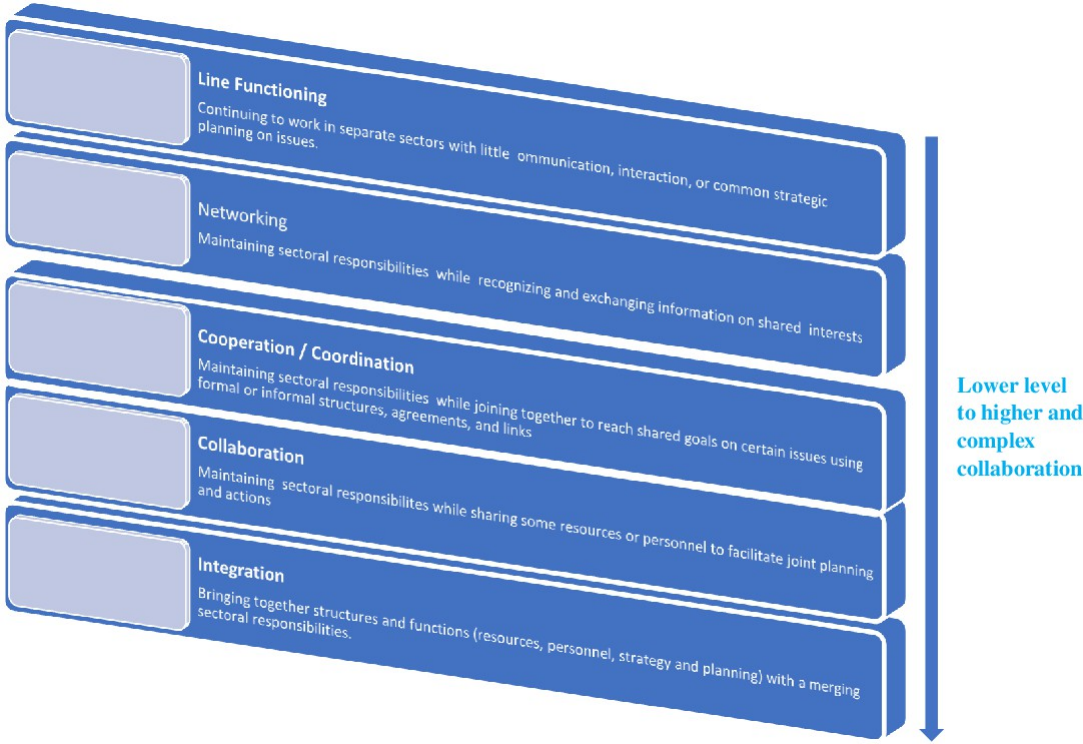
A continuum of multisectoral collaboration. Adopted from Harris and Drimie [33]; Himmelman [34,35].

Prevention and control of COVID-19 demand multifaceted efforts. Some of the actions are sector specific and others are crosscutting (e.g., resource mobilization, common guidelines development and information dissemination), across all sectors. Therefore, the convergence model with varying level but closely managed multisectoral model is chosen (see Fig 1) [33–35].

### 2.3. Study design

This study has employed a case study approach of the qualitative design. Case study is an in-depth empirical method, using multiple sources of evidence, that attempts to systematically investigate unfolding and development of a complex events within its context [36,37].

#### 2.3.1. Sampling and sampling frame

A purposive sampling technique was employed for this study with the potential of offering maximum flexibility to enroll the key informants with rich information [38]. Sampling units were bureaus, departments, and offices at respective hierarchies (region, zone and district). Other organizations/ institutions included in the study were non-governmental organizations and civic organizations enrolled in accordance with their level of involvement and previous acquaintances with the regional health bureau in one way or another.

President’s office/ chief administrator office, Bureaus of Health, Agriculture and Natural Resource Development, Water Energy and Irrigation Development, Women, Children and Youth Affairs, Urban and Housing Development, Transport and Road Development, Finance, Education, Labor and Social Affairs, Government Communication, Debub TV and Radio, Coalition of Religious institution, Civic Organization (Teacher’s Association) and The Ethiopian Red Cross Society, Debub Branch were involved in the study.

One third of the zones with larger population sizes and with high-risk conditions for the covid-19 exposure were enrolled for the data collection. Accordingly: Sidama, Gurage, Wolaita, Gedeo, Gamo and Hawassa City Administration were chosen.

Departments with close mandate to COVID-19 prevention and control were included. These included the office of the chief Administrator, Departments of Health, Education, Agriculture, Water, Sanitation and Energy, Women, Children and Youth Affairs, Labor and Social Affairs, Finance, Urban and Housing Development and Transportation and Road Development.

#### 2.3.2. Data collection

The study has employed key informant interview and document review to collect information. Data collection was conducted between June 25 to July 15, 2019. Key informants enrolled based on their position in the organization and acquaintance to the overall institutional affairs and their level of involvement to the COVID-19 prevention and control. It was due to the requirement of the copious information for a given phenomenon [39].

Data collection instrument was developed against the study objectives by consulting literature and adopting from similar sources. The study guide has encompassed all important dimensions related to the planning, intervention, monitoring and evaluation of the actions. The instrument was commented by professional team for its adequacy and completeness. Data collectors were recruited based on the professional background and previous qualitative data collection experiences. Accordingly, professionals at least with a first degree were involved. Orientation was provided for a day on how to competently collect data (note taking during the discussion). Almost all key informants from the selected institutions were participated in the interview. The participation depended on the level of saturation of information and the capability to capture thick information. Principal investigators have moderated the interview and the assistants took note and recorded the audio tape.

Key informant interview was conducted by the prior arrangement, once, the right person was designated, and communicated. The date and time for the interview was scheduled by discussing with the informant based on the convenience. On the interview day, the team was arrived 15 minutes before the interview time.

Just on time, courtesy greeting was offered, and permission was requested to get into the office. All instrument and audio materials were checked for the functionality before the commencement of the interview. The purpose of the study was briefly informed to the key informant and a letter of permission was given. Adequate time was given to read the support letter to the key informant and the team waited until he/she will formally allow conducting the interview. The interview was conducted by using an interview guide by giving adequate time to respond. Key informants prompted and probed to further elaborate their thoughts. The interview lasted for 40–60 minutes on average. All the key informants’ interview sessions were conducted with the maximum care by maintaining the necessary COVID-19 prevention and control protocols.

When the interview ends, the team left the room by appreciating the informant for the time and information offered. And notice was given when need arise, follow-up communication via phone would continue.

Secondary data were collected from document reviews. Policy related document, minutes of the meeting, decision-based documents at various levels were consulted based on the checklist prepared for this purpose. Hard and soft copies of the document were gathered.

#### 2.3.3. Data analysis

Data were analyzed using a content analysis of the qualitative data analysis method. The key informant interview audio taped material was transcribed verbatim in Amharic (the regional working language) and translated to English. Simultaneously, the handwritten documents were organized into mature notes. Comparison and matching of the handwritten summary note with the transcribed information was done. Ones all the handwritten document and audio taped transcripts organized, descriptive coding was done. Following the coding, data, reduction by side noticing was done. After side noting, creating or identifying major themes, were taken place. The process has taken several consultations to the verbatim transcription and handwritten notes. Codes developed into themes and rearranging the several themes and intermarrying similar themes was done. Finally, interpretation of the themes, in line with the study objectives was conducted [40–42]. Data from the document review were categorized according to the checklist; the availability of the desired documents, to what the implementing entities has organized and utilizes them. Findings were presented based on the major themes in line with the study objectives.

### 2.4. Ethical clearance

This study required the involvement of numerous stakeholders and institutions working in the region. As the result, the study needs to pass all the necessary steps related to the research ethics. The topic under the investigation is critical and concerns all humankinds. The stake was high. Therefore, ethical clearance was obtained from the Southern Nations and Nationalities and People’s Regional Health Bureau institutional ethics review board. Support letters from responsible authorities were obtained and submitted to the organizations/institutions/ individuals participating in the study. Informed written consent was obtained from the study participants before commencing the data collection (the sample was annexed under the supplementary document # 3). The purpose of the study was communicated to the study participants. The benefits of the study were equally being shared. The rights of the study participants were maintained across the study period. All the methods were carried out in accordance with the relevant guideline and regulations witnessed by the ethical review board document annexed as a supplementary document #2. The finding of the study would be communicated to the authorities such as the Regional Health Bureau, Regional President’s Office, and other sectors collaborating in the fight against COVID-19 pandemics. The finding also will be published in scientific journals.

## Result

Since COVID-19 pandemic has multidimensional effect; the response to the problems arising from the disease have necessitated the multisectoral approach. All key informants pointed out their convictions that such a huge problem cannot be countered unless consolidated joint efforts put in place. This is substantiated by the direct statements by the key informants as:

*What we have done is developing recognitions by all members of the society about the depth of the problems. No single sector can fully respond for the disease related challenges. Thus, we requested the involvement of all sectors and people to lend their hands.*

*Multisectoral collaboration is the necessary condition for this disease prevention. For example, our region is bordering with two neighbor countries: Kenya and South Sudan. Due to the common boarder, people move in ward from both boards need serious attention. When they inter, we need place to quarantine them. This action needs resource. Therefore, the bureau of health, bureau of peace and security, bureau of transport and road development and the resource mobilization sub-task force members need joint actions.*

SNNPR government has recognized that proper collaboration is crucial for the prevention and control of the pandemic. Therefore, the process emanated from the very nature of the disease that it intricates almost all spheres of human lives and livelihoods. According to the key informants’ expression it is explicates as:

*One of the reasons necessitated such joint efforts to intervene the disease prevention and control actions are the very nature of the disease*. *For one thing*, *there is no known curative or preventive vaccine so far*. *Moreover*, *the disease affects every corner and life endeavors*, *Therefor*, *collaborative efforts is a must*.

*Look; the disease nature is terrifying. Leave alone us, the poor guys; this disease puzzled the global nations categorized as most developed ones. Therefore, we have realized that unless we move together, we cannot manage this deadly pandemic disease. This is how we have established a joint action point together.*

The collaborative efforts of multiple sectors in SNNPR in connection to COVID-19 measures are evidenced first by its organization into main and sub taskforces at the regional, zonal (zone is the middle administrative structure between the region and the district/woreda), city administration special woreda and woreda levels. This followed by setting joint plan at regional level that eventually cascaded down to the hierarchies until it reached the kebele (is the lowest administrative structure) level.

*When we see the structure of the main and sub taskforces, there is one main taskforce from the region to the district level and 9–11 sub-taskforces in each stratum. To mention some of these: health, media and communication, trade, resource mobilization, peace and security, sanitation and hygiene, agriculture and food security, public services, education etc…*

*With the regard to the collaborative undertakings, we recognized this time strong collaboration is option less. If we do as weak as before, the disease will damage our society. Therefore, by giving attention to the collaborative efforts, we have based our actions on the joint plan and similarly, organized collaborative efforts to accomplish the plan. The joint action helped us to efficiently use the available resources.*

In the stride to prevent and control the disease, several sectors brought together under the main taskforce as stated above. Some of these sectors have notable experiences of collaborative undertakings on cross cutting issues. Some key informants stated that their earlier experiences have helped for the establishment and strengthening of the current collaborative efforts. The key informants’ direct words further echoed this idea as:

*We work in close collaboration with many sectors at zonal level. The same is true both upper and lower levels. In the regard to the prevention and control of this disease, we jointly move out to the community to give information at various gathering places. This includes governmental sectors, university, colleges and civic organizations and religious institutions.*

*The reason why multisectoral approach is necessary for the fight against COVID-19 is clear for us*. *The reason being our sector deals with the most vulnerable group of people who are at highest risk for the disease exposure*. *We made a base on our existing sectoral policy and the directives forwarded from the regional and zonal governments*.

As part of the cascaded joint action plan, each sector has prepared its own plan to tackle the multidimensional burden of COVID-19 disease in their respective sector. For instance, the main taskforce allocated a defined responsibility to the health sector (health bureau) on the health services provision related to the disease. The health bureau structured itself into the Emergency Operation Center (EOC) and assigned and Incident Manager (IM). Several taskforces and technical committees have been established under the EOC.

*The collaborative undertakings within the health sector and other stakeholders as focused on the joint efforts evidenced by the strong functionality of the EOC. The EOC is the center piece of the actions related to the disease prevention and control. On daily basis, EOC follow the undertakings. Starting from the community surveillance to the treatment center actions, all are being assessed, discussed and ways forwarded on anticipating issues.*

Education sector in its part works in close collaboration with the main task force and act as a sector how to materialize the education process by the time when schools were closed due the fear of the disease. They were given a sectoral mandate to prepare school setups for quarantine, isolation, and treatment centers. Another sectoral mandate strictly given for the education sector is how to continue the education process despite the closure of classes.

*Another dimension that symbolizes the need for integrated actions is the presence of multiple taskforces. The main taskforce is mandated to allocate the school for virus positive people to stay. However, schools are under the education department, and it was thus our mandate to furnish the school for the purpose.*

*Following the decision both at the federal and regional level about the necessity of school closure, the education sector is given a mandate to continue education through the virtual means. This has come with numerous challenges. We tried to upload as many books as possible online. At the same time, we established some means to broadcast via the radio and television plasma program. In these manners, we worked closely with the Debub radio and TV enterprise, other sectors, and regional council.*

An issue of food security and ensuring agricultural productivity in the region was strictly given to the agriculture sector. The main taskforce has considered the challenges the disease would impose on this sector. Hence, the sector clearly understood the extent of the damage the disease could cause on the sector and how to tackle such huge burden. The statements from key informants further consolidate this point as:

*The expected burden of the covid-19 pandemic in relation to the agriculture sector is its impact on the production sector*. *It would impact on the transportation of agricultural inputs and deployment of the productive human forces*, *the farmers*, *agricultural development agents and other professionals*.

*With the occurrence of the covid-19 disease, the mandate of agricultural sector further warned by the information of FAO. Added with the occurrence of pastes such as locust and crop disease, the occurrence of covid in turn would further reduce agricultural productivity. Therefore, we have planned to work to compensate for the anticipated challenges by tripling sector productivity.*

By the same token, the bureau of Transport and Road Development works in close collaboration with the bureau of Peace and security and bureau of Health to minimize disease transmission via all transport routs in the region. This is further evidenced by the key informants’ statements as:

*I see the issue of multisectoral collaborative actions in two ways: the first is in its strength and the second one is its weakness. The collaborative undertaking with the peace and security bureau and their respective hierarchies to strengthen the state of emergency is the one that can be sited as a strong one. Corrective action upon those who breach the transport regulation enshrined by the state of emergency are conducted with the traffic police under the peace and security bureau. The collaborative undertaking with the health bureau needs improvement. The health professional assignment at the bus station to screen those with signs and symptoms in collaboration with the others working are not well structured and need further emphasis*

All sectors involved in the taskforce have received sectorial and joint assignments and attempted to effect accordingly. The following key informant information substantiate as:

*Under the canopy of the main task force, the media and communication sub-taskforce here at the regional level works in the provision and dissemination of information aiding for the prevention and control of the disease. We do this in collaboration with relevant sectors media institutions and government communication bureau and its respective branches.*

To ensure the effectiveness of the multisectoral actions; follow-up, monitoring and evaluation of the actions in the region were done in two ways. One is paying physical visit and the other one is virtual events using technology. As stated by the key informants:

*So long as we saw, all taskforce members follow their duties on continues base and monitor and follow it regularly as stated above. The members meet three times a week at the main taskforce and daily on the sub-task force level. By doing so, corrective actions were taken in instant manner.*

The monitoring and evaluation process enables whether the intervention is taking towards goal accomplishment or any drawbacks hampering thereof. Both as the main and sub taskforces conducted the supervision and monitoring process. Most of the times the monitoring and follow-up processes were done three times a week at regional, zonal, city administration and woreda levels by the main taskforces.

*We have a platform to follow the interventions at the zonal and special woreda levels both at the main and sub taskforces. To this effect, we have set office telegram page via which all posts their daily report. We in turn evaluate the actions based on the report and provide feed backs. By doing so, we ensure the multisectoral action for the prevention and control of the disease.*

*With the regard to the monitoring and follow–up, at the sub-ask force level, we gather every week and discuss on the matter, its accomplishment and forward on the way to act. All the team members, whether they move for sectoral, or COVID-19 issue to the lower hierarchies; they oversee the issue of covid-19 activities and pass their observation for us to jointly evaluate. They also give feedbacks on site. We communicate with our sectors at the zones and special woredas via the zoom and telegram to exchange reports related to the covid-19 interventions.*

Several limitations were observed in relation to the intervention of MSA. These are evidenced by the direct words from the key informants as follows:

*In my view, the current strength of the multisectoral approach across the region is not in congruence to the level of the problem. I can say that we had better commitment and actions at early stage but now for unclear reasons, the efforts are shrinking.*

*Despite continuous provision of health information to prevent and control the disease, the level of public awareness and their preparedness to cope with the problem remains rudimentary. People are not seriously taking the matter. Yet, they stand together in crowded manner in social setting. Hand washing is not being practiced in the way it has to be. Public ceremonies are still in place. They worship together. These all show how discrepantly people are acting in the contrary to the established disease prevention and control ways.*

*There is no clear evidence that the approach is leading to the intended goal. Though the public has information on the means of transmission and its eventual effect, it found to be reluctant to implement the principles in the day-to-day life, according to the informant. Thus, actions and public behaviors are favoring the expansion of the pandemic regardless of support and supervisions.*

Key informants mentioned their observation in that the early-stage enthusiasm of working together in collaborative and coordinated manner at the main and sub taskforces. However, the practices gradually fade as the time extends. It was manifested that the acting partners, started to stagger their legs in walking together.

*The efforts first started with great worries and enthusiasm but eventually most people including top leaders started slide away. In our region, other competing agenda such as new regionals governance structures request out computing the current public issue. Moreover, negligence was rampantly exhibited by most portion of the society.*

The separate sectoral approach eventually started dominating the integrated multisectoral approach, thus, each sector moves back to act separately. This is further evidenced by the statements from the key informants as:

*My opinion in the overall collective undertaking on how to identify the most at risk group of population seems weak. For instance, those who work in areas of public transportation are at high risk. Their very livelihood nature, collective gatherings and assignments, lack of information posit them at heightened risk. However, the efforts in addressing these groups look shallow. No adequate protective materials are being distributed to them. I, therefore, call up on all stakeholders to give priority to this group of people.*

*One of the challenges for the multisectoral collaboration is the unnecessary interference of individuals instead of the agreed taskforce related joint actions. This is a sign to what extent some individuals are influencing group actions. The overlapping of actions and individual interference are difficulties weakening the collaborative actions. Some decisions are not well communicated to the main taskforce members. Two or three of the task force members are passing high level decisions without the full consultation. Such and such issues do not signify the proper function of multisectoral collaboration.*

The recent lack of attention among the general public and the leaders in the control and prevention of the disease was seen as the shortcoming. This is evidenced by the key informants’ direct words as follows:

*The monitoring and evaluation process eventually getting weaker to the extent that creating hopelessness. While the disease is escalating, the patterns of population to the disease exposure are further complicating. The initial worries are fading and youth portion of the population boasting as the disease is artifact one and does nothing on them.*

*Despite continuous provision of health information to prevent and control the disease, the level of public awareness and their preparedness to cope with the problem remains rudimentary. Yet, they stand together in crowded manner in social setting.*

Sectoral issues took upper hands than the collaborative undertakings. As this is substantiated by the key informants’ statement as:

*Despite the consolidated joint plan and agreed initiation, there were some lags from members of the taskforces. These were revealed at some district levels. Particularly, some district resource mobilization sub-taskforce did not clearly understand and slowed their efforts to match the demand of the pandemic.*

## Discussion

This study was conducted with the purpose of exploring the patterns of multisectoral actions to combat the deadly disease, COVID-19 in SNNPR, Ethiopia. The study was guided by a convergence framework that elucidates the patterns of multisectoral collaboration; how it is structured, where and how the collaborative undertakings function well and where and how it was not [33].

Hence, discussion entails the major findings in line with the aim of the study.

### Pattern of the multisectoral approach in the fight against COVID-19 pandemic

COVID-19 is a multifaceted problem affecting almost all life dimensions of mankind. Consequently, solutions to combat its effects should be commensurate with the degree of their impact [23,43] In connection to this, the study finding indicated that there are increased stakeholders’ convictions in seeking possible ways to tackle the problem. It was stated that consolidated joint efforts must be put in place to prevent and control its impact. Global experiences clearly indicated that few countries with successful interventions were those who properly utilized multiple sectors. Setyawan and Lestari (2020) and other related studies support this finding that comprehensive and holistic actions are critical to revert gross public problems such as COVID-19 [23,44]. In similar manner the study in China also recommended that better coordination, cooperation, and strong solidarity are crucial to fight COVID-19 pandemic [5].

Experience from the Federal Government of Ethiopia guided SNNPR to promptly intervene the prevention and control of the pandemic. The study unveiled that SNNPR government has recognized the importance of proper collaboration, coordination, and integration of efforts to prevent and control the pandemic Multisectoral collaboration can be effective when the engaging stakeholders do share the resources for the stated purpose. The convergence framework for the effective multisectoral action guides that on the top of other aspects, resource sharing and proper handling is crucial for its proper function [34,35]. However, the study finding showed daunting challenges across the hierarchies. One view is that resource shortage could put the region on increased vulnerability, thus, a collaborative undertaking would have better options. On the other hand, there is a thought that the disease is a fictious one propagated due to political interest. Hence, no need to invest huge resource, which can otherwise be repurposed for job creation.

The contrasting thoughts would create challenges in the overall pandemic handling, therefore, should be seen cautiously. The rapid disease transmission across the entire world including our country and the in well managed pattern region, leveraged all stakeholders to lend hands and work in close collaboration. The finding of this study closely resembles with the study by Martin- Rodriguez, Beaulieu, D’Amour & Ferrada-Videle, 2005 and Schewartz & Yen Muh-Yong, 2017., indicating that any problem such as pandemic diseases need collaborative undertakings [22,24].

This study has shown that previous intersectoral collaborative experiences in some organizations have helped for the establishment and strengthening of the current efforts. In connection to this finding Koplan, Butler-Jones, Tsang & Wang; Chen, et al,2020; have mentioned that the collaborative undertakings of experienced stakeholders had been added values in the prevention and control of public health problems such as SARS [45].

This study has revealed that the sectoral plans were integrated and cascaded from the aggregate core plan. Each sector prepared its own action plan to tackle the multidimensional burden of COVID-19 pandemic in their respective sector and revealed plan interface in their intervention how to prevent and control the spread of the deadly pandemic. The approach abetted sectors to execute their defined responsibilities in a coordinated manner and minimized the overlapping and gapping in the efforts to prevent and control the disease. The finding is supported by the working document of the WHO, FAO and OIE, which dictate that the multisectoral one health approach created the interface of actions to produce effective, efficient or sustainable outcomes [46]. This is also in line with the study guiding framework, the convergence model, which states that multisectoral approach varies based on the purpose it wants to accomplish. When the issue needs to complete integration, all the participating entities join to the extent each loses its early identity. They also can function by working independently to full integration [34,35].

The top leaders’ commitment in the intervention of multisectoral approach is irreplaceable [47]. This study has come with the finding that top leader at regional, zonal, city administration and woreda levels have been playing exemplary roles. These were evidenced as they moved into the community to disseminate information about the disease: the burden it could cause, and how to limit its progression and control in as short time as possible. They have exercised exemplary roles and symbolized the multisectoral actions in the prevention and control process of COVID-19 disease. This echoes with the study conducted by Salunke & Kumar, that expressed leadership willingness and policy mandate as the necessary conditions and driving factors for accomplishment of multisectoral actions for a given project or program. [22,26,44]. Moreover, the finding aligns with the study’s guiding framework that states effective collaboration grows over the continuum of the collaborative framework from the lower levels with the minimum networking and at the higher almost as complete integration. Yet, the pattern is unclear when strictly considering the framework, except that the top leaders moved physical but not well elucidated the pattern of integration as whole [34,35].

This study has elucidated the existence of active involvements of numerous organizations and sectors. Of them some desire special notification are the youth volunteer group, teachers’ association, various organizations of youth and women engagements and contribution in the fight against the disease, signifying of the collaborative actions They have engaged in information dissemination, resource mobilization, temperature check and related activities that help in the stride against the disease prevention, witnessing their multidimensional efforts. This study finding is in close resemblance with the directives of the UNICEF. Both have stated that adolescents in the fight against COVID-19 should not only be considered as affected population but also as highly effective partners. They are meaningfully engaged to be educators and change agents. Their volunteer actions in hand washing campaigns countering miscommunication and stigma in their communities are critical contributions to tackle the problems [48]. The positive efforts of some youth and other population sections in this study is an encouraging experience to motivate and influence the opposing youth in the efforts to tackle the problem.

#### Monitoring, evaluation follow up and support process in the endeavors of the multisectoral actions

Plan for the joint efforts for the COVID-19 disease prevention and control needs close monitoring and follow up whether all the interventions are leading toward the set goal accomplishment [49]. The study explored whether the interventions of the planned activities to combat the COVID-19 pandemic in the region leads toward the desired goal. As indicated in the study; supportive supervision, follow-up, monitoring and evaluation of the collaborative efforts in the process of COVID-19 prevention and control have exhibited important perspective. Unlike the previous approach, contemporary technology aided monitoring and evaluation has been carried out. Travelling to reach out into the zones and special woredas were minimized due to the disease effect. Instead, the virtual meetings through video conferences, zoom meeting, telegram-based information exchange became wonderful alternatives bringing stakeholders close in touch. The study has indicated that the monitoring and evaluation processes have helped to take corrective actions on the identified weakness. The whole monitoring and evaluation process were conducted jointly by main and sub taskforces. Equally important in the monitoring and evaluation process was sectoral based supportive supervision, follow-up and evaluation as aligned with the taskforce actions. Most of the times the monitoring and follow-up processes were done three times a week at regional, zonal, city administration and woreda levels by the main taskforces with some degree of staggering. The approach in this study is in line with the WHO and FMOH guidelines and working directives [50–53].

### Limitation observed in the due course of the multisectoral approaches intervention, monitoring and evaluation?

Despite the encouraging attempts to combat the COVID-19, drawbacks were observed at the intervention of multisectoral collaborative actions [53]. The study has indicated that the following limitations of MSA interventions, follow-up and monitoring: failure to meet three times a week as set earlier to discuss on the intervention issues at all level taskforces; shifting attention out of the highly public emergency (the COVID-19) to other local issues; critically low levels of public awareness and participation despite provision of consistent information; failure to stick to measures such as home stay to limit the progression of disease at the early stages due to various reasons; failure to use preventive measures such as face mask while moving outside one’s home and in various public gatherings and inability to afford mask regularly can be cited among the many.

The nature of the multisectoral action itself is another challenge in the effect of the process. As rightly indicated in the study, there has been eventual slowdown on the initial enthusiasm of working together in collaborative and coordinated manner at the main and sub taskforces. It was manifested that the acting partners, started to stagger their steps of walking together. This is in line with the study conducted by Bennett, Glandon and Rasanthan (2018) indicating that unless proper governance of multisectoral actions put in place, the likely slowdown of the progress of such approach is inevitable [47].

The separate sectoral approach eventually started dominating the integrated multisectoral approach, thus, each sector turned back to act separately. Failure to accomplish the agreed joint plan and submitting exaggerated or false reports just to please the bosses, lack of personal protective materials and failure to properly use the personal protective materials, are among the limitations mentioned in the study. In addition, the unfavorable attitude and practice of communities at various levels in the process of the disease prevention and control have been challenging the joint actions. The observed difference in this regard from person to person and place to place, from urban to rural, some misunderstanding such as the disease is only for the urban or developed nation have been the bottleneck in the prevention and control action [47,48,54]. Moreover, lack of properly directed multisectoral approach as indicated in the convergence model can be referenced among the reasons for its desired functions [33–35].

The recent lack of attention among the public and the leaders in the control and prevention of the disease was seen as the shortcoming. Surprising about this issue is that failure of most stakeholders to stick to the prevention and control strategies albeit the provision of consistent and continuous information about the nature and the impact the disease. Home stay has been one of the strategies to limit disease prevention and control, but people avoid this as they choose to die by the disease than by hunger. Thus, they moved out to work and gain some money to fulfill their necessities. This created another worry as they would further fuel the disease propagation. Studies in Ethiopia have substantiated this finding that inadequate acquaintance of the public and overlapping duties have been some of the drawbacks in the proper interventions of the COVID-19 prevention in the country [26,43].

The resource mobilization and utilization processes had faced another challenge. Lack of common understanding on this issue: failure to execute in accordance with the financial regulation, youth requests of this resource other than the disease prevention and control and diverting the disease existence as an artifact one as if politically motivated. Some youth lack interest and gut to feel the disease is a real one affecting all dimensions and people are the added challenges.

As has been seen in the study that instead of doing things in the agreed up on joint undertakings, joint intervention, joint monitoring, and evaluation few taskforce members interested to reveal their stamina by putting stakes in superior position. This has created some dissatisfaction in collaborating stakeholders and weakened the intersectoral collaborative efforts. WHO report for the European region mentions that the failure to identify co-benefits to act in win-win situation, lack of smooth communication among the collaborating partners, lack of mutual trust and understanding are among the barriers for the successful intervention of multisectoral collaboration [53]. The lack of clear accountability and diffusion of responsibility created a spasm in few cases [47,48,55]. This study is in conformity with the above studies and reports. The study conceptual framework guidance and the finding of this study has some levels of conformity and discrepancies across the continuum [34,35].

#### Limitation and strength of the study

The strength of this study is as it dealt with the very current issue and that is being a concern of all national and global citizens, the timeliness of the study, involvement of as many sectors as possible are some of the strengths. The limitation may be the use of only qualitative method limits its generalizability.

## Conclusion and recommendation

This study was conducted with the broad aim of exploring the patterns of the multisectoral approach in the fight against COVID-19 pandemic in SNNPR.

The study has clearly indicated that firm understanding of the multidimensional impacts of COVID-19 in the region was observed by all stakeholders. Consequently, the regional government and its lower-level administrative hierarchies gave strong priority attention to prevent and control the pandemic through consolidated collaborative efforts of all concerned stakeholders. High level leaders of the region (region to the district) played exemplary roles by organizing structures that would respond to the problem at hand. The structure and function of the task forces have enhanced the response processes by establishing strong vertical and horizontal links across the multiple sectors. This has paved ways to promptly access information, resources, and mechanism of actions. It further helped to revitalize the customary beneficial assets of the society during the humanitarian shocks such as helping each other by sharing what is available at hands.

However, the efforts of multisectoral approach were not implemented as indicated in the study guiding frame with proper functional continuum. Accordingly, several setbacks were observed in the process of multisectoral approach intervention. Monitoring and evaluation of the joint actions exhibited irregularities in conducting meetings as planned and commitments staggered overtime. Shifting of attention and moving back to the status quo to the sectoral dominated intervention were also seen.

In order to make the MSA a sustainable means of responding to the COVID-19 pandemic and beyond, it is recommended that: the initial enthusiasm and commitment should be reiterated; monitoring and evaluation process of the MSA should be strengthened taking the end-to end approach of the convergence frame; subsequent quantitative study should be conducted to generate evidences on the wider scopes of the determinants on the successful intervention of MSA.

## Supporting information

S1 File Study participants’ profile. (DOCX)

S2 File Summarized English version key informant interview transcript. (DOCX)

S3 File Local language detail transcription. (DOCX)
