# Supplementary material for: Exploring the patterns of multisectoral approach in fighting COVID-19 Pandemic in SNNPR, Ethiopia: A qualitative case study approach
Source: PLoS One. 2022 Feb 25;17(2):e0263667. doi: 10.1371/journal.pone.0263667 (PMC8880945; doi:10.1371/journal.pone.0263667)
Supplement: S1 File — (DOCX) [file pone.0263667.s001.docx]

| **S.No.** | **Institution of the Key Informant** | **Age** | **Sex** | **Position** | **Year of service on the position** | **Education** |
| --- | --- | --- | --- | --- | --- | --- |
| 1 | Ethiopia Red Cross Society, SNNPR Branch Office | 40 | M | Regional Managers | 3 | MA |
| 2 | Regional Teachers Association | 41 | M | Chairman | 5 | MA |
| 3 | WCYA Bureau | 34 | F | Head | 2 | BA |
| 4 | Education Bureau | 39 | M | Head | 2 | PhD |
| 5 | Finance Bureau | 45 | M | Head | 2 | Msc |
| 6 | South Radio & TV | 34 | M | Head | 4 | BA |
| 7 | Agri & NR Bureau | 41 | M | Head | 10 | MSc |
| 8 | Health Bureau | 42 | M | Head | 7 | MHA |
| 9 | Transport and Road Dev. Bureau | 37 | M | Head | 2 | MA |
| 10 | Water, Irrigation and Energy Bureau | 36 | M | Acting Head | 4 | Msc |

Additional file 1. Profiles of the Key Informants: a. Regional Center KI’s Profile

| **S.No.** | **Institution of the Key Informant** | **Age** | **Sex** | **Position** | **Year of service on the position** | **Education** |
| --- | --- | --- | --- | --- | --- | --- |
| 1 | Office of the Zonal Administration | 47 | M | Office Head | 2 | MA |
| 2 | Government communication | 39 | M | Head | 1 | MA |
| 3 | WCYA | 34 | M | Deputy | 1 | MA |
| 4 | Education Department | 36 | M | Head | 8/12 | MSC |
| 5 | Finance Department | 35 | M | Head | 4 | Msc |
| 6 | LSA department | 59 | M | Deputy | 5 | BA |
| 7 | Agri & NR Department | 38 | M | Head | 1 | BSc |
| 8 | Health Department | 40 | M | Head | 4 | MPH |
| 9 | Transport and Road Dev. Department | 40 | M | Head for road safety | 3 years | BA |

b. Gammo Zone KI’s Profile

**c. Gedeo Zone Key Informants’ profile**

| **S.No.** | **Institution of the Key Informant** | **Age** | **Sex** | **Position** | **Year of service on the position** | **Education** |
| --- | --- | --- | --- | --- | --- | --- |
| 1 | Zonal Chief Administrator’s Office | 30 | M | Chief Administrator | 2 years | BA |
| 2 | Department of Agriculture and NRD | 35 | M | Head | 3 years | MA |
| 3 | Department Urban Development and Construction | 36 | M | Head | 2 and half years | MA |
| 4 | Women, Children and Youth Affairs | 40 | F | Head | 2 years | BA |
| 5 | Department of Transport and Road Development | 33 | M | Deputy Head | 4 years | BA |
| 6 | Department of Finance | 36 | M | Head | 3 and half years | MA |
| 7 | Department Labor and Social Affairs | 40 | F | Head | 3 years | MA |

d. Wolaita Zone KIs profile

| **SN** | **Position** | **Number of years on the position** | **Level of Education** | **Age** |
| --- | --- | --- | --- | --- |
| 1 | Department Head of City Development and Construction | 1 year | MA in Education | 40 |
| 2 | Head of the Department of Health | 2 year | MSC in Pharmacy | 39 |
| 3 | Department Head of Finance | 2 years | MA in accounting | 40 |
| 4 | Department Head of Transport | 2 years | BA in Education | 48 |
| 5 | Department Head of Peace and Security | 2 years | BA in management | 50 |
| 6 | V/Head of the department of  Government Communications | 1 years | BA in management | 38 |
| 7 | Department Head of  Education | 2 years | MA in English | 40 |
