# Supplementary material for: Exploring the patterns of multisectoral approach in fighting COVID-19 Pandemic in SNNPR, Ethiopia: A qualitative case study approach
Source: PLoS One. 2022 Feb 25;17(2):e0263667. doi: 10.1371/journal.pone.0263667 (PMC8880945; doi:10.1371/journal.pone.0263667)
Supplement: S2 File — (DOCX) [file pone.0263667.s002.docx]

**Additional file 5.**

**Title: Exploring the patterns of multisectoral approach in fighting COVID-19 pandemic in SNNPR, Ethiopia: A qualitative case study approach.**

Abraham A Ali^1^ PhD, [alanoabraham@yahoo.com](mailto:alanoabraham@yahoo.com)

Akmel M Usman^1^ PhD, [abukelsuma09@gmail.com](mailto:abukelsuma09@gmail.com)

Fekadeselassie B Badebo ^1^ MSc, [fekade93@yahoo.com](mailto:fekade93@yahoo.com)

Solomon H Tilahun^1^ MA, sohadi2005@yahoo.com

**^1^ Policy Study and Research Institute, SNNPR**

**Local language transcription**

Gammo Zone

***Profile of the Key Informants***

| **S.No.** | **Institution of the Key Informant** | **Age** | **Sex** | **Position** | **Year of service on the position** | **Education** |
| --- | --- | --- | --- | --- | --- | --- |
| 1 | Office of the Zonal Administration |  | M | Office Head |  | MA |
| 2 | Government communication | 39 | M | Head | 1 | MA |
| 3 | WCYA | 34 | M | Deputy | 1 | MA |
| 4 | Education Department | 36 | M | Head | 8/12 | MSC |
| 5 | Finance Department | 35 | M | Head | 4 | Msc |
| 6 | LSA department | 59 | M | Deputy | 5 | BA |
| 7 | Agri & NR Department | 38 | M | Head | 1 | BSc |
| 8 | Health Department | 40 | M | Head | 4 | MPH |
| 9 | Transport and Road Dev. Department | 40 | M | Head for road safety | 3 years | BA |

**ቁልፍ መረጃ ሰጭ አንድ**

የዞኑ ዋና አስተዳደር ጽ/ቤት ከኮሮና በሽታ መከላከልና ቁጥጥር አንፃር ያለው ቁርኝት ምን ይመስላል?

ሌሎችን ስምምነት፣ ከማስተባበር፣ ዝግጅት አንፃር ምላሽ ኮቪድ 19 በሽታ ከሀገር አልፎ ዓለም አቀፍ ወረርሽኝ እንደሆነ ይታወቃል፡፡ በሽታው ከተከሰተበት ጊዜ አንስቶ እስካሁን እንደዞኑ አስተዳደር ከፍተኛ እንቅስቃሴ እያደረግን ነው ያለነው በተለይ በመከላከል ዙሪያ፡፡

ይህንን በዝርዝር ስናይ ዞኑ ላይ ዓብይ ኮሚቴ አለ፡፡ ይህ ዓብይ ኮሚቴ በውስጡ 11 ንዑሳን ኮሚቴዎች አደራጅተዋል፡፡ አጠቃላይ 18 መዋቅር ያቀፈ አራት ከተሞችንና 14 ወረዳዎችን አቅፎ በዞኑ ውስጥ ያሉትን ሁሉ መምሪያዎችን ያቀፈ ነው፡፡ እያንዳንዱ ንዑስ ኮሚቴ አቅዶ የዕቅዱን አፈፃፀም በየሶስት ቀን እየገመገመ በዚሁ መልክ እየፈፀመ ይገኛል፡፡

**ለምሳሌ፡-** እነዚህ ንዑሳን ኮሚቴዎች ፡-

- ሚዲያና ኮሙኒኬሽን፣
- ጤና፣
- ሁከት ማሰባሰብ፣
- ንግድ ዘርፍ፣
- ፀጥታ፣
- ሳኒቴሽን ንዑስ ኮሚቴ፣
- ግብርና ልማት፣
- ማንግስታዊ አገልግሎት ንዑስ ኮሚቴ፣
- በጐዳና ተዳዳሪዎች ላይ የሚሠራ ንዑስ ኮሚቴ፣
- ትምህርት ግብረ-ሀይል፣
- የትምህርት ሥራ ከኮቪድ በሽታ መከላከልና ቁጥጥር ጋር አያይዞ የሚሠራ ንዑስ ኮሚቴ መሆኑ ተብራርቷል፡፡

እነዚህ ንዑሳን ኮሚቴዎች ያቀዱትን እቅዶች ወደ ተግባር ቀይሮ እየሠሩ ይገኛሉ፡፡

ከዚህ ባሻገር በየከተማውና ወረዳዎች ጭምር የሚከናወኑትን ሥራዎች ደረጃ ለዐብይ ኮሚቴ ቀርቦ በየሶስት ቀናት እንገመግማለን፡፡ የዞኑ አስተዳደሪ የዐብይ ኮሚቴ ሰብሳቢ እንደሆኑ የመከላከሉ ሥራ በሰፊው እየሄደ ነው፡፡

በዚህ ሂደት ውስጥ ዞኑ ተጠሪነቱ ለክልሉ እንደመሆኑ መጠን ከክልል ጋር ያለው መስተጋብርና የሥራ ግንኙነት ምን ይመስላል?

ይህ ዐብይ ኮሚቴ ከክልሉ ጋር በቀጥታ ይገናኛል፡፡ ዕቅዱን አዘጋጅቶ ለክልሉ አቅርቦ ክልሉም ዕቅዱን ገምግሞ መከናወን የሚገባቸውን በፌደራል የጤና ሚኒስቴር ሆነ መንግሥት በሚያስቀምጠውና ከዚያ በሚወርደው አቅጣጫ በተለይ በመከላከሉ ዙሪያ ከላይ የወረደውን በራሱ ደረጃ ገምግሞና አደራጅቶ ለወረዳዎች ከተማ አስተዳደሮች በተዋረድ እያወረደ በተግባር እንዲቀየር እያደረገ ነው ያለው፡፡

ክልሉም ላይ ባለው ተመሳሳይ አደረጃጀት የዞናችን ዋና አስተዳደሪ ይሳተፋል፡፡ ከዚያ የሚወርደውን አቅጣጫ ለንዑሳን ኮሚቴዎች በማውረድ የመከላከሉን ሥራ እየሰራን እንዳለ ገምግመናል፡፡

**የኮቪድ 19 በሸታ ጫናና የስጋት ደረጃ በዞኑ እንዴት ታይቷል?**

እንደሚታወቀው የጋሞ ዞን በተለይ አርባምንጭ ከተማ በሁለት አቅጣጫዎች የሚገቡ ሰዎች ስላሉ በጣም ለበሽታው በቅርበት መስፋፋት ሌላ ዕድል ያላት ከተማ ነች፡፡ **ለምሳሌ፡-** በስተደቡብ ምስራቅ በኩል በሞያሌ በኩል ከውጭ በህገ-ወጥ ንግድ ጋር ተያይዞ ከሞያሌ በኮንሶ አድርገው የሚገቡ በርካታ ኮንትሮባንድ ነጋዴዎች አንዱ የስጋት ምንጭ በመሆኑ ይህንን ከፀጥታ ኃይሉ ጋር በቅንጅት የምንሰራበት አግባብ አለ፡፡

ሁለተኛው የጋሞ ዞን በልዩ ሁኔታ የሚገልፀው በዞናችን ደጋማ አካባቢ ከእያንዳንዱ ቤተሰብ በሚባል ደረጃ በርካታ ሰዎች ከአዲስ አበባ ፈልሰው መኖራቸውና ከዚህ በሽታ መከሰት ጋር ተያይዞ ኑሮአቸው ከእጅ ወደ አፍ የሆኑ እና በጉልበት ሥራ የሚተዳደሩ ወደኋላ እየተመለሱ ይገኛሉ፡፡ እንደሰማነው በአዲስ አበባ በሁሉም ክፍለ ከተሞች በሽታው በአስከፊ ሁኔታ እየተዛመተ ነው፡፡ ስለዚህ ከዚህ በቀጥታ በርካታ ሰዎች እየተመለሱ ያለበት ሁኔታ አለ፡፡ ከዚህ ጋር ተያይዞ በሽታው በስፋት እኛ ዞንም ሊከሰት ይችላል የሚል ስጋት አለ፡፡

እስካሁን ተጠርጥሮ ወደ ማቆያ ከገቡት ውስጥ 1 /አንድ ሰው/ ከዳራማሎ ወረዳ ናሙና ተወስዶ ተመርምሮ በሽታው በመገኘቱ በጤና ማዕከል ህክምና እንክብካቤ እየተደረገ ነው ያለው፡፡ ግን በርካታ ጥርጣሬዎች ኖሮ ውጤቱ ነጌቲቭ የሆነበት ሁኔታም ተስተውሏል፡፡ የደም ምርመራ በአካባቢያችን ስለተጀመረ በበሽታው የተጠቁት በርካታዎች ይገኛሉ ብለን እንገምታለን፡፡ እስካሁንም በሽተኛ ወይንም በቫይረሱ የተጠቃ በከተማችን ጠፍቶ ሳይሆን በቂ ምርመራ ባለመደረጉ ነው የሚል እምነት አለን፡፡

ከዚህ ጋር በተያያዘ የበሽታውን ስርጭትና ስፋት በተሻለ ደረጃ ለመዳሰስ እንዲረዳ በአርባ ምንጭ ከተማ የቤት ለቤት ዳሰሳ ተጀምሯል፡፡ የሙቀት መለኪያ እያንዳንዱ ቤት በመዞር መለካትና ምን ዓይነት ጥንቃቄ ማድረግ እንደሚገባ የጤና ትምህርት እየተሰጠ መሆኑ ቤተሰቡን በሙሉ ሙቀት በመለካት አሰሳ መደረጉ፤ ተመሳሳይ ድርጊት ደግሞ በየወረዳዎች እየተሰሩ መሆኑ፤ ሆኖም ግን እንደ ዞን አስተዳደር ከፍተኛ ስጋት አለን፡፡

ከላይ እንደጠቀሱት ለኮቪድ ምላሽ ግብረ-ኃይሎች መቋቋሙን ገልፀዋል፤ በዚህ ደረጃ ግብረ-ኃይል እንድታቋቁሙ ያደረጋችሁ መነሻ ምንድነው?

ዋናው ምክንያት በሽታው ዓለም አቀፋዊ ወረርሽኝ እንደመሆኑ መጠን እኛጋም መድረሱ እንደማይቀር ግንዛቤ በመውሰድና ዞኑና የዞኑ መቀመጫ ከተማችን አርባ ምንጭ ያላት የኢኮኖሚያዊና ማህበራዊ መስተጋብር የሚፈጥረው ትስስር አንፃር በሁለት በኩል ከውጭ ሰዎች ስለሚገቡ በሽታው ከተከሰተ በኋላ ከመሯሯጥ አስቀድሞ መከላከል አስፈላጊ በመሆኑ፤

ከዚህ ጋር ተያይዞ የፀጥታ ኮሚቴ እንደዚህ ዓይነት በሽታ ጋር ያሉት ወይንም የሚጠረጠሩት ዘው ብለው እንዳይገቡ የፀጥታ ኃይሉ ከሚዲያ ጋር በመሆኑ የበሽታው መተላለፊያ መንገዶችንና ሊወሰዱ ስለሚገቡ ጉዳዮች ኀ/ሰቡ አውቀው እንዲተገብሩ የግንዛቤ ማስጨበጫ ሥራ መስራት፣ ሌላው ደግሞ የሀብት አሰባሳቢ ኮሚቴ ችግሩ ከተከሰተ ሊያስከትል የሚችለውን የሀብት ፍላጐት ታሳቢ በማድረግ ሀብትን የማሰባሰብ ሥራ መገኘቱ፣ ሌላው የመከላከል ተግባር አካል የሆነው ኀ/ሰቡ አስገዳጅ ሁኔታ ካልተፈጠረ በቀር ከቤቱ እንዳይወጣ የማድረግ ሥራ ስለሆነ በዚህ ጉዳይ ሊጐዱ የሚችሉ አቅመ ደካማዎች **ለምሳሌ፡-** የጐዳና ተዳዳሪዎች፣ እንጨት ሸጦ የሚተዳደሩ በሽታው ሲከሰት የሚያስፈልጋቸው ባለማግኘት ሊጐዱ ስለሚችሉ ለነዚህ ዓይነት ደካሞች ድጋፍ የሚሆን ሀብት የማሰባሰብ ሥራ ለመሥራትና ችግሩን በጋራ ተጋግዘንና ተደጋግፈን ለመመከት ነው፡፡ እነዚህ ግብረ-ሀይሎች የተቋቋሙት **ለምሳሌ፡-** ንግድን በሚመለከት የዋጋ ንረት እንዳይመጣና ኀ/ሰቡን ላላስፈላጊ የኑሮ ጫና እንዳይጋለጥ የዋጋ ንረት እንዳይመጣና የተረጋጋ ዋጋ እንዲኖር ቁጥጥር የሚያደርጉበት አግባብ ነው ያለው፡፡

በግብርና ላይ የተቋቋመው ግብ-ሀይል አርሶ አደሩ ጤናው ተጠብቆ በቀጣይ የምግብ እጥረት እንዳይከሰት በሰራው የምርት ሥራ ውስጥ እንዲገባ የሚያደርግ ነው፡፡ እነዚህ ግብረ-ኃይሎች ወይንም ንዑሳን ኮሚቴዎች ከኮቪድ 19 ጋር በተያያዘ ከመከላከል ጋር የተያያዘ ሥራ ያከናውናሉ፤ **ለምሳሌ፡-** ከመንግሥት ሥራ ጋር በተያያዘ የተቋቋመው ግብረ-ኃይል ሠራተኛው በቢሮ ሆነው እንዴት ባለ አግባብ ተገልጋይን ማገልገል እንደለባቸው ተገልጋይ ሲገባ ማድረግ የሚገባው ጥንቃቄ በየሴክተሩ በተለይ ደግሞ በፐብሉክ ሰርቪስ የሚመራ በመሆኑ ሠራተኛው ራሱን ጠብቆ እንዴት አገልግሎት መስጠት ይችላል የሚለውን ፈትሾ የሚተገብር ኮሚቴ ነው፡፡ እንደዚሁ ደግሞ ከላይ የተዘረዘሩት ንዑሳን ኮሚቴዎች የየራሳቸው ተልዕኮ አላቸው፡፡ ይህም አንድ ሴክተር ብቻውን ተንቀሳቅሶ ሊወጣ የማይችል ተግዳሮት በመሆኑ ነው፡፡ ሁሉም ባለድርሻ አካላት እየተሳተፋ የሚገኙት፡፡

**እንደ ዋና አስተዳደር /የዞኑ አስተዳደር/ ለዚህ ሥራ ያነሳሳውና መነሻ የሆነ ሠነድ /ወይም የአሰራር ማዕቀፍ ምንድን ነው?**

ይህ በሽታ ከጀመረ ጊዜ ጀምሮ ከሀገር አቀፍ ደረጃ የፌዴራል መንግሥት የራሱ የሆነ ዕቅድ አዘጋጅቶ በዕቅዱ ዙሪያ አቅጣጫ አስቀምጦ ለክልል አውርዶ ክልሉም በዚያው መልክ የራሱን ድርሻ ለግሶ ለዞኖች አወረደ፡፡ ዞኑም በራሱ ተጨባጭ ሁኔታ ቀይሮ ወደ ወረዳዎች አወርደዋል፡፡ እያንዳንዱ ወረዳና ከተማ አስተዳደር የራሱን ተጨባጭ ሁኔታ ቀይሮ ወደ ቀበሌዎች አውርደዋል፡፡ እያንዳንዱ ወረዳና ከተማ አስተዳደር የራሱን ተጨባጭ ሁኔታ /የሰው ኃይል፣ ሌሎች ግብዓቶችን/ ባገናዘበ መልኩ አቅደው እየሰሩ ይገኛሉ፡፡ በመሆኑም ሥራው መነሻ ያደረገው ከላይ ከፌዴራል ሆኖ በየደረጃው ያሉት የሥራ ባለቤቶች በራሳቸው ነባራዊ ሁኔታ በመቃኘት አቅደው እየተባበሩ ይገኛሉ፡፡

ዋና አስተዳደሩ ይህንን ሥራ የሚመራበት የራሱ የሆነ ዕቅድ አለ፤ አፈፃፀሙም በየሶስት ቀናት ይገመገማል፡፡

**ቅንጅታዊ አሠራር የሚመራበት አግባብ ምን ይመስላል?**

በምን ያህል ፍጥነት እንደምትገናኙ፡-

**ለምሳሌ፡-** የጤና ንዑስ ኮሚቴ ከሀይጂንና ሳኒቴሽን ሥራ ጋር በተያዘ እንደ ፀጉር ቤቶች ያሉበትን አካላዊ ርቀትን ጠብቆና የግል ንፅህናን ጠብቆ አገልግሎት መስጠት የሚችሉበት ሳኒታይዘር አጠቃቀም እንዲሁም አጠቃላይ ንጽህና ተያይዞ የሚሠሩ ሥራዎች አሉ፡፡ ሌላው ደግሞ ወደማቆያ የሚገቡ ሰዎችን በሚመለከት ባሁኑ ጊዜ ትምህርት ቤቶች ዝግ በመሆናቸውና ቦታው ለማቆያ እያገለገለ ስላለ ይህ ሥራ ከትምህርት መምሪያና ት/ቤቶች ጋር በቅንጅት እየተሰራ ይገኛል፡፡

ሁሉም ንዑሳን ኮሚቴዎች አንዱ ከሌላው ጋር ተቀናጅተው ነው የሚሠሩት፡፡

**ለምሳሌ፡-** የፀጥታ ንዑስ ኮሚቴ ከትራንስፖርት ንዑስ ኮሚቴ ጋር እየተጋገዘ ይሠራል፡፡ ምናልባት በትራንሰፖርት አጠቃቀም አካባቢ ችግር ከተፈጠረ የፀጥታ ኮሚቴ ገብቶ የማረጋጋትና የማስተካከል ሥራ ይሠራል፡፡

በአጠቃላይ የእያንዱንዱ ንዑስ ኮሚቴ በየሶስት ቀናት እየተገናኙ የደረሰበትን ይገመግማሉ፡፡ ውጤቱንም ደግሞ ለክልል ያሳውቃል፡፡ ይህም ግልጽ የአሰራር ሥርዓት የተበጀለት ነው፡፡

**አጠቃላይ የዚህ እንቅስቃሴ ግብን ከመረዳት አንፃርስ?**

ሁሉም በተደራሽ አካላት ከመጀመሪያው ጀምሮ በሽታውን የመከላከልና የመቆጣጠር ፋይዳ ይኸውም በሽታው ባጭር ጊዜ ውስጥ በቁጥጥር ሥር እንዲውልና የኀ/ሰቡ ሥጋት እንዳይሆን የማድረግ መሆኑን በመገንዘብ ነው እንቅስቃሴ የጀመረው፡፡

**እስካሁን የተመጣበት እንቅስቃሴን ክንውኖች ወደተፈለገው ግብ እያመሩ ስለመሆናቸው ምን ማረጋገጫ አላችሁ?**

ግባችን መከላከል እንደመሆኑ እንደክፍተት የገመገምነው ጉዳይ አለ፡፡ ይኸውም በመጀመሪያ አካባቢ ላይ የነበረው ጥንቃቄ አሁን በተግባር በሽታው እየሰፋ ሲሄድ ጥንቃቄው እየቀነሰ መምጣቱ ነው የሚታየው፡፡ እውነታው መሆን የሚገባው ጥንቃቄው ይበልጥ መሻሻል ነበረበት፡፡ ይህም የሚያሳየው የመላመድ ነገርና በሌላ ጐኑ ደግሞ እቤት ተቀማጩ ለምግብ እጥረት ከመጋለጥ ሰርቼ ላግኝ በማለት ነው፡፡ በብዛት ሰው የሚያስበው ይህም ጥንቃቄ እንዲቀንስና እንዲዳከም አድርጐታል፡፡ ይህ በዚህ ልክ ተገምግሟል፡፡

**ሁለት ኃላፊነት አላችሁ**

**በጋራ ግብረ-ኃይሉን በመምራት የጋራ ግብን መገምገም እያንዳንዱን ተቋም በተልዕኮ ልክ ገምግሞ አቅጣጫ ከመስጠት አኳያ በጉድለትና በጥንካሬ የታዩ ካሉ?**

እስካሁን ባየነው እያንዱንዱ ተቋም የግብረ-ኃይል አባል እንደመሆኑ መጠን በየዕለቱና በየቀኑ እየተገመገመ ስለመጣ ከላይ በገለጽኩት ልክ የታዩ ክፍተቶች ታርሞ ንዑሳን ኮሚቴዎች በተሻለ ደረጃ እያከናወኑ ያሉበት ሁኔታ አለ፡፡

ነገርግን የወረዳዎችን አፈፃፀም በገመገምንበት ወቅት አንዱ ከሌላው በተለየ ሁኔታ የመፈፀም አዝማሚያ ታይቷል፡፡ ቡራቡሬ አፈፃፀም በመጀመሪያ አካባቢ ታይቶ መስተካል የሚገቡ ነጥቦች በዐብይ ኮሚቴ በኩል ተለቅም ተሰጥቷቸው አሁን እየተሻሻለ ያለበት ሁኔታ ነው ያለው፡፡

**በዚህ በሽታ ምላሽ ሂደት ውስጥ በርካታ ተቋማትን አቀናጅቶ በመምራት ያጋጠማችሁ ተግዳሮት ካለ?**

**የተቋማት ተነሳሽነት፣ አብሮ ከመጓዝ አንፃር**

ዐብይ ኮሚቴ በሥሩ ያሉት ንዑሳን ኮሚቴዎች የተደራጀበት ከበሽታው መከላከልና ቁጥጥር ሥራ አንፃር ሊያበረክቱ ከሚችሉት አግባብ እንደሆነ ቢታይም በአንዳንድ ኮሚቴች እንደ ሀብት ማሰባሰብ ን/ኮሚቴ የተወሰኑ መንጠባጠቦች ይታያሉ፡፡ ይህ ደግሞ በሂደት እየተስተካከለ የሚሄድባት ሁኔታ አለ፡፡ በተለይ በወረዳዎች አካባቢ ሀብትን ከማሰባሰብ ረገድ አንዳንድ ወረዳዎች ስራ መንጠባጠብ መክፈት ብቻ ሳይሆን እንዲያውም የበሽታው ችግር ቢከሰት በምን መልኩ ይከላከላሉ ወይንም ይቋቋማሉ እስከሚባል ክፍተቶች ተከስቶ እንደነበር፣ በተለይ ኳራንታይን አካባቢዎችን ለይቶና ለዓላማው ምቹና ብቁ አድርጐ ከማዘጋጀት አኳያ ክፍተቶች ታይቷል፡፡ ትምህርት ቤቶች ተዘግተዋል ይባልና ድንገት የተጠረጠረ ሰው ቢገኝ ገብቶ ምን ይበላል፣ ምን ይጠጣል፣ ከሳኒቴሽን አኳያም የተለያዩ ሁኔታዎች አንፃር አለመመቻቸቶች እንደተከሰተ ከማይመቹ አካባቢዎች ወደ ሚመቹ አካባቢ ለማዞር እኛ እራሳችን የተባሉትን ቦታዎች ሄደን አይተናል፡፡ ውስንነቶች ነበሩ ነገርግን እነዚህን ውስንነቶች እንዳሉ **ለምሳሌ**፡- በዞኑ የህክምና ማዕከል ባለሀብቶች ሰጥተው እነዚህ ማዕከላት ለህሙማን ተገቢ አገልግሎት እንዲሰጡ የማደራጀትና የማብቃት ሥራዎች ናቸው የተሰሩት፡፡

ስለዚህ ውስንነቶች ቢኖሩም የተሟላ እንዲሆን የተደረሰበት ነው፡፡ ለዚህም ዞኑ በጀት መድቦ እየተንቀሳቀሰ ይገኛል፡፡

**ይህ አዲስ ክስተት ይዞ የመጣው መልካም ተሞክሮ አለ ወይ? ካለስ ምንድንው?**

በእርግጥም ይህ አዲስ ክስተት ነው፡፡ አዲስ ክስተት ከመሆኑ ጋር ሰው እያሳየ ያለው በተለይ ፈጥኖ ከመገንዘብ አኳያ ጥረት ማድረግ በተለይ ባለሀብቱና ሌሎች ባለድርሻ አካላት የመሳተፍ፣ በተለይ ወጣቱ አዳዲስ ነገሮችን ለመፍጠር የሚያደርጋቸው ኢንዱስትሪያል ኮሌጆች ከንክኪ ነፃ የሆነ የእጅ መታጠቢያ ማሽን ከማዘጋጀት አኳያ ሌላው ደግሞ ገጠር አካባቢ ከአካባቢው ከሚገኙ ቁሳቁሶች የእጅ መታጠቢያ ውኃ የሚሄድበትን መሥመር ገጣጥሞ በመስራት **ለምሳሌ፡-** በሁለት ወረዳዎች ያየነው ከእንሰት ኮባ መሀሉን በመውሰድ ልክ እንደቱቦ በመገጣጠም ውኃው ወደ ረጅም ቦታ በየሁለት ሜትር ርቀት እየገጣጠሙ ገበያ የሚሄዱት እንዲታጠቡ ከምንጭ ወደ መንገድ በማውጣት ማገዝ፣ ይህም በተለይ ወጣቶች አዳዲስ ፈጠራና ሃሣብ እንዲያመነጩ ገፋፍቷል፡፡ በተጨማሪ ሰዎች የግል ንጽህናቸው ከወትሮ በተለየ ሁኔታ እንዲያዩ አድርጓል፡፡ **ለምሳሌ፡-** እጅ መታጠብን እንደ ልምድ መውሰድ፣ ከሠላምታ ጋር ተያይዞ የሚከሰተውን ንክኪ መቀነስ፣የመሳሰሉትን የተማርንበትና የቀሰምንበት ሁኔታ ታይቷል፡፡

**ቁጥር 2 የቁልፍ መረጃ ሰጭ ሪፖርት**

**የዞን ወጣቶች፣ ህፃናትና ሴቶች የተቋሙ ከኮቪድ 19 በሽታ መከላከልና ስርጭት ምላሽ ምን ይመስላል በሚል ለቀረበው፡-**

**መልካም፡-** እኛ እንደሴክተር በባህሪያችን ከባለድርሻ አካላት የያዝን ሴክተር ነን፡፡ ከዚህ ከኮቪድ 19 ጋር በተያያዘ በሽታውን በመከላከል ረገድ ጥሩ ሰርተናል ማለት እንችላለን፤ አሁንም እየሰራን እንገኛለን፡፡ በሀገራችን መጀመሪያ እንደተከሰተ የበለጠ የመደናገጥ ሁኔታ እንደነበርና እንደሴክተርም ተደናግጠን ግን በችግሩ ዙሪያ የግንዛቤ ማስጨበጥ ሥራ ሠርተናል፡፡ የኛ ሴክተር አብዛኛውን የህብረተሰብ ክፍል የሆኑትን ወጣቶችን፣ ህፃናትንና ሴቶች የምናቅፍ እንደመሆናችን ይህም 75 በመቶ የህዝቡ ድርሻ ያለው በመሆኑ ለችግር ትኩረት በመስጠት ከሀገር አቀፍ ጀምሮ በክልል እንዲሁም በዞንና በወረዳ እስከ ቀበሌ ድረስ ለዚህ ምላሽ ለመስጠት የሚያስችል አደረጃጀት ፈጥረናል፡፡ በጐ አድራጊ የሚባል ንዑስ ኮሚቴ አለ፤ እኔ እንደወጣቶች ዘርፍ ኃላፊ የዚህ ኮሚቴ ም/ሰብሳቢ ነኝ፡፡ ዋና ሰብሳቢ የዞኑ የቀይ መስቀል ኃላፊ ነው፡፡ ወጣቶችም ከሌሎች የኀ/ሰብ ክፍሎች ጋር በጋራና በግል ተደራጅቶ ለኀ/ሰቡ የግንዛቤ ማስጨበጥ ሥራ እየሰሩ ነው፡፡ ይህ አደረጃጀት እስከ ቀበሌ ድረስ ወርዷል፡፡ ይህ የወጣቶች አደረጃጀት የግንዛቤ ማስጨበጫ መልእክት ከማስተላለፍ ባሻገር በየአካባቢያቸው /በሰፈራቸው/ እጅ የማስታጠብ ሥራ ማስተባበርና ማከናወን፣ ኀ/ሰቡ አካላዊ ርቀትን ጠብቆ እንዲንቀሳቀስ ማድረግ፣ በተለይ ሕዝብ በሚበዛባቸው አካባቢ ማለትም እንደ ገበያ ቦታ ሌሎች ማህበራዊ ክስተቶች /ኩነቶች በሚከናወንበት ቦታ በመገኘት ይህንን ያስተባብራሉ፡፡ ከከተማ ማዕከላት ተነስቶ ወደ ተለያዩ ቦታ በሚሄዱ ተሽከርካሪዎች ተሳፋሪዎች እጃቸውን ታጥበው እንዲገቡ የማድረግ ሥራ ይሠራሉ፡፡

**የናንተ ተቋም ከኮቪድ ምላሽ ጋር ተያይዞ ያለው አደረጃጀትና ብቃት ምን ይመስላል?**

**ከታችኛው ላይኛው ጐንዮሽ ግንኙነት**

ከክልል የሴቶች፣ ህፃናትና ወጣቶች ጉዳይ ቢሮ ለዚህ ምላሽ የሚሰጥ የተደራጀ ግብረ-ኃይል አለ፡፡

እንዲሁም ከክልል ባጠቃላይ ለኮቪድ ምላሽ የሚሆን የወረደ አደረጃጀት አለ፡፡ የተዋቀረ ኮሚቴ/ግብረ-ኃይል አለ፡፡ ይህ ደግሞ በተዋረድ በዞኑም ተደራጅቷል፡፡ **ለምሳሌ፡-** ከነዚህ መካከል ሀብት አሰባሰብ፣ ቁሳቁስ አሰባሳቢና በጐ አድራጊ ኮሚቴዎች ይጠቀሳሉ፡፡ እኛ አሁን እንደሴክተር የራሳችን አደረጃጀት እስከ ታችኛው መዋቅር ያወረድንበት አለ፡፡ ከሌሎች ጋር ደግሞ በበጐ አድራጊ ንዑስ ግብረ-ኃይል በምክትል ሰብሳቢነት የተዋቀርንበት ይገኛል፡፡ ይህንን የተዋቀረውን አደረጃጀት ለማንቀሳቀስ፣ ለማገዝ፣ የሥራ ድጋፍና ክትትል ለማድረግ፣ ግብረ-መልስ ለመስጠትና ሪፖርት ለመቀበል የሚያግዝ የጋራ Telegram group ፈጥረን እየተንቀሳቀስን እንገኛለን፡፡ ከሁሉም መዋቅር ጋር በየቀኑ የምንገናኝበት እንደ ጋሞ ዞን Gammo Zone youth group against covid-19 የሚል የቴሌግራም ቡድን አለ፡፡ በየጊዜው በዚህ ኘላትፎርም እየተገናኘን የሚያስፈልጉትን የግብዐት ጥያቄ ተቀብለን እናሰራጫለን፡፡ በጎ አድራጊዎች ራሳቸውን እየተከላከሉ ሌሎችን እያገዙ እናደርሳለን፡፡ በርግጥ ከዚህ ከመከላከያው ጋር ጥሩ ጅምር እንቅስቃሴ ቢኖርም በመሀል የመቀዝቀዝ ሁኔታ ታይቷል፡፡ ሰው ወደ መለማመድ፣ መሰላቸት ሁኔታዎች እያጋጠሙን ይገኛል፡፡ በተለይ በገጠር አካባቢ የአምልኮ ጊዜ ማስረዘም ችግሩን አሳንሶና ንቆ ማየት፣ ይህ የፈረንጅ በሽታ ነው በማለት ይታያል፡፡ ይህንን ደግሞ ሁላችንም ውስጥ ገብተን እንደገና አጠናክረን ካልሰራን በሽታው ወደ ገጠር ከተዛመተ ከፍተኛ ችግር እንደሚፈጥር ተገንዝበን ለማስተካከል እየሰራን እንገኛለን፡፡ እስከ ቀበሌ፣ እስከ መንደር ድረስ አስቀድሞ በተዘረጋው የኛ የሴቶች ልማት ቡድን በኩል እነርሱ በራሳቸው ተንቀሳቅሰው የግንዛቤ ማስጨበጥ ሥራ ዳር እንዲያደርሱ ያደረግንበት ሁኔታ አለ፡፡

**ተቋማችሁ በራሱ ከላይኛውና ከታችኛው መዋቅር ጋር የሚያደርገው አካሄድ እንደተጠበቀ ሆኖ ከሌሎች ባለ ድርሻ አካላት ጋር የሚያደርገው ቁርኝትና ቅንጅታዊ አሠራር ምን ይመስል ነበር?**

በእርግጥ የኛ ተቋም ከብዙ ባለድርሻ አካላት ጋር ግንኙነት አለው በሴክተር መሥሪያ ቤት ደረጃ እንኳን ብንወስድ አብዛኛዎቹ የኛ ባለድርሻ አካላት ናቸው፡፡ ከዚሁ ጋር ጐን ለጐን ውይይት አለ፡፡ እያንዳንዱ ሴክተር ደግሞ በራሱ መዋቅር ወደታች እንዲያወረድ ተነጋግረናል፣ ከዚሁ ባሻገር ዞረን በጋራ መረጃ በመስጠት እየሰራን እንገኛለን፡፡ የዞን አመራር በሙሉ ወጥቶ በየከተማው ገበያና ሌሎች ሕዝብ በሚበራከትበት ቦታ በጋራ ሆነን የበሽታው ችግር መከላከያ ዘዴዎችን ጭምር እያስተማርን ነው ያለነው፡፡ የጤና ባለሙያ ምክርና የመንግስትን ምክር እንዲያዳምጡ እያልን እንገኛለን፤ በጐንዮሽ ግንኙነት አንፃር የኛን ሴክተር የማያካትት ቡድንና ኮሚቴ የለም፡፡ በዚህም ከሁሉም ጋር አብረን እንሰራለን፡፡ **ለምሳሌ፡-** የወጣት አደረጃጀቶች አሉ፡፡ እነዚህን የወጣት አደረጃጀትን ካልያዝን ማንኛውም እንቅስቃሴ ውጤታማ መሆን እንደማይቻል በመገንዘብ እስከታችኛው መዋቅር ድረስ የተዘረጋውን አደረጃጀት እንጠቀማለን፡፡ እነዚህ የወጣት አደረጃጀቶች ማህበር፣ ፌዴሬሽንና ሊግ የሚባሉ ናቸው፡፡ ሁሉም አደረጃጀቶች ከኛ ጋር ይሠራሉ፡፡

የሴቶች አደረጃጀቶችም በዚህ ልክ አሉ፡፡ እነርሱን ይዘን ሰንሰለታዊ ግንኙነት ፈጥረንና ሌሎች ደግም የበጐ አድራጐት ማህበራትንም ጨምረን ነው የምንቀሳቀሰው፡፡ በየከተማው ማዕከላት ያለች ከከተማው ባሻገር እስከ ገጠር ወርዶ መረጃና የግንዛቤ ማስጨበጫ ሥራ እንዲያከናውኑ እየተደረገ ነው፡፡ የአስከፊነትና የመከላከያ ሥራ ከመከናወኑ በተጨማሪ የደም ልገሣ ሥራም እየተሠራ ይገኛል፡፡ በዚህ እንቅስቃሴ ሂደት ባንድ ጊዜ ተወጥቶ እስከ 5ዐዐ ዩኒቲ ደም የተሰበሰበበት ሁኔታ ነበር፡፡ ይህም ለተጓዳኝ የጤና ችግር ሕክምና ከፍተኛ ድጋፍ ማድረጉ ያለውን ቅንጅታዊ አሠራር ያንፀባርቃል፡፡ ይህም ጠንካራ ቅንጅታዊ አሠራር መኖሩን ያሳያል፡፡

**ቅንጅታዊ አሰራሩ ጠንካራ መሆኑ በምን ይገለፃል?**

በሁለት መንገድ ሊገለጽ ይችላል፡ አንደኛ የመምሪያ ኃላፊ ከኮቪድ-19 ጋር በተያያዘ የቁሳቁስ ኮሚቴ ሰብሳቢ በመሆን እየሰራች ነው፡፡ እንዲሁም እንደወጣቶች ዘርፍ ኃላፊ የበጎ አድራጐት ኮሚቴ ም/ሰብሳቢ መሆኔ በዚህ ሂደት ኮሚቴ ተዋቅሮ ካሉት ጋር በየሳምንቱ ውይይት እናደርጋለን፡፡ አጠቃላይ ስለሥራው ሂደት እንማከራለን፡፡ እያንዳንዱን መዋቅር በድክመትና በጥንካሬ እየገመገምን ቀጣይ አቅጣጫ እናስቀምጣለን፡፡ ጠንካራ ጐን ያለበትን እያበረታታን ደካማ ጐን ያለውን እንዲያሻሽል እንመክራለን፡፡ ስለዚህ በነዚህ ድርጅቶች ምክንያት ከሥራው ጋር እጅግ የተቆራኘ ግንኙነት እንደተፈጠረ እናስባለን፡፡ ችግሩን በዚህ አግባብ በምክክር ምላሽ እየሰጠን እንገኛለን፡፡

**ቅንጅታዊ አሰራር ከሚረጋገጡበት አሠራር አንዱ የጋራ እቅድ፣ የጋራ ተግባር፣ የጋራ ድጋፍና ክትትል ይገኛል፡፡ በዚህ አንፃር የጋራ ዕቅድ ስለመኖሩ?**

የጋራ እቅድ ከዞን ጀምሮ እስከታችኛው መዋቅርም አለ፡፡ ታችኛው መዋቅርም የራሱን እቅድ እንዲያቅድ አድርገን ነው እየሰራን ያለው፡፡

የጋራ ዕቅድ እንደባለድርሻ አካላት ጥምረትና እንዲሁም በሴክተር ደረጃም የታቀደ አለ፡፡ ይህም ኮቪድ-19 ብቻ በሚመለከት ማለት ነው፡፡ ድጋፍና ክትትል እንዲሁም ግምገማ የምናደርገውም በዚህ ዕቅድ መሠረት ነው፡፡ ስለዚህ እኛ እንደ ሴክተር ሁሉንም የማህበረሰብ ክፍል ለማድረስ ምቹ ሁኔታዎች አሉ፡፡ ቀደም ባሉት በተፈጠሩት የሴቶችና የወጣቶች አደረጃጀቶች እስከ መንደሩ ስላሉ ነው፡፡

**የኮቪድ -19 በሽታ በመምጣቱ የሴቶችና ወጣቶች አደረጃጀት በምን አግባብ ተጠናክሮ እየሰራ ይገኛል?**

የበሽታው አስከፊነት አስደንግጧል፡፡ ሁሉንም ሰሉ፣ አብዛኛው ማኀ/ሰብ ከዚህ በፊት በርቀት የምንሰማው በሽታ ወደየአካቦቢያችን እየተቃረበ ነው በማለት መደናገጥን በመፍጠሩ ያለው አደረጃጀት እንዲጠናከርና አስቀድሞ ተዳክሞ የነበሩትም ፈጥነው እንዲወጡ አድርጓል፡፡ ስለዚህ ሰንሰለቱ የላላበት ሁኔታ ነበር ግን በዚህ አጋጣሚ ተጠናክሮ እንዲወጡት እያደረገ ይገኛል፡፡ የኛ ሴክተር ልዩ ሁኔታ መዋቅሩ እስከ መንደርና ሰፈር የተዘረጋ መሆኑ እስከቤተሰብ ዘልቆ የመድረስ ዕድሉ ሰፊ ነው፡፡ ግን ሄዶ ሄዶ ዋናው ግብ ውጤት ማግኘት እንደመሆኑ ደግሞ ሰው የባህሪ ለውጥ አምጥቷል ብለን ስናይ የሚቀሩ ተግባራት እንዳሉ እናያለን፡፡

የግንዛቤ ማስጨበጫ መልዕክት አልደረሰም ማለት አንችልም፤ ሆኖም ግን የባህሪይ ለውጥ በተፈለገው ደረጃ አልመጣም፡፡ ይህ ሁሉ ተደርጐ ግን አሁን በሽታው እንዳይዝ ከማድረግ አንፃር እያንዳንዱ ሰው ራሱን ከመከላከል አንፃር ያለው ደረጃ ሲታይ ገና አሳሳቢ ነው፡፡ አሁንም ጉድለት መኖሩን ያሳያል፡፡

**እንግዲህ ከዚህ በመነሳት እስካሁን የመጣንበት የሚፈለገው ውጤት ካልመጣ ቅንጅታዊ አሰራሩ እንዴት ተገመገመ?**

ይህንን በሚመለከት ግብረ-መልስ ብንሰጥም ከመጣው ለውጥ አንፃር ስንለካ ከመጨባበጥ አንፃር የባህሪ ለውጥ እስከታች ድረስ የሚታይ ቢሆንም አካላዊ መራራቅን በሚመለከትና ከመሸፋፈን አንፃር በማስክ አንፃር ጥሩ ነገር ቢኖርም በገጠር አኳያ አካላዊ ርቀትን በመጠበቅም ሆነ ከመሸፋፈን አንፃር ምናልባት ከአቅርቦት እጥረትም አኳያ ሊሆን ይችል ይሆናል፡፡ ግን ያሉ ውስንነቶች አሉን፡፡ **ለምሳሌ፡-** በበጎ አድራጐት ሥራ የተሰማሩ ወጣቶች ከክልል ጀምሮ እስከ ታች ድረስ በትንሹም ቢሆን ራሳቸውን እንዲከላከሉ አሰራጭተናል፡፡ሆኖም ግን በቂ አልነበረም፤ ለሁሉም ኀ/ሰብ ክፍል ማድረስ አልተቻለም፡፡ አንዳንዶች ገዝተው ለመጠቀም አቅም የላቸውም በርግጥ የባህሪ ለውጥ የመጣባቸው ያለመጨባበጥ አኳያ ሰዎችን ስለማመን አኳያ ጥሩ የሚባሉ ልምዶች እያዳበሩ ይገኛሉ፡፡ **ለምሳሌ፡-** አንድ ሰው ከከተማ ወደ ገጠር ከሔደ የገጠሩ ሰው ይኸንን ሰው ለመቅረብ የመፍራት ሁኔታ ይታያል፡፡ ነገርግን ይህን ያህል ውጤት አምጥቷል ብለን የምንደመድመው ሳይሆን አሁንም በርካታ ጉድለቶች አሉ፡፡

አሁን በግንዛቤ በኩል ያደራጀነውን ሰንሰለት ተጠቅመን መልሰን መላልሰን መሥራት ይጠበቅብናል፡፡ አንዳድን አካባቢዎች የሚታየው ነገር አንድ ጊዜ ሰርቶ ወዲያውኑ ደግሞ መዘናጋት ይታያል፡፡ **ለምሳሌ፡-** የገበያ አካባቢ ዓይነት፤

**የዚህ ዓይነት ያዝ ለቀቅ ድርጊት ከምን የመነጨ ነው?**

**ከላይ እንደተነጋገርነው በጋራ ታቅዶ በጋራ እየተገመገመ ከተመጣ የዚህ ዓይነት ክፍተት እንዴት ሊፈጠር ቻለ? ችግሩን በጋራ ገምግማችሁ የጋራ ጉድለት ከማድረግ አንፃርስ**

አዎን ይህ የጋራ ግምገማ ነው፡፡ በየደረጃው እየገመገምን መጥተናል ክፍተትንም በጋራ ለይተናል፡፡ **ለምሳሌ፡-**በከተማ አካባቢ ያለው የባህሪይ ለውጥ ከገጠሩ ጋር ተመሳሳይ አለመሆኑ፤ ይህ ማለት ገጠሩ ያለው ኀ/ሰብ እንደከተማው የችግሩን ግዝፈት ያለመረዳት መኖሩን ያሳያል፡፡ እንደሴክተርም እንደጋራ ኮሚቴም የገመገምነው ጉዳይ ነው፡፡ እንዴትስ መሻገር እንችላለን ብለንም በጋራ ተወያይተን በቀጣይ በክረምት የወጣቶች በጎ አድራጐት ሥራ ትግበራም አካትተን ለመተግበር የተግባሩ ዋና አካል የኮቪድ-19 በሽታ እንደሆነ አካትተናል፡፡ እስካሁን የመጣንበት እንደተጠበቀ ሆኖ ግን ይበልጥ በቀጣይ አጠናክረን ለመቀጠል ዕቅድ ክለሳ አድርገናል፡፡ **ለምሳሌ፡-** ከክረምት የችግኝ ተከላ ጋር አቀናጅተን የግንዛቤ ሥራ ለማጠናከር እየተሯሯጥን እንገኛለን፡፡

**የናንተ ሴክተር በጋራ ዕቅድ ውስጥ ሁለት ተለይቶ የተሰጣችሁ ተግባሮች አሉ፡፡ አንደኛ የሀብት ማሰባሰብና የበጎ አድራጐት ሥራ፤**

**ሁለቱን ተግባራት በበላይነት ከመምራት አንፃር የሀብት ማሰባሰብ ሥራ ሂደት እንዲሁም የተሰበሰበውን ሀብት በአግባቢ ጥቅም ላይ እንዲውል ከማድረግ አንፃር ያለው ቅንጅታዊ ሥራ፤**

ከሀብት ማሰባሰብ ጋር ቀደም ብለው እንደገለጹት ሶስት ዓይነት ንዑሳን ኮሚቴዎች አሉ፡፡ የሀብት ማሰባሰብ፣ የቁሳቁስ ማሰባሰብና የበጐ አድራጐት ኮሚቴ ናቸው፡፡ ይህ ኮሚቴ በዋናነት በዞኑ አስተዳደር የሚመራ ነው፡፡ ስለዚህ የተሰበሰበውን ሀብት ለሁሉም የማዳረስ ሥራ ተሰርቷል፡፡ የሀብት ክፍፍሉ ለኀ/ሰቡ እንደየጉዳታቸው ደረጃ በማከፋፈል በጣም ለተጎዱት ቅድሚያ በመስጠት የሚከናወን ነው፡፡ ከበሽታው ጋር ተያይዞ የተጎዱት ክፍሎች **ለምሳሌ፡-** ያህል የጎዳና ተዳዳሪዎች፣ ወላጅ አልባ ሕፃናት ለችግር የተጋለጡት እንደ ሴተኛ አዳሪዎች ለነርሱም ተደራሽ እንዲሆን ተደርጓል፡፡ ይህም መረጃዎች ከታችኛው አካል በማሰባሰብ ጭምር ነበር፡፡ በገንዘብ የተሰበሰበው ሌሎች ቁሳቁሶች በማቆያ ላሉት እንደፍራሽ፣ አንሶላ የመሳሰሉት ለአገልግሎት የሚሆኑ ለሁሉም እንዲደርሱ ጥረት እየተደረገ ነው፡፡ ከገንዘብም ሆነ ከቁሳቁስ አንፃር ብዙ ተሰብስቧል ማለት ይቻላል፡፡ ከህዝቡ ስፋት አንፃር ሁሉም ጋር ደርሶ ያስታግሳል የሚል ድምዳሜ መድረስ ባይቻልም ቢያንስ ጊዜያዊ ማስታገሻ ተሰጥቷል ማለት ይቻላል፡፡

**እየተደረገ ያለው ትግበራ ወደ ታለመለት ግብ እያመራ ስለመሆኑ ምን ማረጋገጫ አለ?**

የጀመርነውን አጠናክረን ከሄድን ውጤት ይኖራል ካልን እኛ ምን አለን ነገርግን ማስተካከል የሚገቡን ነገሮችም አሉን **ለምሳሌ፡-** ከቁሳቁስ አንፃር መጠቀም የማይችሉ መድረስ ያልተቻለባቸውን የኀ/ሰብ ክፍሎችንም ጭምር በተለይ ሳኒታይዘርና የፊት ማስክና ጭንብልም አንፃር ከፍተኛ ክፍተት አሉ፡፡ ስለዚህ እርስ በርስ ተረባርበንም ከዚህም ከዚያም ያገኘነውን ቁሳቁስ ታችኛው መዋቅር ብናደርግም ከዚህ ከችግርተኞች ማ/ሰብ ብዛት አንፃር ብዙ አሁንም ድጋፍ ያስፈልጋቸዋል፡፡ እንደመንግስትም ድጋፋ ማድረግ አለብን፡፡ አሁንም ቢሆን የመከላከል ሥራ አጠናክረን መሥራት ያስፈልጋል እንላለን፡፡ ይህ የመከላከል ሥራ በዋናነት ከቁሳቁስ አቅርቦት በተለይም የሳኒታይዘርና የፊት መሸፈኛ ጭምብል አንፃር የሚሠራ ስለሆነ እነዚህንና የመሳሰሉትን ቁሳቁሶችን ተደራሽ ከማድረግ አንፃር ሰፊ ሥራ መስራት ያስፈልጋል፡፡ ሳይደከም እስከመጨረሻ ድረስ መሄድ ያስፈልጋል፡፡ ነገርግን ባንዳንድ ቦታዎች ቅድም ባልኩት ልክ መዘናጋት ስለሚታይ እነዚህን እንደገና አጠናክረንና አበረታተን መቀጠል ያስፈልጋል፡፡ ከላይኛው መዋቅር ማለትም ከክልል ጋር ያለውን ከፌዴራል መንግስት ጋር ያለው ቁርኝት አጠናክረን መሄድ ያስፈልጋል፡፡

**ካልክ አይቀር እስካሁን በነበሩት የጋራ መድረኮች ከክልሉና ከፌደራል በዞን ደረጃ ባሉት የጋራ ኮሚቴዎች ጋር የሚደረገው የክትትልና ግምገማ አካሄድ በምን ያህል ድግግሞሽ እየተከናወነ ነው?**

ከክልል መዋቅር ጋር በየሳምንቱ በቪዲዮ ኮንፍረንስ እናደርጋለን፡፡ ከዚህ ውጪ በዙም አኘልኬሽን በመጠቀም ውይይት እናደርጋለን፡፡ በቢሮ ቁጭ ብለን ርቀታችንን ጠብቀን ውይይት እናደርጋለን፡፡

**የኮቪድ-19 በሽታ በመከላከልና በመቆጣጠር ሂደት የተገኙ መልካም ተሞክሮዎች አሉ ወይ? ካሉስ እንዴት ይገለፃሉ?**

ጥሩነቱ እንግዲህ ህዝቡ በአንድነት ተነሳስቷል ማለት ይቻላል፡፡ ምንም ልዩነት ሳይኖር፣ የባህል፣ የሃይማኖት ልዩነቶች ሳይነፀባረቅ ሁሉም ባንድ ላይ ለመታገል መነሳሳቱ አንዱ መልካም ተሞክሮ ብለው መውሰድ ይቻላል፡፡ አንድነት ፈጥሯል፣ ልዩነትን በማጥበብ ችግሩን ለመመከት በጋራ የመቆም ዕድል ፈጥሯል፡፡ የሃይማኖት ተቋማት ባንድነት ወጥቶ የፖለቲካ ልዩነት ሳይጠብቅ እነዚህ ተፎካካሪ የፖለቲካ ፖርቲዎች ባንድነት ወጥቶ በሽታውን የመታገል፣ ከቁሳቁስ ድጋፍ፣ ከደም ልገሳ አንፃር ቢያንስ ህዝቡ ባንድነት ይህንን አስከፊ ጊዜ ተባብሮ ለማለፍ እየታገለ ይገኛል ማለት ይቻላል፡፡ ስለዚህ ይህ የርስበርስ ትብብርና የጋራ መከላከል ግንባር መፍጠሩን ያረጋግጣል፡፡

**ቁልፍ መረጃ 3 መንግስት ኮሙኒኬሽን መምሪያ**

**የዞን መንግስ ኮሙኒኬሽን መምሪያ የኮሮና ቫይረስ በሽታ መከላከልና ቁጥጥር ሥራ ጋር ያለው ቁርኝት ምን ይመስላል?**

የጋሞ ዞን ኮቪድ-19 መከላከል ኮሚቴ በሥሩ 1ዐ ንዑሳን ኮሚቴዎች አሉት፡፡ ከዚያ ውስጥ አንዱ ሚዲያ እና ኮሚኒኬሽን ንዑስ ኮሚቴ በመምሪያችን የሚመራ ነው፡፡ ይህ ቡድን የሕዝብ ግንኙነት ሥራ ነው የሚሠራው፡፡ ይህ ንዑስ ኮሚቴ ሌሎች ንዑሳን ኮሚቴ በየዘርፍ የሚሠራቸውን እየተከታተሉ ለኀ/ሰቡ ያደርሣል፤ ዘጠኙ ንዑሳን ኮሚቴዎች የሚሠሩአቸው ሥራዎች አሉ፡፡ **ለምሳሌ፡-** በሽታ መከላከልና የሕክምና አገልግሎት በጤና መምሪያ ይመራል፤ ንግድና ገበያ ልማት የንግድና ኢንዱስትሪ መምሪያ ይሠራል፡፡ ሀብት አሰባሳቢ በጐ ፈቃድ አገልግሎት የሚባል ንዑስ ኮሚቴ የሚባል አለ፤ ትራንስፖርትና መንገድ አለ፡፡ እንደዚሁም ሰላምና ፀጥታ ፐብሊክ ሰርቪስ አለ፤ ሳኒቴሽንና ሃይጂን፣ ግብርናና ልማት፣ ሠራተኛና ማህበሪዊ ጉዳይ የሚባሉ ንዑሳን ኮሚቴዎች አሉ፡፡ እነዚህ ንዑሳን ኮሚቴዎች በየዕለቱ የሚያከናውኗቸውን ተግባራቶች እየደረሰን ሠራተኞች መድበን የየዕለት ክንውን በዞኑ የፌስ ቡክ ገጻችን እንለጥፋለን፡፡ የሚዲያና ኮሙኒኬሽን አባል ሆነው የሚንቀሳቀሰው የደቡብ ሬድዮና ቴሌቭዥን ድርጅት የአርባ ምንጭ ቅርንጫፍ Fm 94.9 የዚህ ባለድርሻ አካል እንደመሆኑ ተቀናጅተን የህዝብ ግንኙነት ሥራ እንሠራለን፡፡ የዐቢይና ንዑሳን ኮሚቴዎች በየዕለቱ የሚያከናውኑትን ተግባራት እየተከታተልን በዞኑ አብዛኛው ኀ/ሰብ በሚጠቀሙባቸው ቋንቋዎች፡- በጋሞኛ፣ ዘኢሴኛ፣ ግድቾኛና አማርኛ መረጃዎች ወደ ህዝብ እንዲደርሱ እያደርግን ነው፡፡

እንግዲህ የህዝብ ግንኙነት ሥራ ስንሠራ ዋናው መቋሚያችን ኀ/ሰቡ ተገቢ መረጃ አግኝቶ ራሱን ከኮቪድ-19 በሽታ እንዲከላከል ማድረግ ነው፡፡ በዚህ ረገድ በርካታ ሥራዎችን ሠርተናል፡፡ እንግዲህ ለማስተማሪያ ከተሰሩት ሥራዎች ባነር በማዘጋጀት መልእክቶችን ሕዝብ በብዛት በሚገኙባቸው አካባቢዎች በመስቀል፣ ብሮሸር አዘጋጅተን ሁለት ዙር አሰራጭተናል፡፡ ቀጣይ ብሮሸሮች አዘጋጅተን ለማሠራጨት ስንዘጋጅ ከወደ ጤና ሚ/ር የመጣው መልዕክት በብሮሽር ወረቀትም ላይ ቫይረሱ ሊዛመት እንደሚችል ስለተገለፀ ይህንን ትተን በግድግዳ ላይ በመለጠፍ ስቲከሮች መልእክት ማሰራጨት ቀጥለናል፡፡ ሌላው ደግሞ በአዋጅ መልክ ሕዝብ በብዛት በሚገኙባቸው ቦታዎች ድምጽ የሚያጐላ መሣሪያዎች /እንደ ሞንታርቦ/ በመጠቀም ለማወጅ ያደረግነው ሙከራ ስዎች ግር ብሎ በብዙ ቁጥር ያለጥንቃቄ መሰብሰብ ተቃቅፎ መቆም በመጀመራቸው ቀይረናል፡፡ በመሆኑም አማራጭ መፍትሔ መፈለግ ስላስፈለገ ጥዋትና ማታ መልእክት በመቅረጽ ጥዋትና ማታ በድምጽ በተሽከርካሪ በመዘዋወር ማሰማት ቀጥለን ይህም ሰዎች ገና ከመኝታ በሚነሱበትና ከሥራ ወደ ቤት በሚመለሱበት ጊዜያት ድምጽ እየተወረወረ ስለሚጓዝ እንዲያዳምጡ ለማድረግ ነው፡፡ የተለያዩ የሬድዮና ቴሌቭዥን ዜናዎችን እየሠራን እንዲሰራጭ አድርገናል፡፡ የዞኑ ፌስቡክ ገጽም በርካታ ተከታይ ስላለ መልእክቱን ለማስተላለፍ አግዞናል፡፡ያም የህዝብ ግንኙነት ሥራ ከማሳለጥ አኳያ መልካም ስራ ፈጥሮልናል፡፡ እንግዲህ በዞን ማዕከል የምንሰራቸውን ሥራዎችን እስከ ታችኛው መዋቅር በ14 ወረዳዎች አራት የከተማ አስተዳደሮች ተደራሽ እንዲሆኑ የተለያዩ አካሄዶችን **ለምሳሌ፡-** እንደ ቴሌግራም በመጠቀም መልእክቶች እንዲደርሱ አድርገናል፡፡ ከዞኑ አስተዳደር ጋር ለዐብይ ኮሚቴነትና ከሌሎች ንዑሳን ኮሚቴዎች ጋር ወደየወረዳዎች ተከፋፍለን በመውረድ በተጨባጭ መሬት ያለውን ሁኔታ በማየት፣ በመገምገም ድጋፍ የማድረግ ሥራ ይሠራል፡፡

ሌላው የበሽታ መከላከልና ጤና አገልግሎት እንቅስቃሴ ሲታይ ለይቶ ማቆያዎች እንዴት ተደራጁ የሚለውን ወርዶ ማየት ነበረብን፡ ትምህርት ቤቶች ለይቶ ማቆያ አድርገው ተዘናግቶ የቆዩትን ውስጡ ገብተን ምን ያህል ፍራሽ አለ**?** እንዴት አድርገው አደራጁት የሚለውን ሁሉ በደንብ አይተናል፡፡ አብዛኛው ትምህርት ቤቶች ዝም ብለው ይኼ ነው ብለው የነበሩትን ገብተን አይተን እንዲያሻሽሉ ያደረግንበት ይገኛል፡፡ በጥቅሉ የማስተካከል ሂደት ውስጥ ክፍተቶችን ለይቶ በመጠቆም ረገድ ጥሩ ርቀት የሄድንበት ሁኔታ አለ፡፡ ወደ ወረዳ ወርደን በርካታ ሥራዎችን አይተናል፡፡ በተለይ ከገበያ ማሳካት ጋር ያሉ ሁኔታዎች ከእጅ መታጠቢያ አዘገጃጀት ጋር ርቀትን ጠብቆ ከመገበያየት፣ ሱቆች ሲገበያዩ ርቀታቸውን የመጠበቅ ሁኔታ ገመድ ወጥሮ ከማስተናገድ አኳያ፣ ገበያ ላይ ኢኮኖሚያዊ ምክንያት በሌለበት መንገድ የዋጋ ንረትን ለመቆጣጠር ያለውን ሁኔታ፣ ከትራንስፖርት ጋር ተያይዞ ክልከላውን ተላልፎ እጥፍ አስከፍሎም ከወንበር በላይ ጭኖ የሄዱትን የቀጡበት አግባብ አይተናል፤ በሞተር ብስክለት ላይ አንድ ሰው መቀመጥ አለበት ሲባል ሶስት አራት ሆነው የሚቀመጡትን ለማስተካከል የሄድንበትን ርቀት፣ እኛም ታዝበን ግብረ-መልስ የሰጠንበት እንዲሁም ከሀብት ማሰባሰብ ጋር በምን ሁኔታ ነው ሀብት እየተሰበሰበ ያለው እንዲሁም ኀ/ሰቡን በምን ዓይነት ሁኔታ ነው እያሳተፋ ያሉትና ሀብት ወደ አንድ ማዕቀፍ /መጋዘን/ ከማስገባት አኳያ ያለውን ሁኔታ ያየንበት ሁኔታ ነው ያለው፡፡ በጥቅሉ ከንዑሳን ኮሚቴዎች ጋር የሚዲያና ኮሙኒኬሽን ንዑስ ኮሚቴ እስከታች ወርዶ በቅንጅት የሠራበት ሁኔታ እንዲያው በቀላሉ የሚታይ አይደለም፡፡ ከክልሉ የመንግሥት ኮሙኒኬሽን ቢሮ ጋር በየሳምንቱ ረቡዕ ዕለት በቪድዮ ኮንፍራንስ የተሰሩ ሥራዎችን ወደ ላይ መረጃ እንሰጣለን፡፡ በምን ዓይነት ሁኔታ ላይ እንዳለና በቀጣይ በምን አይነት አግባብ መቀጠል እንዳለበት ደግሞ በኘላን ‘A’ ሰውን በመለማመጥ እዚህ ደርሰናል፡፡ ኘላን ‘B’ ደግሞ ምን መሆን አለበት በሚለው እኛ እንደኛ እየሰራን ነው፡፡ አስቀድሞ እጃችሁን እንደዚህ ታጠቡ፣ ስትቆሙ አፋችሁን ሸፍኑ፣ ከሰው ራቅ በሉ እያልን ጥሬውን ነገር እየሰጠናቸው ነበር፤ አሁን ከመልዕክቱ ጋር ሰው ስለተለማመደ ጅምር አካባቢ የነበረው መደንገጥና መጠንቀቅ አሁን ምን ላይ ደረሰ? አስተምረን የመጣው ለውጥ አለመጨባበጥ በአሁን ላይ በሁሉም ላይ የጋራ ባህሪይ ሆኗል፡፡ ርቀትን ጠብቆ ከመቆም አንፃር ክፍተት አለ፡፡ በከተሞች አካባቢ ይታያልና እስካሁን በማስተማር በሄድንበት በቂ ለውጥ ባለመምጣቱ ተፋፍጐና ተቀርርቦ የሚቆሙትን በሀይልም ቢሆን መስመር ማስያዝ ቢቻል መንግስት ህዝቡን የማዳን ኃላፊነት አለበት ስለዚህ የበሽታው አስደንጋጭነትና ገዳይነት በልኩ ያስተላልፋል፡፡ ማህበረሰቡ ስላለ ምን መደረግ አለበት የሚለውን ከማእከላዊ ጋር ከክልላችን መንግስት ኮሙኒኬሽን ቢሮ ኃላፊ ጋርም በቪዲዮ ኮንፍራነስ ሃሳብ አስተያየት የተለዋወጥንበት ሁኔታ አለ፡፡ እንግዲህ ይህንን ስናደርግ እነማን እነ ማን ነበሩ በአስተምሮት ሥራ ላይ የተሳተፋት ስንል የዞን ዋና አስተዳዳሪ፣ የከተማ ከንቲባዎች በየገበያው ላይ እኔ እገሌ እገሌ እባላለሁ በሽታው ከባድ ነው ባለንበት ሆነን በቀጥታም በተዘዋዋሪ በቴሌቭዥን፣ በሬድዮ ሲተላለፍ እንሰማለን፤ ነገር ግን ሕዝባችን ይህንን አውቆ ምላሽ እየሰጠ እይደለም፣ ይህ ግራ ቢያጋባን ነው ወርደን ለማስተማር የተገደድነው ቅድም እንዳልኩት ሰው ደግሞ ተሰብስቦ ይሄ ነው ወይ አስተዳዳሪያችን ብለው ለማየት ወደዚያ ተሰብስበው ይመጣሉ፡፡ የሀይማኖት አባት መሪዎች፣ የወጣት አደረጃጀቶች፣ የታክሲ ማህበራት የቀይ መስቀል፣ ያላወቁት አደረጃጀት አልነበረም፡፡ ነገር ግን ሰው ድምጽ ወዳለበት በመሰብሰቡ ይሄንን አማራጭ ማካሄድ አልተቻለም፡፡ የአርባ ምንጭ ዩኒቨርስቲ በማህበረሰብ አቀፍ አገልግሎት በርካታ ሥራዎችን እየሠራ ይገኛል፡፡ ሌሎች መንግስታዊና መንግስታዊ ያልሆኑ ድርጅቶችም እየተስፋፋ ይገኛሉ፡፡ ግን የአርባምንጭ ዩኒቨርስቲ የሚያክል የለም፡፡ የአርባምንጭ ዩኒቨርስቲ በዞኑ ሁሉም ወረዳዎችና የከተማ አስተዳደሮች በመድረስ የራሱን ኃይል አስማርቶ በሞንታርቦ ይዞ፣ ሳኒታይዘር፣ ሳሙና፣ ማስክ እንዴት መጠቀም እንዳለባቸው ሁሉ ለየመዋቅሮቹ እየሰጠ የሙቀት መለኪያ ለዞኑ ገዝቶ የሰጠበት ሁኔታ፣ የአርባ ምንጭ ጤና ሳይንስ ኮሌጅም ባቅሙ ወረዳዎች ተከፋፍሎ ሕዝቡን በሞንታርቦ መልእክት ማስተላለፍ ሞክሯል፡፡ ይህ ሁሉ ግን ሲደረግ ከኛ ጋር በመናበብ ነበር መልዕክት በጋራ እንቀርፃለን ይህንን በመውሰድ በሞንታርቦ ይተላለፋል፡፡ የህዝብ አስተያየት የምንሰበስብባቸው የታችኛው መዋቅር ላይ ህዝቡ ምን ይላል የሚለውን እየደወልን ከዚያ ያለውን ነገር ወደዚህ እናመጣለን ለክልል ኢ.ሜል እናደርጋለን፡፡ እያንዳንዱ ነገር ሊሠራ የክልሉ የኮቪድ መካከል አደረጃጀት ማኑዋል ተከትለን ስንሠራ አንድ ለአስር መምጣት አለበት ተብሎ ይህም ህዝብን በብዙ ቁጥር አሰባስቦ ማስተማር ስለማይቻል በሰፈር ያለውን አስር ሰው የሚጠረንፍ አደረጃጀት ሥርዓት ውስጥ ወደታች ከመግባት አንፃር አንዳንድ ጊዜ በአንዳንድ መዋቅሮች ተንሳፍፎ ያገኘንበት ጊዜ ነበር ይህንን ያገኘነው ለድጋፋዊ ክትትል ወደ ታች በወረድንበት ጊዜ በወረቀት ላይ ሪፖርት በጥሩ ሁኔታ የተዘጋጀ ነበር ግን እስቲ ለናሙና ከተሰሩት ውስጥ አንድ ቀበሌ ወስደንና የተደራጀ 1 ለ1ዐ እንይ ስንል እሱን ዛሬ ገና ከናንተ ሰማሁ አለ በሌላ ጐኑ ግን ሪፖርት ይህንን የሚገልጽ ተሠርቷል፡፡ በዚሁም ያገኘነውን ግኝት ይዘን መጥተን እዚህ በካቢኔ ደረጃ የጋራ አድርገናል፡፡ በጋራ ከገመገምን በኋላ የዞኑ አስተዳዳሪውን ጨምሮ ያንን አካባቢ ከሚደግፈው ጋር በጋራ ሆነን ተመላልሰን ስናናግረው ሊቀመንበሩ እኔ አዲስ ነኝ እዚህ ጉዳይ ላይ ሌላ ይደግፍ የነበረው አካል ደግሞ 1 ለ1ዐ ተደራጅቶ ሥራ ጀምሯል ብሎ ሪፖርት አቅርቦ ነበር፡፡ እኛ ህዝቡ ተገቢ መረጃ አግኝቶ ራሱን እንዲንከባከብ እንጂ ላሸንፍህ አሸንፈኝ አይደለም፤ ይህ የውሸት ሪፖርት በኮቪድ ጊዜ ህይወትን ከመብላት ባሻገር ላይ ምንም ጊዜ አይኖርም በሚለው ላይ ተማማርን፡፡ በየወረዳው ላይ እንዳንድ ቦታ ላይ ምን ያህል ሀብት ተሰበሰበ ስንል ሀብት አላሰባሰብንም በያንዳንዱ ቀበሌ በቤተሰብ ተበትኖ ነው ያለው ይላሉ፡፡ እስቲ ያለው ታሳቢ ይሁን ስንል እኛን ሸንግሎ ለመሸኘት የሚያደርጉት ጥረት እንደሆነ ስለዚህ ሃብት በወረዳ ደረጃ በለይቶ ማቆያ የሚሆን እንደሆነ በወረዳ አንድ ማዕከል ተሰብስቦ መቀመጥ እንደሚገባ አፋጣኝ ምላሽ የሚሰጥበት ሁኔታ እንዲመቻች ግብረ-መልስ ሰጥተናቸው በዚህ ልክ በዕለቱ ወረዳዎችን በተለይ ፊት ለፊት የወጡ መሀል ላይ ያሉትን ወደ ኋላ የቀሩት ተብሎ መስፈርት ወጥቶ በዚያ መስፈርት ደረጃ ወደ ማውጣት ደርሰን ነበር፡፡ ነገር ግን አሁን ያለው ሁኔታ የማልዋሻችሁ እንደ ደቡብ ክልል ከኮቪድ የበለጠ ትኩር እያገኘ ያለው የመዋቅር ጉዳይ ሌላ ኮቪድ ሆኗል፡፡

**በናንተ ተቋም የኮቪድን በሽታ መከላከልን ቁጥጥር ሥራ የሚያስተባብር ኃላፊነት የተሰጠው አካል አለ ወይ?**

ኮቪድን በተመለከተ እዚህ ያሉት ፈፃሚዎች ለሁሉም ንዑሳን ኮማቴዎች ተከፋፍለው እያገዙ ይገኛሉ፡፡ አንድ ንዑስ ኮሚቴ **ለምሳሌ፡-** የንግድና ገበያ ልማት ወደ መስክ ሊሄድ ሲፈልግ ማንን ደውሎ ማነጋገር እንዳለበት ሁሉም ተደልድለዋል፤ በዚህ የተመደበው ሄዶ መስክ ሠርቶ ሲመጣ የኤዲቲንግ ሥራ አለ ትክክለኛው መረጃ ተጠናቅቆ በመምሪያው ገጽ ላይ ካስት ይደረጋል፡፡ ከክልል ጋር ለመገናኘት መረጃዎች ተጠናቅረው በመምሪያው ኃላፊ /በኔ/ ፊርማ ይላካል፡፡ እኔን ሳያወያይ ማንም መግለጫ አይሰጥም፡፡ ኮቪድን በተመለከተ የሚዲያና ኮሙኒኬሽን ንዑስ ኮማቴ ሰብሳቢ እኔ ስለሆንኩ አሁን ባለው ስለዚህ ሁኔታ ላይ ከዚህ ወጣ እያሉ ያሉት ሰዎች ጭውውት አዘጋጅተናል፡፡ ይህንን ጭውውት አየር ላይ ለማዋል ነዳጅና አበል ሌሎችንም የሥራ ማስኬጃዎችን ለአበል አድርገን ለዛሬ ማታ ሥራ ለመጀመር የጀንሬተርንም ሥራ ለመጀመር ትውስት እየጠየቅን ነው፡፡ ለዚህ እንቅስቃሴ ተሽከርካሪ ከአርባ ምንጭ ኤፍ.ኤም (FM) እንዋሳለን፡፡ ለሚንቀሳቀሰው ነዳጅና አበል ከኮቪድ ቋት ወጩ ይደረጋል፡፡ ስለዚህ ከዚህ በፊት ኮቪድ መከላከል ላይ መልእክት ቀርፀን ያስተላለፍንበት ሁኔታ ላይ ነው ያለው፡፡ እኔ እናንተም በሄዳችሁበት ሁኔታ ላይ በምትሄዱበት አካባቢ ላይ እንደተሞክሮ እንድታነሱና ሌላውም በዚህ ጉዳይ ላይ የተሻለ መረጃ ኖሮት እኛ የሰራነውን ሥራ ቢሠራ የህዝቡን ጆሮ ማግኘት ይቻላል እንላለን፡፡ ከምንም በላይ ከኮሮና መከላከል ጋር ተያይዞ ፊት እናንተም እንደምታውቁት የማስተማሩ ዘዴ በደረቁ ነበር እኛ ቆመን ስናስተምር ሰው ቆሞ የማላገጥ ሁኔታ ነበር ከመልእክቱ ጋር ተለማምዶ ማለት ነው፡፡ ስለዚህ ምን ማድረግ ይሻላል ሲባል እስቲ እስታይሉን እንቀይር ብለን እኛ ቀብር ሰው እንዲወጣ ጥሩንባ እንጠቀም ነበር፣ እስቲ ይህንን ጡሩንባ እየነፋን አዋጅ አዋጅ አዋጅ ጡ … ጡ ……ጡ / የጥሩንባ ድምጽ/ ሲነፋ የሚያወጣው ጥ.ጥ.ጥ. ጡጡ .. አዋጅ አዋጅ አዋጅ የዓለም ስጋት የሆነው ዘር፣ ቀለም፣ ልጅ፣ አዋቂ፣ ሀብታም፣ ድሀ የማይመርጥ ኮሮና የሚባል ወረርሽኝ ወደ ሀገራችን ገብቷል፤ ከመግባትም አልፎ በየቀኑ በቫይረሱ የሚያዙ ሰዎች ቁጥር እየጨመረ መጥቷል ጡ… ጡ…. ጡ….ጡ ….ጡ …… ጡ.ጡ.ጡ አዋጅ አዋጅ አዋጅ ያልሰማ ስማ ላልሰማ አሰማ አዋጅ አዋጅ አዋጅ ጡ…ጡ…ጡ ልጅ ወላጅንም አስታምሞ የማይቀብርበት፤ እናት ልጇን ቀርባ የማታስታምምበት ወገን ቀርቦ ሟችን የማይገንዘበት አልቅሶ የማይቀብርበት ወረርሽ ወደ ሀገራችን ገብቷል ባሁኑ ወቅት በመስፋፋት ላይ ይገኛል፡፡ ጡ..ጡ… ጡ….. አዋጅ አዋጅ አዋጅ ጡ…. ጡ….. ጡ …. ከዚህ መቅሰፍት ለመዳን ርቀታችሁን ጠብቁ፤ አትጨባበጡ፤ እጃችሁን ሳትታጠቡ አይናችሁን፣ አፋችሁንና አፍንጫችሁን አትንኩ፤ ሰምቶ እንዳልሰማ መሆን ለእልቂት ይዳርጋልና ነገን ለመኖር ዛሬን መጠንቀቅ የግድ ይላል፡፡

ይኼ በአማርኛ የተዘጋጀ ነው፤ በጋሞኛም፤ በዘኢሰኛና በጊጅቶኛ ተዘጋጅቶ በሁሉም ቋንቋ በምንናገርባቸው አካባቢዎች መልዕክቱ ተቀርጾ በሞንታርቦ ቅርብ በሆነ መልኩ እንዲሰሙ በተግባር የዋለ ነው፡፡ ጭውውትም የየዕለት የኑሮ ሁኔታ ባገናዘበ መልኩ ታሳቢ ባደረገ መልኩ እየተከናወነ ይገኛል፡፡

**አብዛኛው ተቋማችሁ በራሱ ደረጃ እየሰራ ላለው መነሻ የሆነ የአሠራር ማዕቀፍ ስለመኖሩ፤ መነሻ ማዕቀፍ ምንድነው?**

ዋናው የክልሉን የኮቪድ መከላከያ ማኑዋል ተከትሎ ዐብይ ኮሚቴ ተቋቋሞ በዐብይ ኮሚቴ ከታቀፋት አንዱ የኛ መንግስት ኮሙኒኬሽን ነው፡፡ ተቋማችን በሰው ተወክሏል ይህ ሰው ደግሞ የዚህ ማኀ/ሰብ አባል ነው፡፡ ዓለም በምን ሁኔታ ላይ እንደሆነ የተነገራቸውን በአግባቡ ያልተገበሩት ምን ሁኔታ እንዳጋጠማቸው በቴሌቭዥን አይተናል፡፡ ሰው በዕድሩ በአባላት ነው የቀብር ጉድጓድ ሲቆፈር የነበረው ነገር ግን በኮቪድ ሁኔታ እስካቫተር ሲቆፍር አይተናል፡፡ ሰው ባልተለመደ ሁኔታ በአንድ ቀን በጅምላ የጦር መሳሪያ ተጨፍጭፎ እንደተገደለ ሲሞትና ሲቀበር ነው ያየነው፤ መጀመሪያ አካባቢ ይህ ሁኔታ ወደ እኛ የማይደርስ መስሎን ነበር ውጭ ነው ቻይና ነው ሲባል ባንድ ጊዜ ዓለምን ወረረ፤ አሁን ባለው አስደንጋጭ ሁኔታ እኛን ድረስ እንግዲህ ምን ዓይነት ሲስተም ብንቀርጽ ይሻላል እኛ ክልልን እየጠበቅን አይደለም ከየትም የምንንነግራቸው የለም ነገር ግን ነባራዊ ሁኔታው አዳዲስ ነገሮችን እንድንፈጥር ከተለመደው አካሄድ ከተለመደው የአዘጋገብ ስታይላችን በጣም Toachy /ታቺይ/ በሆነ መልኩ እንድናዘጋጅ እያስገደደን ነው፡፡

**ይህንን የምትሰሩበት የራሳችሁ የሆነ ዕቅድ ስለመኖሩ?**

መነሻ ዕቅድ አለን ይህንን በደረስንበት እያሳደግን እናገኛለን፡፡ መጀመሪያ ላይ የነበረው ዕቅድ በምንፈልገው ሁኔታ ሊያንቀሳቅሰን ባለመቻሉና ሁኔታውም ተለዋዋጭ በመሆኑ ዕቅዱን መከለስ አስፈልጐን ነበር፡፡ የተገደበች ሆነች የመጀመሪያ ዕቅዱ ሲዘጋጅ በሽታው እኛጋ አልደረሰም ነበር፤ አሁን ግን በዞናችን ዳራማሎ ወረዳ ላይ ተገኝቷል፡፡ የዚያ መነካካት ውጤት የት ሊደርስ እንደሚችል ግልጽ ስለሆነ ከዚህ በኋላ የምንፈጥረው የቅስቃሳ ሜቴዶሎጂ ምን ሊሆን እንደሚችል ነገ ተነገወዲያ ወደ አዲስ ነገሮች ልንገባ እንደምንችል ከዚህ በፊት የምናደርገው ደረቅ ቅስቀሳ እንዳሰለቸ በመገንዘብ አሁን ካለው ነባራዊ ሁኔታ ጋር እሺ አሁን ምን እናድርግ የሞተውን መገነዝ የማይቻልበት አልቅሶ መቅበር የተሳነበት ማልቀስና መቅበር ባህላችንና ሃይማኖታዊ ሥርዓታችን ነበር ነገር ግን በዚህ ጉዳይ ላይ ማልቀስም ቅንጦት የሆነበት ሁኔታ ነው ያለው፡፡ ስለዚህ አንድ ሰው ሞተ ተብሎ ሌሎች ተሰብስበው በበሽታው እንዳይጠቁና እንዳይሞቱ ወደ ቤት ግቡ የተባለበትና ከባህላችንም የወጣንበት ሁኔታ ነው ያጋጠመን፡፡ ሰዎች ራሳችሁን ጠብቁ የሚል መልዕክት በጥሩንባ ስናሰማ ደግሞ ማን ሞተ የሚል ያስተጋባ ነበር፡፡

ስለዚህ እኛ ማታ ጡሩንባ ስንነፋ በቃ መንግሥት የመጨረሻ ጫፍ ስለደረሰ ነው ይኼንን ያመጣው እንስማ እንጠንቀቅ የሚል ነገር በህዝቡ ውስጥ እየሰረፀ ነው፡፡ ስለዚህ ዕቅድ ነበረን ግን በየጊዜው እያደገና እየዳበረ መጥቷል፡፡

እኛጋ ያለው ፈፃሚ ሁሉ ይህ ሰው በላና አውሬ ነገር የማያስቀረውን ኮሮና ስለመጣ ከዚህ የሚበልጥ ተልዕኮ ስለሌለን በክልላችን እያረስን እርሻ ሳይቋረጥ ኮቪድን እንከላከላለን፡፡ አርሶ አደሩ ከመደበኛ የእርሻ ሥራ ሳይፈናቀል ኮሮናን መከላከል አለበት፡፡ የልማት ሥራ እየተሰራ ጐን ለጐን ደግሞ የኮሮና በሽታ የመከላከል ሥራ Exhostively እየተከናወነ ይገኛል፡፡

**ሥራውን በጋራና በቅንጅት የምትሰሩ ከሆነ የድጋፍና ክትትል ግምገማ አግባብ ምን ይመስላል?**

**ቅንጅታዊ ሥራ ምን ይመስላል?**

እንግዲህ እርስዎም እንዳሉት ይህቺን የኛ ቢሮ ያዩ ከኮቪድ በሽታ አስቀድሞ እንደመምሪያ ስንዋቀር የተሰጠችን ናት፡፡ ነገ ሰፋ ያለ የሥራ ቦታ እናገኛለን ብለን እናምናለን፡፡ እስከዚያው ቦታው Setup እንሰራለን ብለን ነው የገባነው ስንገባ 14 ሰዎች ነን እዚህ ውስጥ ያለነው ከኮቪድ ጋር ተያይዞ ደግሞ ሪቀትን ጠብቀን መሥራት አለብን በመሆኑም በኛ Setup ይህንን 14 ሰዎች በአንድ ጊዜ ማስገባት አልቻልንም፡፡ የሚያጠቡና በዕድሜ የገፋትን ሁሉ እንደኛ ተጨባጭ ፖሊሲ፣ ጤናና ሚዲያ በኮቪድ ጉዳይ ላይ እረፍት የወጣ ሁሉ ወደ ሥራ የተመለሰበት ሁኔታ አለ፡፡ የህዝብ ግንኙነት ሥራ በተጧጧፈ አኳኋን መሠራት ስላለበት የሰው ኃይል ይፈልጋል፡፡ በዚህ ሁኔታ ላይም ሆነን ቢያንስ ሠራተኞችን ወደ ቤት ግቡ ከማለት በፈረቃ እንዲሠሩ አድርገናል፡፡ እንግዲህ እኛ እንሰራለን ስንል ጤና መምሪያ ይደግፈናል መከላከል ላይ በጣም ከተሰራ ኋላ ችግሩ ከተፈጠረ በኋላ የምንከፍለውን ዋጋ ይቀንስልናል በሚል አስፈላጊ ግብዓት በመመደብ ከጐናችን ነው፡፡ እኛም አልቦዘንም ግን እጥረቶች አሉብን ይህም የግብዓት እጥረት ነው አንዳንድ ጊዜ ወደታች ወርደን ለመሥራት ከኋላ ክፍት የሆኑ ተሽከርካሪዎች ያጥራሉ፡፡ በዚህን ጊዜ ይህንን ገታ አድርገን የፀጥታ ሥራ ጊዜ ስለማይሰጥ ቅድሚያ ሰጥተን የምናስታግስበት ሁኔታ አለ፡፡ እንደተቋም የራሳችን ተሽከርካሪ ያለመኖር ክፍተት ቢሆንም ያለውን በማብቃቃት ተውሰን እንሠራለን፡፡ ሌሎች ስለሚተባበሩን ተቀናጅቶ ከመሥራት ጋር ተያይዞ በተለይ Event ስልጠና የጐዳና ላይ ቅስቀሳ የችግኝ ተከላ በዚያው ልክ ርቀትን ጠብቆና ማስክ አድርጐ ርቀትን ጠብቆ መረጃውን እንዲሰጥ በጐዳናው ላይ ፖስት ስናደርግ እጅጉን እንጠነቀቃለን፡፡ ገበያ የሚያህል ሕዝብ ጥቅጥቅ ብሎ ፖስት የምናደርግ ከሆነ ሌሎችን ማጋለጥ ነው የሚሆነው ፎቶ እንኳን ስንነሣ ርቀታቸውን ጠብቀው እንዲቆሙ አድርገን ያንን ፓርት አንስተን ፖስት የምናደርገው ወይንም የማይመች ከሆነ አንዱን ግለሰብ ወስደን በርሱ መግለጫ እንዲሰጥ የምናደርግበት ሁኔታ አለ፡፡ ከቅንጅት ጋር ተያይዞ እስከአሁን ባለው ጊዜ ምንም ችግር አላጋጠመንም የሚፈልጉን ይደውልናል በዚያች በሚደውሉበት ጊዜ ያለንን Resource ይዘን የህዝብ ግንኙነት ሥራችንን ለማሳለጥ የራሳችንን ጥረት እያደረግን ነን፡፡

**የተጀመሩት ሥራዎች /የዕቅድ ክንውን ወደታለመለት ግብ ስለማድረስ ያለማድረስ ምን ማረጋገጫ አለ? እንዴትስ ሊረጋገጥ ይችላል?**

እኛጋ ፍጥነት አለ ዛሬ ሠርተን ዛሬ ውጤት ለማየት ጉጉት አለ፡፡ ጊዜ ይወስዳል ታገሁ ይሉናል፡፡ በሌላ ጐኑ ሰው እየተነገረው ፈቀቅ በሉ ራቅ ራቅ ብላችሁ ቁሙ ተብሎ እየተነገረ ሰው የማፈዝ ከሆነ ወደ ተስፋ መቁረጥ ሊወስድ ይችላል፡፡ እንዲህ ዓይነት ነገር በአንድ ቀን በአንድ ጀምበር ለውጥ አይመጣም ቀስ ተብሎ ነው፡፡ ሰው አልሰማም ተግባራዊ አላደረገም ብለን ማቆም የለብንም በድግግሞሽ ለውጥ ይመጣል፡፡ አትጨባበጡን ተሻግረናል፡፡

የቀረው ርቀታችሁን ጠብቁ፣ ሰው ከተሰበሰበበት ስትደርሱ ማስክ ማድረጋችሁን ቼክ አድርጉ /አረጋግጡ/ የሚሉት ክፋይ እያየን ነው ከተሞች አካባቢ የተወሰነ የባህሪይ ለውጥ እያየን ነው፡፡ ወደ ገጠር ገበያ አካባቢ ስንሄድ “ጦስ ኤሬስ” በሚል ተዘናግቶ ክፍተታችንን አይተናል፡፡ እዚህ አካባቢስ ላይ ምንድነው የሚጠበቀው ሲባል ሰው ከጐረቤት ሰው ታሞ ማየትን ይፈልጋል፡፡ እኛጋ ለመረጃ እንዲሆንላችሁ ተጠርጣሪ አንድ ተገኘ ያን ተጠርጣሪ ገባ ሲባል አዲስ ነበር በዞኑ ጤና መምሪያ ኃላፊ መግለጫ ተሰጠ ከዚያም አርባ ምንጭ ገብቷል ተጠርጣሪ ብሎ ዳቦ ስም የሰጡት አውቀው ነው ከዚያም ከአርባምንጭ የሚመጡ መኪኖች እኛጋ እንዳይገቡ በማለት ሌላ ሳንክሽን ከመጣ በኋላ ውጤቱ በ24 ሰዓት ሲገለጽ ውጤቱ ኔጌቲቭ ሲሆን ራሳችሁን ጠብቁ ሲባል ሁሉም ተንጋጋ በፍርሃት በሩን ቆልፎ የሮጠበት ሁኔታ ሁሉ ነበር፡፡ ለጉዳይ የመጣ ሁሉ እዚህጋ ማደር የለብኝም ብሎ ወደ ቤቱ የተመለሰበት ሁኔታ ነበር፡፡ ምናልባት እዚህጋ ሞት ተከሰተ ቢባል ሰው የማይኖረው መጣ ወደሚለው ሁኔታ ሊገባ ይችላል፡፡ ግንዛቤ አለ ግን በድርጊት ላይ መዘናጋት አለ፡፡ አውቆ እንዳላወቀ የመሆን አዝማሚያ አለ፡፡ በጥቅሉ እኛ አሁን የያዝነው ስታንድ ከተሞች አካባቢ ወጣቶች ወጥተው ለእናቶቻቸው ለአባቶቻቸው ምንም ላላወቁት ከፍቶ ይዛችሁ እንዳትገቡ ብለን እየነገርን ነው፡፡ እንዲያም ሆኖ እያወቀ መረጃ እያላቸው መጠንቀቅ ሲገባቸው ወደ ተግባር ለመለወጥ ማገድ የመጨረስ ሁኔታ አለ፡፡ ይኸም ሆኖ ግን ለነርሱ በቀረበ ቋንቋ እነርሱን መቅረብ ያስፈልጋል፡፡ ስለዚህ እኛ ካለፍነው አንፃር መሬት ላይ ያለው ስናይ የማይናቅ ለውጥ አለ፡፡ ይህንን ተስፋ ሳንቆርጥ መቀጠል ያስፈልጋል የሚል አቋም አለን፡፡

**ከበሽታው ጋር ከአራት ወር በላይ ሆኖናል፡፡ በዚህም የመከላከልና ቁጥጥር ሥራ እና ክንውን በመጣንበት ሂደት ምን ትምህርት ወስደናል፣ ምንስ መልካም አጋጣሚዎች ተፈጥሯል?**

እንግዲህ መንግስት እንደመንግስት በመረዳዳት ላይ በደንብ እንድንሄድ በዚህ ቀውስ ወቅት ላይ ገበያው እሳት ሆኗል፤ በዚህ ላይ ኮቪድ መጥቶ ወደቤት ግቡ እየተባለ ነው ገቢያ ተቀዛቅዟል፤ ሰው ከዚህ ተነስቶ አዲስ አበባ ሄደው ቢዝነስ ለመሥራት መተማመን የለውም፡፡ ሸቀጦች ከአዲስ አበባ ነው የሚመጣው ስለዚህ ወቅት የምግብ ቁሳቁሶች፣ ሸቀጦችና በግንባታ ግብዐቶች ላይ በየቀኑ የዋጋ ጭማሪ ይከሰታል፡፡ በዚህ ላይ መንግስት የማስተካከያ እርምጃ እንዲወስድ ይጠበቃል፡፡ በሽታው ረጅም ጊዜ ሊቆይ ከቻለ ህይወት እንዴት ሊቀጥል ይችላል? በሚለው ላይ በእጅጉ ሰው መልስ ይፈልጋል፡፡ መልስ የሚሰጠው አካል ደግሞ ከዚህ ጋር ተያይዞ ጫኝና አውራጅ ራሱ ሥራ ያጣበት ሁኔታ አለ፤ መጀመሪያ አካባቢ መንገዶች ተዘጉ፣ ቀጥሎ ተከፈቱ መንገድ ሲከፈት ደግሞ በእያንዳንዱ መናኸሪያ የሙቀት መለኪያ ይደረግ ተባለ ሙቀት መለኪያው ላይ ድንገት ሀዋሳና ሶዶ መጥታችሁ ካያችሁ ሽጉጥ የምትመስል ልኬት ዘይት በኦርቶዶክስ ቤተክርስቲያን በጥምቀት ጊዜ ጠበል ለመርጨት ሰው “ አባ እኔን እኔን እያሉ እንደያጠምቁ ልክ እንደዚያው ይህችን ሙቀት መለኪያ እኔን እኔን እያለ እጅግ ተፋፍጐ ይሰለፋሉ መኪናው ጥሎን ይሄዳል በሚል ስጋት አስፈላጊ ማስተካከያ ካልተወሰደ ከጥቅሙ ጉዳቱ ሊያመዝን ይችላል፡፡ ይህ ከጤና አንፃር በሽታውን ሊያስተላልፍ እንደሚችል ሊገነዘቡ ይገባል እንጂ ለመርሃ-ግብር ማሟያ መሆን የለበትም፡፡ ምርመራውም ተጋላጭ ነው፡፡ ለይቶ ማቆያ አካባቢ ያለው ዝግጅት ደካማ ነው፡፡ ዳርማሎ ላይ ሰው እግር ኳስ፣ መረብ ኳስ ያለምንም ጥንቃቄ ይጫወታሉ፡፡ አንዷ በበሽታ የተያዘች ከእነርሱ ጋር ስጫወት እንደነበር ይገለፃል፡፡ ለየወረዳው ባሉት ማቆያ እጅግ በተፋፈገ ሁኔታ ሰዎች መቆየታቸው በቂ ዝግጅት ሳይደረግ ብዙ ሰው ከመጣ እንዴት ይደረጋል ማቆያው በተገቢው ካልተዘጋጀ የባሰ በሽታውን የሚያሰራጭ እንዳይሆን ስጋት አለኝ፡፡

በአውንታዊ የሚገለጽ ንጽህናችንን እንድንጠብቅ መደረጉ ይህ በህግ ሆኖ ቢቀጥል ከንክኪ ነፃ መሆን ከንጽህና ጉድለት የሚመጡ በሽታዎችን የሚቀንስ መሆኑን አይተናል፤ ከምንም በላይ እጅን ሳይታጠቡ ቤት እንዳይገቡ ማድረግ፡፡

**ከኮሙኒኬሽን ሥራ ጋር ምን የተሻለ ነገር መጣ?**

አዳዲስ ፈጠራዎችን እንድንጠቀም አድርጐናል አዳዲስ አሠራሮችን እንድንፈጥርና እንድንጠቀም አነሳስቶናል፡፡ የመልእክት አቀራረጽ፣ አደረጃጀትና አነቃቂ ኀ/ሰቡን በሚያነቃቃ ሁኔታ እንዲሆን አጋዥ መሣሪያዎችን እንድንጠቀም ጭውውት ፈጥረናል፣ ተቋማችን በየጊዜው አዳዲስ መረጃ እንደመፈለጉ፣

የመረጃ ፍሰትና ማዕከላዊነትን ስለመጠበቅ፣ ኃላፊነትንና ተጠያቂነትን በጥንቃቄ መወጣትን

አዳዲስ ቴክኖሎጂዎችን ለስብሰባ መጠቀም፣ የመጣ ስብሰባ ወጪ እንድንቆጥብና ተነሳሽነትን አሳይቶናል፡፡

ቁልፍ መረጃ ሰጪ 4. ትምህርት መምሪያ

እንደትምህርት መምሪያ ተቋማችሁ ከኮሮና ቫይረስ በሽታ ጋር ያለው ቁርጭት ምን ይመስላል;

- ሌሎችን ከመደገፍ ከማስተባበር ከላኛውና ታጭኛው መዋቅር ጋር ያለው ግኑኝነትን በሚመለክት፡

ኮቪድ 19 በሽታ ጋር በተያያዘ የትምህርት ሴክተር ሰፊ ጉዳት ደርሶበታል፡፡ በሀገር ደረጃ እንደወረርሽኝ ከታወጀ ከመጋቢት ወር 2012 ጀምሮ የትምህርት ሚ/ር የትምህርት ሥራ መቋረጥ አለበት፤ተማሪ ትምህርት ማቆም አለበት፤ ካለ ጊዜ ጀምሮ እኛም ትኩረት የሰጠነው ወረርሽን ለመከላከል ነው፡፡ የመማር ማስተማር ሥራ እንደወትሮ ተማሪና መምህር ፊት ለፊት አገናኘተን ማስተማር ስለማንችል አንደኛው የመማር ማስተማር ሥራ እንደት ማስኬድ እንችላለን? **ሁለተኛው** ይህንን ወረርሽኝ እንደት መከላከል እንችላለን? በሚል ሁለቱንም አቀናጅተን የማስኬድ ሥራ ለማስኬድ ከክልልም ጀምሮ ግብረ ሀይል ተቋቁሟል፡፡በትምህርት ራሱን አስችሎ ክልላዊ ዞናዊ እስከወረዳና ትምህርተ ቤት ተደራጅተዋል፡፡ በትምህርት ቤት ደረጃ ደግሞ ባለድርሻ የሆኑት እንደሱርቫይዘር የትምህርት ክፍል ተጠሪዎች ዩኒቲ መሪዎች ያሉበት ግብረ ሀይል ተዋቅሮ በመከላከል ሥራ ትልቅ ሚና እየተወጡ ይገኛሉ፡፡ የመማር ማስተማር ሥራ መቋረጡ በራሱ እንደ አንድ የመከላከል ሥራ ተቆጥሮ ተማሪ ቤትም ትምህርት እስከሌለ ድረስ ቁጭ ማለት ስለማይችል መጫወት አለ መንቀሳቀስ አለ፤ ሆኖም በቡድን እንዳይንቀሳቀሱ ለማድረግ ከትምህርት ሚ/ር ጀምሮ የተዘጋጀው የቴክኖሎጂ ትምህርት በማጠናከር በሬዲዮና በቴሌቭዥን በመታገዝ ትምህርት እንዲሰጥ በማድረግ አካላዊ መራራቅን በመተግበር ተማሪዎች ትምህርት ቤት ሳይመጡ እቤታቸው ሆነው መማራቸው በሽታውን የመከላከል አንዱ አካል ነው፡፡ ይህም ማለት ተማሪው እቤት እያጠና እያነበበ የሚውል ከሆነ ከጓደኞች ጋር የመገናኘት አጋጣሚው ስለሚቀንስ በዚሁ ሁኔታ ተማሪው ራሱንና ቤተሰቡን መከላከል መልካም አጋጣሚ ነው ብለን እየሰራን እንገኛለን፡፡ ከዚሁ ጋር ተያይዞ ተማሪው ትምህርት ቤት መሄድ በመቆሙ ለስነልቦና ጉዳት እንዳይጋለጥ ትምህርትን በሬዲዮና ቴሌቭዥን እንዲከታተል እያደረግን ነው፡፡ ይህ ስራ ያለመቋረጥ ተጠናክሮ በዞናችን እሰከትምህርት ቤት ድረስ እንዲዘልቅ እኛ አስቀድሞ በመምሪያው ማኔጅመንት ደረጃ በሚገባ ተወያይተን እስከታች ባለሙያም ደረጃ ወርደው የመከላከል ሥራውን ለማጠናከር ግንዛቤ እንዲፈጠርና ተማሪዎች ምን እየሰሩ እንደሆነም ለማረጋገጥ በዚህ አስቸጋሪ ሁኔታ ውስጠም ሆነን ከመጋቢት ጀምሮ ሁለት ጊዜ መስክ በመውጣት ለወረዳዎች ድጋፋዊ ክትትልና እገዛ አድርገናል፡፡ ከዚያ ውጭ ደግሞ በግብረሀይል መልክ በተደጋጋሚ ወርደን የምናይበት ሁኔታ አለ፡፡ ከድጋፍም አንጻር በሽታውን የመከላከል ሥራን ከጤናና ሎሎች አጋር ከሆኑት መንግስታዊ ካልሆኑት ተቋማት ጋር በመቀናጀት ለንጽህና መጠበቂያ የሚሆኑትን እንደሳኒታይዘርና ፈሳሽ ሳሙና ለሁሉም የታችኛው መዋቅር እንዲደርስ እያደረግን ነው ፡፡

በዚህ ሥራ ሂደት ከናንተ ጋር በአጋርነት የሚሰሩት እነማን እንደሆኑ መጥቀስ ከታቻለ፡- በዚህ ሥራ ሂደት ከኛ ጋር በአጋርነት የሚሰሩት የተወሰኑት አሉት፡- ለመጥቀስ ያክል እንደ ሴፍ ዘቺልድረን የንጽህና መጠበቂያ ቁሳቁሶችን እንደ ሳኒታይዘርና ሳሙና የመሳሰሉትን በማቅረብ ያግዙናል፤ በተጨማሪ የግንዛቤ ማስጨበጫ የሚረዱትን ፖስተሮችን በማዘጋጀትና በማሰራጨት ይረዳሉ፡፡ በዚሁ ረገድ ከኛ ጋር በሰፊው እየሰራ ይገኛል፡፡ ሌላው ቻይ የሚባል ድርጅት ሲሆን ይህም ከኛ ጋር ተቀናጅቶ ቁሳቁስ ድጋፍ ከማድረግም አልፎ ቅስቀሳ በማድረግም ይበልጥ ህብረተሰቡ ራሳቸውን እንዲከላከሉ ተገቢ ግንዛቤ እንዲኖራቸው ለማድረግ በቂ መረጃ በመስጠት በመከላከሉ ዙሪያ ሰፊ ተሳትፎ በማድረግ ላይ ይገኛል፡፡ እንደዚሁም ደግሞ የክልል ትምህርት ቢሮ አስቀድሞ በዞናችን በትምህርት ቤት ምገባ ፕሮግራም በታቀፉት ሶስት ትምህርት ቤቶች በሁለተኛው ዙር የሚመጣውን ምግብ አሁን ትምህርት ስለሌለ ግብዐቱ ከኮቪድ መከላከል እንዲውል አቅጣጫ በማስቀመጡ ይህም በዞን አስተባባሪና ዐቢይ ግብረ ሀይል (በዞን አስተዳደርና ድርጅትም) እንዲሁም በሀብት አሰባሳቢ ኮሚቴም ታዉቆ ለሚመለከታቸው እንዲታደል ተደርገዋል፡፡ ይህም በሽተውን በቅንጅት ለመከላከል የሚደረግ ጥረት አካል ነው፡፡በዚሁ ልክ ሁለተኛ ዙርም አሁን ለማሰራጨት እየተዘጋጅን ያለበት ሁኔታ አለ፡፡ በተለይ በከተማ አካባቢ ለከፍተኛ የምግብ ችግር የተጋለጡ የህብረተሰብ ክፍሎች አሉ፡- እነሱን በተቻለ ልክ ለማገዝ ያለውን በቆሎ ቦሎቄና ጨው ለተማሪ ተብሎ የተዘጋጀውን ለዚህ ተግባር አውለናል፡፡ ይህም በሂደቱ በሽታውን ከዞኑ መንግሰት ከጤናና ሌሎች ከሚመለከታቸው ጋር በቅንጅት ለመከላከል የምናደርገው ጥረት አካል መሆኑ ነው፡፡ ብቻችን ደግሞ እስከታችኛው መዋቅር ድረስ በራሳችን ሴክተር እየተንቀሳቀስን እንገኛለን፡፡ በተለይ ደግሞ በበሽታው የተጠረጠሩት በለይቶ ማቆያ የሚቆዩበት ትምህርት ቤቶች እንደመሆናቸው ለዚህ ዓላማ ብቁ ናቸው ወይንስ ጉድለት አለ የሚለውን ወርደን እናያለን፤፤ በአስራ ስምነቱም መዋቅር ላይ የትኛው ትምህርት ቤት ለዚህ ተግባር ተመረጠ የሚለውን ተከታትለን ዝግጁነታቸውን በማረጋገት ለዐቢይ ኮሚቴ እናቀርባለን፡፡በዚህ መልክ እየንተንቀሳቀስን እንገኛለን፡፡

ይህ አብሮ መስራትና ቅንጅታዊ አሰራር ከበሽታው ባሕሪይ ጋር እንደት እንተመረጠ ከአስፈላጊነቱ አንጻር ጠለቅ አድርገው ቢገልጹ

ያው የበሽታው ባህሪይ ወረርሸኝ ነው እና ከዚያ አንጻር ተቀናጅተን ካልሠራን ልንወጣ አንችልም፡፡ ይህ በሽታ ዓለምን ያንቀጠቀጠና ያስፈራራ በሽታ እንደመሆኑ ተቀናጅተንና ተናብበን ካልሰራን ልንወጣው እንደማንችል በመገንዘብ ነው፡፡ የበሽታው መዛመትና ስርጭት ፍጥነት እንደቀድመው ኤክተሪያል በሆነና በተነጣጠለ አካሔድ ጤናው ለብቻ ትምህርት ለብቻ ሌላውም እንደዚያ የሚሄድ ከሆነ ስለማንወጣው ነው፡፡ በመሆኑ ምጥረታችን ተመጋጋቢና የምንሰራው ህብረተሰብ ያው አንድ ስለሆነ መተናጀትና መደጋገፍ አስፈላጊ ነው፡፡ ሁላችንም ለማገልገል የቆምንለት ያው አንድ ህብረተሰብ በመሆኑ ለምሳሌ ተማሪ ከተጎዳ ወዲያው ወላጅ ይጎዳል፤ ከዚያም ወደሌላው ህብረተሰብ ክፍል መዳረሱ አይቀርም ፤ይህን ለመከላከል ደግሞ አብሮ መንቀሳቀስ የግድ ይላል፡፡ የተገለጸው ነገር ቅንጅታዊ አሠራር አስፈላጊና ግዴታም እንደሆነ በሚገባ ተብራርተዋል፤ ነገር ግን ይህንን የሚያስረዳ ማስረጃ ወይንም ሰነድ አላችሁ መነሻስ የሆነ ምንድነው?

ይህንን እንቅስቃሴ የምንመራበት የጋራ ዐቢይ ግብረሀይል ደረጃ ዕቅድ አለን ፡፡ ትምህርት የዐቢይ ኮሚቴ አባል ሆነው የጋራ ዕቅድ ከዘጋጀ በሓላ ደግሞ እንደትምህርት የራሱን ግብረ ሀይል እስከታች በማቋቋም ከዋናው ዕቅድ መነሻነትና ከክልል ትምህርት ቢሮም የወረደውን በኛ ነባራዊ ሁኔታ ቃኝተን የራሳችንን ዕቅድ አዘጋጅተን እንተገብራለን፡፡

ይህ የጋራና የሴክተር ዕቅድ ምን ያክል የተናበቡ ናቸው? የት ላይ ነው ቁርኝታቸው የሚታየው?

እጅግ በጣም ይገናኛል፡፡ ለምሳሌ አንድ የተጠረጠረውን ግለሰብ የምናቆይበትን ቦታ መምረጥ የዐቢይ ኮሚቴ ሥራ ነው፡፡ ዐቢይ ኮሚቴ የሚመርጠው ትምህርት ቤትን ነው፡፡ ግን ትምህርት ቤቱ በትምህርት መምሪያ በኩል ስለሚተዳደሩ ያለውን የዝግጁነት ሁኔታና ለህዝቡ ያለውን ቅርበት አስቀድሞ በማየት ማረጋገጥ ከኛ ስለሚጠበቅ ና ቢያንስ ቢያንስ በቀበሌ ደረጃ ትምህርት ቤት ስላለ ሌላ ተቋም ተከትሎ ስለሚመጣ የእኛ ሚና ቀዳሚ ይሆናል፡፡ ይህም ካሉት አደረጃጀቶች ሁሉ በተሸለ ደረጃ ተደራሽ የሆነ ተቋም ትምህርት ቤት ስለሆነ ካለው የአካባቢ አቀማመጥም አንጻር ትምህርት ቤቶችን ነው፡፡ በዚህና ሌሎችም የግንዛቤ ማስጨበጫ ሥራ ላይ በጋራ ተቀናጅተን እንሰራለን፡፡

ያቀዳችሁት እቅድና ትግበራ ወደ ታለመለት ግብ እንደሚያደርስ ምን የመከታተያ ስልት አላችሁ?

እንደትምህርት ተቋም ሁለት ሥራ እንደምንስራ ቅድም ጠቅሻለሁ፡፡ አንደኛ የወረርሽን መከላከልና ሁለተኛው ደግሞ የተቋረጠውን የትምህርት ሥራ የማስቀጠል ናቸው፡፡ ሙሉ በሙሉ ዝግ እንዳይሆን ተማሪ በቤቱም ሆነው ትምህርት መከታተል አለበት ብለን ስንከታተል ቆይተናል፡፡ ትምህርት በሬዲዮና በቴሌቭዥን የሚከታተሉበት ሁኔታ በቼክ ሊሰት አዘጋጅተን የዚያ ቼክሊስት አካል የምናደርገው አንዱ ለዚህ ሥራ ግብረሀይል ተደራጀተዋል ወይ? በትምህርት ጽ/ ቤት ና በትምህርት ቤት ደረጃ የተዘጋጀ ግብረ ሀይል መኖሩ ፤ግብረ ሀይሉ ወደሥራ ገብተዋል ወይ ወደስራ መግባቱን የምናረጋገጥበት የቼክሊስት አካል አድርገን ይህንን የትምህርት በሬዲዮና ቴሌቭዥን ትምህርት ስርጭት የምንከታተልበት ሰነድ አዘጋጅተናል በዚሁ መልክ፡፡

ከዚሁ ጋር በተያያዘ ከክልልና ወረዳ ጋር በጋራ የመገምገም ልምድስ ምን ይመስላል?

የጋራ መድረክ ይፈጠራል፡፡ በአካል ከክልል ጋር መገናኘት ባሁኑ ሁኔታ ባይቻልም በዙም ስብሰባ እንገናኛለን፡፡ ከክልሉ ትምህርት ቢሮ ጋር በሳምንት አንድ ጊዜ በመገናኘት እነርሱም ከላይ ከትምህርት ሚ/ር የሚቀበሉትን ወደእኛ ያስተላልፋሉ፡፡ እኛም ያንን ተቀብለን ቼክሊስት አዘጋጅተን ወደታች እናወርዳለን፡፡

በናንተ መምሪያ የኮቪድ -19ን ምላሽ ለመስጠት የተለየ ሰው ተመድበዋል ወይ?

አው አለ፡፡ ይህንን ግብረሄል የሚመራ የዞኑ ትምህርት መምሪያ የትምህርት አመራርና የሠራተኛ አስተዳደር ነው፡፡ ይህ አደረጃጀት እስከወረዳ በዚሁ ልክ ወርደዋል፡፡ ምክምያቱም የተቋሙ ዋና ሀላፊ በተደራራቢ ሥራ ስለሚያዝ ውጤታማ ለመሆን እንደዚህ ዓይነት አደረጃጀት መፍጠር ይበልጥ ውጤታማ ያደርጋል፡፡ በደረጃው ደግሞ የተጠነከረ ኮሚቴ አለ፡፡ በዚህ ደረጃ የሚያከናውኑትን ሥራ በተዘጋጀው ቼክሊስት መሥረት በጋራ ከመምሪያ ሀላፊ ጋር እንገመግማለን፡፡ ይህንን ግምገማ በየሳምንቱ እየተገናኘን ነበር የምንገመግመው፡፡

ለሥራው የሚያስፈልገውን ግብዐት ከመመደብና ከማግኘት አንጻር እንደት ነው የምንትቀሳቀሱት?

አስፈላጊ ግብዐት ከማግኘት አንጻር ከዞንም ድጋፍ ይደረጋል፡፡ ሌሎች መንግስታዊ ያልሆኑት ድርጅቶችም እንደሳኒታይዘር ፈሳሽ ሳሙናና የመሳሰሉትን ያቀርባሉ፡፡ ይህንን ተቀብለን ለተወሰኑት ሴክተሮች ያሰራጨንበት ሁኔታ አለ፡፡ እንዲሁም ታች ላሉት አስራ ስምነቱም መዋቅሮች እንደፖስተረ ያሉትንም አሰራጭተናል፡፡ ከዚህ ውጭ ግን ከክልል ቅድም ከተጠቀሰው የትምህርት ቤት ምገባ ግብዐት ውጭ የመጣ ግብዐት የለም፡፡

ይህ የኮቪድ-19 በሽታ ዘርፈ-ብዙ የሆነ ጉዳት ይዞ እንደመጣ ይታወቃል በሌላ ጎኑ ደግሞ ይህንን በሽታ ለመከላከል በምናደርገው ሂደት ያገኘነው መልካም ተሞክሮ አለ ወይ?

ምንም እንኳን ችግር ሆኖ ቢከሰትም እያስተማረን ያለው በርካታ መልካም ጎኖች አሉት፡፡ ለምሳሌ፡- እንደትምህርት ሴክተር በርካታ የተማርናቸው ነገሮች አሉ. ትምህርት በሬዲዮ ከዚህ በፊትም ትምህርት የምንሰጠበት አንዱ ዘዴ ነበር፤ በዚያን ጊዜ በሬዲዮ የሚሰጠው ትምህርት ውጤታማ እንደሆነ የወረቀት ሪፖርት ያመለክት ነበር፤ አሁን ግን መደበኛ የፊት ለፊት ትምህርት ተቋርጦ ሙሉ በሙሉ በሬዲዮና ቴሌቭዥን ከሆነ በሃላ ብዙ ነገር እንድናይ አድርጎናል፡፡ ይህም ባሁኑ ጊዜ ወላጆች ለልጆቻቸው ሬዲዮ መስራት አለመስራት ከአንድ ቦታ ወደሌላ ቦታ እየዞሩ የሚያረጋግጡበት ዕቃው ከሌለ መግዛት የትኛው መስመር ወይንም ኤፍ ኤም ስርጭት የተሻለ ተደራሽ እንደሆነ ተከታትሎ የሚያረጋግጡበት፤ ከዚህ በፊት በሪፖርት የሚቀርቡትና አሁን በተግባር ያየነው የተለየ ተሞክሮ መሆኑ ነው፡፡ ከዚህ ጋር ተያይዞ የቴክኖሎጂ አጠቃቀማችን ከፍ እያለ መሆኑ ከክልል ጋር ከዚህ በፊት በአካል እየተጓዝን የምናደርገው ሪፖርት ቅብብል አሁን በዙም ስብሰባ ካለንበት ሆነን ማከናወናችን ወጪ ቆጣቢና በመረጃ ልውውጥ ሂደት ፍጥነት የጨመረ መሆኑን አስተውለናል፡፡ ሌሎች ፈጠራ ሥራዎችን ከመቀስቀስ አኳያም ለምሳሌ መድሃኒት ፍለጋ በየሰፈሩ ወጣቶችና ሕጻናት ሳይቀሩ የተለያዩ ሙከራ ውስጥ መግባታቸው ገና በአንደኛ ደረጃ ያሉት ተማሪዎች ቅጠል እየበጠሱ ሀይላንድ ይዘው የሆነ መድሃኒት እየሞከርኩ ነው እያሉ መንቀሳቀሳቸው ባጠቃላይ የትምህርት ተቋማት ከአንደኛ ደረጃ ጀምሮ እስከዩኒቨርሲቲ ድረስ ያሉት በምርምር ሥራ ተሳትፎ እንዲያድግ ማድረጉ ይገለጻል፡፡ በተግባር ስራ ውስጥ እንዲገቡ መደረጉና የቴክኖሎጂ አጠቃቀምም ቡራቡሬ የነበረው እንዲዳብር ማድረጉ ይጠቀሳል፡፡ ለምሳሌ የትምህርት በሬዲዮ ፕሮግራም ውጤታማነትን ስንገመግም ለክተን ፐርሰንት አውጥተን ያየንበት በዞናችን ምን ያህል ተጠቀመ፤ የሽፋን እና ጥራት ደረጃ ምን ይመስላል? በሚል በቅርቡ ከትምህርት ቤት ጽ/ ቤቶች ሃላፊዎች ጋር መድረክ ፈጥረን የተወያየንበት ሁኔታ ነበር፡፡ ከዚህ በፊት የነበርው የቴክኖሎጂ አጠቃቀምና አሁን ያለውን ልዩነት እንዲያዩ አድርገናል፡፡ከዚህ በፊት ሲደረግ የነበርው ሪፖርት ምን ይመስል ነበር አሁን ግን በተግባር ሌላው ሁሉ ዝግ ሆኖ በሬዲዮ ብቻ ትምህርት ይሰጣል ሲባል የተፈተኑ መዋቅሮችም ነበሩ፡፡ በትክክል ተደራሽ መሆን ያለመሆን በተግባር ሣያረጋግጡ የወረቀት ሪፖርት ሲያደርጉ የነበሩት አሁን ግን ደንግጠው የተማሪዎች ወላጆች ፋታ ሳይሰጡአቸው ከዚህ በፊት ሬዲዮ የሌላቸው ሬዲዮ ወይንም ሞባይል የገዙበት ሁኔታ ነበር፡፡ከቴክኖሎጂ አጠቃቀም አንጻርም ጠንካራ ጎን ታይተዋል፤፤ ሌላው ደግሞ ሰው የራሱን ጤንነት ከመጠበቅም አንጻር ከዚህ በፊት ከነበረው ልምድ በተለየ እጅ መታጠብ ምን ያህል ጥቅም እንዳለው ሰው በጣም የተገነዘበበት ምንም እንከዋን ፐሮግራሙ ከዚህ በፊትም በዋሽ ቢኖርም የዚህን ያክል ለውጥ አልነበረም አሁን ግን ከልጅ እስከ አዋቂ ድረስ እጁን ባግባቡ የሚታጠብበት ሁኔታ እየዳበረ ይገኛል፡፡ ይህ ደግሞ ለቀጣይም የተሻለ የጤና ባሕሪይ እንዲኖር መሠረት እየጣለ ነው ማለት ይቻላል፡፡

ቅንጅታዊ አሠራርን ከማጠናከር አንጻርስ በጎ አስተዋጽኦ ኖሮት ይሁን?

አው እንግዲህ ኮቪድ ጠላት ነው፤ ጠላትን ለመመከት ደግሞ አብሮ መቆም የግድ ይላል፡፡ በዚሁም እያንዳንዱ ሴክተር መምሪያዎች መቀናጀት ምን ያክል አስፈላጊ እንደሆነ አይተናል፤ እንዴት ይቀናጃል ለሚባለው በኛ ደረጃ ያየንበት አጋጣሚ አለ፡፡ ለምሳሌ እንደኛ ወላጅ አልባ ህጻናት ና ወላጅም እያሉ አቅም የሌላችው ህጻናት አሉ ፤፤ የእነዚህ ልጆች ትምህርት ጉዳይን በሚመለከት ከ ጤና እርሻና ተፈጥሮ ሀብት ልማት ሠራተኛና ማህበራዊ ጉዳይ መምሪያዎች ጋር በመቀናጀት መሥራት ምን ያክል ጠቃሚ እንደሆነ አይተናል፡፡ ከመረጃ አንጻርም ለምሳሌ ልዩ ፍላጎት ያላቸውን ተማሪዎችን ከማግኘት አንጻር ከሠራተኛና ማህበራዊ ጉዳይ መምሪያ ከሌሎችም ጋር በመቀናጀት ውጤት ያየነበት አግባብ ነበር፡፡ የመረጃ ተአማኒነትን ከማረጋገጥም አንጻር ለምሳሌ ልዩ ድጋፍ የሚያስፈልጋቸው ሕጻናትን በሚመለከት እኛጋ ያለው ና ከሠራተኛና ማህበራዊ መምሪያ ያለው መረጃ ተናባቢነት ለማረጋገጥ ይህ ቅንጅታዊ አሠራር ይደግፋል፡፡ ታችኛው መዋቅር በጋራ ወርደን የመረጃ ታአማንነትን ለማረጋገት የሰራነው ሥራ የቅንጅታዊ አሠራር ውጤታማነትን ያሳየ በመሆኑ ቅንጅታዊ አሠራር ለተሻለ ውጤት እንደሚያበቃ ልምድ የተወሰደበት ነው፡፡

እስካሁን በተመጣበት መንገድ ቅንጅታዊ አሠራር አኳያ እንደጉድለት የታየ ሁኔታ ካለ ቢገለጽ

እንደጉድለት የገመገምነው ነገር በመጀመሪያ አካባቢ የነበረው ጥንቃቄ ሥራ አሁን እየተሰራ ያለው ከትኩረት አንጻር አሁን ትኩረት ማነስ ይታያል፡፡ ከኛም ጀምሮ በህብረተሰብ ደረጃም ስናይ ትኩረት እየቀነስ መምጣት ይታያል፡፡ በሽታው እየጨመረ ነው፡፡ ያነ ገና በሽታው ገና ሳይገባ የነበረውና አሁን የበሽታ ስርጭት እየጨመረ በመጣበት ጊዜ የሚታየው ጥንቃቄ እኩል ያለመሆንና በእኩል ሃይል ያለመንቀሳቀስ መስተካከል አለበት፡፡ በሽታው እስካለ ድረስ ዕረፍት የሚሰጥ አይደለምና፡፡ የኛ የመከላከል ሥራ በዚሁ ልክ እየተጠናከረ መሄድ ሲገባ መዘናጋት አለ፡፡ መዘናጋቱ ከሁለቱም አቅጣጫ ነው፡- ከሚመራውና ከህብረተሰብ ዘንድም ጭምር ነው፡፡ ከበሽታው ገዳይነት ይልቅ ወቅታዊ በሆኑ ሁኔታዎች መወሰድ፤ሳልሰራ እንዴት እቀመጣለሁ ማለት መኖር አለብኝ ከማለት ይልቅ መስራት አለብን የሚል ህብረተሰብ መሆኑ፤ ከስራ አንጻርም ደህንነት ተጠብቆ እንዴት መስራት እንዳለባቸው አስቀድሞ የግንዛቤ ሥራ መሰራት አስፈላጊ በመሆኑ እንደትምህርት መምሪያ ለበሽታው ተጋላጭ የሚሆኑትን የትምህርት ማህበረሰብ እቤታቸው እንዲቆዩ አድርገናል፤ ሆኖም ግን በሌላው አካባቢ እንደዚሁ ህ/ሰቡን ለበሽታው የሚያጋልጡ ስብሰባዎች ትኩረት ተሰጠቶ እየታገዱ ያለመሆን ይታያል፡፡ አንዳንድ ወጣ ገባ ያሉ ነገሮች ለምሳሌ እንደ ትራንስፖርት ያሉት መስተካከል ያለባቸው ናቸው፡፡ አሁን ባለንበት ሁኔታ የበሽታው ስርጭትና ሞት ከቀን ወደ ቀን በከፋ ሁኔታ እየጨመራ ያለውን ያክል የኛም ዝግጅትና ጥንቃቄ ከዚህ በላይ መሆን ይጠበቃል ብዬ አምናለሁ፡፡ ሆኖም ግን በዚያ ልክ አይደልም፡፡ መጀመሪያ አንድና ሁለት ሰው እንደኬዝ ተመዝግቦ በነበረነት ወቅት የሚደረገው ጥንቃቄ ባሁኑ ሰዓት እየተደረገ አይደለም፤ ይህንንም እንደዞን እንደክልልና እንደሀገርም መታየት አለበት እላለሁ፡፡ አስቀድሞ ሰው ለመስራት በህይወት መኖር ያስፈልጋል፡፡ አሁን የሚታየው ሥራን የማስቀደም ሁኔታ ጥንቃቄ ካልተደረገ ምናልባት በሽታውን ከቁጥጥር ውጭ እንዳያደርግ ስጋት አለኝ፡፡

ቁልፍ መረጃ ሰጭ 5. ፋይናንስ መምሪያ

1. የፋይናንስ መምሪያ በዞኑ የኮቮድ-19 በሽታ ከመከላከልና ቁጥጥር ሥራ አንጻር ያለው ቁርኝት ምን ይመስላል?

- ከተለያዩ መዋቅሮች ጋር ወደላይና ታች እንዲሁም ወደጎንዮሽ ያለው የሥራ ግኑኝነት
- ተቋማዊ አደረጃጀትን ለምላሹ ብቁ ከማድረግ አንጻር

እንግዲህ እንደሚታወቀው ኮቪድ -19 ዓለም አቀፍ ወረርሽኝ ተብሎ እንደሀገርም ሆነ እንደክልል ከታወቀ በርካታ ሥራዎች ሲሰሩ ቆይተዋል፡፡ ይህንንም መነሻ በማድረግ የክልሉ መንግስትም ሆነ የክልሉ ብልጽግና ፓርቲ ጽ /ቤት በሸታውን በጋራ ለመከላከል ባወረደው አደረጃጀት መሠረት የኛ ዞንም አደረጃጀቱን ተግባራዊ በማድረግ ረገድ አደረጃጀቱን እስከታችኛው መዋቅር አውርደዋል፡፡ ፋይናንስ የዚህ አደረጃጀት አንዱ ክንፍ በመሆን የሀብት አሰባሰብና በጎአድራጎት ንዑስ ኮሚቴን በበላይነት እንዲመራ ተደርገዋል፡፡ ስለዚህ ፋይናንስ ትልቅ ሥራ እየሠራ ይገኛል፡፡ ከዚያ ባለፈ የተለያዩ ባለድርሻ አካላት ቀድሞ መከላከልም እንዲሁ ደግሞ ከበሽታው ጋር ተያይዞ ሊያመጣ የሚችለው በተለይ ኢኮኖሚያዊ ችግሮች ቀድሞ ለመከላከል ተብሎ ሀብት በማሰባሰብ ረገድ የተለያዩ አካላት ድጋፍ እንዲያደርጉ እያስተባበረ ይገኛል፡፡ እንደተቋም ደግሞ አገልግሎት ሰጭ እንደመሆኑ ከፍተኛ የሆነ ከደንበኞች ጋር ግኑኝነት ስላለ በጋራ ለሠራተኞቻችን ግንዛቤ በመስጠት ጥንቃቄ እንዲያደርጉ ተደርገዋል፡፡ለምሳሌ የእጅ መታጠቢያ ቁሳቁስ በሁሉም የዞናችን መዋቅሮች እንዲኖር በማድረግ ሴክተር መስሪያ ቤቶች ከእጅ ንክኪ ነጻ በሆነ ሁኔታ እጃቸውን እንዲታጠቡ የሚያስችል ቁሳቁስ ገዝተው አሰራጭተዋል፡፡ሳሙናዎችን ማስኮችንም ገዝተው ከዞን እስከወረዳና ከተማ አስተዳደር ላሉት መዋቅሮች አሰራጭተዋል፡፡ ከዚያ ባለፈ አገልግሎት ፈላጊ ወደ ተቋማችን ሲገባ የፊት መሸፈኛ ማስክ ካላደረገ እንዳይገባ የማድረግ ግንዛቤ ፈጥረን እየተገበርን እንገኛለን፡፡ ይህንን አሰራር ሁሉም እንዲተገብሩት በሰርኩላር አስተላልፈናል፡፡ እንዲሁም አጅንም ሳይታጠብ ሰው ወደተቋማችን እንዳይገባ ተደርገዋል፡፡ ከዚያ ባለፉ በዕድሜ የገፉት የመስሪያ ቤታችን ሠራተኞች እንዲያርፉ አድርገናል፡፡ተመሳሳይ አሠራር በወረዳና ከተማ አስተዳደሮች እንዲዘረጋ በሰርኩላር አስተላልፈናል፡፡ ባለሙያዎች ወርዶ ድጋፍም እንዲያደርጉ ተደርገዋል፡፡ የሀብት ማሰባሰቡ ሥራ በየመዋቅሩ ተጠናክረው እንዲቀጥል አድርገናል፡፡ በመሆኑም ፋይናንስ የኮሮና ቫይረስ በሽታን በመከላከል ሂደት ውስጥ የጎላ ሚና የሚጫወት ተቋም ነው፡፡

በዚህ ሂደት ውስጥ ከይላኛው ማለትም የክልል ፋይናንስ ቢሮ ጋር ያለው መስተጋብር ምን ይመስላል?

ቅድም እንዳነሳሁት ዐቢይ ኮሚቴ በዞኑ ዋና አስተዳዳሪ ነው የሚጠረነፈው በስሩ ከስምንት እስከ አስር የሚሆኑ ንዑሳን ኮሚቴዎች አሉ፡፡ ስለዚህ መጋቢት 4 2012 ዓ.ም. በጤና ሚኒስቴር በኩል በሀገራችን በበሽታ የተያዘ ሰው መኖሩ ከተገለጸ በኋላ ሥራው በዐቢይ ኮሚቴ እየተጠረነፈ በየሶስት ቀን እየተገመገመ አቅጣጫ እየተቀመጠ ሀብት የማሰባሰብ ሥራውም በዚሁ አግባብ የተመራበት ሁኔታ ነው ያለው፡፡ ስለዚህ ለብልጽግና ፓርቲም ሆነ ለአስተዳደር ሪፖርት በጽሑፍና በስልክ ከማድረግም ባሻገር የተሰበሰበው ሀብት በአካል በመገኘት መጎበኘትና በጋራ እየገመገምን ቆይተናል፡፡ በዚህ ሂደት ከፍተኛ መናበብ አለ፡፡ ከዚያ ውጭ ወደላይ ከክልል ጋር የሀብት አሰባሳቢ ኮሚቴ ሰብሳቢ አቶ ጥላሁን በነበረ ጊዜ በቪዲዮ ኮንፈራንስ እየተገናኘን አቅጣጫ ይቀመጥ ነበር፡፡ ከዚያ በኋላ ፋይናንስ ቢሮ ሃላፊ የኮሚቴ ሰብሳቢ ከሆነም በሓላ ዘውትር ሀሙስ 4፡00 ላይ እየተገናኘን እንወያያለን፡፡ ከዚያ በሓላ አሁን ተሻሽሎ በዙም እንሰበሰባለን፡፡ ይህ ማለት የሀብት አሰባሰብ ሂደት በምን ደረጃ እንዳላ በየጊዜ እየተገናኘን ሀሳብ የምንለዋወጥበት መድረክ ነው፡፡ከኛ ለምነሱ ጉዳዮች ክልሉ በርካታ ምላሾችን ሰጥተዋል፡፡ ለአብነት አሁን አስቀድሞ የሀብት ማሰባሰብ ሁኔታ በዞን ብቻ ተንጠልጥሎ የነበረውን ወደታች ወረዳዎች የማውረድ፤ አካውንት በየወረዳው እንዲከፈት የማድረግ ሥራ አቅጣጫ መቀመጡ ምክንያቱም በሽታው በታች ሲከሰት ሀብት ፍለጋ ወደዞን መምጣት አይቻልም፤ ስለዚህ ይህንን ለማቃለል በታችኛው መዋቅር ሀብት እንዲያዝ ተደርገዋል፡፡ ከዚያ ባለፈ የሀብት ማሰባስብ ባሻገር የግዥ የክፊያና ወጪ ሥርአቱ የፋይናንስ አሰራርን በተከተለ እንዲሆን አድርገናል፡፡ ይህ መመሪያ ከክልል ካስኬድ ተደርጎ መውረዱ ትልቅ እፎይታ ከመስጠቱም በላይ የህዝብ ሀብት እንዳይባክን ለአደጋ እንዳይጋለጥ እንዳይመዘበር ቁልፍ ሆኖ ጠቅሞናል፡፡ ይህም የሆነው ከክልሉ ጋር ተናብበን በመስራታችን ነው፡፡ አስቀድሞ በቪዲዮ ኮንፈራንስ ቀጥሎም በዙም ስብሰባ እየተገናኘን ተግባራችን እያሳለጥን እንገኛለን፡፡ ከክልል በሀብት ደረጃ ተቆጥሮ የመጣ ነገር ባይኖርም ከአሰራር ደረጃ መልካም የሚባል ግኑኝነት እንዳለ ማረጋገጥ ይቻላል፡፡

ከላይ የተጠቀሱት የአሠራር ሂደቶች መነሻ የሆኑት አዋጆች ደንቦችና መመሪያዎች ካሉ እና ይህም በዞን ደረጃ በምን መልኩ እንደተቀየሰ ቢገለጽ?

መልካም፡- እንግዲህ ይህ በሽታ አስከፊና ትውልድ ገዳይ የሆነ በሽታ ነው፡፡ በጋራ ካልተከላከልን አደጋ ነው፡፡ የኢኮኖሚያችንም ሁኔታ ይታወቃል፤ ኢኮኖሚያችን እንደሀገር የተቀዛቀዘ ነው፡፡ ስለዚህ ይህን ኢኮኖሚ ቢያንስ ማህበረሰቡ ካልደገፈ አደጋ ስለሆነ ለሀብት ማሰባሰብ ማህበረሰቡን በስፋት አነቃንቀናል፡፡ በተለይ የተለያዩ ማህበራዊ መሰረቶች ናቸው በሀብት ማሰባሰብ ሥራ የተሳተፉት፡፡ ለምሳሌ፡- የመንግስት ተቋማት መንግስታዊ ያልሆኑት ድርጅቶች ከፍተኛ የትምህርት ተቋማት ድጋፍ አድርገዋል፡፡ የንግዱ ማህበረሰብ የሀይማኖት ተቋማት በርካቶች ተሳትፈዋል፡፡ ይህ የተሰበሰበው በዓይነትና በጥሬ ገንዘብ ነው፡፡ በዓይነት የተሰበሰበውን ራሱን አስችሎ ጽዱ በሆነ መጋዘን እንዲቀመጥ በማድረግ የወጭና ገቢ አሰራር ራሱን ችሎ ባለሙያ በመምሪያ ሃላፊ ተመድቦ የራሱ አቃፊ በማበጀት ቁጥጥር እየተደረገ የሚተገበርበት ሥርዐት ተበጅተዋል፡፡በካሽ ለሚሰበሰበው በዞን ማዕከል የኮቪድ-19 አካውንት ተብሎ ተከፍተዋል፡፡ በተመሳሳይ በሌሎች ሁሉም ወረዳዎች አካውንት ተከፍተዋል፡፡ ዞን ላይ ባለው ሁኔታ ያንን መመሪያ እንዴት ተጠቀማችሁ ላላችሁ ለምሳሌ የኮቪድ -19 ሂሳብ የሚያንቀሳቅስ አንድ የሂሳብ ባለሙያ መድበን እንድናሰራ መመሪው በሚፈቅደው መሰረት አድርገናል፡፡ ይህንንም የኮቪድ-19 ሂሳብ የሚሰራ ሁሉም ተጠቃሚ እንዲያውቀው አድርገናል፡፡ ለዚህ ሂሳብ ማንቀሳቀሻ ቼክ አዘጋጅተናል፡፡ ከዚያ ውጭ ደግሞ ለእለት ተዕለት ፍላጎት ፐቲካሽ አሰራር ዘርግተናል፡፡ ይህም ለአስቸኳይ ስራ ማለትም እንደ ደም ናሙና ለሚያጓጉዙ አሰቸኳይ ግዥ በሚያስፈልግበት ጊዜ እንዲያገለግል ታስቦ ነው፡፡ መመሪያው እንደሚያዘው ለፐቲ ካሽ እስከ 50000 ብር ይፈቅዳል፤ከዚሁ ጋር ተያይዞ በአንድ ጊዜ ከ5000 ብር በላይ ግዥ እንዳይከናወን ገድቦ ነው የፈቀደው፡፡ ከዚያ ውጭ በኮሚቴ እየተወሰነ ይከናወናል ይላል፡፡ በተጨማሪ ለዚሁ አገልግሎት ግዥ የሚፈጽም ሰራተኛም መድበናል፡፡ ንብረት ክፍል አለ እንዲሁም ገንዘብ ያዥም ጭምር፡፡ ባጠቃላይ ለዚህ ሥራ አራት ባለሙያዎች ተመድበው የዕለት ተዕለት እንቅስቃሴ ያከናውናሉ፡፡ ይህ ማለት የአሰራር ሥርዓትን ዘርግተናል ማለት ነው፡፡ የግድ እኔ እንደመምሪያ ሃላፊ ኖረኩም አልኖርኩም በዚሁ ባዋቀርነው መሰረት ሥራው ይከናወናል ማለት ነው፡፡ ይህ ደግሞ የሚያሳየን ሥራው መደበኛ የፋይናንስ አሰራርን ተከትሎ እንደሚተገበር ነው፡፡ ከዚህ ባሻገር መመሪያው ጥሩ ነገር የፈጠረልን ከድንገተኛ እንቅስቃሴ ማዕከል አገልግሎት ጋር በተያያዘ የማደራጀት ሥራ በፍጥነት እንድናሟላ አድርገዋል፡፡ ለምሳሌ ለማዕከሉ የማቀዝቀዣ ጄኔሬተር ላውንደሪ ማሽን እና ሌሎችንም አስፈላጊ ግብዓቶችን በተፈለገው ፍጥነት እንድንገዛ አስችሎናል፡፡ ይህ በመደበኛ የግዥ አካሄድ ይፈጸም ቢባል የማይቻል ነበር፡፡ የግዥ ጥያቄ ከጤና መምሪያ እንደቀረበ ባስቸኳይ እንድንገዛ ዕድል መፍጠሩ እጅግ እጅግ በርካታ ችግሮችን አቃሎልናል፡፡

ከላይ የመጣ መመሪያ ነው ይህንን ሥራ ያሳለጠልን፡፡ በኛ በኩል የጨመርነውም ሆነ የቀነስነው የለም፡፡ ይመስለኛል ይህ ዓይነት አሰራር ድጋፍ አግኝቶ የወጣው አስቸኳይ አዋጅ መነሻ አድርጎ እንደሆነ አስባለሁ፡፡ የመመሪያው ጥቅም ስራውን ከማሳለጥ ባሻገር በፈጻሚ አካላትም ላይ እምነት እንዲጣል አድርገዋል፡፡ ወጭው የሚታዘዘው በዞኑ ዋና አስተዳዳሪ ወይንም ምክትል ዋና አስተዳዳሪና የጤና መምሪያ ሀላፊ በኩል እንጂ እኔ ራሴ እንደፋይናንስ መምሪያ ሀላፊ ማዘዝ አልችልም፤ የኔ ድርሻ ጥያቄው ከላ ታዞ ሲመጣ ሂደቱን ማሳለጥ ነው፡፡ ሌላው መመሪያው ካገዘን ውስጥ የሂሳብ አመዘጋገብ ግልጽ እንዲሆን አድርገዋል፡፡ በሀብት አጠቃቀም አኳያም ለምሳሌ የተሰበሰበውን እህል እንደጥራጥሬ ማካሮኒ ፓስታ በቆሎ የመሳሰሉትን ሌሎች እንደአልባሳትና የንጽህና ግብአቶችን ጨምሮ የምናሰራጭበት የአሰራር ስርዓት አስቀመወጠዋል፡፡ እነዚህ ንብረቶች በጊዜ አገልግሎት ላይ ካልዋሉ ሊበላሹ ስለሚችሉ በጊዜ ለተጠቃሚ እንዲደርሱ ለማድረግ የተጠቃሚዎች ሊየታ ሥራ ከሚመለከታቸው ባለድርሻ አካላት ማለትም እንደሴቶች ህጻናትና ወጣቶች ጉዳይ መምሪያና የሰራተኛና ማህበራዊ ጉዳይ መምሪያ ጋር በመሆን ይልቅ ተጋላጭ የሆኑት ተከናውኖ የንብረት ዕደላ ተከናውነዋል፡፡ ለምሳሌ አንድ ወረዳ የራሱን ተጠቃሚ ሊየታ ይልካል፡ ይህም ደግሞ እዚህ ባሉት የግብረ ሀይል አባላት ሲረጋገጥ ዕደላ ይከናወናል፡፡ መቼም ሀብት ውስን እንደመሆኑ በቀረበው ጥያቄና ባለን ሀብት መነሻነት ኮታ በማዘጋጀት ይታደላል፡፡ አንዱ ወረዳ 20 ኩንታል ሌላው 10 ኩንታል እንደ ተረጂ ቁጥር ይሰጠዋል ማለት ነው፡፡ መመሪያው መምጣቱ እጅግ ሥራችንን አግዞናል፤፤ አስቀድሞ መመሪያ ባልተዘጋጀ ጊዜ የገባው ሽንኩርት ተበላሽቶብን ነበር፡፡

መመሪያው ለኛ የተላከው ከክልል ፋይናንስ ቢሆንም የተዘጋጀው በፌዴራል የገንዘብ ሚንስቴር ነው፡፡ የክልሉ ፋይናንስ ቢሮ በሸኚ ደብዳቤ ላከልን፡፡ በዚህ ጊዜ መልካም አጋጣሚ ነው የሚለው ከብዙ ዘርፎች ድጋፍ ማግኘታችን ነው፡፡ ሌላው ይቅርና ተቃዋሚ የፖለቲካ ድርጅቶች እንኳ ሳይቀር በሚገርም ሁኔታ ተባብረዋል፡፡

እንደፋይናንስ መምሪያ የኮቪድ-19 መከላከልና ቁጥጥር ሥራ እንዲያስተባብር የተለየ ሰው ተመድበዋል ወይስ እንደት እያደረጋችሁ ነው፡፡

እኔ እንደዞን የዐቢይ ግብረ ሀይል አባልና የሀብት ማሰባሰብ ንዑስ ኮሚቴ ሰብሳቢ ሆኜ ነው የሚሰራው፤የመምሪችን የውስጥ መከላከልና ቁጥጥር ሥራን በሚመለከት የመምሪያው የሰው ሀብት ልማትና አስተዳደር ዳይሬክተር ነው፡፡ይሔውም በተቋሙ ውስጥ የመከላከል ሥራ ባግባቡ መከናወንን ይከታተላል፡፡ ለምሳሌ የእጅ መታጠቢያ ቁሳቁሶች መኖራቸውን ውሀ መኖሩን ሠራተኞችና ተገልጋዮች ወደ መምሪያው ሲመጡ የአፍ መሸፈኛ ጭንብል ማድረጋቸውን ይህንን፣ የጥበቃ ሠራተኞች እየተከታተሉ የሚያስፈጽሙ መሆናቸውን ይከታተላል፡፡ እይ፡ አልኮል በቢሮየ አሁን አለ፡ ባይኖርና ቢጓደል እየተካታተለ ያስሞላል፡፡ በመጋዘን የሚፈለግው ዕቃ ባይኖር ግዥ እንዲፈጸም ያደርጋል፡፡ ከስቶር ወጪ ሆኖ ለስራ እንዲውል ያደርጋል፡፡ ማንም የፊት መሸፈኛ ጭንብል ሳይለብስ የትኛውም ቢሮ እንደማይገባ እንዲሁ ደግሞ እጅ ሣይታጠቡ ወይንም በሳኒታይዘር እጃቸውን ሳይቀቡ የትኛውም ክፍል መግባት እንደማይቻል፤ እርግጠኛ ነኝ እናንተም ታጠባችሁ ወይንም በሳኒታዘር እጃችሁን ተቀብታችሁ ነው ያለበለዚያ የቢሮ ረዳት ባላስገባችሁ፡፡ አንድ ቀን እኔ ቸኩየ ማስክ ሳላደርግ መጥቼ ጥበቃዎች ያለማስክ አትገባም ህግ ያወጣሔው አንተ ነህ ብለው ከልክሎኝ ወደ መኪና ውስጥ ተመልሼ አድርጌ ገብቼያለሁ፡፡

በመምሪያ ደረጃ ሥራውን የምትመሩበት የራሳችሁ ዕቅድ አላችሁ?

በርግጥ መጀመሪያ አካባቢ ዕቅድ በጽሁፍ አዘጋጅተን ነበር፡፡ አጠቃላይ የዞኑን ዕቅድ እኔና ከኔ ጋር ካሉት ሌሎች የኮሚቴ አባላትና ባልደረቦች ጋር ሆነን ሰርተናል፡፡ እንደፋይናንስ መምሪያ ደግሞ የሴክተራችን ቀን ተቀን የምንተገብረውንም ዕቅድ አዘጋጅተን ነበር፡፡ ዕቅዳችን እጅግ ጠንካራ ነበር ፤ ነግርግን እኛም የህብረተሰቡ ነጸብራቅ እንደመሆናችን መዳከማችን ይታያል፡፡ ይሁን እንጂ እንደሰነድ ለዚህ ዓላማ ማስፈጸሚያ ዕቅድ አዘጋጅተን በራሱ አቃፊ አዘጋጅተን አስቀምጠናል፡፡ እንደዚህ የተዘጋጀ ዕቅድ መኖሩ ድንገት እኔ ቢነሳ እንኳን ሥራው ላይ ምንም ክፍተት ሣይኖር የሚመጣው ሰው በቀላሉ እንዲያስቀጥል ያስችላል፡፡ እስካሁን የተሰራጩት ስርጭቶችን የሚያሳይ ሰነድ ሁሉ ተዘጋጀተዋል፡፡ ነገር ግን ሄደት መጠት ወይንም ያዝ ለቀቅ የማድረግ አዝማሚያ በመከሰቱ ሂደቱ በተወሰነ ደረጃ መዳከምን አሳይተዋል፡፡

እንደመምሪያ የዚህን በሽታ መከላከልና ቁጥጥር ሥራ ሂደት ጠንካራና ደካማ ሁኔታዎችን ተከታትላችሁ የምትገመግሙበት አሰራር ስለመኑር ቢያብራሩ?

አዎን የስራ አፈጻጸም ሂደትን እንገመግማለን፡፡ ይህም በተፈጠረው አደረጃጀት መሠረት ነው፡፡ የሀብት አሰባሰብ ንዑስ ኮሚቴ አለ፡፡ ደግሞ በስሩ የንዑስ ንዑስ ኮሚቴ አለ፡፡ አነዚህም የፋይናንስ የቁሳቁስና በጎ ተሳታፊዎች ኮሚቴዎች ናቸው፡፡ የፋይናንስ ኮሚቴ በገንዘብ ነክ ላይ የማሰባሰብንና የማስተዳደርን ሥራ የሚያከናውን ነው፡፡ የቁሳቁስ ኮሚቴ በበኩሉ ደግሞ እንደማስክ ሳኒታይዘር የምግብና ሌሎች ቁሳቁሶችን የሚያስተዳድር ነው፡፡የበጎ አደራጊዎች ኮሚቴ ደግሞ ወጥቶ የማስተማር ሥራ ይሰራሉ፡- ለምሳሌ የኦርቶዶክስ ጳጳሳት የፕሮቴስታንት እምነት ሰባኪዎች በገበያና ሌሎች ህዝብን ሊያገኙ በሚችሉበት ቦታ ወጥቶ አስተምረዋል፡፡ወጣቶች በተደራጀ አኳሃን ወጥቶ ሌሎችንም በማስተባበር የማስተማር ሥራውን ያከናውናሉ፡፡ ይሁን እንጂ ሥራው እዚህና እዚያ የሚሠራውን ያክል የተደራጀ የግምገማ አካሔድ የለም፡፡ መጀመሪያ አካባቢ በጣም ጠንካራ ነበር፡፡ በየሶስት ቀን በግዴታም እየተሰበሰብን እንገመግም ነበር፤ግን ዕየተለማመደ ሲመጣ ትንሽ ትኩረት ማነስ፡- ይህ ትኩረት ማነስ ስንል በመህበረሰቡ ዘንድ የምናየው የባህሪይ ለውጥ መምጣት ሲገባው /በርግጥ አንድ ጥሩ ነገር የታየው እጅ መታጠብ በሚፈለገው ደረጃ ተከናውነዋል ማለት ይቻላል፡፡ ለምሳሌ አሁን እኔ ቤት ብትሔድ ልጆቼ እኔ ቤት ከመግባቴ በፊት አባዬ እጅህን ታጠብ ይሉኛል፡፡ እነርሱም አስሩን የእጅ መታጠብያ ደረጃዎችን ተከትለው ይታጠባሉ፡፡ አሁን ሆቴሎችጋ ብንሔድ በየደጃፋቸው የእጅ መታጠቢያ ውሓና ሳሙና እናገኛለን፡፡ ይህ በመሰረቱ እጅግ ጥሩ ነው፡፡ ከዚህ ጋር በተያያዘ ምንም እንከዋን የጤና ባለሙያ ባልሆንም የተገነዘብኩት ነገር አለ፡፡ አሁን በቢሮአችን እንደቀድሞ በጉንፋን የሚታመም ሠራተኛ የለም፡፡ አንደኛው ሪቀታችን ጠብቀን መንቀሳቀሳችን ሌላውና ዋንኛው የእጅ መታጠባችን ነው፡፡ ሌካስ እጃችን ነው በርካታ ነገሮችን እየነካካ ለበሽታ የሚያጋልጠን ወደሚል እሳቤ የደረስንበት ሁኔታ አለ፡፡አሰተምሮናል፡፡ ሆኖም ግን የግምገማ ሥርዐታችን የተጠናከረ አይደለም፡፡

ይህንን በሸታ መከላከልና ቁጥጥር ሥራ ለማሳለጥ የተለያዩ ተቋማት በጋራ እየተንቀሳቀሳችሁ እንዳላችሁ ግንዛቤ ይዘናል፤ ነገር ግን ሥራውን በተቀናጀ ሁኔታ ከመምራት አንጻር ምን ይመስላል? የቅንጅት ግኑኝነትስ የት የት ደረጃ ነው የተፈጠረው፡

ቅንጅታዊ ጉዳይን ካነሳን በአስተዳደር በካቢኔ ደረጃ በየጊዜ የሚከናወኑት ሥራዎች ይዳሰሳሉ፡፡ የመከላከሉና ጸጥታ ሥራ በራሱ ንዑስ ኮሚቴ ደረጃ ክትትል ይደረጋል፤ ግን የሀብት ማሰባሰቡ ሥራ በሚመለከት በግል ደረጃ እንዳንዱን አሁን ደውየ ቢጠራችው ይመጣሉ ግን በጋራ ሥራዎችን ተቀናጅተን ከመሥራት አንጻር እንደመጀመሪያው ያለመሆን ይታያል፡፡ ቅድም እንዳነሳሁት መጀመሪያ አካባቢ የተከተልንበት ቅንጅታዊ ሥራ አጅግ በጣም ጥሩ የነበረ ነበር፡፡ ቅንጅታዊ ሥራን የሚያስረዱ በጋራ የምንገመግምበት ቃለ-ጉባዔ አለን፡፡ ይሁን እንጂ በታችኛውም መዋቅር ማለትም ወረዳዎችና ከተማ አስተዳደሮች አካባቢም እንደዘመቻ ሥራ አንዴ ቦግ ብለው የመዳፈን ዓይነት አዝማሚያ ይታያል፡፡ ለዚህ መቀዛቀዝ ልበለው መርካት አይነት ሁኔታ የዳረገን አንድ ጊዜ የጤና ሚኒስቴሯ ሪፖርት ሲያደርጉ ዛሬ በበሽታ የተያዘ የለም ባሉበት ቀን ሰው በቃ ፌሽታ በፌሽታ ሆነው የሚበላ በልቶ የሚጠጣ ጠጥቶ በቃ ከዚህ በኋላ በሽታ ኢትዮጵያ ውስጥ የለም በማለት መቦሰት ተጀመረ፤የሌላውን አካባቢ ሁኔታ ባላውቅም እንደኛ ዞን መጥፎ አጋጣሚ ብለን የወሰድንበት ነው፡፡ የሌላውን አካባቢ እስቲ ገና እናጣራለን፡፡ ሚኒስቴሯ በሁኔታው ተስፋ ሰጪነት ያሳዩትን አዎንታዊ ሁኔታ በሽታው እንደለሌ መቁጠሩ በተቀጣጠለው እንቅስቃሴ ውሀ እንደመቸለስ ሆኖብናል፡፡ ይልቁንም በሽታው በሀገራችን የለም ይህ አመራሩ ያመጣው ጣጣ ነው እስከሚባል ተደረሰ፡፡

እስካሁን የተከናወኑ ተግባራት ወደታለመለት ግብ ሊያደርሱ ስለመቻል አለመቻል የተረዳችሁ አግባብ ካለ?

አንድ በጣም የሚገርም ነገር አጋጥሞኛል እኔን፡፡ አንዴ ናሙና ይዞ አድሳባና ሀዋሳ የሚሄዱት ባለሙያዎች ከተፈቀደው አበል በላይ እንዲከፍላቸው ጫና ለመፍጠር የሞከሩበት ሁኔታ ነበር፡፡ ምን ስለአሰራር ታወራለህ? እኛ በሞት መካከል እየሄድን አንተ ደግሞ ስለ ተመን ታነሳለህ ዝምብለህ አትከፍልም? ለምን ትቆጣጣራለህ እኛን እኛኮ ኮሮና ቫይረሱን ከነነፍሱ ተሸክመን ነው የምንሄደው እኛ በነፍሳችን ተወራርደን ለህዝብ ብለን ስንሰራ አንተ ስለገንዘብና አሰራር ተጨነቃለህ? ህዝብ ለኛ ብሎ የሰበሰበውን ገንዘብ ዝምብለህ አትሰጠንም ዓይነት ፍላጎት አሳይተዋል፡፡ እኛ ቆጥበን ስንሰጥ ያንን ያለመቀበልና ያለመርካት አለ፤፤ በርግጥ ጤና መምሪያው ባግባቡ ጥያቄ ያቀርባል፤ እኛም በዚያው አግባብ እናስተናግዳለን ግን ባለሙያዎች ያለመርካትና ጫና ለመፍጠር የማሰብ አዝማሚያ ይታያል፡፡ ይህ ከአስራር ውጭ የሚቀርበው ፍላጎት እንዲታረም በተለያዩ ጊዜያት ገምግመናል፡፡ ከዚያ ውጭ በጣም የሚገርም ነገር ያጋጠመን ወጣቶች መጥተው ይህን የተሰበሰበ ገንዘብ ለኛ ስጡን ኮሮና የሚባል ነገር የለም ብሎ ከዚህ ከተማ ጭምር የመጡበት ሁኔታ እጅግ አስደንግጦኛል፡፡ ይህ ማለት ይህ ሀብት ምን ያህል የህዝብ ሀብት እንደሆነና ህዝቡ በምን ዓይነት ቁረኛ እንደሚከታተል እና ትንሽ ብክነት የሚመስል ነገር ቢታይ በኔና በዋናው አስተዳዳሪ ላይ ምን ዓይነት ነገር ሊከሰት እንደሚችል ማሳያ ነው፡፡ እንደዚህ ዓይነት ነገር እንዳይከሰት በላይ በተቀመጠው አግባብ ወጥሬ ሲሰራ ነበር፡፡ ለዚህም ነው ጥብቅ አሠራር የምንከተለው፡፡ አንድ ጥንድ ማስክ እንኳን ያለ ህጋዊ አሰራር ሊወጣ አይችልም፡፡ ማንኛውም የንብረት ጥያቄ መስመሩን ተከትሎ ተጠይቆ በመጨረሻ እኔ ሳጸድቅ ነው ወጪ የሚደረገው፡፡ አሁን የንብረት ቆጠራ እያስደረግኩ ነው፡፡ ምናልባት ያለእኔ እውቅና ወጪ ተደርጎ እንደሆነ ለማረጋገጥ ያለዚህ አሰረር ከወጣ የንብረት ክፍል ሰራተኞች ተጠያቂ ይሆናሉ፡፡ ይህን አሰራር መከተል ምን ይታወቃል ድንገት በመሞዳሞድ ለጓደኛ ዘመድ አውጥቶ ብሰጥም ተጠያቂ ማድረግ ይቻላል፡፡ አንዳንድ ሰዎች እኛ በመምሪያ ደረጃ ስለምንከለክል አልፎ ሄደው የማስጨነቅ ሁኔታ አለ፡፡ ይሁን እንጂ በጉድለት ደረጃ የከፋ ነገር የለም፡፡ ምክንያቱም አስቀድሞ ጠንካራ አሠራር ሥርዓት ስለዘረጋን ነው፡፡

የኮሮና በሽታ እንደበሽታ ከፍተኛ ና ዘርፈ-ብዙ ችግሮችን ያጋፈጠን እንደሆነ እንረዳለን፡፡ ሆኖም ግን ለበሽታ መከላከልና ቁጥጥር በሚደረገው ሂደት የተገኘ መልካም ተሞክሮ ካለ ቢገለጽ

እጅግ በጣም ሊጠቀሱ የሚችሉትን መልካካሞች አጋጣሚዎች አሉ፡፡ በተለይ ለማሕበራት ሰፊ የገቢ ምንጭ ሆነዋል፡፡ ማስክ ለምሳሌ እኛጋ ነው የሚዘጋጀው፡፡ መጀመሪያ እንደፕሮቶታይፕ የቴክኒክና ሙያ ኮሌጅ ሰርተው ለጥቃቅንና አነስተኛ ማህበራት አስተላለፉት፡፡ ከንክኪ ወጭ የሆነ የእጅ መታጠቢያ ማሽንም አዘጋጀተው ሲያቀርቡ እኛ አንድ ሃያ የሚሆን ገዝተን ለተቋማት አሠራጨን፡፡ ወረዳዎች ደግሞ ይህንን አይተው በራሳቸው ደረጃ ባሉት ተቋማት እያስመረቱ መጠቀም ጀምረዋል፡፡ ሌላው ደግሞ የማስክ ገበያና ምርት ሁኔታ መጧጧፉ ነው፡፡ የአልባሳት ማዘጋጃ ሱቆች በሙሉ ወደማሰክ ሥራና ሽ ያጭ መዛወሩ ሌላው አጋጣሚ ነው፡፡ ሌላው ደግሞ የሳኒታይዘር ምርት ነው. ለምሳሌ እይ ይሕ የአርባምንጭ መምህራን ትምህርት ኮሌጅ የሰራው ሳኒታይዘር ነው፡፡ የአርባምንጭ ዩኒርሲቲ ሆስፒታል አንድ የግል ባለሀብት ጭምር በሳኒታይዘር ምርትና ሽያጭ የገቡበት ሁኔታ አለ፡፡ እንግዲህ ከኮሮና ጋር በርካታ የቴክኖሎጂ አጠቃቀምና ተነሳሽነት የጨመረበት አግባብ አለ፡፡ በርካታ በጎ ጎኖች ማየት ይቻላል፡፡ ማህበራዊ ርቀት ቢባልም ሰዎች እንዲተሳሰቡ ማድረግንም ማንሳት ይቻላል፡፡ ማንም ቢሆን ባለሀብትም ቢሆን ዋስትና ስለሌለ መተሳሰብ እንደአማራጭ ታይተዋል፡፡ ሌላው ያልጠቀስኩት የግል መከላከያ አልባሳትን የቴክኒክና ሙያ ኮሌጆች ያመረቱበት ሁኔታ ሌላው ነው፡፡ ሌላው የኢኦሲ ማዕከል ገብተው የሚወጡት በሰንሰረ በሚሰራ ሙሉ አካላችውን ታጥበው የሚወጡበት ይገኛል፡፡ ይህም አስቀድሞ በትላልቅ አለምአቀፍ ተቋማት አይነት ብቻ የሚገኝ ነበር፡፡ ትላልቅ ሆቴሎች እንደነሀይሌ ሪዞርትና ፓራዳይዝ ሆቴሎች ነበር፡፡ አሁን ግን በእየንዳንዱ ተቋምና የህክምና ማዕከላት እንዲኖር መደረጉ የሚታይ የፈጠራ ስራ ነው፡፡ ምናልባት እናንተም አይታችሁ ልሆን ይችላል እዚህ ለምለም ሆቴል ከሄዳችሁ በእጅ ሳትነኩ እጃችሁን ታጥባችው መውጣት ትችላላችሁ፡፡ ብዙ ነገራችን ላይ ለውጥ እያመጣ ነው፡፡ ወረርሽኙ ባጭር ጊዜ በቁጥጥር ሥር ካልዋለ የማህበራዊ አኗኗር ዘይቤአችንም ጭምር ሊቀይር ይችላል፡፡

የአኗኗር ዘይቤያችን በምን ሁኔታ ሊቀይር ይችላል? መገለጫውስ ምን ሊሆን ይችላል?

ለምሳሌ እኛ ብዙ ባህላዊና ሀይማኖታዊ ልማዶች ያሉን ህዝቦች ነን፡፡ የቀብር ሥርዓት የሰርግ ሥርዓት የሰላምታ አሰጣጥ ሥርዓታችን ቀድሞ እንደነበረ ላይቀጥል ይችላል፡፡ ከዚህ በፊት በጉንጭ የሚደረግ መሳሳምና የእጅ መጨባበጥ እየቀረ ያለበት ሁኔታ አለ፡፡ በሃይማኖታዊ ቦታዎች ከዚህ በፊት የሚደረጉ ልማዶች መፈጸም ያለመቻል፡- እንደኦርቶዶክስ እምነት ሥርዐት መስቀል መሳም ባሁኑ ጊዜ ያለመቻል፡ ምዕመኑ ሄደው ዝም ብሎ መቆም የመሳሰሉት እና በርካታ ነገሮችን እየቀየረ ነው፡፡ በሀይማኖቶች አካባቢ ፈተና እየፈጠረ መሆኑ፡ ከዚህ በፊት የነበረወረ የአብሮነት መገለጫዎች እየቀሩ መሆኑ፤ ከአምልኮ ቦታ ሲወጡ እርስ በርስ ተቃቅፈው ሰላምታ መሰጣጣት እየቀረ ነው፡፡ እኔ አሮጊት እናት አለችኝ ግን ወደገጠር ሄጄ ለርሷ ሲል እጄን ሰጥች ሳልሳለም እመለሳለሁ፡፡ ይሔ ከባድ ነው ግን ይረዱታል ፡ በሌላ አቅጣጫ ደግሞ ኮሮና እኛን አይዝም የሚሉ ይገኛሉ፡፡ አንድ ቤተእምነት ለቅሶ ወላይታ ሄደን ለቀስተኛው የሚያሳየው አኳኋን ስለበሽታው ምንም እንደልሰሙ ነው የሚሆኑት፡፡ አንዲት እህቴ የዚያ እምነት ተከታይ ናትና ስለኮሮና ሳናሳባት እኔን ወደመገሰጽ ትደርሳለች፡፡ ጭራሽ እምነት እንደሌለን አድርጎ ማየት ነበር፡፡ ሆኖም ግን መታወቅ የሚገባው እምነት ሌላ ነው ኮሮና ደግሞ ሌላ ነው፡፡

ቅንጅታዊ አሰራርን ከማዳበርና ማጠናቀር አንጻር ያበረከተው መልካም አጋጣሚ ካለስ?

በቀጣይ በጋራ ለመስራት ምቹ ሁኔታ ከመፍጠርም አንጻር

ማናኛውም ነገር በጎና መጥፎ ጎኖች እንደሚኖሩት ሁሉ ኮሮናም እንደዚሁ፤ ለምሳሌ ጀርሞች ጎጂና ጠቃሚ እንደሆኑ ይነገራል፤ ኮሮናም እንደዚሁ፡፡ አንዱ በጎ ጎኑ ሰው ብርቱ ሰራተኛ እንዲሆን እያደረገ መሆኑ፤ እጅግ በጣም ጥሩ የሆነ ነገር ከዚህ በፊት እኔ ምን አገባኝ ብሎ ለነገሮች ዳተኛ መሆንን በመቀየር አንዱ ለመኖር የሌላው ደህንነት አስፈላጊነት ወሳኝነትን እንዲረዳ አድርገዋል፡፡ በሸታ መከላከል ስርዓትን የሚጥስ ካለ በጋራ የመገሰጽ ና የመሳሰሉት ልምድ መዳበር አብሮ የመቆምን ስርዐት ያጠናክራል፡፡ከዚሁ ሌላ የመረጃ ፍሰትና ህብረተሰቡ ለመረጃ ያለው ተነሳሸነት መጨመሩ ነው፡፡ለምሳሌ ዳራማሎ በሚባል ወረዳ አንድ ከአዲስ አበባ የመጣች ሴት በበሽታው ተይዛለች የሚል መረጃ ሲደርስ አርሶአደር ራሱ ተሯሩጦና ጮሖ ከመጠቆም ይሄዳል፡፡ ከአዲስ አበባ ከሀዋሳ ከአርባምንጭ ሰው ወደሰፈር ገባ ከተባለ ልጁም ቢሆን ተሯሩጦ ወደለይቶ ማቆያ እንዲገባ ያደርጋሉ፡፡ እንግዲህ በሽታው በሀ/ሰቡ ውስጥ የፈጠረው ስጋት ከፍተኛ ነው፡፡ ይዞ ይሄዳል የሚል ስሜት ነው፡፡ ግንዛቤ አለ ግን….. ኮቪድ-19 ቫይረሱ የሚተላለፍበትን መንገድ አውቆ በመጠንቀቅ ረገድ ህዝብ በብዛት በሚሰበሰብባቸው እንደገበያና ሆቴሎች አካባቢ የጥንቃቄ ጉድለት በስፋት ይታያል፡፡

የቁልፍ መረጃ ምንጭ ስድስት እርሻና ተፈጥሮ ሀብት መምሪያ - ስድስት

1. እንደእርሻና ተፈጥሮ ሀብት ልማት መምሪያ በዞኑ የኮቮድ-19 በሽታ ከመከላከልና ቁጥጥር ሥራ አንጻር ያለው ቁርኝት ምን ይመስላል?

- ከተለያዩ መዋቅሮች ጋር ወደላይና ታች እንዲሁም ወደጎንዮሽ ያለው የሥራ ግኑኝነት
- ተቋማዊ አደረጃጀትን ለምላሹ ብቁ ከማድረግ አንጻር

እንደእኛ ጋሞ ዞን ይህ ኮቪድ-19 ወረርሽኝ ከታወቀ ወዲህ ከክልል መጀመሪያ ለኛ መነሻ የሚሆን እስከ ታችኛው እስከ ቀበሌ መዋቅር ድረስ የግብርና ሥራ ሣይቆም በሽታን መከላከል እንዲቻል ቀጣይም ከዚህ ጋር ተያይዞ የሚነሱትን ስጋቶች እንደት መቀነስ ይቻላል በሚል ሥርዓት የመዘርጋት ሥራ ነው የሰራነው፡፡ስለዚህ መጀመሪያ በዞን ደረጃ የተለያዩ ሴክተር መስሪያ ቤቶች በእኔ የሚመሩት እንስሳትና ዓሳ ሀብት ልማት መምሪያ የእርሻና ተፈጥሮ ሀብት ሥር ያሉት አምስት ዘርፎች(የግብዐት ዘርፍ የእርሻ ዘርፍ የተፈጥሮ ሀብት ዘርፍ አደጋ ስጋት ዘርፍ የህብረት ሥራ ዘርፍ) የአካባቢ ጥበቃና ደን ሀብት ጽ/ቤት እና የመሬት አስተዳደር በጋራ የሆነ አደረጃጀት ተፈጠሮ እንዲንቀሳቀስ ከክልል በተቀመጠ አቅጣጫ ነው፡፡ መጀመሪያ ከነርሱ ጋር በመሆን ምን ሊደረግ ይችላል የሚል እሳቤ ይዘን ነው፡፡ ታችኛውስ አካል ምን ማድረግ አለበት የሚለውን የያዘ መነሻ ሰነድ ከክልል ገጠር ልማት ማስተባበርሪያና እርሻና ተፈጥሮ ሀብተ ልማት ቢሮ በኩል በ ኢ-ሜይል ለኔ ተላከልኝ፡፡ ያንን መነሻ አድርገን በመጀመሪያ እኛ ልንረዳ ስለምገባን ውይይት አደረግን፡፡ ሊያመጣ በሚችለው ጫና ላይ መጀመሪያ እኛ ተረድተን ካልተንቀሳቀስን ህ/ሰቡን ማናቀሳቀስ ስለማይቻል ህዝብ ውስጥ ውዥንብርም እንዳይገባ ለመከላከልም ጭምር፤ ከዚያ ጋር ተያይዞ የልማት ሥራ እዚያው ተተክሎ እንዳይሰራ ተነቅሎ ሊወጣም የሚችልበት ሁኔታ እንዳይፈጠር፤ በወረዳ ደረጃ ያሉት ባለሙያች በሽታው በንክኪ ጭምር የሚተላለፍ እንደመሆኑ እኛ በቂ ግንዛቤ ፈጥረን ወርደን ካላሰዬን በውዥንብር ተረጋግቶ የግብርና ልማት ሥራን መስራት ላይችሉ ይችላሉ ብለን ነው የተንቀሳቀስነው፡፡ በዚሁ ሂደት የኛ ሀላፊነትና ተጠያቂነት ምንድነው የታችኛውስ መዋቅር ሃላፊነትና ተጠያቂነት ምን መሆን አለበት የሚለውን አስቀድመን አዘጋጅተንና በዞን ደረጃ በጋራ ተወያይተን ነው ወደትግበራ የገባነው፡፡ የግብርና ሥራ ይህንን ወረርሽኝ ከመከላከልና ቁጥጥር ሥራ ጋር እንዴት ተቀናጅተው ሊቀጥል እንደሚችል ተወያይተናል፡፡ ሥራው እንዴት ሳይንጠባጠብ ሊቀጥል ይችላል ከሚለው በላይ ግንዛቤ ፈጥረን ነው ዕቅድ ያዘጋጀነው፡፡ ይህ ዕቅድ የግብርናውን ዘርፍ ሥራ ከኮቪድ-19 በሽታ መከላከልና ቁጥጥር ጋር አቀናጅትን ከማስቀጠል ታስቦ የተዘጋጀ ነው፡፡ በዚህ ዕቅድ የተካተቱት ጉዳዮች የንቅናቄ ሥራ በምን አግባብ ሊመራ እንደሚገባ የበሽታ መከላከል ሥራ ግንዛቤ ማስጨበጥን ጨምሮ በምን አቅምና እውቀት መተግበር እንዳለበት ታስቦ ነው የተዘጋጀው፡፡ ተግባሩ እንዳይንጠባጠብ ይህ የአመራርና የህዝብ ንቅናቄ ሥራ የተሠራው፡፡ ይህ የግንዛቤ ማስጨበጥ ሥራ በከፍተኛ የድምጽ መሳሪያ/ ሞንታሪቦ እየታገዘ ተሰርተዋል፡፡ የእርሻ ሥራ በሚያከናውኑበት ወቅት እንዴት መሳሪያውን ከቫይረስ ነጻ አድርገው መጠቀም እንዳለባቸው፤ አካላዊ ሪቀታቸውን በምን አግባብ መጠበቅ እናዳለባቸው ሙሉ መረጃ ያካተተ ቅስቀሳና ትምህርት በሁሉም ወረዳዎች ህዝብ በሥፋት በሚገኝባችው አካባቢዎች ተከናውነዋል፡፡ አርሶአደርን ታሳቢ ያደረገ የግንዛቤ ማስጨበጫ ሥራ በሶስት ዙር ተካሂዷል፤ በመጀመሪያ ዙር አምሰት ቀን የፈጀ ቅስቀሳ አድርገን ሂደቱን ገምግመን ካየን በኋላ እንደገና በተደራጀ መልክ ግንዛቤ ማስጨበጫና ቅስቀሳ ሥራ ያከናወንነው፡፡ተመሳሳ አካህድና አደረጃጀት በታችኛው መዋቅር እንዲፈጠር አድርገናል፡፡ ወረዳው በበኩል ልክ እኛ ከዞን ወርደን እነርሱን እንደደገፍን ቀበሌ ወርደው ተገቢ ቅስቀሳና የግንዛቤ ማስጨበጫ ስራ እንዲሰሩ ተደርገዋል፡፡ይህ የባለሙያና አመራር ቡድን መደራጀቱንና ወርደው መስራታቸውን ተመልሰን ያየንበት ሁኔታ ነበር፡፡በዚህ ሂደት እያንዳንዱ የግብርና ሥራ በሚከናወንበት ጊዜ ተሳታፊው ራሱን ከበሽታው መከላከል መቻሉን የማረጋገጥ ስራ እንሰራለን፡፡ ለምሳሌ አሁን የተጀመረው የአረንጓዴ አሻራ ትግኝ ተከላ ሥራ ላይ የሚሳተፉት አካላዊ ሪቀታቸውን መጠበቃቸውንና መከላከያ ቁሳቁስ መጠቀማቸውን እንከታተላለን፡፡ እኛጋ ኮቪድ ከመጣ ጀምሮ ዝናብም ስላልተቋረጠ ሁለቱንም ሥራዎች ጎን ለጎን እያስከሄድን ነው፡፡ ጠቅለል ባለ መልኩ መጀመሪያ እንዴት ግንዛቤ እንፍጠር ቀጥሎም ደግሞ የትግበራ ክትትልና ግምገማ ሂደት የሚሳለጥበትን አደረጃጀት እንዴት እንፍጠር ብለን ተንቀሳቅሰናል፡፡ይህም በሁሉም ሴክተሮችና ባንድ ሴክተር ደግሞ እስከታችኛው መዋቅር ያለውን በማካተት ነው፡፡

ቅንጅታዊ ስራን በዞን ደረጃ ካነሳን አይቀር የእርሻና ተፈጥሮ ሀብት ልማት መምሪያ ከዋናው ግብረ-ሀይል ጋር ያለው ቁርኝትና ቅንጅት ምን ይመስላል?

የኛ ሃላፊነት ሁለት ነው፡- 1) የግብርና ሥራ ሣይስተጓጉል እንዲሰራ ማድረግና 2) በሥራችን ባለው የአደጋ ስጋት ሥራ አመራር በኩል ለኮቪድ በሽታ የሚሆን ሀብት ማሰባሰብና ማሰራጨት ድርሻ ነው፡፡ የአደጋ ስጋት ሥራ አመራር ጽ/ቤት በሀብት ማሰባሰብ ንዑስ ኮሚቴ ጸሐፊ መሆኑ ተቋማችን በዋናው አስተዳዳሪ በሚመራው ዐቢይ ኮሚቴ/ ግብረ-ሀይል በሁለትና ከዚያ በላይ ተሳትፈናል፡፡ ዐቢይ ግብረ-ሀይል ከ9-11 የሚሆኑ ንዑሳን ኮሚቴዎች ያለው አደረጃጀት ነው፡፡ ሌላይኛው ኮሚቴ የገጠር ልማት ኮሚቴ ነው፡፡ በዚሁ ንዑስ ኮሚቴ የኛ ተቋም ሰብሳቢ ነው፡፡ የሀብት አሰባሰቢ ሥራ በመጀመሪያ አካባቢ በየሶሰት ቀናት እየተገናኘን የሀብት አሰባሰብ ሂደት ምን ይመስላል? ችግሩ ቢፈጠር ተጋላጮች የሚበሉት ና ሌሎችንም ግብዐት ማሟላት ስለመቻል እንገመግም ነበር፤ አሁን ነው ትንሽ መቀዛቀዝ ያሳየው፡፡ ሌሎች የሚፎካከሩ ክስተቶች በመፈጠሩ ምክንያት ወደሳምንት እንዲያውም አንዳንዴ በ15 ቀናትም ያለመሰብሰብ ተከስተዋል፡፡

አስቀድመው እንደገለጹልን የራሳችሁ ዘርፍ እቅድ አዘጋጅታችዉ እንደምትንቀሳቀሱ ተገጸዋል፤፤ ለዚህ መነሻው ምንድነው?

ከኮቪድ ጋር የተያያዘ ዕቅድ እንዲናዘጋጅ መነሻ ከሆኑት ውስጥ አንዱ የፋኦ ያስቀመጠው ሪፖርት ነው፡፡ በዚህ ሪፖርት የተጠቆመው ኢትዮጵያ ከኮቪድ ወረርሽኝ በአንበጣ መንጋ በመጠቃት ተባይና አረም ሌሎች ተፈጥሮና ሰው ሠራሽ አደጋዎች በቀጣይ ከፍተኛ የምግብ ዕጥረት ሊያጋጥማት እንደሚችል ተንብየዋል፡፡ እንደሀገር 15 ሚሊየን እንደ ክልላችን 2.5 ሚሊየን እና እንደዞናችን የጎርፍና ናዳ እንዲሁም የአንበጣ መንጋ ወረርሽኝ ክስተት በአስር ወረዳዎች የተከሰተ መሆኑና አሁን ደግሞ ኮቪድ-19 ሰዎች ወደምርት ሥራ እንዳይንቀሳቀስ የሚያደርግ እንደመሆኑ የምርት ማነስ አጋጥሞ ዞናችን ለከፋ የምግብ እጥረት ሊያጋጥም ስለሚችል ይህንን እንደት መቋቋም እንችላለን በሚል ነው ዕቅዱን ያዘጋጀነው፡፡ ከችግሩ ሊያላቅቁን የሚችሉትን አማራጮች መጠቀም እንድንችል ክልሉም በመጀምሪያ አካባቢ በስልክ ከዚያም ቀጥሎ በቪዲዮ ኮንፈራንስና ባሁኑ ጊዜ ደግሞ በዙም እየተገናኘን ሃሳብ በመለዋዎጥና ከነርሱ የተገኘውን አቅጣጫ መነሻ ወደኛ ነባራዊ ሁኔታ ቀይረን ነው ዕቅዱን ያዘጋጀውን፡፡ እንደኛ ዞን ከላይ እንደተጠቀሰው የናዳ ጎርፍና አንበጣ መንጋ ከበሽታው ጋር ታክሎ ከሌላ አካባቢ በተለየ ሁኔታ እንደሚያጠቃን ስላመንን በተለየ ሁኔታ የማካካሻ ዕቅድ ያስፈልገናል ብለን ነው የተነሳነው፡፡ መነሻዎቹ ሁኔታዎች የኛ ነባራዊ ሁኔታና ከላይ የተላከልንን በማጣመር ነው፡፡

የኮቪድ -19 በሽታ መከላከልና ቁጥጥር ሥራ በተቋማችሁ ደረጃ እንዲያስተባብር ተለይቶ የተመደበ ባለሙያ/ሰው ስለመኖሩ፡-

እኛ የቴክኒክ ኮሚቴ አለን እንዲሁም የአመራር ኮሚቴ ቡድን አለን፡፡ የአመራር ቡድን እኔ ነኝ የሚመራው፡፡ የቴክኒክ ቡድንን የእርሻ ቡድን ዳይሬክተር ይመራል፡፡ አመራሩ አመራሩን ወርደው እንደሚደግፍ ሁሉ ባለሙያውም ወርደው ባለሙያ መደገፍ ስላለበት ሁለቱም አደረጃጀቶች አስፈልገዋል፡፡በዚህ አደረጃጀት ታግዘን ታች ያሉት አርሶአደርና ቤተሰቡ እንዲሁም የልማት ሠራተኛው ከበሽታ ራሳቸውን ጠብቀው የግብርና ልማት ሥራ እንዲሰሩ የማድረግ ሥራ ለማሳለጥ ነው፡፡ በሽታው በዚህ ጊዜ ይወጣል ወይንም በዚህ ጊዜ የመከላከያ ክትባት ወይንም የፈውስ መድሃኒት ተገኝተው እፎይ እንላለን የምንልበት ጊዜ ስለማይታወቅ በመከላከሉ ሥራ ጠንክረን መስራት አለብን ብለን ነው በሁለት ኮሚቴ ተዋቅረን እየተንቀሳቀስን የምንገኘው፡፡ በዚሁ አግባብ ለልማት ሠራተኞች ራሳቸውን የሚከላከሉበት የአፍና አፍንጫ መሸፈኛ ጭንብል እጅ መታጣቢያ ሳሙናና ውሀ እንዲሁም ሳኒታይዘር በሁሉም ወረዳዎች ላሉት አድለናል፡፡ የተለያዩ መንግስታዊ ያልሆኑት ተቋማት ድጋፍ እንዲያደርጉ ጥያቄ አቅርበን እየተጠባበቅን እንገኛለን፡፡ ከነዚህና ሌሎች ተቋማትም ድጋፍ እንዳገኘን ወደታች የምናደርስ ይሆናል፡፡

የዕቅድ ክንውን ሂደት የምትከታተሉበት አግባብ እንዴት ነው?

ቅድም እንዳልኩት አመራሩም ሆነ ባለሙያ እስከታች ወረዳና ቀበሌ ድረስ ወርደው ድጋፍና ክትትል ያደርጋል፡፡ወርደው ሱፐርባይዝ ካደረገ በሓላ እዚያው ግብረመልስ ይሰጣል፡፡ከዚያ ተመልስው እዚህ ዞን ደረጃ በጋራ አይተን እያንዳንዱን ወረዳ ደረጃ አውጥተን ጥንካሬውንና ሁድለት ለይተን በኔ ፍርማ በተደራጀና መደበኛ አሠራርን በተከተለ መልኩ ግብረመልስ ይላክላቸዋል፡፡ በዚህም የምናካትተው የዝግጅትና ትግበራ ምዕራፍ በማሳ ዝግጅትና የዘር ሽፋን የቴክኖሎጂ አጠቃቀም እንገመግማለን፡፡ ለምሳሌ ዛሬ አብዛኛው አመራርና ባለሙያ ለድጋፍ መስክ ወጥተዋል፡፡

እስካሁን በተከናወነው የድጋፍና ክትትል ሥራ የተገኙት ክፍተቶች ካሉ?

አንደኛ ባደርግናቸው ድጋፍና ክትትል ሂደት እንደጉድለት ያገኘናቸው በቀበሌ ደረጃ ነው፡፡ አንዳድ ልማት ጣቢያዎች ራሳቸውን ከሥራ ነጥለው መቆየታቸውን ተገንዝበናል፡፡ይህም በምንም ሁኔታ ተቀባይነት የሌለው መሆኑን አስረድተን ይልቁንም የግብርና ልማት ሠራተኛ ልክ እንደጤና ባለሙያ በዚህ ሂደት ላይ ግንባር ቀደም በመሆን ሊያጋጥመን ከሚችለው አደጋ ዞናችንና ህዝባችን መታደግ አለብን ብለው መንቀሳቀስ እንዳለባቸው አቅጣጫ ሰጥተናል፡፡ በልማት ጣቢያ በተፈጠርው ክፍተት መነሻነት አንዳንድ አካባቢዎች የግብርና ቴክኖሎጂ አለመጠቀም ታይተዋል፡፡ ይህንን ክፍተት ከለየን በኋላ አደገኛነቱን ግምግመን ታችኛውን የኛን መዋቅር በራሳችን ደረጃ ጠርተን ግልጽ አቅጣጫ ካስቀመጥን በኋላ በየደረጃው ያሉት የፖለቲካና መንግስት አመራሮች ሴክተሩን መደገፍ ስላለባችው በዞኑ ዋና አስተዳዳሪና በፓርቲው ከፍተኛ አመራር በኩል አመራር አንዲሰጥ ተደርገዋል፡፡

እንደመልካም አጋጣም ልናነሳው የምንችለው በዚህ ዓመት የዝናብ ስርጭት የተሻለ በመሆኑ ምርት ይቀንሳል የሚል ሥጋት የለንም፤ በተወሰነ ደረጃ በጎርፍና በሀይቅ ሙለት ምክንያት የተወሰኑትን ማሳዎችን ብናጣም፡፡የቴክኖሎጂ አጠቃቀም ላይ ውሱኑነት ቢኖርም በማሳ ሽፋን ረግድ ካቀድነው በላይ ሸፍነናል፡፡

በሽታውን ከመከላከል አንጻር እጅን የመታጠብ ልምድ እሰዮ የሚያሰኝ ቢሆንም አካላዊ ሪቀትን በመጠበቅ ረገድ ገና ብዙ ይቀራል፤ አሁንም ተሰብስበው መቆም ስላለ ይህንን ለመሻገር በቀጣይ መስራ ይገባናል፡፡ ከዚህ በፊት በሽታው እኛጋ አልገባም ብለው ሲዘናጉ አሁን በዞናችን መከሰቱን አረጋግጠናል፡፡ እኛ አካባቢ ለየት የሚያደርገው አብዘኛው ደጋ አካባቢ የሸማ ሥራ ልምድ ስላላቸው የተሻለ ገቢ ለማግኘት አዲስ አበባ ሄደው ይሰሩ ነበር፤ አሁን የሸማ ገበያ ከበሽታው መከሰት ጋር ተያይዘው ስለተቀዛቀዘ ወደዚህ እየተመለሱ ነው፡፡ ይህም ለዞኑ ከፍተኛ ሥጋት ነው፡፡ በመሆኑም ይህንን ተገንዝበን የመከላከሉን ሥራ አጠናክረን መቀጠል ያስፈልጋል፡፡ አስቀድሞ በሽታው አልተከሰተም በሚል መዘናጋት እንደነበር ገምግመናል፡፡

ቅንጅታዊ አሠራርን በሚመለከት ያለው አደረጃጀትና አተገባበር እንዴት ነው?

እኛጋ አሁን ቅንጅቱ የተሻለ ነው፡፡ ቅንጅታዊ ሥራ ከተለማመድንባቸው ጉዳዮች አንዱ አካባቢያችን በጎርፍና በናዳ በተጎዳበት ጊዜ ምላሽ ለመስጠት አደረጃትን ቀድመን ነው ያመቻቸነው፡፡ ይህንን አደረጃጀት ለኮቪድ-19 ምላሽም ተግባራዊ እያደረግን ነው፡፡ ለዚህም መገለጫ የሚሆነው ችግሩ ለሀገራችን ስጋት መሆኑ ከተረጋገጠ ጊዜ ጀምሮ ከላይ ፌዴራልና ክልል ሳንጠብቅ የተጉዱትን የህብረተሰብ ክፍሎችን ለማገዝ መንቀሳቀሳችን ነው፡፡ ችግሩን በመገንዘብ በየደረጃው የሚገኘውን ህዝብን በማነቃነቅ ገቢን በማሰባሰብ ተጎጂዎችን ያገዝንበት ሁኔታ ነው ያለው፡፡ በአካባቢ የሚገኙ መንግስታዊና መንግስታዊ ያልሆኑ ተቋማት እንደአርባምንጭ ዩኒቨርስቲ መምህራን ትምህርት ኮሌጅ ጤና ሳይንስ ኮሌጅ የግል ባለሀብቶች እሰከአዲስ አበባ ድረስ ያሉት ሁሉ በድጋፉ እንዲሳተፉ ተደርገዋል፡፡ ቀድሞ ያደራጀነውን ለዚህ በሽታ ስለተጠቀምን ቅንጅታጅን የተሻለ ነው ማለት ይቻላል፡፡

የኮቪድ-19 ወረርሽኝ ለመከላከል በምታደርጉት ንቅናቄ እንደመልካም አጋጣሚ ወይንም አዎንታዊ ጎን ሊሆን የሚችል ልምድ ያገኛችሁበት አጋጣሚ ካለ ቢያብራሩ?

ያው አንድ ነገር ስመጣ በርካታ በአዎንታዊም ሆነ በአሉታዊ የሚገለጹትን ጎኖች ይዞ ይመጣል፡፡ ኮቪድ-19 በሽታም አሉታዊ ጎኑ እጅግ የገዘፈ ቢሆንም የተወሰኑት በአዎንታዊ ማየት የምንችለው ጎን አለው፡፡ በመጀምሪያ እንደግብርና ዘርፍ በአሉታዊ ጎን ያየነው የሙዝ ገበያ መውደቁ ነበር፡፡ ሙዝን የሚያነሳ ያጣበት ሁኔታ ነበር፡፡ ይህም አርሲአደሩ ወደእርሻ ሥራ ሀይሉን አጠናክረው እንዲገባ ማድረግ ችለዋል፡፡ በሌላ ጎኑ ደግሞ የሌሎች አትክልቶችና እህል ገበያ ወዲያው የናረበት ሁኔታ ታይተዋል፡፡ የበቆሎ ገበያ ተቀዛቅዞ የት ይሸጣል ብለን ሰግተን ነበር፡፡ በአራት ወረዳዎች (ከምባ ዳራማሎ ቁጫ ቦሮዳ )ብቻ 82 ሺህ ኩንታል ከኮታ በላይ የተመረተ በቆሎ መውሰጃ አጥተን ነበር፡፡ የኮቪድ ወረርሽን ሲፈጠር ወዲያውኑ የበቆሎ ፍላት በመጨመሩ የተሻለ ገበያ ተፈጥረዋል፡፡ ስድስት መቶ ሓምሳ ብር ይሸጥ የነበርው በቆሎ በአንድ ጊዜ አንድ ሺ ሶስት መቶ ብር የገባበት ሁኔታ ነበር፡፡

አርሶአደሩ የተሻለ እንዲያመርት መልካም ማበረታቻ ሆኗል ማለት ይቻላል፡፡ ሌላው ሰው ወደፈጠራ እንዲያዘነብል የማድረግ ፍንጭ የታየበት ነው፡፡ ለምሳሌ የተለያዩ የእጅ መታጠቢያ መሳሪያዎችን በቴክኒክና ሙያ ተቋማት የማመረት ሥራ እየተሰራ መሆኑ፤ እጅ መታጠቢያ በተለያየ አካባቢ የተለያየ ዓይነት መመረቱ፤ ሌላው የአኗኗር ዘይቤ መቀየሩ ለምሳሌ ከዚህ በፊት ተቃቅፎ ሰላምታ የሚሰጣጡ ሰዎች ዛሬ ግን የእጅ ሰላምታ ሁሉ የማይሰጡበት ሁኔታ እየተለመደ ነው፡፡

ቁልፍ መረጃ ሰጭ ሰባት

የጋሞ ዞን ትራንስፖርትና መንገድ ልማት መምሪያ

የዞኑ ትራንስፖርትና መንገድ ልማት መምሪያ ከኮቪድ-19 በሽታ ጋር ያለው ቁርኝት ምን ይመስላል? ከታችኛውና ላይኛው መዋቅር ጋር የተፈጠር የስራ ግኑኝነት፤ ከማስተባበርና በአጋሪነት ከመስራት አንጻር፤ ተቋሙን ለተልዕኮ ብቁ ከማድረግ አንጻር ምን ይመስላል?

እንደሚታወቀው ይህ የኮቪድ-19 በሽታ የሀገራችን ስጋት መሆኑ ከተወሳ ጊዜ ጀምሮ ይህን በሽታ ከመከላከል አንጻር እንደዞኑ ተጨባጭ ሁኔታ ለዚህ ምላሽ አሰጣጥ እንዲረዳ በዞኑ ዋና አስተዳዳሪ የሚመራ ግብረ ሀይል ተቋቋሟል፡፡ የትራንስፖርት ዘርፍ የመከላከል ሥራ የሚሰራ ንዑስ ኮሚቴ ተጠሪነቱ ለዐቢይ ግብረ-ሀይል ሆኖ ተቋቋሟል፡፡ ይህ ንዑስ ግብረ-ሀይል ከትራፊክ ፖሊስና ሌሎች ከሚመለከታቸው የተውጣጣ ሲሆን በአራት የከተማ አስተዳደርና አስራአራት ወረዳዎች ተቀናጅተው የበሽታውን መከላከልና ቁጥጥር ሥራ እንዲሰሩ እየተደረገ ነው፡፡ የመከላከሉን ሥራ በሁሉ መዋቅሮች ባሉን መናሄሪያዎች የግንዛቤ ማስጨበጫ ሥራ በሞኒታሪቦ በመጠቀም ቅስቀሳ ማድረግ እንዲሁም ሚኒ ሚዲያ ተጠቅመን አሁንም በመናሔሪያ ቅስቀሳ ማድረግ ሲሆን አስቀድሞ ግን ለዚህ የሚሆን አደረጃጀት በመፍጠር ነው፡፡ ይህ የተዋቀረው ኮሚቴ የቁጥጥርና ግንዛቤ ማስጨበጥ ሥራ እስከታችኛው ወረዳና ከተማ አስተዳደር ድረስ በቅንጅት እየሰራ ነው፡፡

የግንዛቤ ማስጨበጫ መልእክት ምንጩ ከየት ነው?

በሽታን ለመከላከል የሚያግዝ መልእክት በዋናነት ከጤና መምሪያ የምናገኝ ሲሆን ከተለያዩ ማህበራዊ ሚዲያም የምናገነውን እንጠቀማለን፡፡ በማዕከል የተዘጋጁትን የመልእክት ማስተላለፊያ ባኔሮችን ስትኬሮችንና ብሮሼሮች እንጠቀማለን፡፡ ለታችኛውም መዋቅር እንዲደርስ እናደርጋለን፡፡

እንደትራንስፖርትና መንገድ ልማት መምሪያ የኮቪድ-19 በሽታ ጫናና ስጋት ደረጃ ለይቶ ከመረዳት አንጻር ያላችሁን ሁኔታ ምን ይመስላል?

የኛ ተቋም ትልቁ ሚና የህብረተሰብን ስለበሽታው ግንዛቤ ማስጨበጥ እንደመሆኑና ሌሎች የመከላከል ተግባራትን እንዲያከነውኑ ማስፈጸም እንደመሆኑ ተሳፋሪዎች ወደመናሔሪያ ሲገቡ እጃቸውን እንዲታጠቡና የፊት መሸፈኛ ጭንብል እንዲያደርጉ ማድረግ ይገኙበታል፡፡በተሸከርካሪ ውስጥ ገብተው ሲሳፈሩ ሪቀታቸውን ጠብቀው እንዲቀመጡ ይነገርላቸዋል፡፡ እንዲሁም በዚያው አግባብ መቀመጣቸው ቁጥጥር ይደረጋል፡፡ ይህ ሥራ ወደ መናሔሪያ ሲገቡና ሲወጡም ተግባሪዊ እንዲደረግ ተከታታይነት ያለው የግንዛቤ ማስጨበጫ ስራና ቁጥጥር ይደረጋል፡፡ በየመናሔሪያ በተቋቋሙት ማህበራት አማካይኝነት በውስጥ ያሉትን የሚኒ ሚዲያዎችን በመጠቀም ተከታታይነት ያለው የግንዛቤ ማስጨበጫ ስራ ይከናወናል፡፡ እነዚህ ማህበራት ተሳፋሪዎች መናሔሪያ ሲገቡና ሲወጡ የመከላከል ተግባራትን ባግባቡ መከተላቸውን ያረጋግጣሉ፤ እንዲፈጽሙም ያግዛሉ፡፡ በተመሳሳይ ሁኔታ ደግሞ በየመናሔሪያው የሰውነት ሙቀት በመለካት የሊየታ ስራም ይሠራል፡፡

በናንተ ተቋም ደረጃ ሠራተኞቻችሁና ሌሎች ደንበኞቻችሁ ለበሽታ ተጋላጭ እንዳይሆኑ ከማድረግ ረገድስ ምን እያደረጋችሁ ናችሁ?

የኛ ሴክተር ባጠቃላይ ሁኔታ አንጻር ካየን ተጋላጭ የሆነ ተቋም ነው፡፡ አስቀድሞ ራሱን ያልተከላከለ ሌላውን ሊጠቅም አይችልምና አስቀድሞ ራሱን እንዲከላከል ለሠራተኞቻችን የግንዛቤ ማስጨበጫ ሥራ ሰርተናል፡፡ከዚሁ ጋር በተያያዘ አስፈላጊ የጽዳት ዕቃዎችንም አከፋፍለን ሰጥተናቸዋል፡፡ በተቻለ መጠን ድጋፍ ተደርጎ ወደ ሥራ የተገባበት ሁኔታ አለ፡፡ በተጨማሪ በመናሔሪያ የሚሰሩት ማህበራትንና እዚያው ለሚሰሩት ሰራተኞች የንጽህና መጠበቂያ ቁሳቁሶችን ማለትም እንደ ሳኒታዘር የፊት መሸፈኛ ጭንብሎችና ሳሙና የመሳሰሉትን እንዲያገኙ ተደርገዋል፡፡ ሙሉ በሙሉ በተሳካ መልኩ አድርገናል ባንልም አቅማችን በፈቀደው ልክ ለታችኛው መዋቅር ራሳቸውን እየተከላከሉ ለሌላው አግልግሎት እንዲሰጡ ጥረት እየተደረገ ነው፡፡

ለዚህ ሥራ መነሻ የሆነ በዚሁ ልክ እንድትንቀሳቀሱ ያደረገ ምንድነው? መነሻ ከየት አገኛችሁ?

መጀመሪያ ከባለድርሻ አካላት ጋር ለመሥራት ከአስቸኳይ ጊዜ አዋጅ ጀምሮ የቁጥጥር ሥራ ለማከናወን የሚያስችል የጋራ ዕቅድ ሰነድ ተዘጋጅተዋል፡፡ ከዚያም በተዘጋጀው ዕቅድ ዙሪያ ሁሉንም ባለድርሻ አካላት ጋር የጋራ የማድረግ ሥራ ተሰርቶ ነው ወደ ትግበራ የተገባው፡፡ መነሻው አስቸኳይ ጊዜ አዋጅ ቢሆንም ወደኛ ነባራዊ ሁኔታ በመቀየር በተለይ ዞናችን ያለበትን ጂኦግራፊያው አቀማመጥና ግኑኝነትን ታሳቢ ያደረገ ዕቅድ መስራት ስላስፈለገ ነው፡፡ ለምሳሌ ዞናችን የሚያዋስኑትና መግቢያ በሮች በሰሜን በሀሜሳ ወንዝ ከወላይታ በኩል ያለው መግቢያ ሌላው ወደቁጫ መግቢያ ጎጋራ በኩል በኮንሶ በኩል በሁሉም መግቢያዎች አካባቢ ቁጥጥርና የሚገቡ ሰዎች ተመርምረው እንዲገቡ የማድረግ ሥራ እየሰራን እንገኛለን፡፡ መምሪያችን ለዚህ ሥራ መነሻ የራሱ ዕቅድ አዘጋጅተውና በየደረጃ ካለው የራሱ መዋቅር ጋር ተግባቦት ፈጥረው ነው ወደሥራ የገባው፡፡

የኮቪድ -19 በሽታ መከላከል ሥራ በመምሪያ ደረጃ የሚያስተባብር የተለየ ባለሙያ ወይንም የተመደበ ስለመሆኑ፤

በኛ መምሪያ ነባራዊ ሁኔታ አንጻር የመምሪያው ሃላፊ ነው ይህንን ሥራ እያስተባበረ ያለው፡፡ የመምሪያው ሃላፊ በጋራ በግብረሀይል ደረጃ የሚነደፉትን ሥራዎችን ወደመምሪያችን በማምጣት ለተዋቀረው ንዑስ ኮሚቴ ኦሪየንት በማድረግ ይሰጣሉ፡፡ አፈጻጸሙን በጋራ ይግመግማል፡፡ አስቀድሞ ታቅደው ወደታች የወረደው ሥራ በዕቅዱ መሰረት ስለመከናወኑ በጋራ ተገምግሞ ከተለየ በሓላ ማሻሻያና መከላሻ ታክሎ እንደገና ወደታችኛው መዋቅር ይወርዳል፡፡

እንግዲህ ከላይ እንደተጠቀሰው መምሪያችሁ በሽታውን በመከላከል ረገድ የጋራ ዕቅድ አዘጋጀተው በቅንጅት እየሰራ እንደሆነ ተጠቅሰዋል፡፡ ይህ ሥራ የራሱ የሆነ የማሥፈጸሚ ግብዐት ያስፈልገዋል፡፡ ከዚህ አንጻር መምሪያችሁ ግብዐትን ከመመደብና ከማፈላለግ አንጻር ያለው ቁመና ምን ይመስላል?

እንግዲህ የዚህን በሽታ ለመከላከል ሀብት ማስፈለጉ ዕሙን ነው፡፡ በኛ በኩል የተወሰኑትን ቁሳቁሶች ወደታችኛው መዋቅር ልከናል በቂ ባይሆንም፡፡ በሥራችን ላሉት 12 ለሚሆኑ መናሔሪያዎች የሳኒታይዘር ሳሙናና የፊት መሸፈኛ ጭንብል አከፋፍለናል፡፡ ለትራፊክ ፖሊሲና ለመንገድ ደህንነት ሰራተኞች ከዞን የሚደረግ ድጋፍ አናሳ ነው፡፡በወረዳ እና የከተማ አስተዳደሮች መዋቅር ሀብት የማሰባሰብ ሥራ ቢኖርም ለአገልግሎት በቂ አይደለም፡፡

በመምሪያ ደረጃ የታቀዱትን ሥራዎች አፈጻጸም የምትከታተሉበትና የምትደግፉበት አሰራር ስለመኖሩ ቢገለጽ፤

በርካታ የቁጥጥር ሥራዎች ተሰርተዋል፡፡ ከአዋጁ ጋር ተያይዞ ከዚህ በፊት ትርፍ ጭኖ የሚሄዱበት ሁኔታ በስፋት ይከሰት የነበረው አሠራርም የላላ እንደነበረ ይታወቃል፤ የቅጣት ሁኔታም አንድ ሰው ትርፍ ጭኖ ከተገኘ ደንብ ቁጥር 395 /2009 መሠረት ነው የምንመራው ፤ ሆኖም ግን በአስቸይ ጊዜ አዋጅ ጋር ተያይዞ የመጀመሪያ ደንብ የላላና አሁን ባለው ሁኔታ ወረርሽኝን ለመከላከልና ለመቆጣጠር እንደማያስችል ስለታመነበት የተሻሻለው መመሪያ በሰርኩላሪ ደርሶናል፡፡ አዲሱ መመሪያ አንድ አሽከርካሪ አንድ ሰው በትርፍነት ከጫነ አንድ ሺህ ብር እንዲቀጣ ተደንግገዋል፡፡ ይህንን መነሻ በማድረግ በርካታ ተሸከርካሪዎች ተቀጥተዋል፡፡ እስከ ትላንትና 12/10/2012 1293 አሽከርካሪዎች በኛ ዞን ተቀጥተዋል፡፡ ከዚያ ውስጥ 1836700 (አንድ ሚለየን ስምንት መቶ ሰላሳ ስድስት ሺህ ሰባትመቶ) ብር ገቢ ሆነዋል፡፡ በርግጥ ዋናው ዓላማ ገንዘብ የማሰባሰብ ሳይሆን ህብረተሰቡ ለበሽታ እንዳይጋለጥ ማድረግ ቢሆንም አንዳንድ አሽከርካሪዎች በሚነገራቸው መረጃ መሥረት ለሕዝብ ጥቅም ቅድሚያ ከመስጠት ይልቅ ለሀብት ስለሚስገበገቡ አስተማሪ ቅጣት መውሰድ ግዴታ ስለሆነ ነው፡፡ እነዚህ የተከናወኑ ሥራዎች ከዕቅድ አንጻር የት ደረጃ እንዳሉና ምን ጠንካራና ደካማ ጎኖች እንደነበሩ በዞን አጠቃላይ የትራነስፖርት ኮሚቴ ደረጃ ውይይት እየተደረገ መሻሻል ባለባቸው ጉዳዮች ተለይተው ከታችኛው መዋቅር ጋር እየተናበብን የምንሄድበት አሥራር አለ፡፡ከዚህ በፊት ሳይቆራረጥ ነበር ነገር ግን ከባለፈው ሁለት ሳምንት ወዲህ መቆራረጥ ይታያል፡፡ ወደወረዳዎችም ለድጋፋዊ ክትትል ወጥተን ነበርና ጥሩ ሥራዎች እየተሰሩ ነው፡፡

ቅድም እንደጠቀሱት ከተለያዩ ማህበራት ጋር ሥራ ከፋፍላችሁ ሰጥታችሁ በጋራ እየሰራችሁ እንደሆነ ተጠቅስዋልና እነዚህን ስራዎች ወደታለመለት ግብ እያመሩ ስለመሆኑስ እንዴት ነው የምትከታተሉት?

አንግዲህ ይህን ሥራ የምናከናውነው ከተለያዩ ባለድርሻ አካላት እንደመሆኑ በኮሚቴ የሚሰራ ሥራ በባህሪይው ይንጠባጠባል፡፡ዞሮ ዞሮ ይሄንን ምንድነው የምናደርገው? እንደማህበራት እነርሱን ከማጠናከር አንጻር በርካታ የግንዛቤ መረጃ ሰጥተን ከዚያ በመነሳት ቁጥጥር አንድ ሰው በትርፍነት እንዳይጫን የማድረግ ሥራ እንዲሰሩ ማድረግንና ሌሎችን በዕቅድ አስረግጠን የሰጠናቸው ተግባራት በዕቅድ መሠረት መከናወናቸውን ቼክሊስት አዘጋጅተን ከነርሱ ጋር በየሳምንቱ ውይይት እናደርጋለን፡፡ አንዳንዴ ሁኔታዎች አሳሳቢ በሚሆኑበት ወቅት በየሶስት ቀናት ሁሉ ክትትል እናደርጋለን፡፡ የስምሪት ዳይረክቶሬት በየቀኑ የሚከናወነውን የተሸከርካሪ ስምሪትና አጫጫን ሁኔታ እየተከታተሉ ለኛ ሪፖርት ያቀርባሉ፡፡ምክንያቱም ማህበራትም የሰዎች ስብስብ እንደመሆናቸው መንጠባጠብ ስለማይቀር የክትትል ሥራን በዚሁ አግባብ እያስኬድን እንገኛለን፡፡

ከሌሎች ተቋማት ጋር በዚህ ሥራ እንቅስቃሴ ወቅት ያላችሁ ቅንጅታዊ አሰራር ምን ይመስላል?

ዞሮ ዞሮ ይህ ሥራ ሁሉም ባለድርሻ አካላት ካልተረባረቡ መሳካት አይቻልም፤በዋናነት እንደዞናችን ትራንስፖርት ኮሚቴ እያደረግን ያለነው፡ ቅድም ለማነሳት እንደሞከርኩት የኛ ባለድርሻ አካላት የዞን ጤና መምሪያ፡ የትራፊክ ፖሊስ ተናብበን ነው የምንሰራው፡፡ ከነርሱ ጋር ካልተናበብን ሥራችን ምንም ሊሳካ ስለይማችል ይህን ከባለድርሻ አካላት ጋር ተገቢ ቁርኝትና ቅንጅት ፈጥረን መስክ ስንወጣም አንድ ላይ ቼክሊስት አዘጋጅተን ነው ድጋፋዊ ክትትል የምናካሂደው፡፡

ከጤና ጋር ያለው ቅንጅታዊ አሰራር እንዴት ይገለጻል?

ከጤና ጋር ያለው ቁርኝት በዚህ ጉዳይ ላይ እጅግ ጠንካራ ነው፤ ያለጤና ምንም መስራት አንችልም፤ እኛ አንድ ተሸከርካሪ ሰውን አሳፍሮ ወደ መዳረሻው ሲደርስ ተሳፋሪዎች የሙቀት ሊኬት እንዲያደርጉ እናደርግ ሙቀት ከፍ ያለው ተሳፋሪ ካለ ወደለይቶ ማቆያ የሚወስደው የጤና መዋቅር ነው፡፡

ከትራንስፖርት አገልግሎትና ከህዝብ እንቅስቃሴ አንጻር እንደስጋት የምነሳው ጉዳይ በተለይ ባሁኑ ጊዜ በሽታው በስፋት ከሚገኝባቸው አካባቢዎች ሰዎች ተሳፍረው ስንቀሳቀሱ ወደመዳረሻ የሚደረገው ጉዞ ማን ከየት ተነስተው የት ሄድ የሚባለው በሚገባ ከመከታተል አንጻር በናንተ በኩል እየተደረገ ያለው ስራ ምን ይመስላል?

የሰው እንቅስቃሴ በብዙ መልክ ይከናወናል፤ አንደኛው ሰው በራሱ እግር ይጓዛል፤ ተላየዩ የትራንስፖርት ምንጮችን ይጠቀማል፡፡ነገሩ በራሱ ከባድ ነው፤ ባለፍው የትራነስፖርት እንቅስቃሴ በክልላችንም በዞናችንም ጭምር ተዘግቶ ነበር፡፡በዚን ወቅት ህገወጦች የተበራከቱበት ጊዜ ነበር፤የተፈቀደላቸው ጭምር ትርፍ መጫንና መረበሽ ሁኔታ ታይቶ ነበር፡፡ሌላ ድርብ ሥራ ወደመሆን የደረሰበት፤ ይህም የሚገለፀው በግል ለቤት አገልግሎት የተፈቀደላቸውና የመንግስት ተሸከርካሪዎች ወደዚህ ሥራ የገቡበት ሁኔታ ነበር፡፡ባለሁለትና ሶስት እግር ተሸከርካሪዎች ከተፈቀደላቸው በብዙ ቁጥር ትርፍ ጭነው የሚተረማመሱበት ሁኔታ ነበር፡፡ይህ ሁኔታ ተገምግሞና ምን መደረግ እንዳለበት ውይይት ከተደረገ በኋላ ከአስቸኳይ አዋጅ ጋር በመገናዘብ እንደገና መናሔሪያ እንዲከፈት የተደረገበት ሁኔታ አለ፡፡ ዞሮ ዞሮ እግረኛውም ወደህብረተሰቡ የሚገባ እንደመሆኑ ከባድ ነው፡፡

ተሳፋሪዎች ከአንድ መነሻ ተሳፍረው ወደመዳረሻ በሚጓዙበት ጊዜ ማን ከየት ተሳፍረው የት ወረደ የሚል ክትትል የሚደረግበት መዝገብ ስለመኖሩ፡-

አሁን በርግጠኝነት አሱን ማዉራት ከባድ ነው፡፡ለምሳሌ ከአዲስ አበባ መውጫ አርባምንጭ ብሎ የተነሳው ተሸከርካሪ በየመንገዱ እያወረደና እያሳፈረ ስለሚመጣ ይህንን መቆጣጠር ከባድ ነው፡፡ ምናልባት ለህልናው ተገዥ ካልሆነ በቀር፡፡ ሲሳፈሩ በቁጥርም ሆነ የት እንደሚሄዱ ማድረግ የሚቻል ቢሆንም በየመንገዱ የሚወርዱትን አድራሻቸውን ተከታትለው ማስፈጸም ከባድ ነው፡፡

በሽታው በሀገራችን እየጨመረ መምጣቱን መረጃዎች ያሳያሉና ምን ሊደረግ ይገባል ይላሉ?

በዚህ ረገድ ሁለት ጉዳዮችን ማንሳት እፈልጋለሁ፤ አንደኛ አሽከርካሪዎች ከመናሔሪያ የተመረመሩትን ተሳፋሮዎችን ጭነው ወደታወቀ መዳረሻ ማሳፈርና ሪፖርት ማድረግ ይጠበቅባቸዋል፤ ሁለተኛ ደግሞ ተሳፋሪም በየመንግድ መሳፈር የለባቸውም፤ በሽታው አደገኛ መሆኑን ተረድተው ከማን ጋር እንደተሳፈሩት ማን ምን እንደሆነ ቢያንስ ሊየታ ባልተደረገበት ሁኔታ መሳፈር ባይምክሩ ይሻላል፡ ይህ የጋራ ሃላፊነት ስለሆነ ነው የሚለው፡፡ሁሉም የየበኩሉን ሃላፊነት ካልተወጣ መጠፋፋት ይመጣል የሚል ስጋት አለኝ፡፡ አሽከርካሪዎችም በዚህ ጊዜ ከገንዘብ ይልቅ ለህሊናቸው ቢኖሩና በኛ በኩልም የቁጥጥር ሥራ በየቅብብሎሽ ቦታ ተገቢ ቁጥጥር ሥራ መከናወን ያስፈልጋል፤ ለምሳሌ ከዚህ አርባምንጭ ተነስተው ወደ ቁጫ የሚሄድ ተሽከርካሪ በሚያለፍባችው ቦታዎች በምዕራብ ዓባያ በሁምቦ በወላይታ ሶዶ ገሱባ ዳና እስከ ሰላም በር ድረስ በየመተላለፊያቸው የተቀናጀ ወጥ የሆነና ከኪራይ ሰብሳቢነት በነጻ መልኩ ቁጥጥር ያስፈልጋል፡፡ ሁሉም ድርሻቸውን በተገቢው ቢወጡ የሚል አስተያየት አለኝ፡፡

እንደመምሪያው በርካታ ጉዳዮች ተነስተዋል፤ እንደዞን የታቀዱት ዕቅዶች ወደታለመለት ግብ ስለመምራታቸው ያለዎት ግምገማ ምን ይመስላል?

ይህ የኮሮና በሽታ መከላከልና ቁጥጥር ሥራ በዚህ ጊዜ ይቆማል የሚል ግልጽ የሆነ የጊዜ ገደብ የለም፤አንደዞን ይህ በሽታ ገዳይና ዘርፈ-ብዙ ተግዳሮትን እያስከተለ መሆኑ የሚታወቅ እንደመሆኑ አስቀድሞ በጋራ የታቀዱትን ግቦችን ለማሳካት የጋራ ክትትልና ግምገማ እንደሚያስፈልግ በመገንዘብና በአፈጻጸም ሂደት ያጋጠሙትን ክፍተቶችን እያረሙ መሄድ አስፈላጊ ነው፡፡እሰካሁን የተገበርናቸው በጥንካሬና ድክመት የታዩትን እንደየሁኔታዎች ተቀብለን ማሻሻያ የሚያሰፈልጉትን የአሰራር ማዕቀፍ ማሻሻያ እንከዋን ቢስፈልግ አዋጅና ደንብንም እስከማሻሻል መሄድ ተገቢ ይሆናል፡፡ሁሌ በጥንካሬ መንቀሳቀስን የሚፈልግ እንደመሆኑ ከዓላማው ያለመንሸራተት አስፈላጊ ነው፡፡ በዚህ አግባብ የክትትልና ግምገማ ሥርዓት ተዘርግቶ እየተተገበረ ይገኛል፡፡

የኮቪድ-19 ወረርሽን ከመከሰት ጋር በተፈጠረው የአሰራር ሂደት እንደመልካም አጋጣሚ ሊጠቀሱ የሚችሉ ሁኔታዎች ካሉ?

ዘርፋችን በባህሪዩ በርካታ የሰውና የንብረት ውድመት እየተፈጸመ የቆየ ዘርፍ ነው፤ ከኮሮና ጋር ስናነጻጽር የኮሮና በሽታ በተወሰነ ደረጃ ተይዘውም የማገገም ዕድል አለ፤ ነገር ግን ትንሽ የሚመስል የትራፊክ አደጋ ለበርካታ የሰው ልጅ ህይወት መቀጠፍ ምክንያት ሲሆን እናያለን፡፡በመሆኑም የትራፍክ አደጋን (በሞት፣ በከባድና ቀላል የአካል ጉዳት) እንደሀገር አስር በመቶ በየዓመት ለመቀነስ ግብ ተጥሎ እየተሰራ ይገኛል፡፡የትራፍክ አደጋ መከላከል አሁን ከኮቪድ-19 መከላከል ጋር በስብጥር እየተሰራ ነው፡፡ይህ ብቻ ሳይሆን አዲስ አዋጅ በመውጣትና የቁጥጥር ሥራ በቅንጅታዊ መንገድ እየተከናወነ በመሆኑ ትርፍ አለመጫን ብቻ ሣይሆን ወትሮ በመደበኛነት ከምጭኑበትን በመቀነሱ ለትራፍክ አደጋ የሚዳረጉት ክስተቶች (ከተፈቀደው ፍጥነት በላይ ማሽከርከር ቀቤቶ አለማድረግ) በመቀናሳችው አደጋውም እንደዞናችንም ሆነ እንደክልል የቀነሰ ይመስላል፡፡ ይህ የሆነው ሁሉም ባለድርሻ አካላት ስለተረባረቡት ነው፡፡ እንዲሁም ሥራዎቻችን በተመጋጋቢ ሁኔታ በጋራ እንድንፈጽም እድል ፈጥረዋል፡፡

አዳዲስ ቴክኖሎጂዎችን በመጠቀም ረገድስ የፈጠረው ዕድልና አጋጣሚ ካለ?

እንደትራንስፖርት ዘርፍ አራት የአሰራር ምሰሰዎች አሉን፡፤ 1ኛ የግንዛቤ ማስጨበጫ በየትምህርት ቤቶች ክበባት አሉን፤ ነባሮችን የማጠናከርና አዳዲስ በማዋቀር የኮቪድን መከላከልን ስራ ጨምሮ መስራት፡ መንገድ ዳር ቀበሌዎች ኮሚቴን በማቋቋም ህብረተሰቡ ራሱን ከትራፍክ አደጋ እንዲከላከል የማድረግ ሥራ ትርፍ እንዳይጫን ተደርበው እንዳይሄዱ እግረኛው የራሱን መንገድ አውቀው እንዲጓዝ ማድረግ፤ ከዚያ ውጭ ህዝብ በሚበዛባቸው አካባቢዎችና በሀይማኖት ተቋማት በሞንታሪቦ በመጠቀም የግንዛቤ መልዕክት የምናስተላልፍበት አንዱ ሲሆን ሁለተኛው ደግሞ የእንጂነሪንግ ሥራ ነው፡፡ እነዚህ በመንገድ ላይ የሚደረጉ ምልክቶች መረጃ የሚሰጡ የሚያስጠነቅቁ የሚከለክሉ ናቸው፡፡ እነዚህን በተገቢ ቦታ በማስቀመጥ አሽከርካሪዎችንና መላው ህብረተሰብ ግንዛቤ ኖሮአቸው እንዲተገብሩ ማድረግ ነው፡፡ ቴክኖሎጂ የተጠቀምነው በቅጣት አፈጻጸም ላይ ነው፡፡ አንድ ተሽከርካሪ ህግ ከተላለፈ ማስተማሪያ የሚሆን ቅጣት ይቀጣል፤ ይህንን የምንፈጽምበት ሶፈትዌር አለን፡፡ ይህ ከታች እስከ ላይ ድረስ የተናበበ ነው፡፡ በዚህ ረገድ የኛ ክልል ጥሩ ሥራ እየሰራ ይገኛል፡፡

ቁልፍ መረጃ ሰጭ ስምንት

የሠራተኛና ማህበራዊ ጉዳዮች መምሪያ

የዞኑ ሠራተኛና ማህበራዊ ጉዳዮች መምሪያ የኮቪድ-19 ወረርሽን ከመከላከልና ቁጥጥር ጋር ያለው ቁርኝት ምን ይመስላል?

በመሠረቱ የዞኑ ሰራተኛና ማህበራዊ ጉዳይ መምሪያ በዚህ የኮቪድ -19 ላይ ሲሰራ ባለቤት ሆኖ ነው ፡፡ ባለድርሻዎችም በርካታ ናቸው፡ ­- ጤና፣ ሴቶች ህጻናትና ወጣቶች፣ ምግብ ዋስትና፣የተለያዩ የሀይማኖት ተቋማት እና ዕድሮችም ሳይቀሩ የኛ ባለድርሻ አካላት ናቸው፡፡ ከመንግስታዊ ተቋማት በበለጠ ከዕድሮች እና ዕቁቦች ጋር ተጣምረን እንሰራለን፡፡ ከዩኒቨርስቲዎችና ኮሌጆች ጋር ጠንከር ያለ ግኑኝነት አለን፡፡እነዚህ ባለድርሻ አካላት አንዳንዴ እኛ ከመቀስቀሳችን አስቀድሞ በመምሪያችን በኩል ሊሰራ ይገባል ብለው ያሰባቸውን ሥራ ከነግብዓት ወደኛ እስከመላክ ድረስ ቁርኝት ፈጥረን እየሰራን እንገኛለን፡፡ መጀመሪያ ሲነሳ አደረጃጀት ፈጥሮ ነው፡- ዐቢይ ኮሚቴ እና ንዑሳን ኮሚቴ ተዋቅሮ እና የጋራ ዕቅድ ተዘጋጅተው ነው ወደሥራ የተገባው፡፡ በዚህ ዕቅድ የተካተተው አስቀድሞ ተጋላጭ የሚሆኑትን የመለየት ማን ምን መሥራት አለበት የሚል ተለይቶ ነው፡፡

እንደሠራተኛና ማህበራዊ መስሪያ ቤት ለኮቪድ በሽታ ይበልጥ ተጋላጭ ይሆናል የምትሉአቸው የህብረተሰብ ክፍሎች ካሉ ገልጸው ቢያልፉ፤

እኛ ተጋላጭ ናቸው የምንላቸው ሁልጊዜ የኛ ማህበራዊ መሰረት ናቸው ብለን ይዘን የምንሰራባቸው አሉ፤ጧሪና ቀባሪ የሌላቸው አረጋዊያን፣ አካል ጉዳተኞች፣ ለምኖ አዳሪዎች፣ ጎዳና ተዳዳሪዎች፣ ወላጅ አልባ ህጻናት እና ሴትኛ አዳሪዎች ናቸው፡፡እነዚህ በይበልጥ ትኩረት ሰጥተን ችግራቸውን ለመቅረፍ የምንንቀሳቀስባቸው ናቸው፡፡ ወደቤተስብ የሚመለሱትን ወደ ቤተሰብ የመመለስ ሥራ እንዲሁም በጣም ተጋላጮች በሆነ ሁኔታ ላይ ያሉትን ወደማቆያ ለጊዜው በማስገባት ምግብና አልባሳት የምንሰጠበትና የግል ንጽህና መጠበቂያ ቁሳቁሶች፡- እንዴ ሳኒታይዘር የመሳሰሉትን ለማዳረስ ተሞክረዋል፡፡ ፖሊስ፣ ሴቶችና ህጻናትና የከተማ አስተዳደር እስከቀበሌ ድረስ ያሉትን አካላትን ይዘን ሰርተን ውጤታማ የሆንንበት ሁኔታ አለ፡፡ ይህ ሥራ እዚህ ከተማ ብቻ ሳይሆን እስከ ወላይታ ዳዉሮ እና ኮንሶ ድረስ ተግባራዊ ያደረግንበት ሁኔታ አለ፡፡ ቁርኝታችን በዚህ መልክ እስከታችኛው መዋቅር ድረስ ነው የተዘረጋው፡፡ መዋቅራችን እስከወረዳ መኖር ብቻ ሳይሆን የዘረጋናቸው ህዝባዊ አደረጃጀቶች እስከቀበሌ ድረስ ነው፡፡በወረዳ ደረጃ ያለው ጽ/ቤት ሆኖ በቀበሌ ደረጃ ሶስት ሶስት በጎ መልእክተኞች አሉ፡፡እነዚህ ሰዎቸ በየቀበሌ ያሉትን ይበልት ተጋላጭ የሆኑትን መልምሎ ለኛ ይልካሉ፡፡እውነትን ለመናገር በዚህ ዙር የተሻለ መረጃ የተገኘው ከኛ ዞን ነው፤ ምክንያቱም እነዚህ የፈጠርናቸው ማህበራዊ መሰረቶችና አደረጃጀቶች በተጋላጮች ሊየታ እጅግ በጣም ውጤታማ መረጃ በማቅረባቸው ነው፡፡ በቁጥርና በእያንዳንዱ ቀበሌ ያሉትን ተጋላጮችን በስም ለይተው አቅርበዋል፡ በመሆኑም ከዚህ ድጋፍ ሲላክ ዝም ብለን በቁጥር ሳይሆን ለማን እንደሆነ አረጋግጠን ነው ፡፡ ሁል ጊዜ ቴሌግራምና ኢሜይል አድራሻችን በተጠንቀቅ ነው የሚጠብቀዋቸው፡፡ በዚህ ዓይነት ሁኔታ የተፈጠሩትን አደረጃጀቶችንና ቀበሊያትን ከኛ ጋር ያሰለፍንበት ሁኔታ ነው ያለው፡፡ የተፈጠረው ጠንካራ አደረጃጀት አንዳንዴ ጠፍተው የሚሄዱትን አግኝተው ከኛ ጋር እንዲያገናኙ አድርገዋል፡፡ ከኛ ጋር በጋራ ከሚሰሩት ተቋማት መካከል አንጋፋው የአርባምንጭ ዩኒቨርሲቲ በርካታ ድጋፍ አድረገዋል፡፡ የምግብ እና የንጽህና ግብዐቶችን ሰጥተውልን ለተጠቃሚዎች አድለናል፡፡ የሀይማኖት ተቋማት በበኩላቸው ደግሞ ለምኖ የሚያደሩትን በማገዝ ላይ ናቸው፤ እነዚህ ለምኖ አዳሪዎችን አንስተን በተወሰነ መጠለያ ለማኖር በርካታ ጊዜ ብንሞክርም ተመልሰው በቤተክርስቲያን አካባቢ ስለሚጠለሉ ከቤተክነት አስተዳዳሪዎች ጋር በመመካከር አስተዳደሩን እነርሱ እንድያግዙን አድርገን በኛ በኩል የምንገዛቸውን እንደንጽህና መስጫ ዕቃዎችን እንሰጣቸዋለን፡፡ ለመጥቀስ ያክል እንደመድሃኒዓለም ገብርኤልና ሚካኤል ቤተክርስቲያኖች አካባቢ ላሉት የተወሰኑትን ግብዓቶች ሰጥተናቸው የማስተዳደሩን ሃላፊነትን እነርሱ እንዲወጡ እያደረግን ነው፡፡

ይህንን በሽታ መከላከልና ቁጥጥር ስራ ውስጥ እንድትገቡ መነሻ የሆነ ነገር ምንድር ነው በዚህ ረገድ እንደመነሻ የተጠቀማችሁት ፖሊሲና ደንብ እንዲሁም ዕቅዶች ካሏችሁ ቢገለጽልን፤

በነገራችን ላይ ይህ ለኛ እንደሠራተኛና ማህበራዊ ጉዳይ መምሪያ ግልጽ ነው፡፡ ሲጀመር የማህበራዊ ደህንነት ፖሊሲ መጀመሪያውም መዳረሻውም ይህ ነው፡- ይህ የመደጋገፍ ሥራ ዘላቂነት ሊኖር በሚችል መልኩ እዚያው ህብረተሰቡጋ ጀምሮ እዚያው እንዲያበቃ በሚያሰችል መልኩ የተቀረጸ ነው፡፡ አሁን ልማታዊ ሴፍትኔት እንበለው በዕድር ደረጃ የሚሰበሰበው የግሪን ባንክ እንበለው በዚህ የፖሊሲ ማዕቀፍ አማካይኝነት የሚተገበር ነው፡፡ ይህ ፖሊሲ በዋናነት ህብረተሰቡ ራሱን በራሱ የሚደግፍበትን የአሰራር ማዕቀፍ መነሻ ያደረገ ነው፡፡ከኮቪድ-19 ምላሽ ጋር በተያያዘ እንደመምሪያችን ያገኘነው መልካም አጋጣሚ ሌሎች ስለሥራችን ግንዛቤ እንዲያገኙ ማድረጉ ነው፡፡ እኛ ከዚህ በፊት ብቻችን ስንጮህ የነበርነውን ዛሬ አድማጭ እንድናገኝ ማድረጉ እንጂ ይህማ እኛ አስቀድመን የምንጠቀምበት አሰራር ነው፡፡ የኛ የሁል ጊዜ ሥራችንና እነዚህ ማህበራዊ ተጋላጮችም ሁልጊዜ የነበሩ ናቸው፡፡

ከዚህ ጋር ተያይዞ በዋናነት ቅንጅታዊ አሠራር አፈጻጸሙ ምን እንደሚመስልና በምን በምን ዓይነት ሁኔታ እንደሚተገበር ዘርዘር አድርገው ቢገልጹ

እኛ እንደሀገር የተጋላጮችን (አረጋዊንና የአካል ጉዳተኞች) ድጋፍ ማዕቀፍ ፖሊሲ ስንቀበል ፖሊሲ ቀርጸን ስንወጣ መቼና ለምን ዓይነት ተጠቃሚነት እንደምናበቃ ግብ አስቀምጠን ነው የተነሳነው፡፡ እዚህ ግብ ለማድረስ ሠራተኛና ማህበራዊ ጉዳይ መምሪያ ብቻ ሰርተው ያሳካል የሚል እምነት የለም፡፡እዚህ ላይ የሚሳተፉ ባለድርሻዎች በርካታ ናቸው፡፡ ሌላው ደግሞ ሥራው የት ሊሆን ሲገባው የት ነው የተጣለው የሚለውንም እንድናይ አድርገዋል፡፡ ሥራው ሠራተኛና ማህበራዊ ጉዳይ መምሪያ ሊያከናውን ሲገባው አንዱ ወጣ ብለው ምግብ ዋስትና ውስጥ ይገኛል፤ ለምሳሌ ልማታዊ ሴፍትነት፤ይህ ፕሮግራም በዚህ ማህበራዊ መሰረት የሚሠራ ሥራ ነው፤ ነገር ግን በተዛባ ሁኔታ ተጠቃሚነት ሌላጋ ወጣ ብሎ የነበረበት መልክ ነበር፡፡ ከዚህ ጋር ተያይዞ የተጋላጮች ትክክለኛ መረጃ እንከዋን ማግኘት የተቻለው ከኛ መስሪያ ቤት ነው፡፡ በመሆኑም ሥራው ሊሰራ የሚገባበት ቦታና ሥራው ያለበት ተራሪቆ የነበረበትን በዚህ ኮቪድ ምላሽ በጋራ ተቀናጅተን መስራት ስንጀምር የትኛው ሥራ የት መከናወን በትክክል ያለንበት ሁኔታ ታይተዋል፡፡ እኛም የትጋና እንዴት መደገፍ እንዳለብን ያየንበት ሁኔታ ነው፡፡ የኛ ተቋም ይደገፍ ስንል በዋናነት ለእነዚያ ማህበራዊ መሰረቶቻችን የሆኑ ተጋላጮች ማለት እንጂ ለተቋሙ ማለታችን አይደለም፡፡ በተለይ ተቐማት ሲደራጁ ግልጽ በሆነው መሰረት መሆን እንዳለበት በዚህ ቅንጅታዊ ክንውን ወቅት ያየንበት ነው፡፡ ሌላው በርካታ ችግሮቻችን የጋራ ጥረት የሚያስፈልጉት ስለመሆናቸው እንደሠራተኛና ማህበራዊ መምሪያ አስቀድመን ያየንበትና እነዚህን ተጋላጭ የሆኑትን ህብረተሰብ አካላትን ከጎዳና አንስተን ወደመጠለያ ለማስገባት ከአርባምንጭ ዩኒቨርስቲ ጋር ተደጋግፈን ቤት አስገንብተን ቀለም ደረጃ ደርሰን እያለን ነው ይህ ኮቪድ በሽታ የመጣው፤ ከኮቪድ ጋርም ተያይዞ ይበልጥ ተጋላጭ የሚሆኑትን የህብረተሰብ ክፍሎች መረጃ ከኛ ጋር ሲያገኙ እኛም ደስ ብሎናል፡፡ ሰው ልጅ ድጋፍ ሲያገኝና ወገን አለኝ ብሎ ሲጽናና ማየት የሚያረካ ነው፡፡

እንደሰራተኛና ማህበራዊ ጉዳይ መምሪያ የኮቪድ -19 በሽታ መከላከልና ቁጥጥር ሥራ በተቋማችሁ እስከታችኛው መዋቅር ድረስ እና የማህበራዊ መሠረታችሁ የሆኑትን አረጋዊያንና አካል ጉዳተኞችን ለመከላከል ያለው ዝግጁነት ምን ይመስላል? ይህንን ሥራ ተከታትለው የሚያስፈጽም አካል ከመመደብም አንጻር

አው ቅድም እንዳልኩት የመምሪያው ሃላፊ የዐቢይ ግብረሀይል አባል ነው፤በንዑሳን ኮሚቴዎች ውስጥ እኛ በተለያየ ደረጃ ያለን የሥራ ሀላፊዎች አለን፡፡ ይህ አደረጃጀትና አሰራር እስከወረዳ እንዲደርስ አድርገናል፡፡ ወደወረዳ ስወርድ ደግሞ ቅድም የገለጽናቸው ባለድርሻ አካላት፡- ዕድሮች ዕቁቦች የሀይማኖት ተቋማት ና ባለሀብቶች ቀረብ ብለው እንዲሳተፉ ያደረግንበት አግባብ አለ፡፡ ስለዚህ እንደፎካል ሰው ብቻ ምድበን የምናስፈጽመው ሳይሆን እንደኮሚቴ ተደራጅተን ነው የምናስፈጽመው፡፡ የመምሪያው ሃላፊ አፈጻጸሙን በየደረጃው ካሉት ንዑሳን ኮሚቴዎችና የማኔጅመንት አባላትና ባለሙያዎች ያገኛል፡፡

ተቋሙ ለኮቪድ-19 ምላሽ የሚሆን የራሱ ዕቅድ ስለመኖሩ

አዎን በመምያሪችን ይህንን ጉዳይ ተከታትልን ምላሽ የምንሰጥበት የራሱ የሆነ ዕቅድ ተዘጋጅተዋል፡፡ ዕቅዱ እንዳንዱ ዐቢይና ንዑሳን ኮሚቴዎች ምን ምን መስራት እንዳለባቸውና ተጋላጮች እነማን እንደሆኑና የት እንደሚገኙ ሁሉ ያካተተ ነው፡፡እያንዳነዱ ተጋላጭ በዓለምአቀፍ ስታንዳርድ መሠረት ምን ያህል እህልና ዘይት እንደሚያስፈልግ እነዚህን ሁሉ ያካተተ ዕቅድ ነው፡፡

በተቋም ውስጥ ለሚከናወነው ሥራ ግብዐት ከመመደብ አንጻርስ?

መጀመሪያ አካባቢ ያለዝግጅት ስለመጣብን ተንገዳግደን ነበር ግን ወዲያዉኑ አስፈላጊ የሆኑትን የመከላከያ ቁሳቁሶችን ያሟላንበት ሁኔታ አለ፡፡በኛ ተቋም ደረጃ ሠራተኞችን በዕድሜ በጤና ሁኔታ ለይተን ዕረፍት እንዲወጡ አላደረግንም፡፡ ምክንያቱም የኛ ሠራተኞች እንደጤና ባለሙያ እንደጸጥታ ሀይሎች ግንባር ቀደም ተሰላፊ ሆነው ኮቪድን መከላከል አለባቸው በሚል እሳቤ ነው የተንቀሳቀስነው፡፡ በዚህ ልክ ሠራተኞቻችን ስናሰማራ የግል መከላከያ ቁሳቁሶችን እንደ እጅ ጓንት የአፍና አፍንጫ መሸፈኛ ጭንብል እና የእጅ ማጽጃ ሳኒታይዘርና ሳሙና ባግባቡ በማደል ነበር፡፡ ክልል ቢሮና ሜሪ ጆይም አግዞናል፡፡ የዞን መንግስትም ከመደበኛ በጀት ወጪ አድርጎ ገዝቶ ሰጥቶናል፡፡ ያን ያክል ልቅቅ ያለ ነገር ባይኖርም ላለው ተልዕኮ የሚበቃ ያክል ሀብት ተመድቦልን እየተንቀሳቀስን ነው፡፡

የድጋፍና ክትትል ሁኔታ በተቋም ደረጃ ምን ይመስላል?

አዎን ወደ ግብ ያመራል፤ ይህንን ስንል መገለጫው ጎዳና ተዳዳሪዎችን ወደቤተሰቦቻቸው ስንመልስ ተቀናጅተን ነው የሠራነው፡፡ በተቀናጀነው አግባብ እነኚህ የጎዳና ተዳዳሪዎች ገብተው የሚቆዩበት ፍራሽ ትራስና የመሳሰሉት የማቆያ ግብዐቶች ያስፈልጉ ነበር፤እነዚህን ሥራዎች ተጠርንፍን ነው የሠራነው፤ ምክያንቱም አንድ ተቐም ብቻውን የሚወጣው ባለመሆኑ፤ ለምሳሌ የግዥ ሥራን እንዲያግዙን ፋይናንስን ይዘን በዕድሜ ህጻናት የሆኑትን እንዲይዙ የሴቶችና ህጻናት መዋቅርን ይዘን ፡ ካምፕ የገቡት ወጥተው እንዳይሄዱ ለጥበቃ ፖሊስን ይዘን እነኚህንና የመሳሰሉትን አብረን ተናብበን ነው የሠራነው፡፡

ቅንጅታዊ ሥራ መሬት እንዲወርድና ውጤት እንድያመጣ ባለድርሻ አካላት ሃላፊነትንና ተጠያቂነትን የሚያሳይ ገዥ የሆነ ሰነድ ወይንም የአሰራር ማዕቀፍ ስለመኖር

አዎ አለን፤ ቅድም በተነጋገርንው መሠረት ገና ሥራ ከመጀመራችን አስቀድሞ ያዘጋጀነው ዕቅድ አብሮ ለመሠራት የሚገዛን ማዐቀፍ ነው፡፡ ዕቅዱን ስናዘጋጅ ማን ምን መሥራት እንደለበት በግልጽ ተቀምጠዋል፡፡ እያንዳንዱ ባለድርሻ አካል የሚሰራው ዝርዝር ተግባራት በዕቅዱ ውስጥ በዝርዝር ስለተቀመጠ እነዚህ ተግባራት ናቸው የሚያስተሳስሩን፤ እያንዳንዱን ተግባር የምናከነውንበት ቢጋር ወይንም የጋራ መግባቢያ ሰነድ ነው የሚመራን፤ ክንውኑን በዚሁ መልክ ስንሰራ የክተትልና ድጋፍ ሥራውም በተመሳሳይ መልኩ ያቀድናቸው ተግባራት በትክክል እየተገበሩ ነው ወይ የላክናቸው ቁሳቁሶች ለተገልጋይ ደርሰዋል ወይ የሚለውን ወርደን እንያለን፡፡

ይህ የግምገማ ሥርዓት በምን ያህል ጊዜና ድግግሞሽ ነበር የሚከናወነው እንዲሁም በሂደት የተገኘ ጉድለት ካለ በምን አግባብ ነው የታረመው?

ይህን ሥራ ስንሠራ ጅምር አካባቢ በንዑስ ኮሚቴ ደረጃ በየቀኑ እየተገናኘን ነበር የምንገመግመው፤ በዚህ ሂደት በምናገኘው ግኝት መሠረት ጠንካራው በጥንካሬ እንዲቀጥል እና ጉድለቱ እንዲሟላ ያደረግንበት አሰራር ነበር፡፡ ሂደቱ ጊዜ እየወሰደ ሲመጣ በየጊዜው እየተገናኘን የምንገመግምበት ሁኔታ እየቀነሰ መጥተዋል፡፡ አሁን ያለው ብቻ ይህን አድርጉ፣ ተጠንቀቁ፣ ተጠበቁ የሚል ዓይነት ሆነ፡፡ ይህ ሁኔታ በዐቢይም ሆነ ንዑሳን ኮሚቴዎች ደረጃ ቀንሰዋል፡፡ በሁሉም ደረጃ ያለው የድጋፍና ክትትል አግባብ እየተቀዛቀዘ ነው፤ በተቃራኒው ደግሞ የበሽታው ስርጭት እየጨመረ ነው፤ ይህ ተገቢ ስላልሆነ መስተካከል አለበት፡፡

የኮቪድ -19 በሽታ መከላከልና ቁጥጥር ሥራ ጋር ተያይዞ በሂደት የተገኘ መልካም አጋጣሚና ተሞክሮ ሊባል የሚችል ልምድ ካለ?

በዋናነት ከኮቪድ-19 ጋር ተያይዞ እንደመልካም አጋጣሚ ሊጠቀስ የሚችል ነገር ቢኖር ቅንጅታዊ ሥራ ጋር የተያያዘ ነው፡፡ በዚህ ሂደት ሰዎች ስለሰው ልጆች ያላቸው አመለካከትና መተሳሰብ እንዲጎልብት የማድረግ ሁኔታ በደንብ ተስተውሏል፡፡ ጤናማ ህሊናና አንዱ ለሌላው ማሰብና ተጋግዞ መኖር ጠቃሚነትን እንዲስተዋል ዕድል የፈጠረ ነው፡፡ የተለያዩና ጫፍ የረገጡ የልዩነት አመለካከቶችንና አስተሳሰቦችን ወደጎን ትተን ከምንም በላይ ክቡር ለሆነው የሰው ልጅ ህይወት ቅድሚያ እንዲንሰጥ ያደረገ ነው ማለት ይቻላል፡፡ ሌላው ለወደፊት ምን ማድረግ እንዳለብን አቅጣጫ የጠቆመ እንደሆነም ይስተዋላል፡- ለምሳሌ በርካታ መልካም የሆኑ በርካታ ግንባታዎችን ብንሰራም ሰብአዊ ፍጡር በተለይም ማህበራዊ ቀውስ ሊያስከትሉ የሚችሉት በየጎዳና የሚኖሩና ለምኖ አዳሪዎች ዛሬ ላይ ባግባቡ ካላስተናገድን ለቀጣይ ከፍተኛ የሆነ ማህበራዊ ቀውስ ሊያስከትል እንደሚችል ግንዛቤ የተወሰደበት ነው ማለት ይቻላል፡፡ ከአካል ጉዳተኞች ጋር በተያያዘ ለ2030 የተቀመጠውን የዘላቂ ልማት ግቦች እንድናስብና በርግጥም ግቡን ማሳካት እንችል እንደሆነ ካሁኑ በጥንቃቄ ማሰብ እንዳለብን ዕድል ፈጥሯል ማለት እችላለሁ፡፡በተለይ ደግሞ የማስፈጸም አቅማችን እንድንፈትሽ አድርገዋል፡፡ የሚያጋጥመንን ችግር በብዙ አቅጣጫ እንድናይና በእጃችን ያለንን በምን መልኩ አዳብረን መጠቀም እንደላብን ዓናችንን እንድነከፍት አድረጎናል፡፡

ቁልፍ መረጃ ሰጭ ዘጠኝ

የጋሞ ዞን ጤና መምሪያ

እንደዞን የጤና መምሪያ የኮቪድ-19 ወረርሽኝ በሽታ መከላከልና ቁጥጥር ሂደት ያላችሁ አጠቃላይ ቁርኝት ፡- ሌሎችን በማስተባበር በቀጥታ በመከላከል ሥራ በመሳተፍ ከላይኛውና ጣቸናው መዋቅር ጋር እንዲሁም ከጎንዮሽ መዋቅር ጋር በጋራና ቅንጅት ለመሥራት ያለው ተቋማዊ ቁመና ምን ይመስላል?

ያው እንግዲህ በሽታው ወደሀገራችን ከገባ ጊዜ ጀምሮ በተበታተነ መልኩም ቢሆን የመከላከል ሥራ ላይ አተኩረን ስንረባረብ ነበረ፡፡ በኋላ ላይ ለመከላከል የሚያስችል አደረጃጀት እንዲኖር ከክልልና ከፌዴራል የጤና መዋቅሮች በተቀመጠው አቅጣጫ መሰረት ዞናዊ አደረጃጀት በመፍጠር እንደዚሁም ደግሞ በሥሩ ንዑሳን ኮሚቴዎችን በማደራጀት ወደተግባሪዊ እንቅስቃሴ ገብተናል፡፡እንደዚን ጤና ደግሞ የተሰጠን ተልዕኮ በሽታን የመከላከልና የህክምና አገልግሎት ንዑስ ኮሚቴ ሰብሳቢ ነኝ፡፡ከዚህ አንጻር ከዩኒቨርስቲ ከኮሌጆች ጋር ከፖሊቴክኒክ ሳታላይት እንዲሁም ከሌሎች ጤና ጋር ከሚሰሩት የሥራ ዘርፎች ጋር ተቀናጅተን ስንሠራ ቆይተናል፡፡ አሁን ባለን ደረጃ በሽታውን ለመከላከልና ለመቆጣጠር በዞን ደረጃ እየተሰሩ ያሉት ሥራዎች መልካም ናቸው ማለት ይቻላል፡፡ ይሁን እንጂ ይህ አደረጃጀት በታችኛው መዋቅር በተቀመጠው አቅጣጫ መሠረት ተግባራዊ ከማድረግ አንጻር መንጠባጠብ አለ፡፡ እንደዚሁም ደግሞ አቅም ከመገንባት አንጻር ሎጂስቲክስ ከማዘጋጀት አንጻር ጉድለት እንዳለ እየገመገምን መጥተናል፡፡ እንደዞን ጥሩ እየተንቀሳቀስን ነው እንላለን፡፡

የዚህ በሽታ ጫና እንደዞን እንዴት ገመገማችሁ?

እውነት ለመናገር በሽታው ሀገር ውስጥ ከገባ በኋላ ትኩረት ሰጥተን ተንቀሳቅሰናል፤ለአብነት በሽታው በክልላችን አላባ ላይ ሲገኝ በጣም ተደናግጠናል፤ ያንን መነሻ አድርገና ህብረተሰቡ ዘንድ ያለው ግንዛቤ እጅግ ያነሰ ስለሆነ በተለይ በማህበራዊ መስተጋብሮች እንደለቅሶ ቤት ዕድር ዕቁብ ህዝብ በብዛት በሚሰበሰብባቸው ቦታዎች ገበያንም ጨምሮ ማህበራዊ መስተጋብሮች ከፍ ያለ ስለነበረ በሽታው እንዳለ ሀሰት እንዳይደለ በሚገባ ማስተማር አለብን ብለን አርአያ እንሁን ብለን፡- እኔ፣ የዞኑ ዋና አስተዳዳሪ፣ የዩኒቨርሲቲ ፕሬዚዳንትና ምክትል ፕሬዚዳንቶች የግንዛቤ ማስጨበጫ ሥራዎችን እዚህ አርባምንጭ ከተማ ላይ እንዲሁም ሰፋፊ ገበያዎች ላይ በሰፊው አስክደናል፡፡ ያው ጅምር ላይ ድንጋጤም ስለነበረና በግንዛቤ ማስጨበጥ ሥራ ላይ ብዙም ስላልሰራን ወደፊት ወጥተን ህዝብን እናንቀሳቅስ ብለን በዚያ ደረጃ ነው የሰራነው፡፡ በተዋረድ ይህ ሥራ በወረዳ፣ ከተማ አስተዳደር አንዲሁም በቀበሌዎች በአስተዳዳሪዎች፣ በከንቲባዎችና በሊቃነመናብርት በኩል መልእክት እንዲተላለፍ ተደርገዋል፡፡በዚያ መልኩ እየሰራን ቆይተዋልና፤ ከጫና አንጻር መንቀሳቀስ በሚገባን ልክ ወይንም መውሰድ በሚገባን እርምጃ ልክ ነው ማለት አይቻልም፡፡ጫናው እየከበደ ሲመጣ ግን የበለጠ መቀዛቀዝ እየታየ ነው ያለው እንደኛ ግምገማ፡፡ በተለይ ባለፉት ሶስት ሳምንታት ተቀዛቅዘናል፡፡በተለያዩ አጀንዳዎች ተወጥረን ወደወረዳዎች አካባቢ ሌሎች የኛን ቀልብ የወሰዱ ጉዳዮች ነበሩና እዚያ ላይ በመወሰድ፤ ያ ሆኖ ሲያበቃ በሽታው በዞናችንም እንዲሁም በክልላችን በተለያዩ ዞኖች በሚያሰፈራ ፍጥነት ከመከሰትም በላይ እንዳንዱን ቤት በር ለማንኳኳት የተዘጋጀ ይመስላል፡፡ጫናው ከፍ ያለ ነው፤ በሌላ ጎን ደግሞ ትኩረት የተቀዛቀዘ መሆኑን አሁን ባለን ደረጃ ገምግመናል፡፡

በሽታውን ለመከላከልና ለመቆጣጠር በሚደረግው ሂደት የባለድርሻዎች ተሳትፎ በዞን ደረጃ እንዴት ይታያል?

አሁን ያለው ቅንጅታዊ አሰራር ከበሽታው ጫና ጋር የሚጣጣም አይደለም የሚል ግምገማ ነው ያለኝ፡፡ እውነት ለማውራት ከላይ ከክልል ጀምሮ መቀዛቀዝ አለ የምል እምነት ነው ያለኝ፡፡ትናንትና የቪዲዮ ኮንፈራንስ በነበረን ይህን ገምግመናል፡፡ሌሎች ዞኖችና የክልል ግብረ ሀይልም ይህንን ገልጸዋል፡፡እኛጋም በዚ መልኩ ነው ፤ በተጀመረው ግሌት አለቀጠለም፤

ለዚህ መቀዛቀዝ እንደምክንያት የሚነሳው ምንድነው?

ምክንያት የሚሆነው የትኩረት ማነስና አመራሩ በተለያዩ አጀንዳዎች መወጠር ነው፤አንዱ በበጀት ማጠቃለያ ላይ በመሆናችን ሁሉም በሴክቶሪያል አጀንዳዎች በማተኮር መረባረብ አለ፤ ከላይ ወደታች የሚወርዱትን አጀንዳዎች የማስፈጸምና ተልእኮ የመከፋፈል ጉዳይ አለ፤አመራሩ ወደታችኛው መዋቅር ያለው ፍሰት እዚህ ያለውን ሥራ እንደቀድሞ ጊዜውን በጠበቀ መልኩ እንዳንከታተል አድርጎታል፡፡ከዚህ በፊት በሳምንት ሶስት ጊዜ በግብረ ሀይል ደረጃ እንገመግም ነበር፤ እያንዳንዱ ንዑስ ኮሚቴ ያከናወነውን ተግባራትና በጉድለት የታዩትን ገምግመን ማሻሻያ አቅጣጫ እናስቀምጥ ነበር፡፡ከዚያ ጥሩ ግስጋሰን እያየን ነበር እውነቱን ለማውራት፤ለምሳሌ፡- አስቸኳይ ጊዜ አዋጅ ከማስፈጸም አንጻር በተለይም ከትራንስፖርት ስምሪት የምግብ ቤቶችን ከመቆጣጠር አንጻር፤ሆቴሎች አካባቢ የሚታዩትን ዳተኝነት ቶሎ ከማረም አንጻር በትራንስፖርት ስምሪት አካባቢ የነበሩትን ችግሮችን ከመፍታት አንጻር ጥሩ ሥራዎችን እንደሠራን አስተውላለሁ፡፡ መቀዛቀዙ ባሁኑ ጊዜ ቅድም ባልኩት ልክ አለ፤ በተለይ የፊት አመራሩ ቀልቡ በሌላ አጀንዳ በመያዙ ነው፡፡ ነገር ግን በሽታው እየሰፋና እየባሰ እየመጣ ነው፡፡ ስለሆነም አሁን መንቃት አለብን በሚል ነው የሚገመግመው፡፡

እንደዞን የጤና መምሪያ የኮቪድ-19 በሽታ ምላሽ እየሰጣችሁ እንደሆነ እንገነዘባለን፤ ለዚህ መነሻ የሆነ መመሪያ ደንብ አዋጅ እንዲሁም ዕቅድ ስለመኖሩ

ከጅምር አንስቶ እንደዞን ጤና መምሪያ በንቃት እየሰራን እንገኛለን፤ እውነትን ለማውራት፤ ከጤና ሚኒስቴር የወረደ አደረጃጀት ስለነበረ ኢኦሲ(EOC) አደረጃጀትና መደበኛ የጤና ስራ የምናስኬድበት፡፡ ሁለት አደረጃጀቶች አሉ፤ አንደኛው ኢኦሲ(EOC) የሚባለው የኮቪድ-19 መከላከል ሥራ ላይ ብቻ አተኩሮ የሚሰራ ሲሆን ሁለተኛው ደግሞ ህክምና ላይ የሚሰራ ሆኖ የሚያካትተው የኳራንታይን ማዕከል አይሶሌሽን ማዕከልና ና የህክምና ማዕከል ላይ የሚሰራ ነው፡፡ እነዚህ ቡድኖች ሌላ መደበኛ የጤና ሥራ ላይ አይሳተፉም፡፡ ተግባሩ ሁሉ ይህ ሆኖ ተመሳሳይ አደረጃጀት ወደወረዳም ወርደዋል፡፡መደበኛ የጤና አገልግሎትን የሚያስኬድ ቡድን ሃላፊነት ሰይመን ሰጥተናል፡፡ ኢኦሲ ላይ ኢንሲዴንት ማናጀር ሰይመናል በዚሁ ሥር ደግሞ ኦፕሬሽን ማናጀር ሰርቨይላናስ ሊደር እና ኳራንታይን ሊድረ ብለን ስራውን የሚመሩ ሃላፊዎችን መድበናል፤እነዚህ በርካታ አደረጃጀቶች መደበኛ ፕሮግራማቸው ይህ ነው፡፡ ስለዚህ ጋይድላይንና ተግባሮችን የምንመራባቸው የአሰራር ማኑዋሎች አሉን፡፡ዕቅዶችም አሉን በዚያ መሰረት እየሰራን እንገኛለን በዞን ጤና መምሪያ፡፡ ነገር ግን ወደታች ስንወርድ በዚህ ልክ እየሄደ ነው ማለት ድፍረት የለንም፡፡ምክንቱም መንጠባጠቦች ስለሚኖሩ ማለት ነው፡፡

እንደዞን ጤና መምሪያ እንደአሰራር የተዋቀሩ አደረጃጀቶች ከበሽታ ቅኝት ጀምሮ እስከህክምና ድረስ ያሉት ቡድኖች ተናቦና ተቀናጅተው ከመስራት አንጻር በየደረጃው የተመደቡት ባለሙዎች ራሳቸውን ጠብቀው ለተልዕኮ እንዲሰማሩ በማድረግ ረገድ በቂ የግል መከላከያ ቁሳቁስ ከማቅረብ አኳያ በምን ደረጃ ላይ ናችሁ?

ይህ በኢኦሲ ስር የተዋቀሩት ቡድኖች ሥራቸውን በየቀኑ ይገመግማሉ፤ አሁን በዚህ ሰዓት ግምገማ ላይ ናቸው፡፡ግምገማቸው የሚያተኩረው በየወረዳ ያለው የበሽታ ቅኝት ቡድን ሥራ አፈጻጸም ምን ይመስላል? ስንት ተጠርጣሪ ተለየ? ስንት የማህበርሰብ ሰርቨይላንስ ስራ ተሰራ? ስንት ቤት ተደራሽ አደረጉ? ምን የውዥንብር ወረዎች ተሰሙ? ምን የሚያነቁ ክንውኖች ተገኙ? በማለት በየቀኑ ይገመገማሉ፡፡ ለምሳሌ ኳራንታይን ላይ ስንት ሰዎች አሉ? ምን ምልክት ያሳያል? እዚያ ላይ ሎጂስቲክስ ተሟላ ወይ አልተሟላም? የምግብ አቅርቦትስ እንዴት ነው? ምን ችግር ተከሰተ? ብሎ የሚገመግም ቡድን አለ፡፡ እንደዚሁ ደግሞ አይሶሌሽን ማዕከል ላይ፡ አይሶሌሽን ማዕከል አልተሟላም ነበር፤ለምሳሌ የኢሌክትርክ መስመር ዝርጋታ የውሀ መስመር ዝርጋታ ያልተሟሉ ነበሩ፤ እነዚህን ጉድለቶች እጅግ ባጠረ ጊዜ ለማሟላት ምን ስልት ብንከተል ይሻላል የሚለውን ባጠቃላይ በኢኦሲ ስትራክቸር ውስጥ ያሉትን ሥራ ሂደቶችን በየቀኑ ይገመገማል፡፡ በህክምና ማዕከል አንድ ህመምተኛ አለች አሁን፤ ለዚህም ህክምና አገልግሎት የሚሰጡ ባለሙያዎችን ከተለያየ የሙያ ዓይነት በሁሉአቀፍ ሁኔታ በስነልቦናም ከማዘጋጀት አንጻር የግብዐት አቅርቦትን ጨምሮ ሁሉም ይገመገማል፡፡ ከግብኣት አቅርቦት አንጻር በተለይ የግል መከላከያ ቁሳቁስ ጉድለቶች አሉ፤ ግን አሁን ሥራ እንዳንሠራ በሚከለክል ደረጃ አይደለም፡፡ ሆኖም ግን ይህ ሁኔታ በሽታውን ሙሉ በሙሉ ቁጥጥር ስር እስኪናውል ያለመንጠባጠብ መሟላት ያስፈልጋል፤ አሁን በጀት ከዞኑ ቋት እየመደብን እየሰራን እንገኛለን፡፡ በሂደት የበጀት ችግር ሊገጠመን እንደሚችል ስጋት ቢኖረንም ከክልል ጤና ቢሮ ጋር በመመካከር አሁን ሊያሰራ የሚችል የግል መከላከያ ቁሳቁሶች በእጃችን አሉ፡፡ሌሎች የህክምና ቁሳቁሶች ፡- አልጋ መድሃኒት ና የህክምና መገልገያ ዕቃዎችን ከአርባምንጭ ዩኒቨርስቲ ድጋፍ እያገኘን ነን፡፡ ምንም እንከዋን ከግብኣት አንጻር መንጠባጠብ ቢኖርም አሁን ሊያሰራን በሚችል ቁመና ላይ ነን፡፡ነገር ግን mechanical ventilatorna ICU ዕቃዎችንን ከማሟላት አኳያ ጉድለቶች አሉ፡፡ የኦክስጂን አቅርቦትን በሚመለከት የክልል ጤና ቢሮ አስሞልቶ ይልክልናል፡ ከኛ የሚጠበቀው ሰልንደሩን አጓጓዘን ወደክልል መላክ ብቻ ነው፡፡ሆኖም ግን በዞን ደረጃ ያሉትን የኦክስጂን ሰለንደሮችን ለመላክ እየሰበሰብን ነው፡፡

በርካታ ሥራዎች በኢኦሲ ደረጃ በየቀኑ እየተገመገመ እንደሚሰራ ተረድተናል፤ ነገር ግን በመምሪያ ደረጃ በምን ያህል ጊዜ ነው ስራው የሚገመገመው?

በየቀኑ ለኔ በተሌግራም ይላክልኛል፤ለምሳሌ ትናትና በቀን 10/10/2012 ዓ.ም. የውሎ ግምገማ ብሎ በዳራማሎ ወረዳ የታዩት ጉድለቶች ተብሎ ከቅኝት ሥራ በኳራንትን ውስጥ ያሉትን ተጠርጣሪዎችን መረጃ አሟልቶ ይዞ ከማስተላለፍ ረገድ ጋር ተያይዞ የታየው ክፍተት ተደጋጋሚ ስለሆነ የርስዎን ትኩረት ይሻል፤ ሌሎች ወረዳዎች የተሻሉ ናቸው ተብሎ በተሌግራም ተልኮልኛል፡፡ እኔ ደግሞ ይህን መነሻ አድርጌ ቀጥታ ለዳራማሎ ወረዳ አስተዳዳሪ ነው የሚደውለው፤የናነተ ኦኦሲ ፋንክሽናል አይደልም፤ መረጃ እየሰጠ አይደለም፤ መረጃ ሕይወት ነው ግን የበሽታ ቅኝት ሥራ የተጠናከረ አይደልም፤ የናንተን ትኩረት ይሻል ብዬ እዚያው ግብረመልስ እሰጠዋለሁ፡፡ እርሱ ደግሞ ይህንን ወስደው ለወረዳ ጤና ጽ/ቤትና ለግብረ ሀይል አቅርቦ ከገመገመ በኋላ መልሶ ለኔ ግብረ መልስ ይልክልኛል፡፡ በዚህ መልኩ የዕለት ተዕለት መረጃው ይደርሰናል፡፡ እኔም ደግሞ ለዞኑ ዋና አስተዳዳሪ በየዕለቱ መረጃ እሰጣለሁ፡፡ሌላው ደግሞ ከግብኣትና ፋይናንስ አንጻር ክፍተት ከተፈጠረ ራሴ ጣልቃ ገብቼ እፈታለሁ እንጂ ለዋና አስተዳዳሪ አላቀርብም፤ ምክንቱም ይህ የእኔ ሃላፊነት ስለሆነ ነው፡፡ በእኔ ደረጃ ሊፈታ ካልቻለ ለዋና አስተዳዳሪ አቅርቤ በጋራ ተወያይተን ካቅማችን በላይ ከሆነ ከዪኒቨርሲቲ ጋር ተመካክረን እንፈታለን፡፡ዩኒቨርሲቲው ይተባበረናል፡ በዚህ መልኩ ችግሮቻችን እየፈታን መጥተናል፡፡

ከግብኣት አቅርቦትና ፋይናንስ አኳያ ማነቆ እንዳይሆንብን ለምሳሌ በቅርቡ የልብስ ንጽህና መስጫ ማሽን ግዥን በሚመለከት ተቸግሬን በአንድ ቀን አዝዘን አስገዝተናል፡፡ጄኔሬተርም እንደዚሁ በአንድ ቀን ገዝተናል፡፡ግዥን ባስቸኳይ እንድንፈጽም የሚያግዝ ለዚህ ጊዜ የሚያግዝ የግዥ መመሪያ ተዘጋጅተው በመላኩ ብዙ የግዥ ማነቆዎችን ፈትተዋል፡፡

እንደዞን እስካሁን የመጣንባቸው መንገድና ትግበራ ወደታለመለት ግብ እያደረሰ ስለመሆኑ ማረጋገጫችሁ ምንድነው? እንዴትስ ታረጋግጣላችሁ?

ከጤና ሴክተር አንጻር የከፋ ጫፍ ሊሆን ይችላል ብለን ወይንም አደገኛ አዝማሚያ ቢመጣ ምን ያክል ሰው ሊጠቃ ይችላል ምን ያክል ሰው ፅኑ ህሙማን ማከሚያ ይገባል ምን ያክል ስው ሜካኒካል ቨንቲሌተር ይፈልጋል ምን ያክሉ ደግሞ በሽታው ሳይጸና ሊያገግም ይችላል ብለን በዚያ ደረጃ አቅደን እየተንቀሳቀስን እንገኛለን፡፡ አሁን ላይ ሆኜ ሳስበው ግን በጣም ብዙ መስራት እንዳለብን ነው የሚሰማኝ፡፡ለምሳሌ አሁን ለህክምና መስጫ ያዘጋጀነው ማዕከል 80 አልጋ ብቻ ያለው ነው፡፡ ግን የበሽታው ስርጭት በ80 ያበቃል ወይ ብለን ከፍ ላለ ቁጥር መዘጋጀት አለብን፡፡ ምናልባት እንደሚሊኒየም አዳራሽ እንኳን ባይሆን የተሻለ አቅም ያለውን ቦታ ማዘጋጀት ያስፈልጋል፡፡ከዚህ አንጻር እምብዛም አልሰራንም ግን በቀጣይ መስራት እንዳለብን ይሰማናል፡፡አሁን ባለው መቀዛቀዝ የምንቀጥል ከሆነ ዝም ብለን ለይስሙላ እንደምንሰራ ነው፡፡ስለዚህ አሰቀድመንና አርቀን ማሰብ ያስፈልጋል ብዬ አምናለሁ፡፡ ለአብነት አሁን ዪኒቨርስቲ ውስጥ ያሉትን አዳራሾች ለመጠቀም ማቀድና ክፍፍል ሰርተን ለመጠቀም ከዩኒቨርስቲ ፕሬዚዳንቶች ጋር ተመካከረናል፡፡ ሁሉም ባላድርሻ አካላት ለሰፊ ተግዳሮትን (woreste case senario) መዘጋጀት ያስፈልጋል ብለን አቅደን እየተንቀሳቀስን እንገኛለን፡፡ ወደምናስበው ግብ አሁን ባለን ቁመና ለመድረስ እንቸገራለን፡፡ስለዚህ ቶሎ ብለን ማረም አለብን የሚል አቋም አለኝ፡፡

ከኮቪድ በሽታ ምላሽ ጋር ተያይዞ የታየ ወይንም የተገኘ አዎንታዊ ጎን ካለ ቢገለጽ፡-

አንዱ ኮቪድ በሽታ ወደ ሀገራችን ከገባ ጀምሮ መደበኛ የጤና አገልግሎት መቀዛቀዝ ታይቷል፡፡ሆኖም ግን እንደመልካም አጋጣሚ ሊጠቀስ የሚችል፡- የቤት ለቤት ቅኝት ሥራ መጀመሩ ለሌሎች በሽታዎች ክትትል ጠቃሚ እንደሆነና ከህብረተስብ ጤና ቅኝትና ምላሽ ስራ ጋር ማያያዝ እንደሚቻል በዚህም ሌሎች በሽታዎችን እንደ ወባ ኩፍኝና ሚዝል ማካተት እንደሚቻል እና ይህንን እንደመልካም አጋጣሚ ማነሳት ይቻላል፡፡ ሌላው የእጅ መታጠብ ባህላችን ጨምረዋል፡ ከዚህ ጋር ተያይዞ ተላላፊ በሽታዎች እን ጉንፋን እና የመሳሰሉት በሽታዎች መቀነሳቸውን ታዝበያለሁ፡፡ ሌላው ወደህብረተሰብ ተደራሽ ማድረግ ሥራ ጥሩ ተሞክሮ የተወሰደበት ነው ማለት ይቻላል፡፡ አሁን ካለው የሚዝል ዘመቻ ጋር የቤት ለቤት ቅኝት ማገናኘት ሁለተኛ ዙር ዘመቻ ለማጠናከር በርካታ ጥቅም ይኖረዋል፡፡

ሌላው ሊጠቀስ የሚችለው የባለድርሻ አካላት ጉዳይ ነው፡፡ይህንን ኮቪድ-19 በሽታ ወረርሽኝን ምላሽ ለመስጠት የተፈጠረው አብሮ መስራት (ቅንጅታዊ ስራ) ለሌሎች ስራችንም ጥሩ ተሞክሮ ያገኘንበት ነው፡፡ አንድ ላይ አቅደን አንድ ላይ ተግብረን አንድ ላይ ግምግመን ውጤት የምናይበት ዕድል ፈጥሮአል፡፡

የጤና መረጃ ሥርዓትን ከማጠናከርና አዳዲስ አሰራርን ከመፍጠር አኳያስ?

ይህ ቭረቹዋል ስብሰባ የተጀመረው በዚህ ወቅት ነው፡፡ ከላይ ከጠ/ሚ/ር ጀምሮ እኛ አሁን ከክልሉ ር/መስተዳድር እና የጤና ቢሮ ሃላፊ ጋር በቪዲዮ ኮንፈራንስ ነው የምንገናኘው፡፡ ስለዚህ ይህን አሰራር ለሌሎች ስራዎቻችን መጠቀም እንችላለን፤ በአካል መሰብሰብ ሳያስፈልግ በሞባይል አፕሊኬሽን በመጫን ብቻ ስራዎችን መስራት እንደሚቻል ያየንበት ሁኔታ ነው ያለው፡፡ የትም ሆነን ስራ መስራት እንደሚቻል እና ለተለያዩ ማህበራዊ ኢኮኖሚያዊ እና ፖለቲካዊ ስራዎችም መጠቀም እንደሚቻል ተሞክሮ የተወሰደበት ነው፡፡ ይህ ልምድ በጤናው ዘርፍ የተጀመሩትን በቴክኖሎጂ የሚታገዙትን ሥራዎችን በፍጥነት ከመተግበር አኳያ የተሻለ ልምድ የተገኘበት እንደመሆኑ ለምሳሌ እንደማህበረ ሰብ የጤና መረጃ ሥራም ላይ የራሱ የሆነ አዎነታዊ አሻራ እንደሚያሳርፍ ይጠበቃል፡፡

እንደባለድርሻ አካል የአርባምንጭ ዩኒቨርሲቲ አስተዋጽኦ ትንሽ ቢብራራ

የአርባምንጭ ዩኒቨርስቲ የአመራር አካላትን ጨምሮ ከጅምሩ አንስቶ በሽታን በመከላከል ሂደት የህዝቡን ንቃተ-ህሊና ከማሳደግ አንጻር በሁሉም ዩኒቨርሲቲ በሚያካቲታቸው አካባቢዎች ዞሮ ገበያን ጨምሮ በሞነታሪቦ ጭምር ተገቢ መረጃ ለህዝቡ የማድረስ ሥራ ላይ ተሳትፈዋል፡፡ሁሉንም የዞናችን ወረዳዎች እና ኮንሶን ደራሼንና አሌን ጨምሮ ጎፋንም ጨምሮ በተደራጀ መልኩ ይሰራሉ፡፡ሁለተኛ በዐቢይና ንኡሳን ኮሚቴዎች በንቃት ያሳተፋሉ፡፡ሶስተኛ ከግብዓት አቅርቦት አንጻር በደንብ በመቀናጀት እየሰራን እንገኛለን፡፡ ለምሳሌ የምርመራ ላቦራቶሪ እስከማደራጀት ድረስ ዋጋ ከፍለዋል፡፡እንደመደበኛ ስራቸው በቁርጠኝነትና በተነሳሽነት መንፈስ ሆነው ነው የሚሰሩት፡፡የህክምና ግብዓቶችን ከማሟላት አንጻር አልጋ ሙሉ በሙሉ እነርሱ ናቸው ያሟሉት፡፡ኳራንታይንና የአይሶሌሽን ማዕከል የዩኒቨርስቲ ነው፡፡ስለዚህ አብዛኛውን ሥራ ከነርሱ ጋር ተቀናጅተን ነው የሰራነው፤ በመሆኑም የተሰጡ ናቸው ማለት ይቻላል፡፡ሌሎች የሚጓደሉ ነገሮችን ለማሟላት ዝግጁ ናቸው፡፡ከባለሙያ ትብብርም አንጻር በርካታ ሲኒየር ሀኪሞችና የጤና ባለሙያዎች ከሰርቬይላንስ ማዕከል ጀምሮ እስከህክምና ድረስ ተመድበው እየሰሩ ይገኛሉ፡፡ስለዚህ ዩኒቨርስቲው እያደረገ ስላለው የላቀ አስተዋጽኦ ልመሰገን ይገባል፡፡

| **S.No.** | **Institution of the Key Informant** | **Age** | **Sex** | **Position** | **Year of service on the position** | **Education** |
| --- | --- | --- | --- | --- | --- | --- |
| 1 | Ethiopia Red Cross Society, SNNPR Branch Office |  | M | Regional Managers | 3 | MA |
| 2 | Regional Teachers Association |  | M | Chairman |  | MA |
| 3 | WCYA Bureau |  | F | Head | 2 | MA |
| 4 | Education Bureau |  | M | Head | 2 | PhD |
| 5 | Finance Bureau |  | M | Head | 2 | Msc |
| 6 | South Radio & TV |  | M | Head | 4 | MA |
| 7 | Agri & NR Bureau |  | M | Head | 1 | MSc |
| 8 | Health Bureau |  | M | Head | 2 | MHA |
| 9 | Transport and Road Dev. Bureau |  | M | Head | 2 | MA |
| 10 | Water, Energy Bureau |  | M | Acting Head |  |  |

የክልል ማዕከል ቁልፍ መረጃ ትራንስክሪፕሽን

ቁልፍ መረጃ ቁጥር 1. ቀይ መስቀል ደቡብ ቅርንጫፍ

የኮቪድ-19 በሽታ ለመከላከልና ቁጥጥር ሥራ ሂደት የቀይ መስቀል ማህበር ደቡብ ኢትዮጵያ ቅርንጫፍ ያለው ቁርኝት ምን ይመስላል?

በነገራችን ላይ የኢትዮጵያ ቀይ መስቀል ማህበር ሲመሰረትም ጀምሮ ህዝብንና ሀገርን የሚፈታተኑ በውስጥም ሆነ በውጭ ሰው ሰራሽም ሆነ ተፈጥሮአዊ ሲደመር ደግሞ በአስቸኳይ ጊዜ አዋጅ የሚታገዙ ወረርሽን ዓይነት በሽታዎች ሲከሰቱ ቀድሞ ፊት ለፊት ወጥቶ ህዝብን በማስተባበር አባል ሀገራቱንም ጭምር ልክ አሁን ኮቪድ-19 በህዝብ ከሚያስከትለው አሉታዊ ተፅዕኖ አኳያ የሌሎች ሀገሮችንም ድጋፍ እንደሚጠይቅ የሌሎች እህት ማህበራትንም ድጋፍ በማስተባበር ለመስራት ነው ከሌሎች በተለየ መልኩ የተቋቋመ ተቋም ነው ቀይ መስቀል፡፡በኢትዮጵያ ውስጥም ይህ ማህበር በተመሳሳይ መልኩ እንዲሰራ ፈቃድ የተሰጠው ተቋም ነው፡፡በመሆኑም ኮቪድ-19 በዓለም የጤና ድርጅት ዓለምአቀፍ ወረርሽኝ እንደሆነ መግለጫ ከተሰጠ ጊዜ ማግስት ጀምሮ ነው ወደ ሥራ የተገባው፡፡ትልቁ ፈውስ ወይንም መድሃኒት ነው የተባለው የህዝቡን ግንዛቤ ማሳደግ እንደመሆኑ ከዚህ ጋር ተያያዥ አቅርቦቶችን ማመቻቸት ስለሆነ ድርጅቱ መደበኛ ስራውንም ቢሆን የሚያከናውነው በርካታ በጀት መድቦ ሳይሆን ግን በጎ ፈቃደኞችን በማሰማራት እንደመሆኑ በእጁ ያሉትን በጎ ፈቃደኞችን ወደህብረተሰብ በማሰማራት የግንዛቤ ማስጨበጥ ስልጠና በመስጠት ነው የጀመረው፡፡ በክልሉ ሁሉም ዞኖችና ልዩ ወረዳዎች ያሉትን የጤና ተቋማትን በመጠቀም ነው ሥራዎችን የሰራነው፡፡ ልክ እሱን ሥራ ላይ እያዋልን እያለ የኢትዮጵያ ጤና ሚኒሰቴር የህብረተሰቡን ግንዛቤ በማሳደግ ረገድ ከነበረው የረጅም ጊዜ የበጎ ፈቃድ አገልግሎት አንጻር የኢትዮጵያ ቀይ መስቀል ማህበር አመራር ሃላፊነት እንዲወስድ ባለው መሠረት የትምህርትና ቅስቀሳ ሥራ ሃላፊነት የተሰጠን፡፡ ይህን ተደራቢ ሃላፊነት ይዘን ነው ወደሥራ የገባነው፡፡በዚህ ሂደት ላይ እያለን የክልላችን መንግስት ልክ የጤና ሚኒስቴር በሄደበት መንገድ ተመሳሳይ ዓላማ ያላቸውና ብዙ አባላት ያላቸው እንደመምህራን ማህበር የሴቶች ፌደሬሽን የስፖርት ኮሚሽን የወጣቶች ፌደሪሽን እና 12 የክልል መስሪያ ቤቶች የታቀፉበት የበጎ ፈቃድና የሀብት አሰባሰብ ግብረ ሀይል ተቋቋሟል፡፡ ይህን ንዑስ ግብረ ሀይል ካለው ልምድ አንጻር የክልሉ ቀይ መስቀል እንዲመራ ሀላፊነት ተሰጥቷል፡፡እንግዲህ ይህንን ሃላፊነት ወስደን በቀጥታ ለዚህ ግብረ ሀይል አስቀድሞ ከጤና ሚኒስቴር በኩል ለኛ የተሰጠውን ስልጠና ለአባላቱ ነው የሰጠነው፡፡ከዚህ በኋላ ይህ ግብረ ሀይል በየሥሩ ላሉት ግንዛቤ በማስጨነጥ ነው ወደሥራ የተሰማራው፡፡ እንግዲህ በዚህ ሶስት ኮሚቴዎች አሉት፡- በክቡር ር/መስተዳድር የሚመራው ዐቢይ ኮሚቴ አለ፣ ለርሳቸው ተጠሪ የሆነ በአቶ ጥላሁን የሚመራው ንዑስ ኮሚቴ አለ፣ የሀብት ልማትና የበጎ ፈቃደኞች ኮሚቴ ቴክኒክ ኮሚቴ ሲሆን የኛ ኮሚቴ ተጠሪነቱ ለክቡር አቶ ጥላሁን ነው፡፡ እርሳቸው ሁለቱን ኮሚቴ ይጠረንፋሉ፡፡ እንግዲህ የኛ በኛ ደረጃ እነዚህን ሂደቶች አልፎ ነው የመጣው፡፡ በዚህ ሂደት ላይ የመጀመሪያ ዕቅዳችን በመጀመሪያ የጠቀስናቸውን ተቋማት የየራሳቸው ማህበራዊ መሰረት ስላላቸው፡ እና መደበኛ አባላችን 2.7 ሚሊየን አባላት አሉን ይህን አባል ተደራሽ ማድረግ ቢቻል ወደበጎ ፈቃደኞች ሲመጣ 5017 በጎ ፈቃደኞች አሉ፤ በየትኛውም ጠርዝ ለሚያጋጥሙን አደጋዎች ተወርዉረው በፍጥነት መድረስ የሚችሉ የሠለጠኑና የአደጋ መከላከያ ልብስ ሁሉ ያላቸው፡፡ስለዚህ ይህንን ሀይል እኛ ብንጠቀም መምህራን ማህበር ደግሞ ከ144000 በላይ አባላት አሏቸው፤ ይሄን ብንጠቀም፤ ወደ 44 ስፖርት ፌደሬሽን አሉ፤ የስፖርት ኮሚሽን ኮሚሽነር የዚህ ንዑስ ኮሚቴ አባል ናቸው፡፡ እንግዲህ የኮሚቴ ሰብሳቢ እኔ ነኝ፤ የክልሉ ሴቶች ህጻናትና ወጣቶች ጉዳይ ቢሮ የወጣቶች ዘርፍ ምክትል ሀላፊ የዚህ ኮሚቴ ምክትል ሰብሳቢ ነው፡፡ የክልሉ ወጣቶች መዋቅር በዚህ ተደራሽ እንደርጋለን፤ ሴቶችንም ደግሞ በሴቶች የኮሚቴ አባል በኩል፤ የወጣቶች ዘርፍ በስሩ ቅድም የተጠቀሱትን አደረጃጀቶችን የያዘ በመሆኑ እነዚህን ሃይሎች ሁሉ ከተጠቀምን እስከ 500000 በጎፈቃደኞችን ማሰማራት እንደምንችል ታሳቢ በማድረግ ነው፡፡ ይህንን ሁሉ አደረጃጀት በሚገባ ፈትሸን ከተጠቀምን ከዚህ ውሥጥ ሀገሩንና ህዝቡን የሚከላከል 500000 ሺህ የሚሆኑ በጎ ፈቃደኛ አባላትን ማሰማራት እንችላለን ብለን አቅደን ነው የተነሳነው፡፡ 500000 ካቀድነው ውስጥ 368000 በጎ ፈቃደኞች ባሁኑ ሰኣት ሥራ ላይ ናቸው፡፡

ቅንጅታዊ አሰራሩ ይህን ይመስላል፡፡በዚህ ደረጃ ከተቋማት ጋር ቁርኝት ፈጠርን እየተንቀሳቀስን እንገኛለን፡፡እሱ ብቻ አይደለም እነዚህን ተመሳሳይ መዋቅር በ25ቱም ዞኖች አሉ የሀዋሳ ከተማን ጨምሮ፡፡ በየሳምንቱ ኣርብ የቪዲዮ ኮንፈራንስ እያደረግን ሳምንታዊ አፈጻጸማችን እንገመግማለን፡፡ በሂደት አንዱ ከሌላው ልምድ የምናገኝበት ነው፡፡ከኛ አቅም ከፍ ያሉና ስትራቴጂክ የሆኑና የበላይ አመራር ውሳኔ የሚሹ ጉዳዮች ተዘርዝረው ለነርሱ ይቀርባሉ፡፡ለሚቀጥለው መድረክ ከበላይ አመራሩ የሚሰጡትን አቅጣጫዎች ይዘን እንገናኛለን፡፡ ይህ ሁኔታ ከተፈጠረ ጊዜ ጀምሮ እስከአሁን ድረስና የሚቀጥል ቪዲዮ ኮንፈራንስ አለን፡፡በዚህ ንዑስ ኮሚቴ ያሉት የየተቋማት ሃላፊዎች እንደመሆናቸው የኮቪድን ስራ ከመደበኛ ሥራቸው ጋር በማቀናጀት ወደመስክ ወርደው ሁሉ ያከናውናሉ፤ ገምግሞ አቅጣጫ አስቀምጦ ይመለሳሉ፡፡የተቸገሩት ችግሮች ካሉም ፈትተው ይመለሳሉ፤ ትልቅ ችግር ሆኖ የሰነበተው እነዚህ በቀላል ሊበላሹ የሚችሉ የንጽህና መስጫ መሳሪዎች ሜድካል ፋሲሊቲዎች ተሰብስበው በተወሰነ ቦታ ተቀምጠው የቆየውን ሄደው መፍትሔ ሰጥተዋል፡፡ በቪዲዮ ኮንፈራንስ በምናደርገው ውይይት በርካታ ትግሮችን መፍትሔ እየሰጠን መጥተናል፡፡

እሱ ብቻ አይደለም በኋላ ትጎበኙታላችሁ፡በዚህ አጋጣሚ ኮቪድ የወለዳቸው ትላልቅ ዕድሎችም አሉን፤ ቅንጅታዊ አሰራራችን እያጠናከረ መጥቷል፡፡ በዚህ አጋጣሚ በደንብ ተቀናጅተን የምንሰራበት መሠረት እየጣልን ነው ማለት ይቻላል፡፡

ይህ ቅንጅታዊ አሠራር እንዴት እንደተጀመረና አሁን ያለው አፈጻጸም መነሻ ምን እንደሆነ በጋራ የማቀድ የመምራትና መገምገም ጉዳይ፡-

በነገራችን ላይ ሀገራዊ የድጋፍና ክትትል ቡድን ሁለት ጊዜ መጥተዋል፤በግብረመልሳቸው ለሌሎችም ክልሎች ሞደል የሚሆን ተሞክሮ እንዳገኙት ነው የገለጹልን፤ እንደመገለጫ ክልላዊ ዕቅድ መኖሩና ወጥ የሆነ ስትራክቸር ደግሞ አለን፤ ደቡብ ላይ የበጎ ፈቃደኞችና ሀብት አስተዳደር ንዑስ ኮሚቴ እዚህ ያዘጋጀነውን ዕቅድ ወደ ታች ማለትም ለዞንና ልዩ ወረዳ አውርደናል፡፡ ዞኖች ደግሞ በተራቸው ወደወረዳ አውርደዋል፡፡ ይህ ሰነድ ነው ትግበራችን የሚመራው፤ ሪፖርትንም በዚህ መነሻ ነው ተከታትለን የምንወስደው፡፡ በዚህ ሪፖርት የምናየው እያንዳንዱ ዞንና ልዩ ወረዳ ምን ያህል በተገቢው መንገድ ፈጽመዋል የትኛው ዝቅ ያለ አፈጻጸም ላይ ነው የሚለውን እናያለን፡፡እነዚህ ስምሪት ላይ ያሉት በየጊዝው አፈጻጸማቸውን የሚጋሩበትና የሚገማገሙበት የጋራ ቴሌግራም ቻናል አለ፡፡ በዚህም ሥራውን በየቀኑ አንዱ የሌላውን ተደራሽ አድርገው የሚወያዩበት ሁኔታ አለ፡፡የ25ቱም መዋቅር እኔጋ በቀን ይደርሰኛል፡፡ ይህም ሥራችንን በጋራ የምንለካበትና ተመሳሳይ አካሔድ ያለበት ነው፡፡ ለምሳሌ አሁን እኛ እዚህ እየተወያየን አንድ ሰው አንዱን ዞን ሄደው ቢጎበኝ ተመሳሳይ ነገር ያገኛል፡፡ እንበል ጉራጌ ዞን ቤንች ሸኮ ዞን እንዲሁም ደቡብ ኦሞ ብትሄዱ ተመሳሳይ ትግበራ ታያላችሁ፡፡ ይህም ሁሌ ስለምንገናኝና ስለምንወያይ ነው፡፡ ይህንንም በሶስት መንገድ እናከናውናለን፡- አንደኛ በቪዲዮ ኮንፈራንስ፣ ሁለተኛ በቴሌግራም ቻናላችንና ሶስተኛ በአካል ወርደን ድጋፋዊ ክትትል በማድረግ ነው፡፡ በዚህ ሂደት ውስጥ ሪፖርት እንለዋወጣለን፤ እንግዲህ በደቡብ ላይ ያለውን ለውጥ በዚህ መለካት ይቻላል፡፡ እኛም እንደሌሎች ሱማሌ ና ጋምቤላ ከውጭ ቀጥታ ከሚዋሰኑት ጋር እንዋሰናለን፤ ከማቆያ ሰብሮ መውጣት እና አምልጦ መውጣት እኛጋም አለ፡፡ አንድ ሁለት የሚሆኑት በዚህ በጌዴኦ በኩል እኛጋም አምልጦ የገቡበት ሁኔታ ነበር፡፡ ነገር ግን የዚህ ግብረ ሀይል ሚና ለግንዛቤ መፍጠርያ ዘልቆ ይገባል የኛ ቡድን፤ የታዘባቸውንና ያያቸውን ከአቅሙ በላይ የሆኑትን በሙሉ ይዞ ይመለሳል፡፡ ከዚህ ጋር በተያያዘ የህግና ጸጥታ ጉዳይ የሚከታተል በክልሉ ምክትል ርዕሰ መስተዳድር ማዕረግ የፐብሊክ ሰርቢስና ጸጥታ ክላሰተር አስተባባሪና የሠላምና ጸጥታ ቢሮ ሀላፊ በሆኑት በክቡር አቶ አለማዬሁ ባወዲ ሰብሳቢነት ላለው ግብረሀይል ዝርዝር ሁኔታዎችን ያቀርባሉ፡፡ በዚህ ሂደት ላይ የኛ ሚና በአጋጣሚ የምናያቸውን ተያያዥ ጉዳዮችን ለሚመለከተው ማቅረብ ሲሆን የጸጥታና ህግ ጉዳይ ግብረ ሀይል ተገቢውን ያከናውናል፡፡ ይህ ሂደት የፈጠረልን ሥራችንን በጋራ ተጋግዘን ማከናወን ብቻ ሳይሆን ግልጽ ቁርኝት የፈጠርንበት አግባብ ነው፡፡ ሌላው በጎ ጎኑ ይህ ዓይነት አሰራር አልፎ እኛጋ መምጣት ብቻ ሳይሆን በየደረጃው ወደጎንዮሽ የተፈጠረው አደረጃጀት በራሱ አቅም የሚችለውን መፍትሔ ይሰጣል፡፡ ከአቅማቸው በላይ የሆነ ጉዳይ ብቻ ነው ወደበላይ አመራርና ዐቢይ ግብረ -ሀይልጋ የሚመጣው፡፡

የኮቪድ-19 ወረርሽኝ ምላሽ ጋር ተያይዞ የተፈጠረ አዎነታዊ ገጽታ ካለ ቢገልጹልን

በነገራችን ላይ ከኮሮና ቫይረስ ጋር ተያይዞ በርካታ አሉታዊ ጎኖችን ማንሳት የሚቻል ቢሆንም ለጊዜው ገታ ላድርግና ወደ አዎንታዊ ገጽታ ሲመለስ ከአኗኗራችን ጋር በተለይ ከግልና አካባቢ ንጽህና አንጻር የእጅ መታጠብ ልማድ በአንክሮ የሚታይ ነው፡፡የህብረተሰብ አመለካከትንም ቀይሮታል፤ አንድ የህጻናት ሀኪም ጓደኛ አለኝ፤ ያለኝን ላካፍላችሁ፡- እንደቀልድ ነው ያነሳው ግን ለኔ ቁም ነገር ነው፡፡ጠፋችሁ …ሃሃሃ….ሁሁሁ.. ሳቅ…ለካስ ባክቴሪያ ነው ወደእርሱ ቤት የሚያመላልሰን ብዬ ከት ብዬ ሳቅሁ፡፡ አሁን እግዚአብሔር ይመስገን ይሕ የለም፤ ይህ ልምድ ሊቀጥል ይገባል፤ ብዙ ወጪ የማጠይቅ ግን በቀላሉ እጃችን በሳሙናና ውሀ ደጋግመን በመታጠብ ጤንነታችን መጠበቅ እንደምንችል ልምድ የወሰድንበት ነው፡፡ያደግንበት ገጠር ነው፤ በአፈርና በአመድ እጃችን በማሸት እየታጠብን ነው ያደግነው፡፡ይህ መልካም ባህል መሆኑን ተገንዝበን አጠናክረን ማስቀጠል አለብን፡፡ የጤናው ሴክተር እንደሚለው ጤናማና አምራች ዜጋ የመፍጠር የሚለው አሁን የተረጋገጠለት ይመስላል፤ አንዱ እሱ ነው፤ ሁለተቻው ጠንካራ የሆነ የቡድን ሥራ የሚያነቃቃ በተለይ ሰው በዚህ ዓይነት በተጠራ ጊዜ ወዲያ መድረስ ተነሳሽነት መጎልበት በፕሮግራም አፈጻጸም ያለመንጠባጠብ ማለት እኔ በሚመራው ግብረ-ሀይል ሌሎችን እንዴት እንደታዘባችሁ ባላውቅም ትልቅ የልማት መሳሪያ ሆኖ አግኝቻለሁ፡፡በተለይ በተለይ ለቀይ መስቀል፤ ቀይ መስቀል የህዝብ ተቋም ነው የሀብት ምንጩም ህዝብ ነው፤ ነገር ግን ባለፉት 80 ዓመታት አመራረሩም ሆነ ህዝቡ ያልተረዳውና እንደ መንግስታዊ ያልሆነ ተቋም ተመስሎ የሚታይ ነበር፡፡ነገር ግን አመራሩ እንዲረዳው ለካስ ቀይ መስቀል የህዝብ ተቋም ነው ብሎ ግንዛቤ እንዲያገኝ ያደረገ አጋጣሚ ነው፡፡ ኮቪድ ለኛ በግል ሥራችንን ህብረተሰብና የመንግስት አመራር እንዲያውቀው ዕድል የፈጠረ ነው ማለት ይቻላል፡፡ በክልሉ 15 ዞኖች ውስጥ የቀይ መስቀል መዋቅር አለ፤ በዚያ ሁሉ ቀይ መስቀል የዚህ ንዑስ ኮሚቴ ሰብሳቢ ሆኖ ይሰራል፡፡ ተነጥሎ ብቻውን ከሚፈጋበት ትልቅ ተቀባይነት እንዲያገኝ ዓላማውን እንዲያሰርጽ፣ ትልቅ ዕድል የተፈጠረበት ነው፡፡ እሱ ብቻ አይደልም፤ ዛሬ ተሳክቶልን ኮቪድ ቁጥጥር ሥር ቢዉል እንኳን እኔ እንደቀይ መስቀል ይህንን የተፈጠረውን አደረጃጀት እንደ አንድ የልማት ክንፍ ለሌሎች የልማት ሥራዎች ይዤ ነው የሚቀጥለው፡፡ 2010 ላይ በሚኒስትሮች ምክር ቤት ተሻሽሏል፤ በዚሁም በአካባቢ ጥበቃ በጤና በትምህርትና ንጹህ መጠጥ ውሀ ላይ ጭምር ገብተን እንዲንሰራ አሁንም ይህ ሲባል መንግስት ለአምቡላንስና መድሃኒት አገልግሎት ከሚሰጠው ድጎማ ውጭ ለልማት የሚሰጠን ሀብት የለም፡፡ነገር ግን ፕሮግራሞቻችን ሲሰፉ ሊያግዙን የሚችሉ ወደ 201 ሀገራት አባል ነን እኛ፡፡ በነገራችን ላይ በዚህ አባልነት እንደ ታዳግና ደሃ ሀገር አንቆጠርም እኛ፤በአቋም ደረጃ የአሜሪካ ቀይ መስቀል ማህበርና የኢትዮጵያ ቀይ መስቀል ማህበር እኩል ነው፡፡ በልመና ሳይሆን በመተባበርና በመካባበር ላይ የተመሰረተ ነው፡፡ የምናቀርባቸው ሀሳቦች ፕሮጀክቶች ናቸው ብዙን ጊዜ፤ በሶስት በዞኖች ላይ የ20 ሚሊየን ዩሮ ፕሮጄክቶችን ከትናንትና ወዲያ ተፈራርመናል፡፡ ይህ ፕሮጄክት ከትላልቅ ሀገሮች ጋር በትብብር መንፈስ የሚደረግ ነው፡፡ ለማለት የተፈለገው ለነዚህ ማስፈጸሚያ የሚሆን አደረጃጀት የተፈጠረበት መሆኑን ነው፡፡ ይህን ትልቅ አቅም ያለውን አደረጃጀት ይዞ መሄድ ለቀይ መስቀል ትልቅ የልማት አቅም መሆኑን ነው፡፡ ሶስተኛውና ትልቁ ነገር በማህበሩ ታሪክ የመጀመሪያ የሆነውን የሳኒታይዘር ማቀነባበሪያ ወልዶልናል፡፡ የጥራት ደረጃ በአይሶ 20 የተረጋገጠ ምርት ለጎረቤት ሀገሮችም ማቅረብ በሚቻል ጥራት ደረጃ ነው እያመረትን ያለነው፡፡ ሌላው በሀገራችን በንድፈሀሳብ ብቻ አተኩሮ የነበሩት እንደቴክኒክና የሙያ ማሰልጠኛ ተቋማት ባሁኑ ሰዓት በእግር በመርገጥ የሚሰራ የእጅ መታጠቢያ ማሽን ሰርተዋል፡፡ ትናንት ሻሸመኔ ባጋጣሚ ረብሻውን ለማስታገስ ስንቀሳቀስ ያየሁት አሁን ደግሞ በሰንስር የሚሰራ የእጅ መታጠቢያ ማሽን ነው፡፡ይህ ትልቅ ስኬት ነው፤ ይህንን የሰራው ጭንቅላትና እጅ ከዚህ በፊትም ነበር፤ነገር ግን ገፍተው ወደዚህ ሃሳብና ፈጠራ እንዲገቡ ኮቪድ ዕድል ፈጥረዋል፡፡ ከዚሁ ጋር በተያያዘ የአፍና አፍንጫ መሸፈኛ ጭንብል ብንወስድ በርካታ ዓይነት ስራዎች ተሰርተዋል፡፡ አንዱ ለምሳሌ በኛ ግብረ-ሀይል የተሰራው ይህ የኛና የአጋሮቻችን አርማ ያለበት ይህ ማሰክ ነው፡፡ እነዚህን ዕድሎችን ይዞ ነው የመጣው፤ ነገ ደግሞ ምን ፈጠራ ሊታክል እንደሚችል የምናው ይሆናል፡፡ በዚህ አናቆምም፤ ይህ በኛ በጎ ፈቃደኞች የተመረተ የዓይን የአፍንጫና የጆሮ ጠብታ ነው፡፡ የምርት ጥራት እንዲረጋገጥ ለሚመለከተው ልኬናል፡፡ሌላው የእጅ ጓንት ካምፓኒ ለመክፈት (እንከዋንም ተከሰተ ብዙ ጥፋት ሣያስከትል እንሸኘዋልን) እስካሁን በነበረው ሁኔታ በሳኒታይዘር አቅርቦት አኳያ የጤና ተቋማት ፍላጎት ማሟላት አይቻልም ነበር፤ ያለው ተሰብስቦ ሁሉ እዚህ እንዲመጣ ነው የጤን ቢሮ ደብዳቤ ላኛ የጻፈው እኛ እንዲናሟላ፤ ፍጆታው ከፍተኛ ነው፡፡ 20 ሺህ ሊትር ለዚያ የሚሆን ግብዓት ሀብት የክልሉ መንግስት ድጋፍ አድርገዋል፡፡ ነገርግን ቀጣይነት ባለው ሁኔታ የመንግስት ና የመንግስት ልማት ተቋማት ከዚህ እንዲያገኙ የግዥ ኤጄንሲ ለተቋማት የጻፈው፤ ይህ ቀላል አይደለም ነገር ግን ከዚህ በፊትም ሊሰራ የሚችል ነበር፡፡ በቀላሉ ሰርተን የሀገራችን ልማትና ዕድገት ማፋጠን የምንችልባቸው ያመለጡን ዕድሎች እንድናይ አድርጎናል፡፡ ይህን ሥራ ከጀመርን ወዲህ ገቢያችንም በብዙ ሚሊየን ብር አድገዋል፡፡ሌላው ላስቲክ የእጅ ጓንት ከፍተኛ ፍላጎት ቢኖርም አቅርቦት ችግር አለ፡፡ ምክንያቱም ከውጭ ስለምናስገባ፤፤ ለዚህ የሚሆን ጥሬ ዕቃ በሀገራችን እያለ፤ ከዚህ በፊት ትግራይ ክልል ማምረት ሞክረው ግን የተቋረጠ መሰለኝ፡፡ ይህን የእጅ ጓንት ለማምረት አስጠንተናል፤ ለማሽን ግዥ ወደ 8 ሚሊየን ብር ያስፈልጋል፡፡ ይህንን ገንዘብ በሶስት ወር ሽያጭ ማግኘት እንችላለን፡፡ ለዚህ የሚሆን ጥሬ እቃ 70 በመቶ እንሰት እና 30 በመቶ የጎማ ዛፍ ነው፡፡ባለሙያ ነው ከውጭ የምናመጣው፡፡ ቴክኖሎጂው በጣም ቀላል ነው፡፡ ባለሙያ ከእስራኤል ሀገር ለማምጣት ንግግር ጀምረናል፡፡ኮቪድ ይሄንንም ይዞ መጥተዋል ለማለት ነው፡፡

በድጋፋዊ ክትትል ወቅት መታረም የሚገባቸው ጉድለት ናቸው የሚትሉት ካሉ

እንደአጠቃላይ እውነት ለመናገር እንደደቡብ ክልል የምንሰማቸው ሪፖርቶች አስደንጋጭ አይደሉም፡፡ባለፉት ጊዜያት ያስተናገድናቸው ኬዞቹም ቢሆኑ ከአዲስ አበባ ና ከሞያሌ ለይቶ ማቆያ ሰብሮ የመጡት ናቸው፡፡ ስለዚህ በተወሰነ ደረጃ ትኩረት ሰጥተን መርተናል የሚል አስተያየት አለኝ፡፡ በሌላ ጎን ደግሞ ወደታች እየወረድን ስንሄድ ህዝቡ ቸልተኛ ሆነዋል የሚባለው ከህዝቡ ሳይሆን ከአመራሩ እንደሆነ፤ አመራሩ እያየ እንዳላየ ካለፈ ህዝቡ ምን ማድረግ ይችላል? የጸጥታ ሀይሉ እንደዚያው እያየ ዝምብሎ ካለፈ አስቸጋሪ ነው፡፡ወደታች እየወረድን ስንሄድ የምናየው ይህንን ነው፡፡ በበላይ አመራር ደረጃ በየሳምንት ይገመገማል አቅጣጫዎች ይቀመጣሉ፤ ተግዳሮቶች ይፈታሉ፤በድጋፋዊ ክትትል ይታጀባሉ፤ ስለዚህ አመራርን ሰጥቶ በመቀበል ላይ ያሉ ክፍተቶች ባስቸኳይ ካልጠበቡ ሀዋሳን ጨምሮ በክልሉ ያሉት ትላልቅ ከተሞች የጥፋት ማዕከል የማይሆኑበት ምንም ምክንያት አይኖርም፡፡እኔም በሳምንት አንድ ጊዜ ተወርዉሬ ዞሬ አይቼ ድጋፍ አድርጌ እመለሳለሁ፡፡ለምሳሌ አሁንም በገጠር አንድ ባለሁለት እግር የሞተር ብስክሌት ከ3-4 ሰዎች ይጭናል፡፡ የትራፍክ ፖሊስ እያየ ያሳልፋል፤ ይህ ምን ማለት ነው? ይህ ጉዳይ ጥፋት ነው ከተባለ አንድ ሞተር ሳይክል በዚህ ዓይነት ሁኔታ ስንት ሰዎችን ያመላልሳል? እነዚህን ሰብስበን ብንመረምር ከ10 ውስጥ 5 በበሽታው የተያዘ እናጣልን? ሰባት ሞቶ በአስከሬን ምርመራ አምስት ከተገኘ፤ እይታችን እስከዚህ ሰፋ ማድረግ ያስፈልጋል፡፡በተለይ በታችኛው መዋቅር የአመራር ቁርጠኝነት ያስፈልጋል፡፡ ቀብር ሥነሥርዓት ላይ ምንም ለውጥ የለም፤ይህን ማስቆም እኮ ይቻላል፤ ከተሞች ላይ ለምን ቆሜ? ከከተሞች በላይ የገጠሩን መቆጣጠር ይቀላል፡፡ የሆነ ሆኖ የአመራር ዘይቢያችን አስተማሪና የችግሩን ስፋት ልክ ካልሆነ በተወሰነ ሰው ምክንያት ሀገርና ህዝብ ሊጠፋ ስለሚችል ጥናታችሁም ይህንን ፍንትው አድርጎ ቢያሳይ መልካም ነው እላለው፡፡

ክልል ማዕከል ቁልፍ መረጃ ሰጭ 2.

የክልል መምህራን ማህበር

የተቋም ለኮቪድ-19 ወረርሽኝ መከላከልና ቁጥጥር ሥራ ጋር ያለው ቁርኝት ምን ይመስላል?

ከዚህ ከኮቪድ-19 በሽታ ጋር ተያይዞ በሀገር ከመከሰቱ በፊት በዓለም መከሰቱን በተለያዩ ሚዲያ ሲገለጽ እንደሰማነው እና የሀገሪቱ የጤና ሚኒስቴርና በስሩ ያሉት መዋቅሮች በሽታውን ለመከላከል የግንዛቤ አስፈላጊነት በየደረጃው ያለው ባለድርሻ አካላት መሳተፍ አስፈላጊነትን እንደክልሉ መምህራን ማህበር ተገንዝበን በራሳችን ደረጃ በስራ አስፈጻሚ ተወያይተን በማስወሰን የራሳችንን ንዑስ ኮሚቴ አቋቋምን፡፡ ከዚያም በታችኛው መዋቅር ከኛ ልምድና ተሞክሮ እንዲወስድ እንደአቅማችን (ማህበራችን ከአባላት በምትዋጣ መዋጮ ነው የምንቀሳቀሰው) የአስር ሺህ ብር ድጋፍ ለቀይ መስቀል ማህበር አድርገናል፡፡ ሌላው ታችኛው መዋቅር ከዞን ወረዳና እስከትምህርት ቤት ድረስ ባለው ግንዛቤ የመፍጠር ሥራ ከመሥራት አንጻር በመጀመሪያ ደረጃ በሃዋሳ ከተማ ሪቀትን በመጠበቅ ራሳችውን ስለመጠበቅ እንደቀይ መስቀል ማህበር ካሉት ባለድርሻዎች ጋር ስልጠና ሰጥተናል፡፡ ከዚያም በክልል ደረጃ የተሰሩትን ሥራዎች እንደአብነት በመያዝ በታችኛው መዋቅር በተለይ መምህራን ጋዋን ለብሰው ወጥተው ህዝቡን እንዲያስተምሩ የማድረግ እጅ እንዲያስታጥቡ እና አቅመ-ደካማ የሆኑትን እንዲደግፉ ከማድረግ አንጻር በየደረላው ካሉት የኛ መዋቅሮች ጋር በርካታ ስራ ለመሥራት ሞክረናል፡፡ የሠራናቸውን ሥራዎች በፌስቡክ ገጻችንና በደብዳቤ ለሚመለከታቸው አሳውቀናል፡፡

በዚህ ምላሽ ሂደት ከቀይ መስቀልና ከናንተ መዋቅር ውጭ ትብብር የሚታደርጉት ተቋማት ካሉ?

ከዚህ ጋር ተያይዞ ከክልሉ ሴቶችና ህጻናት ቢሮ ጋር በመሆን በዲላ ከተማ ሴት መምህራን ጋዋን ለብሰው የግንዛቤ ማስጨበጫ ስራ እንዲሰሩ ተደርገዋል፡፡ ይህን ያደረግነው ሞንታሪቦና ጄነሬተር በራሳችን ተከራይተን ነው ቅስቀሳ ያደረግነው፡፡ በጌዲኦኛና በአማሪኛ ቋንቋዎች በከተማ ውስጥ በመዘዋወር ነው የተከናወነው፡፡ሌላው ከክልሉ ቀይ መስቀል ጋር በመተባበር በዚያውም በንዑስ ኮሚቴ ውስጥም ስላለን የድርሻችን እየተወጣን ያለንበት ሁኔታ ነው ያለው፡፡በክልላችን ቢያንስ እስከ 10000 የሚሆኑ በጎ ፈቃደኛ መምህራን ስላሉ እነዚህንና ሌሎች መምህራን የመመልመል ሥራ በታችኛው መዋቅር በጥሩ ሁኔታ ነው የተከናወነው፡፡ የአባላትን መዋጮ ከማሰባሰብና ለዚህ ዓላማ እንዲያግዝ ከማድረግም አንጻር ለምሳሌ የጉራጌ ዞን አስከ 15000 ብር በማዋጣት ወረዳዎች እስከ 1000 ብር እሰከ ሁለት ሺህ ደቡብ ኦሞም ጋሞ እንዲሁም ደግሞ በኮንታ ልዩ ወረዳ በቤንች ሼኮ ዞን አካባቢ እና ሌሎችም አካባቢ መምህራና ገዋን ለብሶ በመውጣት መናሔሪያና ገበያ አካባቢ የግንዛቤ ማስጨበጫ ቅስቄሳና የእጅ ማስታጠብ ሥራ ሠርተዋል፡፡ አዚህ አበላ ቱላ ክፍለ ከተማ የማህበሩ ሥራ አስፈጻሚ ሊቀመንበሩን ጨምሮ መምህራንን አስተባብሮ የግንዘቤ ማስጨበጫ ሥራ ሠርተዋል፡፡ በሌሎች የክልል አካባቢዎችም ከተለያዩ ባለድርሻ አካላት እንደጤና ቢሮና ሌሎች ትላልቅ ተቋማት ድጋፍ በመጠየቅ ለመሥራት ያቀድናችው በድጋፍ ውስኑነት አልተሳካም፡፡ ይሁን እንጂ መምህራን ራሳችውን በመጠበቅና ለህብረተሰቡ የመረጃ ምንጭ በመሆን በየትኛውም ሁኔታ ሪቀታቸውን በመጠበቅ ቤተሰቦቻቸውንና ልጆቻቸውን እንዲጠብቁ በማህበሩ በኩል መልእክት ተደራሽ ተደረጎላቸዋል፡፡ ሌላው ስኖር ነው እኔ የሚኖረው የሚለውን መርህ በማንገብ የኑሮ ትስስር አይቀረነት በመገንዘብ በረጃ በማስተላለፍ ረገድ ሰፊ ስራ ተሰርተዋል፡፡

ይሁን እንጂ ባሁኑ ጊዜ የተወሰነ መቀዛቀዞች አሉ ግን ይህ መቀዛቀዝ ሥር እንዳይሰድ መስራት ያስፈልጋል፡፡

በሽታው የማጥቃት አቅሙን እየጨመረ ባለበት ሁኔታ የምላሹ ሁኔታ መቀዛቀዙ ምንድነው ምክንያቱ?

የተነሳሽነት መቀነሱ ነገር በሁሉም ተቋማት አካባቢ የሚታይ ነው፡፡ በመጀመሪያ አካባቢ እዚህ እኛ ተቋምም ሰው ከመግባቱ በፊት እጅ እንዲታጠብ አስገዳጅ ሁኔታ ነበር፤ እንዲሁም ሌሎች ተቋማትም እንደዚሁ፤ መጀመሪያ አካባቢ ሁሉም በሚባል ደረጃ የግንዛቤ ማስጨበጫ ስራ በሚገባ ይሰራ ነበር፡፡እየተቀዛቀዘ የመጣበት ሁኔታ የድጋፍና ክትትል ሁኔታ ከኛም ጀምሮ ጉድለት ስላለ ነው፡፡ በኛ በኩል ወርደን ተገቢ ድጋፍና ክትትል እንዳናደርግ የተሸከርካሪና በጀት ውሱኑነት አለብን፡፡በእጃችን ያሉትን አቅምና መልካም ተሞክሮችን እንዳናሰፋ በተለይ ከሴቶችና ህጻናት ቢሮ ጋር ሆነን በጌዴኦ የሰራነውን ዓይነት ሴት መምህራንን የተጠቀምንበት ሴቶች ካላቸው ተጋላጭነትና ሰፊ ልምድ (በጎጂ ባህል ተጠቂነት፤ ከልጆች ጋር ያላቸው ቅርርብ) እኛ ራሳችን የማህበሩ አመራር መምህራን እንደመሆናችን ወርደን መስራት ማስተማርና አርአያ መሆን እንዳለብን እንገነዘባለን፤ ግን የአቅም ውሱኑነት አለ፡፡ በመሆኑም እዚህ ሆነን ይህን አድረጉ ያን አድርጉ ከማለት ባሻገር በየደረጃው ያሉትን አመራሮችንና አባላትን አነቃንቀን ማሰማራት አልቻልንም፡፡ ሳኒታይዘርና ማስክም በበቂ አላገኘንም፤ ሆኖም ግን ከጨርቅ የሚሰፋውን በኛ ወጭ አሰፍተን የተወሰኑትን አሰራጭተናል፡፡ በዚህም የተወሰኑት መምህራን ወጥተው እንዲያስተምሩ አድርገናል፡፡

መምህራንን ብናንቀሳቅስ በክልላችን የሚገኙ ከ7 ሚሊየን በላይ ተማሪዎችንና የነርሱን ወላጆች በቀላሉ ተደራሽ ማድረግ ይቻል ነበር፡፡ ስለዚህ እኛ ከዚህ ወደ ዞን ዞኑ ደግሞ ወደ ወረዳ ወረዳም ወደ ትምህርት ቤትና ቀበሌ ድረስ በየተዋረድ ወርደን ብናስተምርና ድጋፍ ብናደርግ አስፈላጊ የባህሪይ ለውጥ በማምጣት ህብረተሰብ ራሱንና ቤተሰቡን መከላከል ይችላል የሚል የጸና እምነት አለኝ፡፡

የናንተ ማህበር ከኢትዮጵያ መምህራን ማህበር ጋር በዚህ በሽታ መከላከልና ቁጥጥር ሂደት የተፈጠረ ቁርኝት ካለ?

የኢትዮጵያ መምህራን ማህበር ገና ከጅምሩ የበጎ ፈቃደኛ መምህራን መልምላችሁ ላኩልን ብለው ጠይቀውናል፤ እኛም ከሁሉም መዋቅር ዞንና ወረዳ መልምለን ኮታችን ልከናል፡፡ ይህም ስልጠና ለመስጠት ነው፡፡ ከዚያ ውጭ እዚህ እኛ ለምንሰራው የግንዛቤ ማስጨበጫ ሥራ እንድንሰራ በስልክና በደብዳቤ ነው የተገለጸልን፡፡ የሰራናቸውን ሥራ ሪፖርት በፖስታ በስልክና በቴሌግራም እንልክላቸዋለን፡፡ ከዚያ ውጭ ከነርሱ በሥራ ማስኬጃ ድጋፍ በኩል የተደረገልን ድጋፍ የለም፤ ሌላው ያደረግነው ነገር ቢኖር በሀዋሳ ከተማ እያስገነባን ያለውን ህንፃ ችግሩ እየሰፋ ከመጣ ለማቆያ ወይንም ለህክምና እንዲጠቀሙ ለማድረግ አስበን የገለጽንበትና በሚዲያም ጭምር ያሳወቅንበት ሁኔታ ነበር፡፡ እኛ የተንቀሳቀስነው ቀዳሚ ተጎጆ ተማሪዎችና የተማሪ ቤተሰቦች ናቸው (ይህም የክልሉ ህዝብ ባጠቃላይ እንደማለት) አስበን ባለን ውስን አቅም ነው፡፡ ለዚህም ያቀድናቸውን ዳር ለማድረስ ድጋፍ እንዲደረግልን የተለያዩ መንግስታዊና መንግስታዊ ያልሆነ ተቋማትን ጠይቀናል ግን ምላሽ አላገኘንም፡፡ እኛ የዐቢይ ኮሚቴ አባል አይደለንም ግን በንዑስ ኮሚቴ ሥር የበጎ ፈቃድ አገልግሎት ኮሚቴ አባል ነን፤ ይህም ኮሚቴ የሚመራው በቀይ መስቀል ነው፡፡ በዚያ ንዑስ ኮሚቴ ከአርቲስቶችና ሙዚቀኞች ጋር በመሆን በሚዲያ የሚተላለፉትን መልእክቶችን ኤዲት እናደርጋለን፡፡ለምሳሌ የደቡብ ሙዚቄኞች ማህበር የሰራውን ኤዲት ያደረግን እኛ ነን፡፡ በበጀት የደገፈው ሌላ የመንግስት አካል ሆኖ እኛ በሙያ ደግፈናል፡፡

በዚህ የወረርሽኝ መከላከልና ቁጥጥር ሥራ ውስጥ እንድትሳተፉ ያደረገው ምክንያት ምንድነው?

በዋናነት ያነሳሳን ነገር ሀገራዊ ሁኔታና የኢትዮጵያ መምህራን ማህበር ያስቀመጠው አቅጣጫ ነው፡፡ ከዚህ መነሻነት እኛም በክልል ደረጃ ብንሰራ ይሻላል የሚለውን አስበን የአቅም ግንባታና የግንዛቤ ማስጨበጫ ስራዎችን ቀይ መስቀል ማህበር ወጪውን ሸፍኖልን የሰራነው በራሳችን ተነሳሽነት ነው፡፡ ከኮቪድ-19 ጋር በተያያዘ ስራውን የሚመራ የህዝብ ግኑኝነት ኮሚቴ አለን፤ በዚያ ኮሚቴ አማካይኝነት ወደየዞኑ እየደወልን አፈጻጸሙን እንከታተላለን፡፡ የተሰሩት ሥራዎች ሪፖርት በፎቶ በተደገፈ እንዲላክልን እናደርጋለን፡፡ ከዚህ ጋር በተያያዘ በርካታ በሰነድ የተደገፉ መረጃዎች አሉን፤ ካስፈለገ እንሰጣቸዋለን፡፡ ባነር አሳትመናል እንዲሁም የእጅ መታጠቢያ ማሽን ከቴክኒክና ሙያ ኮሌጆች ጋር በመሆን የተወሰኑትን ወጪ ሸፍነን ለተወሰኑት መዋቅሮች ልከናል፡፡ ሌሎች የምግብና አንዳንድ ቁሳቁሶች ድጋፍም አድርገናል፡- ለምሳሌ ጎፋ ላይ ማህበሩ ከአባላት መዋጮ ሰብስበው ከተወሰኑት ቀበሊያት ለተወጣጡ ከ100 በላይ ለሆኑት የንጽህና መጠበቂያ ቁሳቁሶችና ዜይት እንዲሁም ሌሎች የምግብ ሸቀጦችን አድለናል፡፡ ተመሳሳይ ድጋፍ በጋሞ በኮንታ ሌሎችም አካባቢ ተከናውነዋል፡፡ እንደዚህ ዓይነት በፎቶ የተደገፉ ማስረጃዎች አሉን፡፡ ሌላው አሁን እዚህጋ የሚታየው በኮንታ ወረዳ መምህራን ገበያ የሚመጡትን ገዋን ለብሶ ገበያተኞችን እጅ እያሳጠቡ መሆኑን ነው፡፡ ይህ ባስኬቶ ላይ፡ ይህ ደግሞ ጉራጌ ላይ የተሰራ ነው፡፡ ጉራጌ ዞን በ15000 ብር የሚያወጣ ሳኒታይዘር ሳሙናና ሌሎች ቁሳቁሶችን ለዞኑ የቀይ መስቀል ማህበር ሰጥተዋል፡፡ ይህ አሁን መምህራን ለሰብአዊነት በሚል የተሰጠ ስልጠና ነው፡፡ እንግዲህ ከተለያዩ ተቋማት ጋር በመሆን በርካታ ሥራዎችን ስንሰራ ቆይተናል፡፡ እያንዳንዱ ዞን በገንዘብና በቁሳቁስ የደገፉበት ሪፖርት በየጊዜው ለኛ ከታችኛው መዋቅር ይላክልናል፡፡ ደቡብ ኦሞና አርባምንጭ ከተማም ላይ መምህራን በመናሔሪያ ፊት ለፍት ሆነው ተሳፋሪዎችን እጅ እያሳጠቡና የግንዛቤ ማስጨበጫ በመስጠት የተሳተፉበት ነው፡፡ አንዳንድ ቦታዎች በተሻለ የምንቀሳቀሱ ያሉትን ያህል እንቅስቃሤያቸው ደካማ የሆኑትም አሉ፡፡ ለዚህም የኛ በበቂ ሁኔታ ወርደን ድጋፍና ክትትል ማድረግ አለመቻላችን ነው፡፡ የህዝብ ግኑኝነት ስራችንን በቴሌግራምና በፌስቡክ ፔጃችን ተደራሽ እናደርጋለን፤፤ ለምሳሌ በኔ ስም አማነ በሚል በተከፈተ የፌስቡከ ፔጅ በየቀኑ መረጃ እልጠፋለሁ፤ ሁሉም አባሎቻችንና ደጋፊዎቻችን በዚህ ሁኔታ መረጃ ያገኛሉ፡፡

ማህበራችን በዞንና ወረዳ ደረጃ ከባለድርሻ አካላት ጋር የሚሰሩትን ክንውኖችን የምንከታተልበት የቪዲዮ ኮንፈራንስ አንዱ መስመራችን ነው፡፡

ቅንጅታዊ ስራ በተለያዩ እርከኖች ያለው ቁመና ምን ይመስላል?

ዋናው የማህበራችን የስራ ትብብርና ቁርኝት ከቀይ መስቀል ማህበር ጋር ነው፡፡ በዐቢይ ኮሚቴ ስር የሀብትና በጎ አድራጎት ንዑስ ኮሚቴ አለ፡፡ እና በዚህ ንዑስ ኮሚቴ ተሳታፊ ነን፡፡ ከሌሎች በርካታ ተሳታፊ ባለድርሻ አካላት ጋር በዚሁ ማዕቀፍ እንሳተፋለን፡፡ በዚሁ ንዑስ ኮሚቴ ከሌሎች ጋር በመሆን እስከ 500000 በጎ ፈቃደኛ አባላትን ለመሳተፍ አብረን እንሰራለን፡፡ በዞን ደረጃም የኛ መምህራን ማህበር ቀይ መስቀል ማህበር ባለባቸው ዞኖች ላይ በመተባበርና በመቀናጀት እየሰራ ነው፡፡ የኛን መምህራን አንቀሳቅሰን በጋራ በማሰማራት የተሻለ ስራ ሰርተናል ማለት ይቻላል፡፡ እኛም አቅደን እየሰራን እንገኛለን፤ በቀጣይም ተቀናጅተን በመስራት ለውጥ እናመጣለን፡ ሌላው በጋራ ስንሰራ የነበረው የኢንተረፕራዝና እንዱስትሪ ልማት ቢሮ ነው፡፡ ሌሎችንም ተቋማት በጋራ እንዲንሰራ ጥያቄ አቅርበን እየተጠባበቅን እንገኛለን፡፡ በቀላሉ ሳኒታይዘር ሳሙና እና የፊት መሽፈኛ ጭንብል ብናገኝ ከዚህ የተሻለ ስራ መስራት እንችላለን፡፡

ከቀይ መስቀል ጋር በመቀናጀት በክልሉ በተለያዩ አካባቢዎች ወላይታ፣ ሀላባ፣ ከምባታ እና ሌሎች አካባቢዎችም ከሚዲያ ሰዎች ከአርትስቶችና ኮሜዲኖች ጋር በመሆን የግንዛቤ ማስጨበጥ ስራ ስንሰራ ቆይተናል፡፡ ሁሉም ስራዎች የተሰሩት በቀላል ወጪ በበጎ ፈቃደኝነት መንፈስ ነው፡፡

የዕቅድና ግምገማ ሥራዎቻችን ወደታለመለት ዓላማ ስለማምራታቸው እንደት ተረጋገጠ?

የክትትልና ግምገማ ስርኣታችን እየተዳከመ መምጣት ብቻ ሳይሆን ወደ ተስፋ መቁረጥ እደረስ ነው፤ ምክንያቱም በሽታው እየተስፋፋ እየመጣ የኛ ተግባር መዳከሙ ነው፡፡ አንዳንድ ተስፋ የሚያስቆርጡ ሁኔታዎች በህብረተስብ ዘንድ ይታያሉ፡፡ በተለይ አካላዊ ሪቀትን ጠብቆ ከመቆም አንጻር የፊት መሸፈኛ ጭንብል ከመጠቀም በርካታ ጉድለቶች መታየቱ በተለይ ሰፊ የግንዛቤ ማስጨበጫ ስራ ከተሰራ በሃላ መሆኑ ነው፡፡ የኛም የተለያየ መዋቅር ከዚህ በፊት በተነሳበት ግሌትና ወኔ ያለመጓዝና መንሸራተትና አቅም ማነስ ይዞናል፡፡ ሌላው የክልሉ ወቅታዊ ሁኔታ አብዘኛውን ከተግባር እንዲነጠልና ተስፋ እንዲቆርጥ እያደረገ መሆኑ መቀመጫችን የት ነው የሚሆነው ብሎ ወሬ አድማጭ መሆኑ በየደረጃው ያለው ግብረ ሀይልም ሆነ ባለሙያ ተግባሩን ትተዋል፡፡ ይህም በክልላችን የኮሮና ወረርሽን የሚፎካከር ከእሱም በላይ ትኩረት ያሻ የክልል አደረጃጀት ጥያቄ ተነስተዋል ማለት ይቻላል፡፡

ኮቪድ-19 በሽታን ለመከላከልና ለመቆጣጠር በሚደረገው ሂደት የተገኘ መልካም ተሞክሮ ካለ

ኮቪድ-19 በሽታ ከመጣ ወዲህ ምንም እንከዋን የጤና ባለሙያ ባልሆንም ሌሎች ሰዎችም እንደሚያወሩና እኔም እንደተገነዘብኩ ህብረተሰቡ እጁን በውሀና በሳሙና መታጠብን በመለማመዱ ከንጽህና ጉድለት ጋር የሚከሰቱ ተላላፊ በሽታዎች ወደ ሰማኒያ በመቶ ያክል መቀነሳችውን ነው፡፡ እንደ ታይፎይድ ባክቴሪያ የመሳሰሉት መቀነሳቸውን ነው፡፡ ከዚህ የተማሪነው ኮሮና ቢኖርም ባይኖርም እጃችን ዘውትር በሳሙናና በውሀ በመታጠብ በርካታ ተላላፊ የሆኑትን በሽታዎችን መከላከል እንደምንችል ነው፡፡ሌላው ደግሞ ከባለድርሻ አካላት ጋር ተወያይተንና ተገናኝተን ስራችንን እንድንሰራ የተፈጠረው ዕድል መሆኑ ነው፡፡ ከቴክኖሎጂ አጠቃቀምና ፈጠራ ስራ ከመስራት አካያ የቴክኒክና ሙያ ኮሌጆች በርካታ የእጅ መታጠቢያ ማሽኖችንና ሌሎች ገብኣቶችን ማምረት መጀመራቸው እንዲሁም ወጣቶች በርካታ የፈጠራ ስራ ላይ መሳተፋቸው የሚጠቀሱ ናቸው፡፡

የባለድርሻ አካላት ተሳትፎ የተሻለ እንዲሆን ያደረገው ምንድነው መገለጫውስ?

ከተለያዩ ተቋማት እንደሴቶችና ህጻናት ስፖርት ኮሚሽን ቀይ መስቀል ከመሳሰሉት ጋር በጋራ ተቀናጅተን ያከናወንባችው ስራዎች ምሳሌ የሚሆኑ ናቸው፡፡ሪቀታቸውን ጠብቀው ባደረጉት የብስክሌት ግንዛቤ ማስጨበቻ ፕሮገራም መረጃ ተደራሽ ከማድረግ ባሻገር የደም ልገሳ ማድረጋቸው እጅግ ትልቅ የአብሮነት መገለጫ የሆነ ጉዳይ ነው፡፡ በሀገር አቀፍ ደረጃ በዚህ ዙሪያ በተደረገው ግምገማ ከክልሎች የኛ የተሻለ አፈጻጸም የታየበት ነበር፡፡ መምህራንን አስተባብሮ የበጎፈቃድ ስራ በማሰራት የተሻለ ተሞክሮ ያሣየ ክልል እንደነበረ ተጠቅሰዋል፡፡ በማህበራዊ ሚዲያና በዋናው ሚዲያ አጠቃቀምና ዞኖችን በማነጻጸርና በማወዳደር በተደረገውም የተሻለ ቅንጅታዊ ስራ የታየበት እንደሆነ ማንሳት ይቻላል፡፡

ክልል ማዕከል ቁልፍ መረጃ 3.

ጤና ቢሮ

የኮሮና ቫይረስ በሽታ በመከላከልና ቁጥጥር ሂደት የጤና ቢሮ ቁርኝት ምን ይመስላል?

- በማስተባበር ከሌሎች ጋር ተደጋግፎ ከመስራት እና ቢሮውን ለተልዕኮ ብቁ ከማድረግና በአጋርነት ከመስራት አኳያ

በሽታው መከሰቱ እንደታወቀ የፌደራል መንግስት አቅጣጫ ከመውረዱ አስቀድሞ በክልላችን ርዕሰመስተዳድር መሪነት ለችግሩ ምላሽ ሚሰጥ ግብረሀይል እንዲቋቋም ተደረገ፡፡ ያኔ ስንነሳ ኮር ኮሚቴውን ርዕሰመስተዳድሩ እንዲመራና የቴክኒክ ኮሚተውን ጤና ቢሮ እንዲመራ ነበር፡፡ በዚያ አደረጃጀት የበሽታው አሉታዊ ጎንና ይበልጥ ሊያጠቃ የሚችል የህብረተሰብ ክፍሎችንና አካባቢዎችን የመለየት ስራ እንሰራ ነበር፡፡ በዚህ ሂደት የሃዋሳ ከተማንም ጨምረን ነበር፡ ምክንያቱም ሀዋሳ ከተማ ካለው ነባራዊ ሁኔታ ዝግጅት ከሌሎች ጨመር ማድረግ ስላስፈላገ፡፡ በኋላ ሁኔታውን ስናየው የቴክኒክ ሥራ በጤናው ሴክተር ብቻ የምንወጣው ባለመሆኑ ተጨማሪ ዘጠኝ ሴክተሮችን በማካተት የባለድርሻ አካላት ተሳትፎ የጨመርንበት ሁኔታ አለ፡፡ ከነዚህ ዘጠኛ ንዑሳን ኮሚቴዎች ውስጥ ጤናው የሚመራው የኮቪድ-19 በሽታ መከላከልና የጤና አገልግሎት ንዑስ ኮሚቴ አንዱ ሆኖ ነው የተደራጀው፡፡ ይህን አደራጃጀት እስከ ወረዳ በተነቃቃ ሁኔታ የተዋቀረና እስከቀበሌም ለማድረስ የተሞከረበት ነው፡፡ እንደጤናው ሴክተር ዝግጅት ስናደርግ ታሳቢ ያደረግነው የተለያዩ የመረጃ ምንጮችን ተጠቅመን የበሽታው ከፍተኛ የጎጂነት ደረጃ (the worest case scenario) ወስደን ነው ያቀድነው፡፡ የዓለም ጤና ድርጅት ባወጣው የበሽታውን ሂደትና የሚያመጣው ውጤት ሚዛን መሠረት 80 በመቶ በበሽታው ቢያዝም በተመላላሽ ሊታከም የሚችል አሊም ምልክት ሣያሳይ የሚድን እንደሚሆን፤ 15 በመቶ ሆስፒታል ተኝቶ መታከም የሚያስፈልግ ከዚም 5 በመቶ ፅኑ ህክምና የሚያስፈልጉ እና 2 በመቶ ደግሞ ሰው ሰራሽ የመተንፈሻ መሳሪያ እንደሚስፈልግ በተተነበየው መሰረት መዘጋጀት ጀመርን፡፡ ይህም የኳራንታይን ማዕከላትን የለይቶ ማቆያ ማዕከላትንና የህክምና ማዕከላትን ማቋቋም እና ለማዕከላቱ አስፈላጊውን ግብዓቶችን የማሟላትን ያቀፈ መነሻ ዕቅድ አዘጋጅተን ነው ወደሥራ የገባነው፡፡

የጤና ሚኒስቴር ዕቅድና የክልሉ ጤና ቢሮ ዕቅድ የመናበቡ ደረጃ ምን ይመስላል?

እኛም ስንነሳ ከዜሮ አልተነሳንም፤ እንዲሁም የዩናትድ ኪነግደም ኢምፔሪያል ኮልጅ ያወጣውና የሀገራዊ ትንበያ ይዘን ከሀገራዊ ትንበያ ጋር በሚጣጣም መልኩ ነው ያቀድነው፡፡

የዐቢይና የንዑሳን ግብረ ሀይሎች የጤናውንም ጨምሮ ዕቅዶች የመናበብ ሁኔታና ለሁሉም ዕቅዶች መነሻ የሆኑት ሁኔታዎች ብቢራሩ፡-

መጀመሪያውኑም ዘጠኝ ንዑሳን ኮሚቴዎች ሲቋቋሙ መነሻው የባለብዙ ድርሻ አካላት ተሳትፎ በሚል ዕቅድ ተዘጋጅቶ ነው፡፡ ጤናው የራሱን ሲያዘጋጅ የባለድርሻ አካላት ዕቅድ መነሻ አድርጎና ከዚያ ቀድተው ነው ያዘጋጀው፡፡አንዳንድ ሴክተሮች ዕቅድ በየዘርፋቸው ለማዘጋጀት ሙሉ በሙሉ ግልጽ በሆነ ሁኔታ ሊጣጣም ይቸገሩ ይሆናል፤ ዞሮ ዞሮ ትላልቅ ሴክተሮች የዕቅዱ አካል ሆነው ነው የሄዱት፡፡ ለምሳሌ ሀይጂንና ሳኒቴሽንን በቀጥታ የውሀ ቢሮ እንዲመራ አድርገናል፤ እንደዚያውም የትራንስፖርትንም እንደዚው፤ንግዱም ላይ በመጀመሪያ አካባቢ የነበረው የገበያ ቀውስ ከባድ ስለሆነ እነርሱም ያንን ዘዘርፍ በሃላፊነት እንዲመሩ የተደረገበት ሁኔታ ነበር፡፡

ሥራዎችን ከታችኛውና ላይኛው እንዲሁም ውደጎነዮሽ ካሉት መዋቅሮች በተደራጀ የቅንጅት አግባብ የተተገበረበት ሁኔታ ያለው ገጽታ ቢገለጽ?

በዚህ ሂደት ውስጥ እንደመልካም አጋጣሚ ነው ብዬ የሚወስደው ችግሩ ምንም ከባድ ቢሆንም በሂደቱ በተረባረብንበትና በጋራ ለመስራት በተጣጣሪንበት ሂደት የጤናው ሴክተር ችግር ጎልቶ እንዲታይ መሆኑ ነው፡፡ በዚያው ልክ ከታችኛውና ላይኛው መዋቅር ጋርም በመልካም ተነሳሽነትና ቁርጠኝነት መግባባት ተፈጥሮ የተንቀሳቀስንበት ሁኔታ ታይተዋል፡፡ አመራራችንም ይህንን እንዲያውቀው ነው ያደረገው፡፡ በየሳምንቱ ራቡዕ ከጤና ጥበቃ ሚኒሰትር ጋር የማይቋረጥ ምክክር መድረክ አለ፤ ዞኖች ጋር ማክሰኞ 8፡30 ጅምሮ የማይቐርጥ መድረክ አለ፡፡ የክልል ግብረሀይል በመጀመሪያ አካባቢ በሳምንት ሶስት ጊዜ እንገናኝ ነበር፤ ሰኞ፣ሮብና ዓርብ፡፡ ስለዚህ ይህንን ያክል መድረኮች ላይ የመረጃ ልውውጥ ይደረጋል፤ ከኛ የሚፈለገውን እናቃብላለን፤ ከነርሱ ለኛ የምጠቅመንን እንወስዳልን፡፡በዚህ አግባብ ነው ለመሄድ የሞከርነው፤ ታችም ተመmሳሳ ግብረ ሀይል ለማቐቐም ነው የተሞከርው፤

የኛ ከሌላው ትንሽ ለየት የሚለው ሙሉ ጊዜውን ተተክሎ የሚሰራ ኢኦሲ ማዕከል አለ፡፡ ይህ በክልልና ዞን ያለመቆራረጥ ቅዳሜና እሁድን ጨምሮ የሚሰራ አደረጃጀት ነው፡፡ እነርሱ ደግሞ በየቀኑ ነው አፈጻጸማቸውን የሚገመግሙት፡፡ የመረጃ ልውውጣችንም እጅግ የተሳለጠበትና ያሉትን ቴክኖሎጂዎችን እንደ ቪዲዮ ኮንፈራንስ ዙም ስብሰባ ቴሌግራም የመረጃ ልውውጥ የተጠቀምንበት ነው፡፡ ሌላው ህብረተሰቡም የጤናውን ዘርፍ ጫና እንዲረዳ ያደረገ ክስተት ነው ማለት ይቻላል፡፡

ይህ ሣይታቀድ የተከሰተ ክስተት እንደመሆኑ ከችግሩ ስፋት አንጻር ለዚህ ምላሽ ሚሆን ግብዐት ከማግኘት አንጻር በምን አግባብ እየተወጣችሁ ነው?

በዚህ ሂደት ውስጥ ሁሌ እኔ ለባለድረቦቸም የሚለው ትርፍ ነገር አገኛለው ወይንም ይገኛል ብዬ ሳይሆን ማግኘት ሲገባን ያጣነውን ነው እያገኘን ያለነው፡፡ ምክንቱም ዓለም ራሱ ለጤናው ሰሴክተር አስፈላጊውን ድጋፍ አድረጎ ቢሆን ኖር ለዚህ አንዳረስም ነበር፤ ዘርፉ ሰፊ የቴክኖሎጂና ሳይንስ ፈጠራ የሚፈልግና ተለዋዋጭ ባህሪያት ያለበት ሆኖ ሳለ በቂ ትኩረት የተነፈገው ነበር፡፡ በሌላ አንጻር በርካታ ሀብት ለኒውክሌርና ለሽብር ወጥተዋል፤ ሀሳብ የነበረው ሰው ራሱን በጦር መሳሪያ ያጠፋል ዕተባለ ቫይረስ ሳይታሰብ እየጨረሰ ነው፡፡ በጤናው ላይ ያጣነቸው ነገሮች ዘርፈ ብዙ ጉዳት ይዞ መከሰቱን ነው ያየነው፡ በጤና ላይ ያለመስራት በኢኮኖሚ ላይ ያለመስራት ነው፤ ፖለቲካውንም ሁሉንም ነገር ይዞ ሊሄድ የሚችል እንደሆነ ነው የታየው፡፡ ስለዚህ ከዚህ አንጻር ሲታይ በቂ ነገር ተደረገዋል ተብሎ አይወሰድም፡፡ ስለዚህ የክልሉ መንግስትም ተገዶ መጀመሪያ አካባቢ 70 ሚሊየን ከዚያም ቀጥሎ ወደ 200 መቶ ሚሊየን ከመጠባበቂያ ወጮ አድረጎ ጥያቄ ስናቀርብ የመደበበት ሁኔታ አለ፡፡ እንግዲህ እነዚህ ለዚህ ችግር ጊዜ የተመደበው በጀት አስቀድሞም ለተገቢ በሽታ መከላከል ሥራ የሚያስፈልገውን ዝርዝር በጀት እየጠየቀ ቢሆንም በተለያዩ ምክንያት ያልተፈቀደ ጉድለት ማሟያ እንጂ ተጨማሪ አዲስ በጀት እንዳይደለ ነው የሚገነዘበው፡፡ ሁሉም ነገር ቆሟል ምን ይደረግ፤ ችግሩ አፍጦ በመምጣቱና አማራጭ በማሳጣቱና ጉዳዩ ኮቪድ በመሆኑ ምክንያት በተወሰነ ደረጃም ቢሆን ትኩረት እንዲያገኝ ተደረገዋል፡፡ እንዲያውም ሌሎች የዚህን ያህል ትኩረት ያገኘ አይደለም፡፡ የጤናው ሴክተር ምላሽ እቅድ ብቻውን ው 400 ሚሊየን ነው፡፡ ከዚህ አንጻር ገና አምንት ነው ከመቶ ሚሊየን በታች ነው፡፡

እየተከናወኑ ያሉት መርሀ -ግብሮችና ድርጊቶች የታለመለትን ግብ ከማሳካት አንጻር የተገመገመበትና በሂደት የተገኘ ውጤት አንዲሁም መሻሻል የሚገቡ ጉዳዮችም ካሉ ቢገለጽ፤

ግብ ከማሳካት አንጻር ወደድምዳሜ ለመድረስ ገና ይመስለኛል፤ በርግጥ በእሰካሁን ሂደትም ቀላል የማይባል ስራ እንደተሰራ እናምናለን፤ ተስፋ ሰጭ ነገሮች ቢሰሩም ደግሞ በሌላ ጎኑ የሰጋናቸው ነገሮች አልቀሩም፡፡ ሁሉም ነገር መጥተዋል፤ ሲመጣም ደግሞ በሥነ-ልቦና ተዘጋጅተን ለመስራት እየሞከርን ነን፡፡ አሁን ያለው ሥጋት ለምሳሌ የሰሞኑ ክስተት ኮቪድን ሙሉ ማሰራጫ ነው የሆነው፡፡ በመሆኑም የኢትዮጵያ ማእበል (wave) የባሰ ሊሆን ይችላል፡የሚል ግምት አለኝ፡፡ሀገሪቱ በዚህ ልክ ሰለባ ከሆነች ማናችንም ልናመልጥ አንችልም፡፡ የኛ የመጀመሪያ the woreste case scenario ብለን የተነሳነው እንደአሁኑ ያለውን ሁኔታ ሳይሆን በመደበኛ አካሔድ ነበር፡፡ አሁን ይህ ከታከለ ምን ሊሆን እንደሚችል በጣም ያሰጋል፡፡ የመጀመሪያ ሶስት ወር ገምግመን ሪፖርት አድረገናል፡፡ አሁን ግን ከፊታችን ያለውን ክረምት ከመምጣትና በሰሞኑ ክስተት ሰው ከቤት ተጠርጎ ወጥቶ ያለምንም ጥንቃቄ መቀላቀል ጋር ተያይዞ በጣም ከባድ ነገር ሊያጋጥመን ይችላል አግዚአብሔር ካልጠበቀን፤ ባስቀመጥናቸው የግምት እሳቤዎቻችንና የበሽታው መተላለፍ ምጣኔም ሊቀየር ስለሚችል፡፡ ተስፋ ሰጭ ነገሮችን ለማምጣት ተሞክሮ ነበር፤ እስካሁን በመጣነው ሂደት ቀድሞ ያሰብነውን ያህል ጫና አለመከሰቱ ተስፋ ስጭ እንደሆነ እየወሰድን የነበረን የላቦራቶሪ ምርመራ ውስንነት እንደተጠበቀ ሆኖ፡፡ በርግጥ የላቦራቶሪ ምርመራችን ሁሉንም ህብረተሰብ ተደራሽ ማድረግ ነበረበት፡፡ ስለዚህ አሁን የተፈጠሩት ህዝባዊ እንቅስቃሴዎች ለበሽታ ስርጭት ያላቸው አስተዋጽኦና ቀድሞ ከነበረው ጉድለት ጋር ታክሎ ባሁኑ ሰዓት ያለምነውን ግብ አሳክተናል ለማለት እቸገራለሁ፤ ምናልባት ይህንን በመጪው መስከረም ወር አካባቢ ብንገመግም ይሻላል ብዬ አምናለሁ፡፡

በታችኛው መዋቅር ውይይት ሂደት የተነሳው የኮቪድ ምላሽ ባሁኑ ጊዜ ከመጀመሪያው አንጻር ሲታይ እየተቀዛቀዘ ነው ይላሉ፤ በዚህ አንጻር የርስዎ እይታ ምን ይመስላል?

በትክክል ይገልጻል የታችኛው ግምገማ መሬት ላይ ያለውን እውነታ፤ እንደመደበኛ የዚህ ስራ ግምገማ አካል እንደጤና መምሪያ ዘውትር ማክሰኞ ከዞኖች ጋር በዙም ስብስባ እንገመግም ነበር፤ የባለፈው ማክሰኞ እለት እኛም ይህንን ሁኔታ ተገንዝበን የዞን አስተዳዳሪዎች የጤና መምሪያ ሀላፊዎች ባሉበት የክልሉን ግብረ ሀይል ይዞ የክልሉ ርዕሰ መስተዳድር ናቸው ሁኔታውን የገመገሙት፡፡ የግምገማው ዋና ጭብጥ የነበረው እንቅስቃሴያችን ተዳክመዋል ፤ ስለዚህ ማጠናከር አለብን የሚል ነበር፡፡ይህን ስራ ወደ መተው ሄደናል፤ በሳምንት ምንም ሳነሰላች ሶስት ቀን ተገናኝተን የምንገመግም አካላት አሁን አሁን አንድ ጊዜ ተገናኝተን መገምገም እያቃተን ነው፤ ስለዚህ እንደገና አንስተን እንያዘው ብለናል፤ በመሆኑም የታችኛው መዋቅር እይታም ትክክል ነው፡፡

ይህ ደግሞ በህብረተሰቡ ዘንድም በተመሳሳይ ሁኔታ እየተንጸባረቀ ነው፡፡ ትዝ ይለኛል የመጀመሪያ ኬዝ በክልላችን ሀላባ ላይ ሲገኝ በተከታታይ ሶስት ቀን ከተማ ውስጥ ሰው አይወጣም ነበር፤ ወጠታችሁ ተንቀሳቀሱ ተብሎ ሁሉ አይወጡም ነበር፤ ዛሬ ግን በሽታው በየአካባቢው መሰራጨቱ እየተነገረ በሞቱት አስከሬኖች ላይ ሳይቀር በሽታው መገኘቱን እየሰሙ በተቃራኒው መዘናጋት አለ፡፡ ይህ በአመራሩም ሆነ በህዝቡ የሚገለጽ ስለሆነ ግምገማው ትክከለኛ ነው፡፡ እንግዲህ እኛም ይህንን ተገንዝበን ጉዳዩ እንደገና በትኩረት ሊያዝ ይገባል ያልነው፡፡ አንዳንዱ እኛጋ አልደረሰም በማለት ሌላው በሽታው በአንዳንዶች ምልክት ሳያሳይ የሚቆይ በመሆኑና 91 በመቶ እንደሀገር ስንገመግም ምንም ዓይነት ምልክት ያለማሳየታቸው ሊያዘናጋን አይገባም ነበር፤ ግን በዚህም ህብረተሰቡ አይ ፡ይህ ለካ ከጉንፋንም ያነስ ነው እንዴ ወደማለት ይህን ተከትሎ መቀዛቀዝ አለ፡፡

የኮቪድ -19 ወረርሽን ለመከላከልና ለመቆጣጠር በተደረገው ርብርብ ሂደት የተገኘ አዎንታዊ ገጽታ ካለ ቢገልጹልን?

እንደመልካም አጋጣሚ የማየው በጤናው ሴክተር ላይ የነበረውን የተንሸዋረረ አመለካከት እንዲስተካከል ማድረጉ፤ሴክተሩ ምን እንደሚፈልግ አስፈጻሚውና ፖሊሲ አውጭው በሚገባ እንዲረዳ አድረጎታል፡፡ እና ራሳችን ጤና ተቋማት ግንባታ እንጂ የተሟላ አገልግሎት በሚሰጥ መልኩ አደራጅተን አናውቅም፡፡ ለምሳሌ፡- ላቦራቶሪ ለህክምናው ወሳኝ ሂደት አንደሆነ አስበን ማሟላት አንደሚገባ አልተንቀሳቀስንም ነበር፡፡ በዚህ ሂደት ሁሉ የጤናው ሴክተር ተገቢ አቴንሽን እንዲያገኝ መደረጉ ነው፡፡ ሁለተኛው ኮቪደ-19 መጀመሪያ ምዕራባዊንን ነው የመታው፤ ከኤሺያ ተነሳና ከፍተኛ ዱላ በምዕራባውያን ላይ ነበር፡፡ በተለምዶ የኛ ገንዘብ (የጤናው ዘርፍ) ተብሎ የሚታወቀው ከምዕራባውያን በድጋፍ የሚመጣ ነው፡፡ ከምዕራቡም ወደ አሜሪካ ያደላ ነው ብዙ ነገር ቢታይ ማለቴ ነው፡፡ እነርሱ ሲመቱ ትንሽ ጊዜ ወደላይ ጮሁና ሀብተ ማሰባሰብን ከሀገር ውስጥ ማፈላለግ የተገባው፡፡ አንዳንድ ስራዎችን አጥፈው ለዚህ አገልግሎት ማዋል፤ ወደህብረተሰብ ውስጥ ገብተው ሀብት ማሰባሰብ ስራ መጀመር፤ በመሆኑም አስቀድሞ ባብዘኛው በውጭ ሃይሎች ጥገኛ የሆንነው ሙሉ በሙሉ ደሃ ስለሆንን ነው ወይስ የግንዛቤ ክፍተት ስላለ ፡፡ ለምሳሌ ክትባት ሙሉ በሙሉ በውጭ ድጋፍ የሳንባ ነቀርሳ ህክምና የወባ በሽታ ህክምና ሁሉ ሙሉ በሙሉ በውጭ ድጋፍ መሆኑ በጥንቃቄ ማየት እንደሚያስፈልግ ሁሉ በዚህ ጊዜ ወደሀገር በቀል ምንጭ እንዲናይ ማድረጉ፤ የጤናው ሴክተር ዘላቂነት እንዲኖር ልክ እንደማህበረሰብ አቀፍ የጤና መድህን ዓይነት ለሌሎች ጤና ሴክተርም የሀገር ውስጥ ገቢ ማመንጨትን መመደብ አስፈላጊነት ትኩረት ያገኘበት ትልቅ ትምህርት የተወሰደበት ነው፡፡ ከቴክኖሎጂ አጠቃቀም አንጻር አዳዲስ አሰራሮችን ከመከተል አንጻር እንደዙም የስብሰባ ቴክኖሎጂ በስፋት እንድንጠቀም የተደረገበት፡ ኢኦሲ አሰራርን በሚገባ እንድንጠቀም የተደረገበት፤ ከዚህ በፊት ኢኦሲ ይባል እንጂ እንደዚህ ጊዜ በተደራጀና በተጠናከረ ሁኔታ ተመርቶ አያውቅም ነበር፡፡

ክልል ማዕከል

ቁልፍ መረጃ ሰጭ ቁጥር 4. የትምህርት ቢሮ

የክልሉ ትምህርት ቢሮ ከኮቪድ-19 በሽታ ጋር ያለው ቁርኝት ምን ይመስላል? ከታችኛውና ላይኛው መዋቅር ጋር የተፈጠር የስራ ግኑኝነት፤ ከማስተባበርና በአጋሪነት ከመስራት አንጻር፤ ተቋሙን ለተልዕኮ ብቁ ከማድረግ አንጻር ምን ይመስላል?

እንግዲህ ኮቪድ -19 ወረርሽኝ ከተከሰተበት ጊዜ አንስቶ የመጣንበትን እንደመግቢያነት ለማንሳት ያክል እውነቱን ለመናገር የትምህርት መዋቅር እንደዚህ ጊዜ የተፈተነበት አጋጣሚ አልነበረም፡፡ እንደትምህርት ዘርፍ ምን አደረግን የሚለውን ብናገር እንዴት ነው የሆንነውና የመጣነው የሚለውን ለማንሳትና በዚህ ሂደት ውስጥ የባለድርሻ አካላት በበጎም ሆነ በአሉታዊ ጎን የሚነሳ ተጽዕኖ ምን እንደሆነ ለማየት ትምህረት ይቁም ተብሎ ከተገባ በኋላ በክልላችን ያሉትን 127 የተለያዩ ዓይነት የመማሪያ መጻህፍት መካከል 50 ዓየነት ብቻ ናቸው አንድ ለአንድ ተደራሽ ሆኑት፤ሌሎች በጋራ ነው የተሰጡት፤ ስለዚህ ሁሉም ተማሪዎች ሁሉንም መጻህፍት ወደቤቱ ይዞ አይደለም የሄደው፤ አጥና ለማለትም የተቸገርንበትና ያኔ ሲወሰን በቅርቡ ትመለሳላችሁ በሚል እሳቤ ስለነበረ፤ግን ተማሪዎች በቤታቸው በሚቆዩበት ጊዜ መጠቀም ስላለባቸው በተማሪዎች ቁጥር መጽሀፍ ተደራሽ ለማድረግ ያ የተባለው 15 ቀን ሳያልቅ የትምህርት ቢሮ አዲስ የቴሌግራም ቻናል ከፍተን መጀመሪያ የራሳችን ፈቃድ ያላቸውንና በኛ የታተሙትን መጽሀፍት በሙሉ ጫንንላቸው፤ ቀጥሎ ደግሞ በትምህርት ሚኒስቴር በኩል የታተሙትን አስፈቅድን ካወረድን በኋላ ባጠቃላይ ከ1ኛ -12ኛ ክፍል ያሉትን መጽሐፍትን በኛ ቻናል ላይ ጭነን እንዲጠቀሙ እያደረግን ነን፡፡ሆኖም ግን ስናየው ካሉን በብዙ ሚሊየን ከሚቆጠሩት ተማሪዎች መካከል ከ15-20 ሺህ የሚሆኑ ብቻ ተጠቃሚ መሆናቸውን ነው፡፡ በርግጥ በቤተሰብ ደረጃም የሚጠቀሙትን ካካተትን የተማሪዎች ቁጥር ከፍ ሊል ይችላል፤ባጠቃላይ የተማሪ ቁጥር ከ0-12 ያሉት እስከ አምስት ሚሊየን የሚጠጉ ናቸው፡፡ ይህንን ከፍተኛ የሆነ ክፍተት አይተን ሁለተኛ ዙር 15 ቀን ሳደርስ ለማድረግ የሞከርነው፡፡ተማሪዎች እቤት ሆነው ትምህርት እንዲከታተሉ የትምህርት በሬዲዮ በሶዶ ሚዛንና ይርጋለም ላይ ያሉት ጣቢያዎች በሁሉም ቦታዎች የሚደመጡ ናቸው፡፡ይህ ማለት ተደራሽነትን በሚመለከት ረገድ ማለት ነው፤ ከ1-4 ላሉት ክፍሎች ከዚህ በፊት የተቀረጹት የትምህርት ዓይነቶች አሉ፤ ነገር ግን እኛ ያሰብነው ከሰባት በላይ ላሉት ነው፤ ከ7-12 ላሉት ለሁሉም አምስት አምስት የትምህርት ዓይነቶች ተመርጠው ሀዋሳ ከተማ ውስጥ ካሉት መምህራን መርጠን እዚሁ ስቱዲዮ ፈጥረን ሪከርድ አድርገን ለትምህርት በሬዲዮ ፕሮግራም እንዲተላለፍ ሰጥተናቸዋል፡፡ምንም እንከዋን ሰፋ ላለ የክልላችን ነዋሪዎች ቢደርስም ችግር አጋጠመን፡፡ ስንጠቀም የነበረው ቴክኖሎጂ ጊዜ ያለፈበት አጭርና መካከለኛ መገዶች መሆኑ ነው፡፡ባብዘኛው ሰው አሁን እየተጠቀመ ያለው ኤፍ ኤም ነው፡፡ ሁሉም በሚባል ደረጃ በሞባይል ስልኮች እና በጂ-ፓስ እና በመሳሰሉት የሚደመጠው ኤፍ ኤም ነው፡፡ ገጠር ሄደን ልጆችን ስንጠይቃቸው የለም በስልክ ልናዳምጥ አልቻልንም ይላሉ፡፡ባብዘኛው በድሮ ሬዲዮኖች ብቻ ነው አጭርና መካከለኛ ሞገድ ማግኘት የሚቻለው፡፡ ይህንን ችግር ካየን በኋላ ከሌሎች ጋር ትብብር ማድረግ የጀመሪነው፡፡ከደቡብ ሬዲዮና ቴሌቭዥን ድርጅት ጋር ተነጋግረን ከሰኞ እስከ ዓርብ ድረስ ከሰዓት በኋላ 11 ቅርንጫፎች አየር ሰዓት ስላላቸው ወጪያቸውን ሸፍነን ማሰራጨት ጀመርን፡፡ ይህ አቀራረብ ተደራሽነትን ከበፊቱ እየተሻሻለ መጥተዋል፡፡በከተማ አካባቢ ላሉት በተጨማሪ በቴሌቭዥን በፕላዝማ የተወሰኑትን ትምህርቶችን በተለይም ከላቦራቶሪ ጋር ተያያዥነት ያላቸውን በዪኒሴፍ ድጋፍ እኛ ወጪያቸውን ሸፍነን እንዲሰራጭ እያደረግን እንገኛለን፡፡በዚህ ሂደት ከብዙ ባለድርሻ አካላት ጋር በትብብር እንሰራለን፡፡ከታችኛው መዋቅር ጋር በመጀመሪያ አካባቢ ክፍተት ነበር፤ ትምህርት ተቋርጠዋል ሲባል ትምህርት ቤት ዘግቶ የመውጣት አዝማሚያ ሁሉ ነበር፤ ከትምህርት ሚ/ር ጋር ያለን ግኑኝነት ሳይቋረጥ በዙም ባጋጣሚ ሚኒስትራችንም ከሳይንስና ኤኖቨሽን ሚኒስቴር ስለመጣ ቀደም ብሎ ነው በነገራችን ላይ ይሄ ዙም ቴሌኮንፈራንሶችን ቪዲዮ ኮንፈራንሶችን ያስጀመረው፤ በመሆኑም ምንም ሳይቆራረጥ ነው የቀጠለው፡፡ወደታች የነበረው ግኑኝነት መጀመሪያ አካባቢ አስቸጋሪ ነበር፤ ዙምንም ለማስለመድ ጊዜ ወስዶብን ነበር፤በተወሰነ ደረጃ ወደ አይሲቲ ማዕከልም እየሄድን ቪዲዮ ኮንፈራንስ ከማድረግ ስንሞክር ነበር፡፡

በዚህ ሂደት ከሌሎች አጋሮች ጋር ወርደን የትምህርት በሬዲዮ ስርጭት ይደመጣል ወይ የሚለውን ከመሞከር አልፎ ሌሎች አንደግብርና ቢሮ ለስራ ጉዳይ መስክ በሚወጡበት ወቅት በሚሄዱበት ቦታ ሁሉ ሥርጭቱ ስለመደመጡ እንዲያረጋግጡ እያስደረግን ነው፡፡ ከክልሉ ምክር ቤት ጋር በቋሚ ኮሚቴ የመስክ ምልኬታ ወቅትና በየደረጃው ባሉት የቋሚ ኮሚቴ በኩል እንዲያረጋግጡ የማድረግ ስራ ተሰርተዋል፡፡ከነርሱ ግብረ መልስ እንወስዳለን፡፡ምንም ከሌለበት እንዲከታተሉ አድረግናል እንጂ ሙሉ ሸፍነናል ማለት አንችልም፤ እንደ አርብቶ አደር አካባቢና አንዳንድ የክልላችን አካባቢዎች ለትምህርት በሬዲዮና ቴሌቭዥን ብቻ ሳይሆን ባጠቃላይ ከመረጃ ተደራሽነት የራቁ እንዳሉም በዚህ አጋጣሚ የታየበት ሁኔታ ነው፡፡

የትምህርት ሴክተሩ በዐቢይ ግብረ-ሀይል ደረጃ ምን ይመስላል?

ባጋጣሚ እኔ የማህበራዊ ክላስተር ሰብሳቢ ስለሆንኩ የግብረ- ሀይሉ ምክትል ሰብሳቢ ነኝ፡፡ እንደተቋም ግን እንደሌላው የካቢኔ አባል እንሳተፋለን፡፡በዐቢይ ግብረ- ሀይል ለትምህርት ቢሮ ተልዕኮ ተሰጥተዋል፡፡ የትምህርት ተቋማትን ለኳራንታይን ለለይቶ ማቆያ እና ለህክምና ማዕከልነት ማዘጋጀትን በሚመለከት ነበር ነገር ግን አስቀድሞ ከላይ ከዩኒቨርሲቲዎችና ኮሌጆች መጀመር አለበት ስለተባለ እንደተቋም የተሰጠን አሳይመንት እሱን ማስተባበር ነው፡፡ ይህ ተልዕኮ እንደተቋም ይሁን እንደግለሰብ ለመለየት እቸገራለሁ ግን በክልሉ ያሉት አስሩም ዩኒቨርሲቲዎች ለማቆያ የሚሆን ቦታ እንዲያዘጋጁ ሀላፊነት ስንሰጣቸው ቀደም ብሎ የጀመሩት ሶስት ዪኒቨርሲቲዎች ነበረ ግን አጠቃላይ ሁሉንም ዩኒቨርስቲዎችን ያስተባበርን እንደተቋም እኛ ነን፡፡ ሌላው በግብረ-ሀይል የነበረንና እንደተቋም የነበረን ከዚህ ወረርሽን ጋር በተያያዘ ገቢያቸውን አጥተው በጎዳናና መንገድ ዳርና ዳር ያሉትን ህጻናትና ወጣቶች አንስተን ከተማ ውስጥ ባሉት ትምህርት ቤቶች ውስጥ እንዲያርፉ አድርገናል፡፡ ለመጠቀስ ያህል፡- ዲላ፣ ሀዋሳ፣ ወላይታ፣ አርባምንጭ፣ ሆሳዕና እነዚህ ትልልቅ ከተሞች ላይ ትምህርት ቢሮ ለምገባ ፕሮግራም ያቀደውን በጀት በማዞር አግልግሎት እየሰጠ ይገኛል፡፡ በየዓመቱ ሁለተኛ ቴርም ላይ ለተማሪዎች የሚደረገውን ምገባ ፕሮግራም እንደጀመርን ከአንድ ወር በኋላ ይህ ችግር ሲከሰት ወደዚህ ነው ያዞርነው፡፡ለምገባ ፕሮግራም እህል የምንገዛው ከአርሶአደር ህብረት ሥራ ዩኒየን ነው ፡፡ከሲዳማ አካባቢ ሲዳማ ኤልቶ፣ጉራጌ ስልጤ አካባቢ ስልጤ ቢልቅ፤ ወላይታ ዳውሮና ጎፋ አካባቢ ዳሞታ ዩኒየን እንደዚሁም አርባምንጭ አካባቢ ከነዚህ የአርሶኣደር ዪኒየኖች ያላቸውን እህል በየሶስት ወር በምንሰበስብበት አግባብ የመጀመሪያውን ሩብ ዓመት እህል በየአካባቢያቸው ለተጎዱት ህጻናት ቤተሰቦች ለምሳሌ ሀዋሳ አካባቢ ሜሪ ጆይ ሀዋሳ ከተማ አስተዳደር አርባምንጭ ሶዶና ሆሳዕና ላይ በየከተማው ባሉት ትምህርት ቤቶች ውስጥ ለተቋቋሙት ማዕከላት (ማእከሉን እኛ ብቻ ሳንሆን የሴቶች ህጻናትና ወጣቶች ቢሮ ሠራተኛና ማህበራዊ ጉዳይ ቢሮ) አረጋዊያንን ህጻናትን በመመልመል እነርሱና የድርጅት ማዕከልም ይሳተፋል፡፡ ለነዚህ ሰዎች የሚቀርበውን እህል፡- በቆሎ ስንዴ ቅንጨ ዜይት ጨው በቢሮ ተገዝተው የተቀመጠውን ሁለት ዙር የማድረስ ሥራ እኛ አከናውነናል፡፡

የኮቪድ-19 ወረርሽኝ ምላሽ ሂደት ከቅንጅታዊ አሰራር ጋር ተያይዞ ምን ዓይነት አስተዋጽኦ አድርገዋል ይላሉ? ለቀጣይስ ምን መልካም ተሞክሮ ሊወሰድ ይችላል? የተለያዩ ግብረ ሀይሎች አሉና እነዚህን ሁሉ አቀናጅተው ለተልዕኮ በማሰማራት የታለመላት ዓላማ እንዲሳካ ከማድረግ ረገድ የክትትልና ግምገማ ሥርዓት ምን እንደሚመስልና በሂደት የታዩት አዎነታዊና አሉታዊ ገጽታዎችም ተገምግሞ ከሆኑ ቢገለጽ፤

በተወሰነ መልኩ ትምህርት የምናገኝበትም አለ፡ በተወሰነ መልኩ ቻለንጅድ የሆንንበትም አለ፡፡ በመጀመሪያ ደረጃ ግብረ-ሀይሉ በክልል ማዕከል ተቋቋመ፤ ይህ የተቋቋመው ግብረ-ሀይል ሁሉንም ነገር ብቻውን መስራት ስለማይችል ተቋማትን መሰረት ያደረገ ንዑሳን ኮሚቴዎች ተቋቋሙ፡፡ ስድስት ሰባት የሚሆኑ፡- ንግድን በተመለከት የሚያጋጥሙትን ችግሮችን ለመቅረፍ በንግድና ገበያ ልማት ቢሮ የሚመራ፤ጤናን በሚመለከት ዋናው የመከላከልና ቁጥጥር ሥራ የሚመራ በጤና ቢሮ የሚመራ፤ኮሙኒኬሽን በሚመለከት የህዝብ ግኑኝነት ኮሚቴ ማረሚያ ቤትን ፀጥታን በሚመለከት ሰላምና ፀጥታ ቢሮ በሚመራው ኮሚቴ የሀብት ማሰባሰብን ሥራ የአደጋ ስጋት ሥራ አመራርና ፋይናንስ ቢሮ የሃይጂንና ሳኒቴሽን ሥራ በሚመለከት በውሀ ማዕድንና ኢኔርጂ ልማት ቢሮ የሚመራ ኮሚቴ ተጋላጮች ሊየታ ሴቶች ህጻናትና ወጣቶች ቢሮ ጋር ሰራተኛና ማህበራዊ ጉዳይ ቢሮ የሚመራው በንደዚህ ዓይነት ሁኔታ ኮሚቴ አዋቅረን ሁሉም ኮሚቴ በየሳምንቱ ሪፖርት ያመጣል፡፡ ዐቢይ ግብረ-ሀይል መጀመሪያ አካባቢ ስራው መስመር እስከሚይዝ ድረስ በሳምንት ሰኞ ራቡዕና ዓርብ ሶስት ጊዜ በተከታታይ ኦንላይን እየተገናኘን እየገመገምን ቆይተን ወደኋላ ላይ ስራው መስመር ውስጥ ስገባ እንዲሁም ስራው ከማዕከል ወጣ ብሎ መስራትንም ሲጠይቅ በሳምንት ሶስት ጊዜ የምንገናኝበት ወደ አንድ ጊዜ ዓርብ የተራዘመበት ሁኔታ ነበር፡፡ እንዲሁም የግድ ቀጠሮ መጠበቅ ብቻ ሳይሆን እንደሁኔታው እየተገናኘን የምንገመግምበት አግባብ ነበር፡፡ በተለይ ከንግድ ጋር ያለው ኮሚቴ ከጸጥታ ጋር ያለው ኮሚቴ ከዋሽ ጋር ያለው ጤናውም ራሱ ጥሩ ሰርተዋል የሚል አስተያየት አለኝ፡፡ ሀብት የሚያሰበስብ ኮሚቴ እና ሌሎች ደግሞ አካባቢ ብዙም ተሳክተዋል የሚል ድምዳሜ የለኝም፡፡ በዋናነት ከጤናው ጋር በተገናኘ ብዙም እንደሌሎች ክልሎች የኛ የተዋጣ ነው የሚል ግምት የለኝም፤እንደክልል የተሞከረ ነገር አለ ግን ካየሔው እስካሁን የምርመራ ላቦራቶሪ ሶስት ነው ያለው ነገር ግን የክልሉ አስተዋጽኦ ሊባል የሚቻለው እዚሁ ያለችው አንዷ እንጂ አሁን ወላይታ ያለው ቢሮ ምንም ያደረገው ነገር የለም፤ የአርባምንጭንም በራሳቸው ጥረት እንደሆነ ፤ ለዚህም ዩኒቨርሲቲዎች በራሳቸው ጥረት ሲያደርጉ በርካታ አደናቃፊ የሆኑ ብዙ ነገሮች ነበሩ፡፡ ከዚህ ከኛ ሳይድ ኮሚቴ ከሚትለው ከማዕከል ጀምሮ፤ምንም ይሁን መጀመሪያ በዚህ መልኩ ተቀናጅተን መጀመራችን ጉልበት ሆኖልናል፡፡ ከቅንጅት አንጻር አሁንም ቢሆን በቂ ቅንጅት ተፈጥሮአል የሚል እምነት የለኝም፡፡ ጣልቃ መገባባት አለ፤ የስራ ድንበር ያለማወቅ አለ፡፡ለሥራው የሚያስፈልግ ሀብት በመመደብ ረገድ ጉድለት አለ፤ቅንጅታዊ ሥራን ተቋማዊ ከማድረግ ይልቅ ግለሰባዊ የማድረግና የመቃቃር ሁኔታ ይታይበት ነበር፤

ቅንጅታዊ ሥራ ሲጀመር በዐቢይ ኮሚቴ ደረጃ በሂደቱ ላይ ተሳታፊ ተቋማትና ንዑሳን ግብረሀይሎች ሃላፊነትና ተጠያቂነትን በግልጽ አስቀምጦ ግምግሞ ተጠያቂነትን ከማስከተል አንጻር የታቀደ ጉዳይ ካለ፡

ከዕቅድ ለመነሳት በመጀመሪያ ዕቅዱ ላይ ችግር ያለ አይመስለኝም፤ሥራዎችን ተቋማዊ አድረጎ ከመውሰድ ይልቅ ግለሰባዊ አድርጎ የማሳየት ለምሳሌ አንድ ግልጽ ምሳሌ ሊስጥህ ሀብት የማሰባሰብ ሥራ አቶ ጥላሁን የእርሻና ተፈጥሮ ሀብት ልማት ቢሮ ሀላፊ ስለነበሩ አደጋ ስጋት ስራ አመራር ስለሆነ ሀብቱን የሚያመጣው ይህ ተቋም ደግሞ በእርሻና ተፈጥሮ ሀብት ቢሮ ሥር በመሆኑ የተሰጠውን ሃላፊነት አሁን ቦታውን ለቆ ወደሌላ ቦታ ሲሄድም ሥራውን ይዞ መሄድ ዓይነት፡ በተግባር ሥራውን በቅርበት የመከታተል አጋጣሚ ሳይኖር ሥራውን ይዞ መሔድ ሌሎች ተመሳሳይ ሁኔታዎችም አሉ፤ ይህን ባጋጣሚ ፊት ለፊት ስለመጣ ነው፡ የትራንስፖርት ጉዳይ እንዲመራ በህጋዊ አግባብ የተቋቋመው ተቋምና የሚመራው አካል በዐቢይ ኮሚቴ ውስጥ እያለ የትራንስፖርት ክልከላ ተቋሙ ሳያውቅ በሰላምና ጸጥታ ቢሮና በርዕሰ መስተዳድር ውሳኔ የተሰጠበት አጋጣሚዎችን ማንሳት ይቻላል፡፡ ኮሚቴ ደረጃ ሣንነጋገር ያስቆማል ደግሞ አስቸጋሪ ሁኔታ ሲያጋጥም ያስጀምራል፡፡ ይህ የሚያሳየው በተቋማዊ አሰራርና በግብረ ሀይል በተቀመጠው አግባብ ሳይሆን በተወሰኑ ግለሰቦች ፍላጎት የሚደረግ አሰራር ይታያል፡፡ከህዝብ ግኑኝነት ሥራ አንጻር የመንግስት ኮሙኒኬሽን ቢሮ የደቡብ ሬዲዮና ቴሌቭዥን ድርጅት የጤና ቢሮ የመንግስት ኮሙኒኬሽን ዳሬክቶረት በየደረጃው እያሉ እኔ አንድም ቀን የጤና ቢሮ ኮሙኒኬሽን ዳይሬክተር ወይንም የክልሉ መንግስት ኮሙኒኬሽን ቢሮ ሃላፊዋ ከአንድ ጊዜ ውጭ ወጥተው መረጃ ሲሰጡ አላየሁም ግን ሁል ጊዜ በመገናኛ ብዙሀን እየወጣ የሚሰጠው ሌላ አካል ነው፡፡ በመሆኑም ችግሩ የዕቅድ ሳይሆን ሥራውን የግል አድረጎ የመውሰድ ነው የሚመስለኝ፡፡ ለምሳሌ እኔ በግሌ የዚህ ዐቢይ ግብረ-ሀይል ምክትል ሰብሳቢ ሆኜ አንድም ቀን ትራንስፖርት ይቁም ሲባልም ይቀጥል ሲባልም አልሰማሁትም፡፡ ጸጥታ ቢሮ ከድርጅትና ርዕሰ መስተዳድር ጋር ቁጭ ብሎ የወሰኑበት ነው፡፡ እኛም መግለጫውን በፌስ ቡክና ሌሎች የመገናኛ ዘዴዎች ከህዝብ ጋር እኩል ሰምተን ነው ፡ በመሆኑም ቅንጅታዊ አሰራር አለ ለማለት የሚያስደፍር ነገር የለም፡፡

ኮቪድ -19 ወረርሽን በመከላከልና ቁጥጥር ሂደት ውስጥ የተገኙት መልካም ተሞክሮች ካሉ፡-

በርካታ ሊቀሰሙ የሚችሉ ልምዶች አሉ፡ በዚህ ሂደት ውስጥ አንደኛ ካልነበረበት ቅንጅት ወደ መከሰት የመጣው፤ አስቀድመ ያልኩት ጉድለት እንዳለ እንጂ ምንም የለም ማለት አይደለም፤ ወደላይና ወደ ታች የሚደረጉት ግኑኝነቶች ለምሳሌ እኔ የማነሳቸው ሀብትን በውጤታማ መንገድ መጠቀም የአሰራር ድግግሞሽን ማስወገድ ወይንም መቀነስ የአካላዊ ወይንም ፊት ለፊት በመቀመጥ የሚደረገውን ስብሰባዎችን መቀነስ፤ በዚህም ሀብትን በቁጠባ መጠቀምና ወጪ መቀነስ፡- ለምሳሌ እንደትምህርት ቢሮ አንድ ስብሰባ ታችኛውን መዋቅር ጠርተን ለማድረግ ከስድስት መቶ ሺህ ብር በላይ ይጠይቅ እንደነበር ግን አሁን ተመሳሳይ መልእክት በዙም ያለምንም ወጪ ማስተላለፍ መቻላችን፤ ያለንን ሀብት ቴክኖሎጂና ሌሎች አስተሳሰቦችን እንዲንጠቀምና እንዲናቀናጅ አድረጎናል፡፡ እንግዲህ ልምዱን በተገቢ ወስዶ ሥራ ላይ ማዋል ምን ይሁን ባላውቅም ቅድም ያነሳናቸውን የስራ ሀላፊነት መጣረዝን የመቀነስ ዕድል ይፈጥራል የሚል አስተያየት አለኝ፡፡ ምን ምን ሥራዎች እንደሚጣረዙ ተልዕኮች ጭምር፡-ለምሳሌ ቴክኒክ ሙያና ሳይንስና ኤኖቨሽን ምን ላይ ምን ላይ ይጣረዛሉ ምን ላይ ምና ላይ ይለያያሉ የሚለውን እንድናይ አጋጣሚ የፈጠረ ነው፡፡ እንዲሁም ማን ከማን ጋር በምን ዓይነት ሁኔታ በጋራ ሊሰራ ይችላል የሚለውንም ለማየት ዕድል ፈጥሮአል፡፡ ለምሳሌ ትምህርት ቢሮ ከሳይንስና ኤኖቨሽን ቢሮ ካለው የአይ ሲ ቲ ጋር በምን ተባብረን መስራት እንዳለብን ያሳየ ነው፡፡ በየተቋሙ ያለው አይ ሲ ቲ ከሌሎች ሥራ ሂደቶች ጋር ተቀናጅተው የመስራት ዝንባሌ በምን ሊያግዝ እንደሚችል ሌሎችም እንደዚያው፡ ሀብት የት የት እንዳለ በጋራ እንድናይ ረድቶናል፤ ሥራዎች ባንዱጋ በሀብት እጥረት ታነቆ ሌላውጋ ሀብት ተደብቆ እንዳይቆይ መልካም አጋጣሚ ፈጥሮልናል፡፡ ሌላው ምናልባት ሌላ ጊዜ እንጠቀም አንጠቀም ባላውቅም በግዥ ሥርዓት የተፈጠረው ውጤታማነት ሊነሳ የሚገባ ነው፡፡ በልዩ ውሳኔ ባጭር ጊዜ ግዥ እንዲፈጸም መደረጉ በመሆኑም ብዙ ትምህርት የተወሰደበት ነው ማለት ይቻላል ዋናው ነገር እነዚህን ልምዶችን መዝግቦ ማስቀጠል መቻል አስፈላጊ ነው፡፡

ቁልፍ መረጃ ሰጪ ቁጥር አምስት ሴቶች ህጻናትና ወጣቶች ጉዳይ ቢሮ

የናንተ ተቋም የኮቪድ -19 በሽታ መከላከልና ቁጥጥር ሥራ ጋር ያለው ቁርኝት ምን ይመስላል?

ከታችኛውና ላይኛው መዋቅር ጋር የተፈጠር የስራ ግኑኝነት፤ ከማስተባበርና በአጋሪነት ከመስራት አንጻር፤ ተቋሙን ለተልዕኮ ብቁ ከማድረግ አንጻር ምን ይመስላል?

የኮቪድ -19 በሽታ ስናነሣ እንደሴቶች ህጻናትና ወጣቶች የምናየው 75 በመቶ የሆነውን የህብረተሰብ ክፍልን በመያዝ ነው፡፡ እንደክልል በርዕሰ መስተዳደሩ በሚመራው ዐቢይ ግብረ-ሃይል ውስጥ አባል በመሆን ለኮቪድ -19 መከላከልና ቁጥጥር ሥራ የበኩላችን እያበረከትን እንገኛለን፡፡ ይህን ሥራ አብረን ስንሰራ በዐቢይ ኮሚቴ ስር ንዑስ ኮሚቴ አለ፤ በዚያ ስር ደግሞ የንዑስ ንዑስ ኮሚቴ አለ፡፡ እንደየኮሚቴው ባህሪይ የስራ ዕቅድ አዘጋጅተው እየተንቀሳቀሰ ነው፡፡ የኛ ቢሮ በሀብት ማሰባሰብ ኮሚቴ አባልና በዚያው ስር ባለው የአላባሳት ምግብና የመሳሰሉት አቅርቦቶችን የሚመለከት ንዑስ ኮሚቴ ሰብሳቢ በመሆን እየተሳተፍን ነው፡፡ ይህ አደራጃጀት ከክልል ጀምሮ እስከ ቀበሌ ድረስ ነው፡፡ በቀበሌ ደረጃ የኛ አደረጃጀት በሴቶች ማህበር አመራሮች ስላሉ መዋቅሩ እስኪዚያ የሚደርስ ነው፡፡ ኮቪድ የአንድ ሴክተር ሥራ ስላልሆነ እኛም ከሌሎች ባለድርሻዎች ጋር ተቀናጅተን የጋራ ሃላፊነትንና እንዲሁም የሴክተር ድርሻችን በተሳተፍንበት ንዑስ ኮሚቴና እንደመዋቅርም ባለን አደረጃጀት እየተወጣን እንገኛለን፡፡ ለማህበራዊና ኢኮኖሚያዊ ችግርች ይበልጥ ተጋላጭ የሆኑትን የህብረተሰብ ክፍሎች፡አረጋዊያን የጎዳና ተዳዳሪዎች ሴትኛ አዳሪዎችና የአካል ጉዳተኞች ለበሽታውም ይልቅ የሚጋለጡት መደገፍ ስላለባቸው በክልላችን ርዕሰ መስተዳድር አስተባባሪነትና ሌሎች በማሳተፍ የሚደገፍበት ሥራ እየተሰራ ነው፡፡ የደሀ ደሃ ሆነው በሴፍትኔትም ያልታቀፉትን ለይቶ ለድጋፍ ለማቅረብ የቢሮ ሃላፊን ጨምሮ ምክትል ቢሮ ሀላፊዎችና ሌሎች የስራ ሃላፊዎች ተሳትፎ መልምለዋል፡፡ እስካሁን ያለው በክልለ ደረጃ በግብረ,ሀይል በኩል በታቀደውና በንዑሳን ግብረሀይሎች ደረጃ መከናወን የሚገቡ ሆነው በሴክተር መስሪያ ቤት ደረጃ ከሚኒስቴር መስሪያ ቤት በኩል ዕቅድ ወርደውልን እኛም በኛ ደረጃ ከልስን የምንተገብረው ደግሞ አለ፡፡ ይህንን የምንተገበርበት ዐቢት ኮሚቴን በክላስተር ደረጃ የተደራጀ አራት ንዑሳን ኮሚቴዎች አሉን፡፡ እቅዳችን እስከዳይሬክቶረት ደረጃ ና አስከታችኛው መዋቅር አውረደን አፈጻጸሙን ቤደረጃው እየገመገምን እንገኛለን፡፡ የክትትልና ግምገማ አግባባችንም በቢሮ ደረጃ ከአይሲቲ መዋቅር ጋር በመደጋገፍ በየሳምንቱ በቪዲዮ ኮንፈራንስ ከታችኛውም ተቐማት ጋር እያደረግን እንገኛለን፡፡ በየጊዜው የምናደርገው ግምገማ ከላይ በሶስት አቅጣጫ ያወረድነውን ተግባራት በዞንና ልዩ ወረዳ ደረጃ ባሉት ዐቢይና ንዑሳን ኮሚቴዎች ደረጃ የተገመገመ እኛጋ ሲላክ እኛ ደግሞ የምንገመግምበት መድረክ ነው፡፡በተጨማሪም የመረጃ ልውውጥ እንዲፋጠን የቢሮ ቴሌግራም ፔጂ ከፍተን በዚሁም የክትትልና ድጋፍ ስራውን እያሳለጥንነው፡፡ በጎ ፍቃድ የወጣቶች አገልግሎት ከክልሉ ቀይ መስቀል ጋር በመሆን ሰፊ የበጎ-ፈቃድ አገልግሎት እየተሰጠ ነው፡፡ ይህ የበጎ ፈቃድ አገልግሎት በቀይ መስቀል ማህበር ስብሳቢነትና በኛ ቢሮ ምክትል ቢሮ ሃላፊና የወጣቶች ዘርፍ ሃላፊ በምክትል ሰብሳቢነት ይመራል፡፡ የበጎ ፈቃድ ስራ የቢሮአችን መደበኛ ስራ አካል ነው፤ ሁልጊዜ በጋና ክረምት በጎ ፈቃድ ሰጭ ወጣቶችን በማስተባበር የምናከናወነው ነው፡፡ የወጣቶች በጎ ፈቃድ አገልግሎት ከኮቪድ -19 ጋር ተያይዞ የምንከታተልበት የተለየ የቴሌግራም ፔጅ ከቀይ መስቀል ማህበር ከመምህራን ማህበር ጋር በጋራ የምንሰራው ነው፡፡ እነዚህን ሥራዎች ከባለድርሻዎች ጋር በጋራ የምንሰራው ነው፡- እነዚህም ቀይ መስቀል ሠራተኛና ማህበራዊ ጉዳዮች ቢሮ ጤና ቢሮና ሌሎችም እንደ ወጣቶችና ሴቶች አደረጃጀቶች ጋር በጋራ እንሰራለን፡፡ በተለይ ከሰራተኛና ማሕበራዊ ጉዳዮች ቢሮ ጋር ያለው ቅንጅት እጅግ የተሻለ ነው፡፡ ድጋፍ የሚያሰፈልጋቸውን የህብረተሰብ ክፍሎችን ከመለየት አኳያ በየደረጃው ካሉት መዋቅሮች ጋር በጋር ነው የምንሰራው፡፡

የሴቶች ልማት ቡድን በኮቪድ ምላሽ ጋር በተያያዘ በምን ደረጃ እየተሳተፈ ነው?

ቅድም የሴቶች አደረጃጀት እስከቀበሌ ይወርዳል ስንል የሴቶች ልማት ቡድንን ጨምሮ ነው፤ የመንግሰት መዋቅር እስከቀበሌ የሚወርደው በሴቶች ልማት ቡድን ሲሆን የድርጅት ክንፍ በሴቶች ሊግና ፌዴረሽን በኩል ነው፡፡ እነዚህ የሴቶች ልማት ቡድኖች ፌዴረሽኖችና ሴቶች ሊግ አባላት እንደቀድሞ 30 30 ሆነው ሳይሆን አሁን ኮቪድ -19ን ለመከላከል በሚያስችል መልኩ አመራሮች ራሳቸው ግንባር ቀደም በመሆን ራሳቸውን በመጠበቅና ሊፈጸም የሚገቡ ጥንቃቄዎችን አስቀድሞ ራሳቸው ተግብረው ሌሎችን በማሰተማር ስራ እንደአርአያ በመሆን እያስተማሩ ይገኛሉ፡፡

ሌላው ሴቶችን የሚጎዳው ጉዳይ ኮቪድ -19 በሽታ ባስከተለው የእንቅስቃሴ ገደብ ለአስከፊ የኢኮኖሚ ችግር የሚጋለጡ ሴቶች እንደመሆናቸው እነዚህን የማቋቋም ስራ ግንባር ቀደም ተግባራችን አድርገን እየሰራን ነው፡፡ ከዚህ በተጨማሪ የክቡር ጠ/ሚ/ር ፐሮጄክት የሆነው የማዕድ ማጋራት ሥራ ከመተግበር አንጻር በየአካባቢው ሴቶች በአቅማቸው በልማት ቡድን ደረጃ 50 ብር መቶ ብር እንዲሁም የላቸውን እያዋጡ እቅም የሌላቸው በረሀብ እንዳይሞቱ ድጋፍ እያደረጉ ናቸው፡፡ ብር ብቻ ሳይሆን ካላቸው እህል ጥራጥሬ ስራስር እንዲሁም ባንዳንድ አካባቢ አንድ ብር ለአንድ እናት በሚል መርህ ገንዘብ በማሰባሰብ የሚተጋገዙበት አግባብ ተፈጥሮአል፡፡በዚህ ሂደት በዓይነትና በገንዘብ የተሰበሰበው ወደ ገንዘብ ሲቀየር አስከ 29 ሚሊየን ብር የሚገመት እንደሆነ አይተናል፡፡

ከዚህ ጋር ተያይዞ የሴቶች ለኮቪድ-19 ያለው ተጋላጭነት ከፍ ያለ በመሆኑ ራሳቸውን እንዲጠብቁ በተገኘው አጋጣሚ ሁሉ መረጃ የማድረስና የማስተማር ሥራ እየሰሩ ናቸው፡፡ ሴቶች ባላቸው የቤተስብ ሃላፊነት ምክንያት እቤት የሚቀመጡ ወደ ገበያ ወፍጮ ቤት ወደመሳሰሉት ቦታዎች በተደጋጋሚ የመውጣት ግዴታ ስላለባቸው በሚሄዱበት ቦታ ራሳቸውን እንዲጠብቁ ና የመከላከያ ቁሳቁስ እንዲጠቀሙ ማስክ እንከዋን ባይገኝ ስካርፕ በአፋቸውና አፍንጫቸው እንዲያደርጉ በማድረግ ግንባር ቀደም ሴቶች ትልቅ ሃላፊነት እየተወጡ ናቸው፡፡

ባንዳንድ ሁኔታ እናቶች ሴት ልጆቻቸውን ወደወፍጮ ቤት ይልካሉ፤ በዚህን ጊዜና በየሰፈሩ ሲጫወቱ ለበሽታ እንዳይጋለጡ ከወጣት በጎ ፈቃደኛ አደረጃጀቶች ጋር በመሆን ያስተምራሉ፡፡ ጠቅለል ስናደርግ ሴቶች በርካታ ስራዎችን እየሰሩ ይገኛሉ፡- ማስተማሩ መደጋገፉና ራሳቸውንም ከበሽታ ለመከላከል የሚያደርጉት ጥረት በአዎንታዊ ያየናቸው ናቸው፡፡

ለኮቪድ -19 በሽታ መከላከል ስራ እንደ ቢሮ ሀብት መድባችሁ ከመንቀሳቀስ አንጻር

አጠቃላይ የሀብት ማሰባሰብ ስራን በሚመለከት የሀብት ማሰባሰብ ንዑስ ኮሚቴ ሃላፊነት ወስደው ከመንግስታዊ መንግስታዊ ካልሆኑት ተቋማት እንዲሁም ከህዝቡ ሀብት በማሰባሰብ ለበሽታው መከላከል ይመድባሉ፡፡ በዚህ ሂደት በክልል ደረጃ እስከ 300 ሚሊየን ብር እንደተሰበሰበ ይታወቃል፡፡ በኛ ደረጃ ደግሞ ላቀድነው ስራ የሚሆን ሀብት የመደብንበትና የፌደራል ሴቶች ህጻናትና ወጣቶች ሚኒስቴርም የላኩትን ዕቅድ ስለመፈጸማችን ድጋፋዊ ክትትል ስመጡ ባዶ እጃቸውን ሳይሆን ሁለት ጊዜም ሲመጡ የተለያዩ የቦንዳ ልብሶችና ሌሎች ግብዓቶችን በሁለት መኪና ጭነው ነው የመጡት፡፡ እኛም ይህንን ለታችኛው መዋቅር አሰራጭተናል፡፡

የቢሮአችን ሥራ በባህሪይው ንቅናቄ እንደመሆኑ የኮቪድን ሥራ ከመደበኛ የንቅናቄ ስራችን ጋር አያይዘን እንሰራለን፡፡ የኛ መዋቅር የሚያስገነባው ሌላ ፕሮጄክት የለንም፤ የኛ ፕሮጄክት የሰውን አቅም (የሴቶችን ወጣቶችን አቅም ለተልዕኮ) ማብቃት ነው፡፡ የኮቪድን ሥራ ከመደበኛ የቢሮና የድርጅት ሥራ ጋር እንደት አቀናጅተን መስራት እንዳለብን በክልል አስተባባሪ ደረጃ በተሰጠን አቅጣጫ የሶስት ወር ዕቅድ አዘጋጅተን በማነጅመንት ደረጃ ግምግመንና ከታችኛው መዋቅር ጋር ተግባቦት ፈጥረን እየተገበርን እንገኛለን፡፡

በቢሮአችሁ ሰራተኞችን ከኮቪድ -19 ከመከላከል አንጻር ምን እየተሰራ ነው?

የሰራተኛውን ደህንነት ከመጠበቅ አንጻር ከክልሉ የፐብሊክ ሰርቪስና ሰው ሀብት ልማት ቢሮ ጋር በከፍተኛ የቅንጅት ስሜት እየተገበርን እንገኛለን፡፡ ሥራችንን ከመገምገም አኳያ በሁሉም መዋቅር ከሼካ ዞን ውጭ በአካል ደርሰን ገምግመን ግብረመልስም የሰጠንበት ሁኔታ አለ፡፡ በሄደንባቸው አካባቢዎች ካጋጠሙን ጉድለቶች ውስጥ የንጽህና መጠበቂያ ቁሳቁሶች በከፍተኛ ሁኔታ ታይተዋል፡፡ ወጣቶች በበጎ ፈቃድ አገልግሎት እየተሳተፉ ራሳቸውን ለመከላከል የሚያስችል የግል መከላከያ እያገኙ አለመሆን ነው፡፡ ይህን አይተን ከተመለስን በሓላ ከራሳችን በጀት የፋይናንስ ቢሮ ጋር ተወያይተን 750000 ብር መድበን የንጽህና መጠበቂያ ቁሳቁሶችን ገዝተን አሰራጭተናል፡፡ ይህንን ችግር አይተን ከተመለስን በሓላ ለዐቢይ ኮሚቴ አቅርበናል፡፡ በዐቢይ ኮሚቴ በሳምንት ሁለትና ሶስት ጊዜ እየተገናኘን እንገመግማለን፡፡ በኛ ሴክተር ደረጃ በየቀኑ እየተገናኘን እንገመግማለን ፤ እንደዚሁ ደግሞ በንዑስ ኮሚቴ ደረጃ፡፡ ሌላው ደግሞ ልዩድጋፍ የሚስፈልጋችውን ዜጎችን ለይቶ የመደገፍ ሥራ በልዩ ሁኔታ በክልሉ ብልጽግና ፓርቲ የፖለቲካ ዘርፍ ሃላፊ የሚመራ ሆኖ ይህም በታችኛውም መዋቅር በዚሁ አግባብ ተጠርንፎ በቅንጅት እንዲሰራ ታስቦ የወረደ መሆኑ ነው፡፡ ይህንን ትግበራ ሁላችንም ባለድርሻ አካላት በዙም ጭምር እንገመግማለን፡፡

ባደረግነው ንቅናቄ የንጽህና መጠበቂያ ቁሳቁሶች ከአዲስአበባ ከተማ አስተዳደር ድጋፍ ተሰጥቶናል፡፡ በግል የህክምና አገልግሎት የተሰማሩትና ሌሎችም በጎ አድራጊዎች የለገሱልንን በቀይ መስቀል በኩል እንዲሰራጭ አድርገናል፡፡ የስርጭት አግባብ መስፈርት ወጥቶ በዐቢይ ኮሚቴ ተወስኖ ለሁሉም መዋቅር እንዲደርሰ ተደርገዋል፡፡ ይህንን ከቀይ መስቀል ጋር የሚያሰራጩት የወጣት አደረጃጀቶች ስለሆኑ ሳይዘገይ ከተጠቃሚዎች እንዲደርስ ይደረጋል፡፡

በክልላችን የሀብት አሰባሰብና ስርጭት ሶስት አካሔድ ይከተላል፡- 1) አልባሳትና የምግብ ቁሳቁሶች በአደጋ ስጋት ሥራ አመራር በኩል፤ 2) የንጽህና መጠበቂያ ቁሳቁሶች በቀይ መስቀል በኩል እና መድሃኒትና የህክምና መገልገያ ቁሳቁሶች በጤና ቢሮ በኩል እንዲሰራጩ ይደረጋል፡፡ በሶስቱ በኩል በተቋቋመው ኮሚቴ አማካይኝነት ወጥ በሆነ መልኩ ለሁሉም መዋቅር እንዲደርስ ይደረጋል፡፡ ይህንን ድርጊት ስንፈጽም በጋራ ተቀናጅተን ነው፡፡ በንዑስ ግብረ ሀይል ደረጃ በየቀኑ ገምግመን የለየናቸውን ጉድለቶችን ለዐቢይ ግብረ ሀይል እናመጣለን፡፡ በዐቢይ ኮሚቴ ደረጃ በሳምንት ሶስት ቀን እየተገናኘን እንገመግማለን፡፡ ሰብሳቢው ድንገት ሀገራዊ ስብሰባ እንከዋን ቢሄድ በምክትል ሰብሳቢ በኩል ሳይቆራረጥ እንዲካሔድ ይደረጋል፡፡ ከፌደራል የሚኒስቴር መስሪያ ቤት ጋር በሳምንት አንድ ጊዜ ተገናኝተን ስራችንን እንገመግማለን፡፡ በሚኒስቴር ደረጃ ያሉት ሶስቱም ዘርፎች የየራሳቸውን እዚህ ካሉት የዘርፍ ሀላፊዎች ጋር እኔንም ጨምሮ ለየብቻው ይገመግማሉ፡፡ ምክንያቱም ባንድ ላይ ብቻ ተገምግሞ የሚቀር ከሆነ አንዱ ይጎላል፤ ሌላው ተገቢ ትኩረት ላይሰጠው ይችላል፡፡ ሚኒስቴር መስራያ ቤቱ ከላይ ከተጠቀሰው በተጨማሪ ለህጻናት የሚሆን ግብዓቶችም ሰጥተውናል፡፡ በክልላችን ወደ 23 የሚሆኑ የህጻናት ማቆያ ማዕከላት አሉን፡፡ ለነዚህ ማዕከላት እንህጻናት አስፈላጊ የሆኑትን ግብዓቶች፡- ዳይፔር ወተት እና ሩዝ የመሳሰሉትን አሰራጭተናል፡፡ በዚህ ሂደት ውስጥ የፌደራል ሚኒስቴር መስሪያ ቤትም ደግፎናል፡፡ የስራ ግምገማ ስናደርግ ይህንንም ታሳቢ ተደርጎ ነው፡፡ ሀዋሳ ከተማ ውስጥ ሴፍ ሀውስ የሚባል ማዕከል ውስጥ ጥቃት የደረሰባቸው ሴቶችና ህጻናት የሚቆዩበት ማዕከል አለ፡፡ እዚህ ላሉትም የተለያዩ ድጋፍ ሰጭዎችን አስተባብረን ድጋፍ እያደረግን ነን፡፡ ከዚህ በፊት ይህ ማዕከል ከውጭ በሚገኝ ዕርዳታ ይደገፍ ነበር፡፡ አልባሳትንና የንጽህና መስጫ ቁሳቁሶችን በመስጠት ሁለት ዙር ደግፈናል፡፡

ተቋማችሁ የኮቪድ-19 በሽታ ለመከላከልና ለመቆጣጠር ባደረገው ሂደት ውስጥ እንደ መልካም ተሞክሮ ያገኘው ነገር ምንድነው? ቅንጅታዊ አሰራርን ከማጠናከር አንጻርስ የታየ ነገር ካለ፤

ቅንጅታዊ አሰራርን ከማጠናከር አንጻር የተሻለ አጋጣሚ ፈጥሮአል ማለት ይቻላል፡፡ በርግጥ የኛ ተቋም ካለው ልምድ አንጻር ከበርካታ መዋቅሮች ጋር ስለሚሰራ የተሻለ ልምድ አለው፡፡ በዚህም ፊት የወጡ አደረጃጀቶችም አሉ፡፡ በጣም የሚያስቸግሩም ነበሩ፡፡ አሁን ከኮቪድ ጋር ሆነው ሁሉም ባለቤት ሆነው ባሉበትና በተቀናጁበት ባለቤት ሆነው እንደሚሰሩ ነው የማየው፡፡ እንደዐቢይም ሆነ ንዑሳን ግብረ-ሀይሎች ሁሉም በተጠናከረ ሁኔታ ተቀናጅተው እየሰሩ እንዳለ ይሰማኛል፡፡ በዚህ ሂደት ውስጥ የታዘብኩት ለአንድ ሰው ብቻ ይተው ማለት ሳይሆን ሁሉም እኔ ድርሻ አለኝ ብሎ እየሰራ ነው፡፡ በዚህ ሂደት ውስጥ እንደሴክተር ተለይቶ የተሰጠውን ስራ የሚሰራ አካል ለምሳሌ እንደጤና የበሽታውን ሥርጭት በሚመለከትመረጃ ለህብረተሰብ የማዳረስ ስራ ተሰጥቶታል፡፡ ሌሎች የግብረ-ሀይሉ አባላት ደግሞ በየንዑስ ኮሚቴ የተሰጠንን ሥራ በመስራት ያለመቆራረጥ እንተጋገዛለን፡፡ የቅንጅታዊ አሰራር መገላጫ ከሚሆኑት ውስጥ ኮቪድ -19 በሽታ በራሱ ዘርፈ ብዙ አቅጣጫ የሚዳስስ እንደመሆኑ ምላሹም ዘርፈ ብዙ መሆን ስለሚያስፈልግ በኢኮኖሚው በማህበራዊው በፖለቲካውና በመሳሰሉት ተደጋግፎ እየተሰራ ነው፡፡ ከላይ በዝርዝር ያነሳናቸው ድጋፎችም ዜጎች በመሠረታዊ ፍላጎት ባለመሟላት ለሞትና በሽታ በተጨማሪ እንዳይጋለጡ በማሰብ የተሰራው የዚህ መገለጫ ነው፡፡ ሌላው ደግሞ በየደረጃው የመተሳሰብና የመደጋገፍ ባህላችን ይበልጥ እንዲጎለብት የማድረግ ሁኔታ ፈጥሮአል፡፡ መንግስታዊም ሆነ መንግስታዊ ያልሆኑ ተቋማት የችግሩን አሳሳቢነትን በመገንዘብ አስቀድሞ ያቀዱትን ዕቅድ ሰርዞ ወይንም ከልሶ በጀት ለኮቪድ-19 ማዞር ህብረተሰቡ ካለው ቀንሶ ለሌላው (አርሶአድር ከእህሉና ከከብት ሌላው በማሰብ ይህም ከዚህ በፊት የነበረንን የመረዳዳት ባህላችን ያጠነከረ) የማካፈል እና ሁሉም ጉዳዩ የእኔ ነው ስሜት የተንቀሳቀሰበት ሂደት ለቅንጅታዊ አሰራር ተልቅ ትርጉም እንዲሰጠው አድርገዋል፡፡ ይህ ቅንጅታዊ አሰራር ከላይ በጠንካራ መሰረት የተጀመረ በመሆኑ እስከታችኛው መዋቅር ድረስ በተሻለ ሁኔታ መኖሩን በተለያዩ ጊዜ ለድጋፋዊ ክትትል በወጣንበት ያየነው ነው፡፡ ከቅንጅት አንጻር መነሳት የሚገባው ከሚዲያ ተቋማት ጋር የተደረገው ሥራ ነው፡፡ ከክልል መንግስት ኮሙኒኬሽን ከደቡብ ሬዲዮና ቴሌቭዥን ድርጅት እነዲሁም የግል ሚዲያ ተቋማት ጋር በርካታ ሥራዎችን በጋራ ሰርተናል፡፡ እናንተም እንደህብረተሰብ አካል አይታችሁና ሰምታችሁ ይሆናል፡፡ የህብረተሰቡ ግንዛቤ በሚገባ ስላላደገ ተደጋጋሚ የግንዘቤ ማስጨበጫ ሥራ አካል ጉዳተኛ ስፖርተኞችን በማካተት ከሀዋሳ ጀምሮ እስከ ሼካ ደቡብ ኦሞ ድረስ ቅስቀሳ ሲደረግ ታሳቢ የተደረገው አካል ጉዳተኞች ሲያስተምሩ ህዝቡ በቁጭት ይከታተላል በሚል ነው፡፡ትምህርት ማስተማር ብቻ ሳይሆን ደምንም በመለገስ ተሳታፊ ሆነዋል፡፡ የሚዲያ ተቋማት በራሳቸው ደረጃ የሚሰሩት እንደተጠበቀ ሆነው ከኛ ጋርም በጋራ በርካታ ሥራዎችን የፈጠራ አቅም ያላቸውን ወጣት ሪፖርተሮችን በመጠቀም የሚያነቃ ማስታወቂያዎችን የሰሩት ሊጠቀስ ይገባል፡፡ በክልል ባህል ቱሪዝምና ስፖርት ቢሮ የስፖርተ ኮሚሽን ስር ያሉት እነዚህ አካልጉዳተኛ ስፖርተኞች የሰሩት ስራም ከቅንጅት አንጻር በርካታ ስራዎችን መስራታችን የሚያረጋግጡ ገጽታዎች ናቸው፡፡

በሂደት እንደክፍተት ታይቶ መሻሻል አለበት የተባለ ካለም ቢገልጹ፣

እንደጉድለት የማነሳው ባሰማራነው ልክ ለወጣቶች የተለያዩ የንጽህና ማስጠበቂያ ቁሳቁሶችን ማሟላት አለመቻላችን ነው፡፡ ወጣቶች የኮቪድን ተልዕኮ ጨምሮ በርካታ መደበኛ የክረምት በጎ ፈቃድ አገልግሎት (አቅም ደካሞችን ቤት መስራትና ማደስ በግብርና በጤና በተለያዩ ተሮገራሞች ተሳታፊ ስለሚሆኑ) ላይ ይሳተፋሉ፡፡ አንዳንድ አካባቢ የሚያስፈልጉትን አሟልቶ የሚያቀርቡ ቢኖሩም ባብዘኛው ግን ክፍተት ይታያል፡፡ ይህ በቀጣይ የሚስተካከልበት ሁኔታ ቢኖር መልካም ነው፡፡ በብዙ ባለድርሻ ተሳትፎ የግንዛቤ ማስጨበጫ ስራ ቢሰራም በህዝቡ ዘንድ ይህንን ተቀብሎ ከመተግበር አንጻር ከፍተኛ ጉድለት ይታያል፡፡ አካላዊ ሪቀትን በመጠበቅ ለበሽታው ተጋላጭነት ህዝቡ ራሱን እንዲጠብቅ የተቀመጠው አቅጣጫ ሲዛነፍ እናያለን፤ ለምሳሌ የሐይማኖት ተቋማት ማህበረ-ምዕመናኑ የጋራ አምልኮ ለማድረግ እንዳይገቡ ተብሎ እያለ አሁንም ተሰብስበው የሚያመልኩት አሉ፡፡ አንዳንድ አካባቢ ቤተክርስቲያን መሄድ ሲገደብ በግለሰብ ቤት በርከት ብለው ተሰብስበው የማምለክ ሁኔታ ይታያል፡፡ በዕጃችን ባሉትና ቀላል በሆኑት ሁኔታዎች መከላከል ስንችል ይህንን ወደጎን ትተን ከባድ ወደሆነው እየተጓዝን እንገኛለን፡፡ አንዳንድ አካባቢ ትኩረት ማነስም አለ፡ በዚያው ልክ የቅንጅታዊ አሰራርምክፍተት ያለበት፡፡

ጥሩ ጅምር ነበር ግን በአሁኑ ጊዜ መቀዛቀዝ ታይተዋል የሚሉ አለና ይህንን እንዴት ያያሉ?

በኔ ግምገማ ከባለፈው ሚያዝያ ጀምሮ ማስተማሩ በተወሰነ ደረጃ መቀዛቀዝ ታይተዋል፡፡ ምን ሊባል እንደሚችል ባላውቅም ሰው ተላምደዋል ሊበል እኔ እንጃ ባንዳንድ አካባቢ ማዳመጥንም የተውበት አለ፡፡ ይመስለኛል አሁን በሚታየው ክልላዊና ሀገራዊ አጀንዳ የመግባት ሁኔታ አለ፤ሊታወቅ የሚገባው ሌላው ሌላ ነው፤ህይወት ህይወት ነው፡፡ ማንኛውንም አጀንዳ ልናሳካ የምንችለው ህዝብ ስኖር ስለሆነ በዚህ በቀላል መከላከል በምንችለው በሽታና በረሀብ የሰው ልጅ እንዳይሞት ተግተን መስራት ያስፈልገናል፡፡ ይህ መቀዛቀዝ በክልል ደረጃም ተገንዝበን በአስተባባሪ ደረጃ ተገምግሞ እስከታችኛው መዋቅር ድረስ ባሉበት የሃሳብ ልውውጥ ተደርጎ እንደገና ማጠናከር አሰፈላጊ መሆኑ ተረጋግጦ ወደስራ እንዲገባ አቅጣጫ ተቀምጠዋል፡፡ ከኮቪድ-19 ወረርሽኝ ከክልል አልፎ እንደ ሀገርና አለም አቀፍ እንደሴቶች ህጻናትና ወጣቶች ትልቅ ማነቆ የሆነው እቤት ውስጥ የሚደረጉ ጥቃቶች ናቸው፡፡ ይህንን ክስተት ለመከላከልና ለማስቆም ከፍትህ አካላት ጋር በቅንጅት እየሰራን ነው፡፡ ከኮቪድ ጋር ተያይዞ ፍርድ ቤቶች በከፍል ዝግ በመሆናቸው ወንጀል የሰሩት ሁሉ ወደ አለመጠየቅ ደረጃ መድረሳቸው የዚህ ዓይነት ጥቃቶች እንዲበራከቱ እያደረገ በመሆኑ ከአቃቤ ህግና ፍርድ ቤቶች ጋር በመቀናጀት እንደዚህ ዓይነት ወንጀል የሰሩት እንዲጠየቁ የማድረግ ስራ እየሰራን ነው፡፡

በዚህም ከዚህ በፊት ከፍትህ አካላት ጋር የተፈራረምነውን መግባቢያ ሰነድ እንደገና መከለስ ስላስፈለገ በመዋቅራችን አዲስ የተካተተውን የወጣቶችን ዘርፍ በማካተት የመግባቢያ ሰነድ እየከለስን ነው፡፡ በዚህም ፍርድ ቤቶች የሴቶችንና ህጻናትን ጉዳይ እንደቀለብ ጉዳይ ያለምንም ቆይታና ረጅም ቀጠሮ ማየት እንደሚገባ ተወያይተናል፤ በቅርቡ ተግባራዊ የማይደረግ ከሆነ ያለምንም ማመንታት እንደገና ማንሳት እንዳለብን ቆርጠናል፡፡ ይህንን ስራ በቅንጅት ለመስራት ከፍርድ ቤቶች አቃቢያነ ህግ እና ከፖሊስ አካላት ጋር የጋራ ዕቅድ አዘጋጅተን ለታችኛው መዋቅር ካወረድን በኋላ ስራው ምን እንደሚመስል ለማየት በቅንጅት ወደ ታችኛው መዋቅር በጋራ ወርደን ድጋፍና ክትትል ያደረግንበት ሁኔታ አለ፡፡ በዚህ ድጋፋዊ ክትትል ወቅት ከሴቶችና ህጻናት ጥቃት አኳያ በርካታ የአፈጻጸም ጉዳዮችን የሚዳስስ ቼክሊስት አዘጋጅተን ነው የወረድነው፤ እያንዳንዱ መዋቅር ምን እንደሰራ ያየንበት ነበር፡፡በዚህ ወቅት የሚደረጉ የሴቶችና ሕጻናት ጥቃቶች ዓይነታቸው መጠናቸውንና ባህሪያቸውን በሚመለከት በዓለም አቀፍ ደረጃ የተባበሩት መንግስታት የሴቶች ዘርፍ ባስጠናው ችግሩ የገዘፈ እንደሆነ የጠቆመ ሲሆን የሀገራችንም ሚኒስቴር መስሪያ ቤቱ እያስጠና ነው፤እኛም እንደክልል ልናጠና ዕቅድ አለን፡፡ኮቪድ በፈጠረው የቤት መቀመጥ ሁኔታ በርካታ ማህበራዊና ኢኮኖሚያዊ ገጽታዎች ለውጥ በቤተሰብ ደረጃም ያለመግባባት በመፍጠር በቀላል ወደ ግጭት እንዲገቡ እያደረገ ነው፡፡ በ15 መዋቅሮች ወደ 108 የሚሆኑ ድብደባዎች ሪፖርት ተደርገዋል፡፡ እነዚህ ድብደባዎች ከፍ ያሉ ናቸው፤ መለስተኛ ድብደባዎች ለፖሊስ ሪፖርት አይደረጉም፡፡ ወደ 68 የሚሆኑ ጠለፋዎች ተፈጽመዋል፡፡ የሚገርመው ጉዳይ ከዩኒቨርሲቲ የመጡት ሴት ተማሪዎች በአንድ ልዩ ወረዳ አግብተው አግኝተናል፡ አንዷ አርሶ አደርን አግብታለች፤ መቼም ይህ ሙሉ በሙሉ በፍላጎት የተደረገ ጋብቻ ነው ማለት አይቻልም፡፡ ጌዴኦ ላይ አባት ልጅን የደፈረበት ፤ አርባምንጭ የሰባ ዓመት አዛውንት ዲዳ የሆነችውን ልጇ ደፍረዋል፡፡ በርካታ ግድያዎች እየተፈጸሙ ነው፡፡ ቤንች ሼኮ ላይና በጉራጌና ስልጤ ላይ በእያንዳንዳቸው አራት ግድያዎች በባል ሚስትን የገደለበት ሁኔታ ተከስተዋል፡፡ ከዚህ በፊት በክልላችን የማይሰማውና በሰውም በፈጣሪም አጸያፊና ነውር የሆነው ግብረሰዶም የመለማማድ ሁኔታ በክልላችንም እየተፈጸመ መሆኑ፤ እነዚህ እንግዲህ ታች በወረድንበት ጊዜ ከአመራሩ ያገኘናቸው መረጃዎች ናቸው፡፡ በዚህ መነሻ ሰነድ አዘጋጅተን በየደረጃው ከሚገኙት የባለድርሻ አካላት ጋር በቀጣይ መሆንና ማድረግ ስለሚገባን ውይይት አድርገናል፡፡

ቁልፍ መረጃ ሰጪ ቁጥር ስድስት ትራንስፖርትና መንገድ ልማት ቢሮ

የናንተ ተቋም የኮቪድ -19 በሽታ መከላከልና ቁጥጥር ሥራ ጋር ያለው ቁርኝት ምን ይመስላል?

ከታችኛውና ላይኛው መዋቅር ጋር የተፈጠር የስራ ግኑኝነት፤ ከማስተባበርና በአጋሪነት ከመስራት አንጻር፤ ተቋሙን ለተልዕኮ ብቁ ከማድረግ አንጻር ምን ይመስላል?

የኮቪድ -19 በሽታ መከሰት በሀገራችንና በክልላችን ከተረጋገጠ ጊዜ ጀምሮ በኛ ተቋም በሁለት መንገድ አቅደን እየተንቀሳቀስን እንገኛለን፡፡ ሴክተራችን እንደሚታወቀው በ ሚሊየን የሚቆጠሩ የህብረተሰብ ክፍሎች አገልግሎት የሚያገኙበት ተቋም ነው፡፡ በመሆኑም በውጭ የትራንስፖርት ሥራ የሚደግፉትና በመስሪያ ቤት ደረጃ ደግሞ የውስጥ ና የውጭ ደንበኞችን የሚያስተናግዱ ሰራተኞች ከክልል እስከ ወረዳ ድረስ አሉ፡፡ ከዚህ በሽታ ጋር በተያያዘ ምን ማድረግ አለብን ስንል ሁለት ዓይነት ዕቅድ አዘጋጅተናል፡፡ አንደኛው ዕቅድ በተጠሪ ተቋማትና በኛ መካከል ሊኖር የሚገባ ሥራ ምንድነው በሚል አቅደን እየሰራን ነን፤ ሌላው ደግሞ በዚሁ በሴክተር ቢሮአችን ያሉት ሰራተኞች ራሳቸውንና ደንበኞቻቸውን እንዴት መከላከል ይችላሉ የሚል እቅድ አዘጋጅተን ተጠሪ ለሆኑት ታችኛው መዋቅር ተቋማትም ምን ማድረግ እንዳለባቸው በቪዲዮ ኮንፈራንስም ጭምር ተወያይተን ዕቅዱ እንዲደርሳቸው በማድረግ እየሰራን ነው፡፡ በቢሮአችን ደረጃ የሰራተኞች ተጋላጭነትን ከመቀነስ አኳያ በዕድሜ የገፉትን ተጓዳኝ በሽታ ያለባቸውን እንዲያርፉ በማድረግና በቦታ ጥበት ምክንያት ተፋፍጎ ከሚሰሩት ውስጥ በፈረቃ እንዲሰሩ አድርገናል፡፡ ሌላው ደግሞ የተቋማችን አገልግሎት ፈልጎ የሚመጡት ደንበኞች ወደ ህንጻ ከመግባታቸው አስቀድሞ እጃቸውን እንዲታጠቡ ከእጅ ንክኪ ውጭ በሰንሰር የሚሰሩ የእጅ መታጠቢያ እቃ በማዘጋጀት እጃቸውን ታጥበውና ማስክ አድርገው እንዲገቡ እያደረግን ነው፡፡ በተጨማሪም ወደሃላፊ ቢሮ ሲገቡ ደግሞ እጃቸውን በሳንታይዘር እንዲያጸዱ ይደረጋል፡፡ እያንዳንዱ ዳይሬክቶረት በስራቸው ያሉትን ሰራተኞችን ደህንነትን ከማስጠበቅ አኳያ እንደሳኒታይዘር ሳሙና የመሳሰሉትን በመግዛት እንዲያቀርቡ ይደረጋል፡፡ ከሀዋሳ እንዱስትሪ ፓርክ የአፍ ና አፍንጫ መሽፈኛ ጭንብል በመግዛት ከፌደራል የትራንስፖርት ሚኒስቴርና የመንገድ ልማት ባለስልጣን የተበረከቱትን ሳኒታይዘሮችን የተወሱንትን እዚህ ካስቀረን በኋላ ሌላውን ለዞኖችና ልዩ ወረዳዎች አሰራጭተናል፡፡ በቢሮም በጀት መድበን ግዥ በማከናወን እየተጠቀምን እንገኛለን፡፡ ለተወሰነ ጊዜ የሚሆን መጠባበቂንም በእጃችን ይዘን እንገኛለን፡፡ በንደዚህ ሁኔታና ዝግጅት የሰራተኞቻችን ደህንነት እየጠበቅን ከመሆንም ባሻገር አሁን ጊዜው ክረምት ከመሆን አንጻር የመስክ ሰራተኞቻችን በቻይናና ኮሪያ ላይ በአገልግሎት የዋለውን ዓይነት አልባሳት እንዲጠቀሙ ግዥ ፈጽመን እያደልን ነው፡፡ ይህ ልብስ ከንክኪ ነጻ ያደርጋል፡፡ ጎርፍና ዝናብ እንዳይነካ ያደርጋል፡፡ በሞተር ሁለት ሰዎች ድንገት ቢሳፈሩ አንደኛው ከሌላይኛው ሰውነት ጋር እንዳይነካካ ያደርጋል፡፡ እንግዲህ እነዚህ ጥረቶች ሙሉ ባይሆኑም የሰራተኞቻችን ደህንነት ለመጠበቅ እየተወሰዱ ያሉ እርምጃዎች ናቸው፡፡

በተቋም ውስጥ የኮቪድ-19 መከላከልና ቁጥጥር ስራ የሚያስተባብር የተለየ የተመደበ ስሰው ስለመኖር

አዎን አለ፡፡ የውስጥ ሰራተኞችን ደህንነት በሚመለከት የሚከታተል ሰራተኛ አለ፡፡ የክልሉ ፐብሊክ ሰርቪስና የሰው ሀብት ልማት ቢሮና የሰራተኛና ማህበራዊ ጉዳይ ቢሮ ጋር ሆነው የሰራተኛ ደህንነነት ስለመጠበቁ ይከታተላሉ፡፡ በዋናነት የቢሮአችን የአስተዳደርና ፋይናንስ ዘርፍ ሀላፊ አስፈላጊውን ግብዓት በማሟላት የሚከታተል ነው፡፡ ከቢሮ ውጭ ከትራንስፖርት ጋር ተያይዞ መስክ ላይ ያለውን ሥራ በሚመለከት በዐቢይ ግብረ ሀይል የትራነስፖርት ንዑስ ግብረ ሀይል ሆነው የተደራጀ እኔ የሚመራው አለ፡፡ ይህ ቡድን በየሁለት ቀን እየተገናኘ ሥራውን ይገመግማል፡፡ በየቀኑ ደግሞ መዋቅሮችን በአምስት ኮማንድ ከፋፍለን መረጃውን በየቀኑ ከመዋቅሮች እንሰበስባለን፡፡ በየቀኑ የተሰበሰበው ተጠናቅረው በሁለተኛው ቀን ለኔ ይቀርባል፡፡ በሶስት ቀን አንዴ ለሚደረገው የዐቢይ ኮሚቴ መድረክ ደግሞ እኔ አቀርባለው፡፡በመጀመሪያ አካባቢ በጣም የተጋጋለና ጥሩ ሥራ ስንሰራ ነበር፤የትራንስፖርት ዘርፍ በመጀመሪያ በክልሉ ባሉት 180 መናሔሪዎች ሚኒ ሚዲያዎች መረጃ ለማስተላለፍ እንዲዘጋጁ የእጅ ማስታጠብ ስራ እንዲጀመር ተደርገዋል፡፡ ይህንንም እንደመንግስት ብቻችን መወጣት ስለማንችል ማህበራንና የግል ባለሀብቶችን በማስተባበር የንጽህና መስጫ ቁሰቁሶችን የማሟላት ስራ አከናውነናል፡፡ ተሳፋሪዎች ወደተሽከርካሪ ከመግባታቸው አስቀድሞ ባሌቤቶች ተሸከርካሪውን ውስጥ በኬሚካል እንዲረጩ ይደረጋል፡፡ በንደዚህ ሁኔታ እየሰራን ሂደቱ እጅግ አስፈሪ እየሆነ ሲመጣ ለ15 ቀናት መናሔሪያዎችን ዘግተን የትራንስፖርት እንቅስቃሴ እንዲቆም አድረገን ቁጥጥር ሲናደርግ ነበር፡፡ የፌደራል መንግስት የአስቸኳይ ጊዜ አዋጅ ካወጀ በኋላ ወደእኛ ነባራዊ ሁኔታ በመቀየር ከዚህ በፊት ከሚጭኑት በ50 በመቶ ቀንሰው እንዲጭኑ ፈቅደንና ተሳፋሪው ደግሞ ክፍያውን በእጥፍ እንዲጨምር በማድረግ አፈጻጸሙን እየተከታተልን እንገኛለን፡፡ አስቸኳይ ጊዜ አዋጅ ከመውጣት በፊት እኛ ዘግተን ነበር ግን አዋጁ ከወጣ በኋላ ከፈትን፡፡ምክንያቱም በሽታው ምን ያህል ጊዜ ሊቆይ እንደሚችል ስለማይታወቅ በሌላ በኩል እንቅስቃሴ መዝጋት የኢኮኖሚና ማህበራዊ ቀውስ ሊያስከትል ስለሚችል ይህንን ታሳቢ በማድረግ ነው አዋጁም የወጣውና እኛም የተከተልነው፡፡በርግጥ ውሳኔው እንቅስቃሴን ስትራቴጂካሊ ሊያቆም ወይንም ሊገድብ በሚችል መልኩ ነው የተወሰነው፡፡ እንደምናየው አሁን ያለው እንቅስቃሴ እንደበፊቱ አይደለም፤ ገንዘብ ጨምረዋል፤ የመጫን አቅም ቀንሰዋል፤ ሌሎች አስገዳጅ ሁኔታዎችም ተካትተው እንደቀደመው አይደለም፡፡ በመሆኑም አስገዳጅ ሁኔታ ካልሆነ በቀር አርሶአደሩ እዚህም እዚያም ገበያ እየፈለገ የሚጓዝበትን ሁኔታ ገድበዋል፡፡ ስለዚህ በዚያ መሠረት እየተገበረ ይገኛል፤ ይህ ብቻ ሳይሆን ተሳፋሪው በእጥፍ ስለሚከፍል ሌላ ሰው በትርፍነት እንዲጫን አይፈቅድም፡፡ ይህም በዋና ዋና መንገዶች መለማመድ መጀመሩ ለቁጥጥር ስራችንም አስተዋጽኦ እያበረክተ ነው ማለት ይቻላል፡፡ የቁጥጥር ሥራ በመጠናከሩ በዚህ ሶስት ወር ጊዜ ብቻ እስከ ዘጠኝ ሺህ አሽከርካሪዎችን ቀጥተናል፡፡ በዚህም ወደ 10 ሚሊየን ብር ለመንግስት ገቢ ሆነዋል፡፡በራሳችን ህግ አውጥተን ወጥ የሆነ ነገር ባይኖርም ባንድ ሰው ትርፍ በመጫን 1000 ብር እንዲቀጡ ሰርኩላር በማስተላለፍ ተግባረዊ እያደረግን ነው፡፡ ስለዚህ አንድ ተሽካርካሪ እስከ 20000 ብር ሁሉ ሊቀጣ ይችላል ማለት ነው፡፡ በአሽከርካሪና ተሳፋሪ በኩልም ጥንቃቅ እንዲደረግ እያሳሰብን ነው፡፡ በቅርቡ ደግሞ ይህ የሰራነው ስራ ውጤት አስገኝተዋለዋል? የሚላከው ሪፖርት ትክክል ስለመሆኑ ባለሙያዎችን ልኬን ለማረጋገጥ የሄድንባቸው ሪቀቶች አሉ፡፡ ይህ ብቻ አይደለም መናሔሪያዎቻችን አካባቢ የሙቀት ሊኬት እንዲካሄድ እያደረግን ነው፡፡ ካሉን መናሔሪያዎች 66 የሚሆኑ ሙቀት ይለካሉ፡፡ ይህ ሁሉ እኛ በላክነው ሣይሆን እዚያ ያሉት ከባለሀብቶች ና ማህበራት ጋር በመቀናጀት ገዝተው ያደራጁት ነው፡፡ ሰለዚህ እነዚህን ስራዎች በጋራ እየሰራን እንገኛለን፡፡

ቅንጅታዊ አሰራር ሂደት ምን ይመስላል?

ቅንጅታዊ አሰራርን በሁለት መልኩ እናያለን፡- በድክመትም በጥንካሬም፡፡ በጠንካራ ሁኔታ ከኛ ጋር ተቀናጅተው እየሰሩ ያሉት የጸጥታው መዋቅር ነው፡፡ በተለይ የወጣው የአስቸኳይ ጊዜ አዋጅ ያስቀመጣቸው ክልከላዎች በስራ ላይ ስለመዋላቸው ከሚቆጣጠሩ የትራፊክ ፊሰት ፖሊሶች ጋር በመልካም ቅንጅት እየሰራን ነው፡፡ በዚህ ረገድ የተናበበ ሥራ እየሰራን ነን፡፡ ከዚህ በፊት የትራፊክ ፖሊስ በመንገድና ትራንስፖርት ቢሮ ስር ነበር አሁን ግን በፖሊስ ኮሚሽን ስር ነው፡፡ ስለዚህ ቅንጅታዊ ስራ አስፈላጊ ነው፡፡ እስካሁንም ያለው ቅንጅታዊ ስራ የተሻለ ነው፡፡ በተለይ ዋና ዋና መንገዶች ላይ ቅንጅታዊ አሰራር የተሻለ ነው፤ ነገር ግን ገባ ባሉት መንገዶች አሁንም ትርፍ መጫን ሙሉ በሙሉ ቀርተዋል ማለት አይቻልም፡፡ ምናልባት እዚያ አካባቢ የጸጥታው አካላት ከባለንብረቶችና አሽከርካሪዎች ጋር የመሞዳመድ ሁኔታ ሊኖር ይችላል፡፡ በተለየ በዚህ በኮቪድ ወቅት ጥሩ መደጋገፍና መናበብ አለ ማለት ይቻላል፡፡ ከቀይ መስቀል ጋር ለሰራተኞቻችን በሚያቀርቡት የንጽህና መጠበቂያ ቁሳቁሶች አቅርቦት በኩል የተሻለ ነው፡፡ ሌላው መንግስታዊ ካልሆኑት ማህበራት ጋር ከመንግስታዊ ከሆኑት ጋር የተሻለ ነው፡፡ አሁን በመናሔሪያ ላይ ያሉት ማህበራት ቁሳቁሶችን በመግዛት በማቅረብ እጅ በማሳጠብና ሰዎች ንጽህናቸውን እንዲጠብቁ በማስተማር ከፍተኛ ስራ እየሰሩ ናቸው፡፡ በተደጋጋሚ የሚናነሳው ከጤና ጋር ያለው መስተጋብር ሊጠናከር ይገባል እንላለን፡፡ አሁን ቤት ለቤት አሰሳ ይደረጋል ነገር ግን 15 ቀን ያህል ዞረን የምናገኘውን ያህል ቁጥር በአንድ ቀን በመናሔሪያ ውስጥ ልናገኝ እንችላለን፡፡ በርካታ ሰው በየቀኑ መናሔሪያ ገብተው ይወጣል፤ እኛ በአቅማችን የሚገቡትንና የሚወጡትን እንመዘግባለን፡፡ መሳሪያዎች ባሉበት አካባቢ ሙቀት እንለካለን፤ ማቆያም እንዲዘጋጅ ጠይቀናል፡፡ ሰው ሲሳፈር በተዘጋጀው ፎርም የት እንደሚሄድ እንመዘግባለን፡፡ ሙቀት ተለክቶ ከመጠኑ ከፍ ያለ እንደሆነ እንዲቆይ እናደርጋለን፡፡ ከአዲስአበባ ጠፍቶ መጥተዋል ባንሳ ተገኝተዋል የተባሉት በመናሔሪያ በኩል ነው፡፡ እኛ ተሳፋሪ እንመዘግባለን ግን ከጤናው ጋር በዚህ አግባብ ያለን ቅንጅት የላላ ነው፡፡ ይህ ስራ እንደተጀመረና መጠናከር እንዳለበት ቢያዝ ጥሩ ነው እላለዉ፡፡ ጤና ሴክተሩ ከኛ በየቀኑ የተሳፋሪውን ዝርዝር ቢቀበልና በመከላከልና ቁጥጥር ስራ አካል ቢያደርገው ጥሩ ነው የሚል ግምት አለኝ፡፡ ለወደፊትም በሽታው ከኛ ጋር ምን ያህል ጊዜ እንደሚኖር ስለማይታወቅ ይህ አሰራር መጠናከር አለበት፡፡ በዞን ደረጃ ወዳሉት መናሄሪያዎች ስንወርድ የሙቀት መለኪያ መሳሪያዎች ባሉበት አካባቢዎች ላይ 66 በሚሆኑት ሙቀት እንለካለን፤አሁን አሁን እየገዙ ሁሉም እያደረጉ ናቸው፡፡ የተሳፋሪ ምዝገባ መጀመሪያ አካባቢ ሞቅ ብሎ ግን ወደኋላ የቀዘቀዘበት ሁኔታ አለ፡፡ ከጤና የሚመጡ ባለሙያዎች በተወሰነ የመንግስት ሥራ ሰዓት ዓይነት ተከትሎ ስለሚሰሩ እነርሱ ለምሳ በሚወጡበት ጠዋት ከመግባታቸው በፊትና ማታ ከወጡ በሃላ ወደመናሔሪያ የሚገቡት አይለኩም፡፡ ይህንን ለወደፊት እንዴት ወጥ አድርገን እንሰራለን በሚለው ላይ ክፍተት አለ፡፡ ሌላው የመንግስት መስሪያ ቤቶች በመናሔሪያ አካባቢ ለሚሰሩት ሰዎች ለይቶ ድጋፍ ሊያደርጉ ይገባል፡፡ ዕቃዎች ይገዛሉ፤ ገንዘብ ይሰበሰባል፤ ግን ገንዘብ እየተንቀሳቀሰ አይደለም፡፡ ዝም ብሎ ቁጭ ብለዋል እንጂ ለማን ምን ያስፈልገዋል ተብሎ በጣም በሽታ ሊያተላልፉ የሚችሉ በመናሔሪያ አካባቢ የሚሰሩተ በተለምዶ ወያላ ለሚባሉት የአኗኗር ዜይቤአቸው የጋርዮሽ ለሆኑት እንዴት ራሳቸውንና ሌሎችን መከላከል እንደሚችሉ አስገላጊ ግብአት ማቅረብ ይጠበቃል፤ ማህበራት ላጭር ጊዜ ነው ማቅረብ የሚችሉት፤ ስለዚህ መንግስት ስትራቴጂካል በሆነ ሁኔታ አይተው ማቅረብ ያስፈልጋል፡፡ አሁንም መናሔሪያ መታየት ያስፈልጋል የሚል ጠንካራ አቋም አለኝ፡፡

በዋናው ግብረ ሀይል ደረጃ ያለው ቅንጅታዊ ስራ ምን ይመስላል?

በዋናው ግብረ ሀይል ደረጃ በሳምንት ሁለት ጊዜ እየተገናኘን ሪፖርቶችን እያቀረብን ነው፡፡ በመጀመሪያው ሰሽን መጀመሪያ አካባቢ ግብረ ሀይልን ሞቅ የሚያደርግ የትራንስፖርት ዘርፍ ሪፖርት ነበር፡፡ አዋጁ ከወጣ በሓላ ከኛ የሚጠበቅ የነበረው ምን ያክል ተቀጣ እንጂ ምን ዓይነት ሰው ምን ተደረገ የሚል የተሻለ አስተሳሰብ የታከለበት የለም፡፡ በዚህ ዙሪያ ላይ ብዙ እየተወያየን ስላይደለ መጠናከር አለበት፡፡ ተጨማሪ የአሰራር ስርዓቶችን መፍጠር ያስፈልጋል፡፡ የመናሔሪያ አገልግሎት ጋር በተያያዘ አምስት ነገሮችን ለይተን ተግባራዊ እናደርግ ብለናል፡- የመገምገሚያ መስፈርት መሆን የሚገባቸውም እነዚህ ናቸው፡፡ የዘጋነውን መናሔሪያ የምንከፍተው 1ኛ መናሔሪያው በትክክል መጽዳቱን ማረጋገጥ፣ ያነ አንድ ጊዜ አጽድተን ገብተናል፤ ይህ የአንድ ጊዜ ስራ ነው ወይ ሁል ጊዜ መጽዳት የለበትም ወይ? 2ኛ ሙቀት የሚለካ ሰው በቦታው መገኘት አለበት ከሙቀት መለካት ጎን ለጎን የማቆያ ቦታ ያስፈልጋል የሚል ነገር አለ፤ 3ኛ የንጽህና መስጫ ቁሳቁሶች በየቀኑ ተሟልተው በተግባር ላይ ሊዉሉ ይገባል፡፡4ኛ ሚኒ ሚዲያዎች ሳያቋርጡ ስራዎቹን መስራት አለባቸው፡፡ ይህ ነገር አሁን ቀጣይነት ባለው መልኩ እየተገበረ ነው ብለን ስናስብ ጉድለት ያለ ይመስላል፡፡

ኮቪድን ለመከላከልና ለመቆጣጠር ባደረግነው ርብርብ ያገኘነው መልካም ተሞክሮዎች ካሉ

በጎ ነገር አትርፈናል ብለን የምንወስደው ምናልባት አንድ ክስተት ሲመጣ ይዘው የሚመጣው መልካም አጋጣሚ ሊኖር ይችላል፡፡ አንዱ ተሞክሮ ሃላፊዎች ለሰራተኞቻቸው እንዲያስቡና እንዲጠነቀቁ አደርገዋል ማለት ይቻላል፤ በተለይ በዝቅተኛ ደመወዝ የሚሰሩ እንደ ጥበቃ የጽዳት ሰራተኛ እና መዝገብ ቤት አካባቢ ያሉት በሽታው ካመጣው የገበያ ንረት ጋር ተያይዞ ኑሮን መቋቋም አልቻሉም፡፡ ስለዚህ ወደፊት እንዲውም እንደዚህ ዓይነት ሰራተኞች በዘላቂነት የሚደጎሙበት ማዕቀፍ መንግስት ሊያስብ ይገባል፡፡ የነዚህ ሰራተኞች ጉዳይ ምን ማድረግ እንዳለብን እንድናስብ አድርጎናል፡፡ በማህበር አደራጅተን የምግብ ሸቀጦችን በዝቅተኛ ዋጋ የሚያገኙበትን ሁኔታ ማመቻቸት እንደሚያስፈልግ ያሳሰበ ነው፡፡ ከሀምሌ አንድ ጀምሮ ለማደራጀት እያሰብን ነው፤ በአበል መልክ መደጎም አዋጪ አለመሆን ብቻ ሳይሆን ዘላቂነትም አይኖርም፡፡ ሁለተኛው ከግኑኝነት አንጻር በአመራር ደረጃ ወደላይም ወደታችም ቴክኖሎጂ ተጠቅመን እንድናሳልጥ አድርጎናል፡፡ ከፌደራል ሴክተር መስሪያ ቤትም ሆነ ከዞኖች ጋር የምንገናኘው በዙም ነው፡፡ ይህ ልምድ አሁን በበሽታ ጊዜ ብቻ ሳይሆን በመደበኛ ስራችንም እንድንጠቀም ያሳሰበን አጋጣሚ ነው፡፡ ለምሳሌ ለሁለትና ሶስት ሰዓት ስብሰባ ብዙ ገንዘብና ጊዜ መስዋዕት አድርጎ ብዙ ኪሎሜትሮችን ተጉዞ መምጣት በመተው በቴክኖሎጂ ታግዘን ማከናወን እንድንችል ዕድል ፈጥሮአል፡፡ በግል ደረጃም ካየን ባሁኑ ጊዜ ሰው በተከታታይ እጁን ስለሚታጠብ እንደጉንፋን የመሳሰሉት በሽታዎች እየቀነሱ ናቸው፡ ለካስ የሰው ጤና በእጁ ነው የተባለው እውነት ነው ብዬ እንዲቀበል አድርጎኛል፡፡ እጅ በመታጠብ ብቻ ለህክምና የሚወጣ ብዙ ወጪ መቀነስ እንደሚቻል ያሳየ ነው፡፡ ሌላው በተቋማትና ወጣቶች ላይ ፈጠራ እንድጎለብት አድርገዋል፡፡ የቴክኒክና ሙያ ኮሌጆች ከእጅ ንክኪ ነጻ የሆኑትን የእጅ መታጠቢ ማሽኖችን መስራት መቻላቸው፡፡ እቤታቸው ቁጭ ብሎ የተለያዩ አልባሳትን እንደ ማስክ ስካርፖችና ሌሎችንም ይሰራሉ፡፡ አንዳንዴ ችግር ብልሃትን ይወልዳል የሚለው አባባል እውነታነትን ያየንበት ነው፡፡

ቁልፍ መረጃ ሰጪ ቁጥር ሰባት ውሀ ማዕድንና ኢኔርጂ ልማት ቢሮ

የናንተ ተቋም የኮቪድ -19 በሽታ መከላከልና ቁጥጥር ሥራ ጋር ያለው ቁርኝት ምን ይመስላል?

ከታችኛውና ላይኛው መዋቅር ጋር የተፈጠር የስራ ግኑኝነት፤ ከማስተባበርና በአጋሪነት ከመስራት አንጻር፤ ተቋሙን ለተልዕኮ ብቁ ከማድረግ አንጻር ምን ይመስላል?

የኮቪድ-19 በሽታ በክልላችን ከመከሰት አስቀድሞ ሀገራዊና ዓለምአቀፋዊ ሁኔታ በመረዳት የክልላችን መንግስት ዝግጅት ሲያደርግ ነበር፡፡የክልላችን ዝግጅት አንዱ አካል የሆነው የበሽታውን መከላከልና ቁጥጥር ሥራ በግብረ-ሀይል እንዲመራ ታስቦ የተቋቋመ ዐቢይ ግብረ ሀይል ነው፡፡ቢሮአችን የዐቢይ ግብረ-ሀይል አባልና በሥሩ የተቋቋመው የሀይጂኒና ሳኒቴሽን ንዑስ ግብረ ሀይል ሰብሳቢ ነው፡፡ በዐቢይ ኮሚቴ በሚሰጠው አቅጣጫ መሠረት የራሱን ዕቅድ በማዘጋጀት እስከታችኛው መዋቅር ድረስ አድርሰንና ከሌሎች ባለድርሻ አካላት ጋር በመሆን እየሰራን እንገኛለን፡፡ ኮቪድና ውሀ ከፍተኛ ቁርኝት አላቸው፡፡ ይህንን በሽታ ለመከላከል የግልና አካባቢ ንጽህና መጠበቅ እጅግ ወሳኝ ጉዳይ ነው፡፡ በመሆኑም ካደረግናቸው ድርጊቶች ውስጥ የመጠጥ ውሀ ተቋሞቻችንን ለአግልግሎት ማብቃት ሲሆን ከነዚህም ውስጥ አስቀድሞ በተለያዩ ብልሽት ምክንያት አገልግሎት የማይሰጡትን ባስቸኳይ ተጠግኖ አግልግሎት እንዲሰጡ ያደረግንበት፤ በግንባታ ሂደት የነበሩት ግን መጠናቀቅ በሚገባው ጊዜ ያልተጠናቀቁ ፕሮጄክቶችን ተጠናቅቆ ለአገልግሎት እንዲበቁ ያደረግንበት ይገኛሉ፡፡ የውሀ መሰረት ልማት ተደራሽ ባልሆነባቸው አካባቢዎች ውሀን በቦቴ የማዳረስ ስራ እንዲሁም ለመጠጥ በቀጥታ ሚቹ ያልሆኑ ውሀን በኬሚካል በማከም የማዘጋጀት፤ተጋላጭ የሆኑትን የሕብረተሰብ ክፍሎችን ለመከላከል ከሌሎች እንደ ጤና ትምህርትና መሰል ተቋማት ጋር እስከታችኛው መዋቅር ድረስ በመንቀሳቀስና በየሳምንት እየተገናኘን ሥራን በመገምገም እገልግሎት እየሰጠን እንገኛለን፡፡ አደረጃጀቶቻችን ባብዘኛው ይህን ይመስላል፤ ከዚህ ጎን ለጎን ደግሞ በመዋቅርችን በኩል ስለ ውሀ ተቐማት በየጊዝው መረጃ እያገኘንና እያስተካከልን ነው የምንሰራው፡፡

በተቋማችሁ ደረጃ ይህንን በሽታ ለመከላከልና ለመቆጣጠር ያደረጋችሁ ጥረት ምን ይመስላል?

እንደተቋም ይህንን በሽታ በመከላከል ረገድ የምንሄድበት አግባብ ቅድም ለመጥቀስ እንደሞከርኩት ከመጋቢት ወር ጀምሮ የመጠጥ ውሀ ተቋማት በሙሉ አቅማቸው ወደማመንጨት እንዲገቡ የመረባረብ ስራ ሰርተናል፡፡ እዚያ አካባቢ ተጋላጭ ሊሆኑ ይችላሉ ያልናቸውን 25 ተቋማት ሙሉ በሙሉ ለአግልግሎት ብቁ አድርገናል፡፡ 422 ተቐማት ከአገልግሎት ውጭ የነበሩትን አግልግሎት እንዲሰጡ በማድረግ 1.5 ሚሊየን ህዝብ ውሀ እንዲያገኝ አድርገናል፡፡ በዚህም ከዚህ በፊት በከፍል የሚያገኙት በተሟላ ሁኔታ እንዲያገኙና አስቀድሞ ምንም የማያገኙ ደግሞ በከፍልም እንዲያገኙ አድርገናል፡፡ የፓምፕ ችግር ላለባቸው ከኛ በጀት በማኔጅመንት ወስነን 34 የሚሆኑ ፓምፖችን ገዝተን ፍትሀዊ በሆነ ሁኔታ ደልድለናል፡፡ ከዚህ በተጨማሪ ሮቶችን የውሀ ማከሚያ ኬሚካሎችንና ከምግብ ውጭ የሆኑትን ሌሎች ቁሳቁሶችንም ደግፈናል፡፡ለክልል ጤና ቢሮ ባለ 4 እና 5 ሊትር የሚይዙ 200 ጀርካኖችን ለግሰናል፡፡ አላባ ዞንን ጨምሮ በርካታ ሮቶችንና 400 የሚሆኑ ጀርካኖችን አሰራጭተናል፡፡ እንሁዱም ውሀ መቅጃ ወደ 2000 የሚሆኑትን ሰጥተናል፡፡ የሴቶች ንጽህና መገልገያ ሞዴስ 1150 የሆንና የተለያዩ ሳሙናዎች ከ6600 በላይ ለግሰናል፡፡ ይህም ወደ 29000 የሚሆኑ እጅግ በጣም ተጋላጭ ይሆናሉ ተብሎ ለሚገመቱት ተደራሽ ሆነዋል፡፡ እነዚህንና የመሳሰሉትን ድጋፎች እንደዪኒሴፍ ካሉት አጋሮች ጋር ነው እየተወጣን ያለነው፡፡ የውሀ ማከሚያ ክሚካሎች እንደ አኳ ታበ ክሎሪን እና ፒዩር የሚባሉ ለየዞኖች ተሰራጭተዋል፡፡ በዚህም ከ27000 በላይ ህዝብ ተጠቃሚ ሆነዋል፡፡ ከዚህ ውጭ የማቴሪያል ድጋፍ ጋር በአካል በመገኘት የእጅ መታጠብ ዘመቻን በተለያዩ ቦታዎች ለምሳሌ ሀዋሳ ከተማ አሰተዳደር በሀዲያ ዞን ኮንሶ ዞን በመሳሰሉት የማነቃቃት ስራ ሰርተናል፡፡

የኮሮና ወረርሽኝን በመከላከል ሂደት ከፌደራል ሚኒስቴር መስሪያ ቤታችሁ ጋር ያለው ቁርኝት ምን ይመስላል?

ከሚኒስቴር መስሪያ ቤታችን ጋር ያለው ግኑኝነት ባጠቃላይ ጥሩ ነው፤ ይህንን ችግር ለመቅረፍ የሚያግዝ ድጋፍ በሚመለከት ዕቅድ አዘጋጅተን ልኬናል፤ በዚህ ረገድ የተለያዩ መጠን ያላቸውን የውሀ መያዣ ሮቶች፣ ጀርካኖችንና ፓምፖችን ልከውልናል፡፡ ባሁኑ ጊዜ 28 ሚሊየን የሚጠጋ ብር የተለያዩ የኤልክትሮሜካኒካል ዕቃዎች ተገዝተው እንዲሰጡን ዕቅድ አቅርበናል፡፡ ድጋፉ በዓይነት እንጂ በገንዘብ አለመሆኑ እንደጉድለት ያየንበት ነው፡፡ በበጀት ችግር ምክንያት መድረስ ያልቻልንባችው አካባቢዎች አሉ፡፡ የክልሉ መንግስት በተወሰነ ደረጃ ወደ 5 ሚሊየን ብር ያክል ለማገዝ ጥረት እያደረግ ቢሆንም፡፡ ነገር ግን የውሀ እጥረት ባለባቸው አካባቢዎች ቦቴ በኪራይ በማስገባት የምናቀርበው የውሀ አገልግሎት ውድ ከመሆኑ ጋር የውስጥ አቅማችን እየፈታተን ነው፡፡ ቦቴ የገባባቸው አካባቢዎች ደቡብ ኦሞ ፡- በና ፀማይ፣ ዳሰነች፣ ሀመር ፣ኛንጋቶም አና ጋሞ ዞን ማርታ ጋርዳ ወረዳ ናቸው፡፡ እነዚህ አካባቢዎች ከኮቪድ በተጨማሪ የኮሌራ ወረርሽኝ የታየባቸው አካባቢዎች ናቸው፡፡ ከዚህ ውጭ ለኮሮና ድጋፍ ብላቴ ዙሪያ ካራት ከተማ ማሬቆ ወረዳ ጉራጌ ዞን እንዲሁም ሀዋሳ ከተማ ለኬሚካል ሪጭት ቦቴዎችን አሰማርተናል፡፡ በጎፋ ዞን ዛላ ወረዳ በስልጤ ዞን ምዕራብ አዘርነትና ዳሎቻ ወረዳዎች በዳውሮ ዞን ተርጫ ከተማ ደቡብ ኦሞ ዞን ማሌ ወረዳና ዙሪያ ተጨማሪ ቦቴ አሰማሪተን የውሀ አቅርቦትን እያዳረስን ነው፡፡ እነዚህን ቦቴዎች በማሰማራት በቀን ከ66ሺህ ሊትር በላይ ውሀ ለህብረተሰቡ እያቀረብን እንገኛለን፡፡

ለኮቪድ-19 ተጋላጭ በመሆን ወይንም በመጠርጠር በለይቶ ማቆያ ሥፍራ ለተቀመጡት ስዎች የውሀና ንጽህና አቅርቦት ላይ ያላችሁ ተሳትፎ ምን ይመስላል?

የውሀ አቅርቦትን ስናነሣ እነዚህንም ተቋማት በዋናነት ባማከለ መልኩ ነው፡፡ የሳኒቴሽንና ሀይጂን ንዑስ ግብረ-ሀይል ውስጥ የጤና ቢሮ በምክትል ቢሮ ሀላፊ አማካይኝነት በአባልነት ስለሚሳተፍና በጋራ ስለምንሰራ እነዚህ ተቋማት አስቀድሞ ሲለዩ የውሐ አቅርቦትን በሚመከለት በሚያቀርቡት ጥያቄና በጋራ በምናደርገው ምልከታ መሰረት ሮቶና የውሀ መስመር የማገናኘት ሥራ ተሰርተዋል፡፡ በየዩኒበርስቲዎች አካባቢም ለኳራናታይን በተለዩት አካባቢ ውሀ አቅርቦት እጥረት እንዳይኖር ተቀናጅተን እየሰራን እንገኛለን፡፡

ቅንጂታዊ ስራ ሂደትን የምትገመገሙበትና ግብረ-መልስ የምትሰጡበት አግባብ ምን ይመስላል?

ይህን የህይወት ማዳን ሥራ ስናከናውን ቅንጅታዊ አሰራር መሰረታዊ ስለሆነ እንደበቀደሙ በላላ ቅንጂታዊ አሰራር ማሳኬድ የምታሰብ እንዳይደለ ተገንዝበናል፡፡ ስለዚህ በተቻለ መጠን በተለያዩ አማራጮች እንገናኛለን፤ አንዱ አማራጭ የቴለግራም ዋሽ ክላስተር ከዚህ ቀደም ያመቻችነው በየትኛውም ወረዳ ችግር ሲከሰት ወዲያው ሪፖርት የሚያደርጉበትና እኛም ተቀብለን ምላሽ የምንሰጥበት መስመር በጥሩ ጎን ያየንበት ነው፡፡ ከቅንጅት አንጻር ከዕቅድ ጀምሮ የጋራ ለማድረግ ተሞክረዋል፡፡ ሆኖም ግን እዚህም እዚያም በራስ የመሄድ ዝንባሌዎች አሉ፡፡ ሌላው እያንዳንዱ ሴክተር ለብቻው የሚሰራውን ደግሞ ሌሎች ድጋፍ ከሚያደርጉት አጋሮች ጋር የሚሰራው አለ፡፡ በዓይነት ድጋፍ የሚያደርጉ በርካታ ተቋማት አሉ፡፡ አንዳንዱ ሮቶዎችን ሳኒታይዘር ሳሙናና የመሳሰሉትን በመስጠት የደገፉን ነበር፡፡ ቅንጂታዊ አካሄዳችን በጋራ አቅዶ መስራ ብቻ ሳይሆን ሀብትንም በመጋራት ይገለጻል፡፡ ለምሳሌ አንዳንድ ተቋማት በዓይነት ድጋፍ ሲያደርጉልን ያ ንብረት ለጤና የሚጠቅም ከሆነ በቀጥታ ለነርሱ በመስጠት እንጋራለን፡፡ ከዚህ ጋር በተያያዘ የሳኒታይዘር ምርት በራሳችን ደረጃ ለማምረት የሚያስችል ተቋም ገንብተን በሚቀጥለው ማክስኞ ክቡር ርዕሰ መስተዳድር ባሉበት ልናስመርቅ አቅደናል፡፡ ይህም በዘርፋችን በተለያዩ ሁኔታ ለስራ የተሰማሩት ለበሽታው ተጋላጭ እንዳይሆኑ የሳኒታይዘር አቅርቦት እንዳያጥርም ጭምር ታስቦ ነው፡፡ በተለይ በውሀ ግንባታ በማዕድን ማውጣት እና በመሳሰሉት ተጋላጭነትን ከፍ በሚያደርጉት ስራዎች ዘርፍ የሚሰማሩትን ለመከላከል ታስቦ ነው፡፡ የማዕድ ማጋራትን ድጋፍ ከመተግበር አንጻር በቢሮአችን በዝቅተኛ ክፊያ የሚሰሩት ግን በአንጻሩ በረከት ያለ ቤተሰብ ያላቸውን እንዲሁም በተቋማችን ዙሪያ ያሉትን አቅመ ደካሞችን በመለየት እስከ 100 ለሚሆኑት ድጋፍ አድርገናል፡፡ ኮቪድን እየተከላከልን ሀገራዊ የሆኑት ሌሎች እንደ ደም ልገሳና አረንጓዴ አሻራ ዘመቻ ላይ ጎን ለጎን እየተሳተፍን እንገኛለን፤ ይህም ለቅንጂታዊ ሥራ የራሱ የሆነ በጎ ገጽታ ያሳየ ነው፡፡ ዋናው የቅንጅታዊ ሥራ መነሻችን ህይወትን ማዳን እንደሆነ በማውሳት ነው ወደዚህ የገባነው፡፡ ከቅንጅት አንጻር እየተመካከርን እያሻሻልን የመጣንበት አግባብ ነው ያለው፡፡ ከክትትልና ግምገማ አኳያ በየሳምንት እየተገናኘን ስራችንን የምንገመግምበት አሰራር ተዘርግተዋል፡፡ በንዑስ ግብረ ሀይል ደረጃ ያሉት የጤና ቢሮ ውሀ ሥራዎች በሳኒቴሽንና ሀይጅን ዙሪያ የሚሰሩ መንግስታዊ ያልሆኑ ተቋማትና ሌሎች ባለድርሻ አካላት ለማንኛውም ስራ ወደታችኛው መዋቅር ስንወርድ የኮቪድ ስራ በመከታተልና በመደገፍ እዚያው የምንችለውን ግብረ መልስ በመስጠት አውራ የሆኑ ጉዳዮችን ይዘን በመመለስ በንዑስ ግብረ ሀይል ደረጃ ደግም እንገመግማለን፡፡ አሁን ኢንቴርነት ተቋረጠ እንጂ ከዞንና ልዩ ወረዳ መዋቅሮቻችን ጋር በዙምና ቪዲዩ ኮንፈራንስ በየሳምንቱ እንገናኝና ስለስራው መረጃ እንለዋወጥ ነበር፡፡ በዚህ ሂደት ውስጥ ማነቆዎች ምን እንደነበሩ የተፈቱትም ካሉ እንዲሁም የከፍተኛ አመራር ትኩረት የሚሹ እንደሆነ ለይተን ገምግመን አቅጣጫ እናስቀምጣለን፡፡ ለምሳሌ የሮቶ የፓመፕ እና ሌሎች ግብአቶች ጥያቄዎች ቀርቦ እየቻልን ያልፈታናቸው ከሆኑ ሂስ እየወሰድን እያስተካከልን ነው የመጣነው፡፡

በርካታ የውሐ ተቋማት ተበላሽቶ የነበሩት በቅርቡ ተጠግኖ ስራ ላይ እንደዋሉ ቅድም አንስተው ነበር፡ በዚህች አጭር ጊዜ የዚያን ያህል ሥራ እንድትሰሩ ያነሳሳችሁ ምንድነው?

በዋናነት ለዚህ አፈጻጸም አስተዋጽኦ ያደረገው ስራዎችን ተከፋፍለንና ተቀናጅተን መስራታችን ነው፡፡ ችግሮችን በክላስተር ደረጃ ተከፋፍለን አይተን ምላሹ ምን መሆን አለበት ብለን ደግሞ በጋራ መረባረባችን ነው፡፡ ሁሉም ክትትል ያደርጋል፤ በተለይ ደግሞ ከውሀ ስራዎች ጋር የፈጠርነው የስራ ትብብር የነርሱን ማሽነሪዎችን ለዚህ ጉዳይ ተጠቅመናል፡፡ ሌሎች አጋሮች፡- እንደ ዩኒሴፍ፣ ፕፒል እን ኒድ፣ ዎርልድ ቭዥን የመሳሰሉትን በአቅርቦት ታችኛውን መዋቅር እንዲደግፉ በማድረግ በርካታ ስራዎችን ሰርተናል፡፡ እንደጉድለት መወሰድ ካለበት ከዚህ ቀደም በዚህ ልክ እየሰራን ቢሆን ኖሮ የተቋሞቻችን የማስፈጸም አቅምና የአገልግሎት ተቋማት ምርታማነት ምን ያህል ባደገ ነበር ያሰኛል፡፡ ከዚህ የሚወስደው ብዙ ዕድሎችን አምክነናል ብዬ ነው፡፡ ለወደፊት ግን በርካታ ትምህርት ወስደናል፡፡ በመሆኑም በቀጣይ ይህንን ልምድ ወስደን የታችኛው መዋቅርና ህዝባችንን ጥያቄ በጊዚ ለመፍታት ጥሩ ተነሳሽነት ፈጥሮልናል፡፡ ጠረፍ እስከ ጠረፍ የጥገና ቡድናችን የተበላሹ የውሀ ተቋማትን እየጠገኑ ምላሽ እየሰጡ ናቸው፤ የፋይናንስ ዘርፍም ለነርሱ የሚያስፈልገውን ድጋፍ እነርሱ እዚህ መምጣት ሳይጠበቅባቸው እዚያ እንዲላክ እያደረገ ስራውን እናፋጥናለን፡፡ ሂደቱም ሁሉም ለኮቪድ የሚደረግውን ምላሽ በባለቤትነት ስሜት እንዲይዝ አድረገዋል፡፡ባጠቃላይ በዚህ ልክ ሥራዎች በዐቢይና ንዑሳን ግብረ ሀይሎች ዕየተገመገመ መሰራት ለሌሎች ሥራዎችም ጥሩ አቅም የፈጠርንበት ሁኔታ ነው የታየው፡፡

ቁልፍ መረጃ ሰጭ ስምንት፡- ክልል ፋይናንስ ቢሮ

የናንተ ቢሮ የኮቪድ-19 መከላከልና ቁጥጥር ሂደት ያላችው አጠቃላይ ቁርኝት ምን ይመስላል?

- በማሰተባበር ረገድ ከሌሎች ጋር በአጋርነት ከመስራ አካያ ተቐማችሁን ለተልዕኮ ብቁ አድርጎ በማዘጋጀት በጋራ ማቀድና መምራት በመሳሰሉት

የኮቪድ በሽታ ከመጋቢት 03 ጀምሮ በሀገራችን ከተከሰተ ጊዜ አንስቶ እንደ ክልል መንግስት ዐቢይ ኮሚቴ እንዲዋቀር ተደርገዋል፡፡ የኛ ቢሮ በዐቢይ ኮሚቴ አባል ሆኖ በዚያው ሥር ደግሞ በንዑስ ኮሚቴ የሀብት ማሰባሰብ ሥራ ይመራል፡፡ ሀብትን ለማሰባሰብ የተለያዩ ባለሙያዎች ከኛ ቢሮና ከሌሎች ተቋማት ያካተተ የቴክኒክ ኮሚቴ በማደራጀት በ25 ታችኛው መዋቅርም ተመሳሳይ ኮሚቴ በማዋቀርና ግኑኝነት በማድረግ እንዲሁም ከፌደራል ገንዘብና ኢኮኖሚ ትብብር ሚኒስቴር ጋር ረቡዕ ዕለት በየሳምንቱ በዙምና ቪድዮ ኮንፈራንስ በመገናኘት የኛን ሪፖርት በመስጠትና የነሱንም ግብረ-መልስ በመውስድ ከፍተኛ ሥራ ሲሰራ ቆይተዋል፡፡ ከዚህ አንጻር የሀብት ማሰባሰብ ስራን ተቋማችን በበላይነት በሰብሳቢነት እየሰራ ባለው ላይ እሰከታችኛው መዋቅር በዓይነት በጥሬ ገንዘብና በቁሳቁስ እስካሁን ድረስ 324 ሚሊየን ብር ለመሰብሰብ ተችለዋል፡፡ ለዚህ አገልግሎት የሚሆን አካውንት በያንዳዱ መዋቅር እስከልዩ ወረዳ ድረስ ተከፍተዋል፡፡ በተከፈተው አካውንት የተሰበሰበው ጥሬ ገንዘብ እንዲገባ ተደርገዋል፡፡ ይህ የባንክ አካውንተ መንግስትና በግል አካውንተ በየአካባቢው ባለው ባንኮች ነው የተከፈተው፡፡ በዚህ የሀብት ማሰባሰብ ወቅት የታየው የህዝቡ ከፍተኛ የሆነ መነሳሳትና የትብብር መንፈስ ነው፤ ገንዘብ ያለው ገንዘቡን ሌላው በዓይነት ከብት ጥጃ በቅሎ ከነሙሉ ዕቃ ወተታም ላም ሁሉ ሳይሰስቱ እያመጡ ያስረከቡበትን ስናይ እጅግ የሚያስደንቅ መነሳሳት በተለይ የክቡር ጠቅላይ ሚኒሰትር የማዕድ ማጋራት ቅስቀሳ መነሻነት ወቅታዊ ሁኔታ በፈጠረው በኑሮ ችግር ውስጥ ያሉትን ፤ አስቀድሞ ህዝብ በብዛት በሚገለገልባቸው ተቋማት እንደ ሆቴሎች ውስጥ የሚሰሩት አሁን ያንን ሥራ ማከናወን ባለመቻላቸው ከምግብ ችግር እንዳይጋለጡ ህብረተስብ ለጥሪው የሰጠው ምላሽ ከሀገር አልፎ ለአፍርካ ሀገራትም ተሞክሮ ሊሆን ይችላል የሚል እምነት ነው ያለኝ፡፡ ስለሆነም በዚህ የሀብት ማሰባሰብ ሂደት ያየናችው በጎ እሴቶች ለወደፊትም ማሳደግ የሚገቡ ናቸው፡፡ ከሌሎች ባለድረሻ አካላት ጋር መቀናጀት አስፈላጊነት ሲነሳ እንደሚታወቀው ክልላችን ከሁለት የአፍርካ ሀገሮች ጋር ድንበር የሚትጋራ እንደመሆኗ በድንበር በኩል የሚገቡ ዜጎች እዚያው ኳራናታይን ሆነው አስፈላጊ የመጠለያ የህክምናና የምግብ አገልግሎት እንዲያገኙ ከጸጥታና ጤና ተቋማት ጋር ተቀናጅተን የምንሰራበት ሁኔታ ነው ያለው፡፡ ለምሳሌ እኛ ሀብት ለማሰባሰብ አሰቀድሞ የግንዛቤ ማስጨበጫ ስራ ካልተሰራ ሀብት ሰው ሊሰጥ አይችልም፡፡ ግንዛቤ ያለው ሰው ነው ሀብት አውጥቶ መስጠት የሚችለው፤በሌላ ጎን ደግሞ ህብረተሰቡ ግንዛቤ ኖሮት ራሱን ለመከላከል ካልጣረ እኛ ሺህ ጊዜ ሀብት ብናሰባስብ ጥቅም አይኖርም፡፡ በኛ ኮሚቴ ሥር ከሀብት ማሰባሰብ ውጭ የበጎ ስራ የሚሰሩ ንዑስ-ንዑስ ኮሚቴ አለ፡፡ ይህ ደግሞ የሚሰራው ህብረተሰቡ ስለበሽታው በቂ ግንዛቤ እንዲኖረው የቅስቀሳና ግንዛቤ ማስጨበጫ ትምህርት የሚሰጡ ናቸው፡፡ በዚህ መልክ አቀናጅተን እየሰራን ነው ያለነው፡፡

ይህንን የኮቪድ -19 ስራ በዚህ መልክ እንድትሰሩ መነሻ የሆነ ነገር ምንድነው?

አንዱ መነሻ የሆነው ይህ ወረርሽን በዓለም አቀፍና በሀገር ደረጃ ከተከሰተ በኋላ ኮሚቴ ተቋቋሞ መስራት አለበት ተብሎ ከፌደራል መንግስት/ ከጠቅላይ ሚንስትር አቅጣጫ ወርደው በየክልልና ታችኛው መዋቅር ለማደራጀት ታስቦ በሁሉም መዋቅር ዐቢይና ንዑሳን ኮሚቴዎች አቋቁሞ ወደሥራ ገብተናል፡፡ ስለዚህ አቅጣጫው ከፌደራልና ከክልል መንግስት የወረደ አቅጣጫ ነው፡፡ ዜጎችን መታደግ አለብን የሚል የክልል መንግስትም ውሳኔ ነው፡፡ ሁለተኛው ደግሞ እኛ እንደ ሀብት ማሰባሰብ ኮሚቴ የፌደራል ግንዘብ ሚኒስቴር መመሪያ ቁጥር 63/2012 የሚል አዘጋጅተዋል፤እኛም ያንን ተከትለን መመሪያ ቁጥር 31/2012 የሚል አዘጋጅተናል፤ያንን መመሪያ ተክትለን የሀብት አፈጻጸም ሁኔታዎችን የምንከተልበት አግባብ ነው ያለው፡፡ ሶስተኛው መነሻ የተለያዩ የአስቸኳይ ጊዜ አዋጆች ናቸው፡፡ እነዚህ አዋጆች ምን ምን መተግበር እንዳለባቸው፤ እነዚህን በአዋጀ የተቀመጡትን አስገዳጅ ሁኔታዎችን ለመተግበር ለዜጎች ምን ሊደረግ ይገባል፤ አቅም ያለውን የሌለው አለ፤ ሥራ የፈታ አለ፤እነዚህን ዘጎች እንዴት መታደግ ይቻላል? መንግስት ብቻውን ይህንን ሁሉ ሊወጣ ይችላል ወይ? ይህ አይቻልም፤ ካልተቻለስ? ህዝቡን ማስተባበር አለብን የሚለው አቅጣጫ ተይዞ በነዚህ በሶስቱ መነሻነት ነው ወደሥራ የተገባው፡፡

የኮቪድ -19 እያስከተለ ያለውንና አሁን ያስከተለውን ጫና እንዴት ገመገማችሁ?

ጫናው በጣም ከባድ ነው፤ አሁን እኛ ኢኮኖሚ ዘርፍ እንደምንመራ ሁሉ ተውና በሽታው በኛ ክልል ተከስቶ ገና ወሬ ተሰምቶ ባለበት ሁኔታ ሰው ተደናግጦ ከኢኮኖሚ መስተጋብር በመላላቱ በገቢ ማሰባሰብ ሂደት ከፍተኛ ተጽዕኖ ፈጥሯል፡፡ በሽታውን ለመከላከልና ለመቆጣጠር በተቀመጠው አቅጣጫ እና ሰዎች በበሽታው ባደረባቸው ስጋት ምክንያት እንቅስቃሴ በመገደቡ በኢኮኖሚ ሴክተር ላይ እየታየ ያለው ጫና አሳሳቢ ነው፡፡ ሌላው አስጊ ጉዳይ ማህበራዊ ቀውስ እንዳያስከትል ነው፤ ካሉን 25 መዋቅሮች ውስጥ ከ18 መዋቅር በላይ ለሠራተኞች የወር ደመወዝ መክፈል ያልቻሉበት ሁኔታ ሁሉ እየተከሰተ ነው፡፡ ይህንንን የማነሳው በበጀት አካል የታወጀውን ገቢ አሰባስበው ወደ ካዝና ማምጣት ያልተቻለበት፤ መንግስትም ችግሩ ከተከሰተበት ጊዜ አንስቶ በአንዳንድ ዘርፎች የታክስ ስረዛ ወይንም እፎይታ በመስጠቱ፡- ሀዋሰ ከተማ ላይ ከ400 ሚሊየን ብር በላይ ታክሲ ተሰርዘዋል፡፡ በመሆኑም ከግንቦት ጀምሮ ደመወዝ መክፈል አልቻለም፡፡ ትላልቅ በተባሉት ዞኖችና ከተሞች ቀድሞ የተሻለ የኢኮኖሚ እንቅስቃሴ ባለባቸው አካባቢዎች የፈጠረው ጫና ከባድ ነው፡፡ እኛ እየገመገምን በመጣንበት አግባብ ከፍተኛ ጫና ያለ ቢሆንም የዚያን ያህል ደግሞ መዘናጋት አለ፡፡ ከፍተኛ የሆነ የግንዛቤ ችግርም አለ፡፡በመሆኑም እየሰራን ያለነው እጅግ አስከፍ ሁኔታ ሊፈጠር ይችላል በሚል እሳቤ ነው፡፡

እንደቢሮ የራሳችሁን ሠራተኞችንና ደንበኞቻችሁን ከኮቪድ-19 ለመከላከል ምን እየሰራችሁ ነው?

እንደ ቢሮ እንደሚታዩት ከበር ጀምሮ በሽታውን መከላከል የሚያስችል ስራ እየሰራን ነው፤ መጀመሪያ ሰራተኞቻችን በሁለት በመክፈል አስቀድሞ የታወቀ የጤና ችግር ያለባቸውንና በዕድሜ የገፉትን ሠራተኞችን ከቤታቸው ሆኖ ሥራውን እንዲሰሩ በክብር ሸኝተን ሌሎች ደግሞ ተፋፍጎ እንዳይሰሩና በመካከል የሚፈለገው ያህል አካላዊ ሪቀት እንዲኖር ለማድረግ በፈረቃ በጥዋትና የከሰዓት እንዲሰሩ በማድረግ፤ ሁሉም ሰራተና ቢሮ ሲገባ እጅ እንዲታጠብን በየመካከሉ ደግሞ እጅን በሳኒታይዘር እንዲያጸዱ ለሁሉም ሰራተኛ ሳኒታይዘር ና የአፍና አፍንጫ መሸፈኛ ጭንብል ገዝተን ሰጥተናል፡፡ ከንክኪ ነፃ የሆነ የእጅ መታጠቢያ ማሽን ከቴክኒክ ሙያና ስልጠና ኮሌጅ ገዝተን በግቢያችን መግቢያ ላይ አሰቀምጠን ሁሉም ሰራተኛ ግቢ ሲገባ ታጥበው እንዲገባ እያደረግን ነው፡፡ ሰራተኞቻንንም ሆኑ ባለጉዳይ ግቢ ስገቡ ታጥበው እንዲገቡ ውሀና ማስታጠብያ ማስቀመጥ ብቻ ሳይሆን የሚከታተል ባለሙያ ሁሉ መድበን እየተከታተልን ነው፡፡ ቢሮአችን እንደሚታወቀው በርካታ ደንበኛ ያለበት እንደመሆኑ መጠን አንደኛ ቢሮ በሚመጡበት ጊዜ ማስክ እንዲያደርጉ አስገዳጅነት አስቀመወጠን፤ አካላዊ ቅርርብ እንዳኖር በማድረግና ምልልሱን ለመቀነስ የተለያዩ የግኑኝነት ዘዴዎችን እንደ ኤሌክትሮኒክስ ዳብዳቤ ልውውጥ ቪዲዪ ኮንፈራንስና የመሳሰሉትን የቴክኖሎጂ ትሩፋቶችን በመጠቀም እያሰተናገድን ነው ያለነው፡፡

ይህንን የውስጥ መከላከልና ቁጥጥር ስራ የሚያስተባብር በቢሮ ደረጃ የተለየ ባለሙያ ተመድበዋል?

አዎን ለዚህ የተመደበ ሰው አለ፤ አንደኛ የቢሮ የጽ/ቤት ሀላፊ አቶ ዘርፉ አጥናፉ ይህንን በዋናነት ይመራሉ፤የሰው ሀብት አስተዳደር በሰው ሀብት በኩል ያለውን የሚመሩ ሆነው የአስተዳደርና ፋይናንስ ዘርፍ ሃላፊ አቶ ታምሩ ታፌ የቴክኒክ ኮሚቴውን ከመምራት ባሻገር ይህንንም በበላይነት እንዲከታተሉ አድርገናል፡፡ በእንደዚህ ዓይነት ሁኔታ ከመቀናጀትም በላይ ደግሞ እንደቢሮ ሃላፊ እነርሱ ያመጡትን በጋራ እየገመገምን አቅጣጫ እያሰቀመጥን እየመራን እንገኛለን፡፡

ቅንጅታዊ አሰራርን ከማሳለጥን አንጻር በጋራ የታቀደውን በጋራ የመምራትና ግብረ መልስ የመስጠት እንዲሁም ዕቅድን የመከለስ አዝማሚያ ምን ይመስላል?

እንደሚታውቁት በሀገራችን የኮሚቴ ሥራ አንደንዴ ፈጥነው የመውጣት አንዳንዴ ደግሞ የመጎተት አዝማሚያ ይታያል፡፡ አሁን ያለው እንቅስቃሴ ጅምር ላይ እንዳለው አይደለም፤ ጅምር አካባቢ በጣም የተቀጣጠለ በሳምንት ሶስት ጊዜ እየተገናኘን የምንወያይበትና ከታችኛውም መዋቅር እንደዚሁ በሳምንት ሀሙስ ሀሙስ በቪዲዮ ኮንፈራንስ የምንገናኝበት ሁኔታ እንዲሁም በዙም ስብሰባዎችን የምንገናኝበት፤ ሴክተሮችም እንደዚያው ነበር፤ አሁን ግን ከወቅታዊ ሀገራዊ ጉዳዮችም ጋር ተያይዞ መቀዛቀዝ ይታያል፡፡ዕቅድ ከማቀድና ያንን ከታችኛው መዋቅር ጋር የጋራ አድርጎ ከሜሄድ አንጻር ችግር የለብንም፡፡ ነገር ግን ከመተግበር አንጻር አብሮ ክትትልና ግምገማ አድርጎ ከሜሄድ አንጻር የእግር መጎተቶች አሉ፡፡ አንዳንዴ ወደ ፊት አንዳንዴ ወደ ሀላ የማለት አዝማሚያ አለ፡፡ ምን ማሳያ አለ?

ዕቅድን ወደታች ስለማውረዳችን የተደራጀ ሰነድ አለን፡፡ ይህንን ሥራ ስንሰራ ከሌሎች ሴክተሮችም ጋር በጋራ ነው የምንሰራው፡፡ ለምሳሌ ከከተማና ቤቶች ልማት ቢሮን የጤና ቢሮ ጋር በመሆን በኛ አስተባባሪነት ከፌደራል ሚኒስቴር መስሪያ ቤት ጋርም በመሆን በጂፒ ኤስ ኮርዲኔት በማዘጋጀት የበሽታ መከላከልና ቁጥጥር ሥራ ለመሥራት በሀርድ ኮፒና በሶፍት ኮፒ ማዘጋጀታችን አንዱ ማሳያ ነው፡፡

የክትትልና ግምገማ ሥርዓትስ በሁለቱም በኩል ምን ይመስላል?

የክትትልና ግምገማ ሥራ እየተሰራ ነው፡፡ የአቢይ ኮሚቴ ሥራ ግምገማ በሚመለከት የክልሉ ብልጽግና ፓርቲ ጽ/ ቤት ጭምር አመራሩ ተግባሩን ከሌሎች የልማት ሥራዎች እንደ ግብርና ሥራ የአረንጓዴ አሻራ እና ሌሎችንም ጋር አቀናጅተው እንዲያከነውኑ አቅጣጫ አስቀምጦ አፈጻጸሙን በቼክ ሊስት ይከታተላል፡፡ እንደሀብት አሰባሳቢ ኮሚቴ ደግሞ ይህ ተሰብስበዋል የተባለው ሀብት በትክክል መኖር ያለመኖሩን ገንዘቡ በካሽ ንብረት በየዓይነት መኖርን የምንከታተልበት እቦታው በመገኝት ምልከታ እናደርጋልን፡፡ ይህ በኛ ብቻ ሳይሆን የአስቸኳይ ጊዜ አዋጅ አፈጻጸም በሚከታል በፌዴራል ግብረሀይል ጭምር ክትትል አድርጎ ለው አፈጻጻም አበረታችና ለሌሎች ክልሎችም ተሞክሮ መሆን እንደሚቻልም ግብረመልስ የክልላችን ምክትል ርዕሰመስተዳድር ባሉበት ሰጥተውናል፡፡ ስለዚህ የሀብት አሰባሰብ ሥርዓታችን በዚህ ልክ ምስክርነት ያገኘ መሆኑን እና በዶክሜንትም ሊረጋገጥ እንደሚቻል ደጋፍ ሰነድ ሊሰጣችሁ እችላለሁ፡፡ በተከፈተው አካውንት ምን ያህል ገንዘብ ገባ፤ ምን ያህል ንብረት ተሰበሰበ፤ እንዲሁም በትክክል ምን ያህል ሰው ተገቢ ድጋፍ አገኘ? የሚለውን ቼክ ሊስት አዘጋጅትን ክትትል እናደርጋለን፡፡

እንዳንድ ቦታዎች ደግሞ ጉድለት አይተናል፡- የተሰበሰበው ገንዘብና ንብረት የት አለ ብለን ስንጠይቅ አሰባስበን ነበር ግን አሰራጭተናል የሚል የማስመሰል ሪፖርት ያቀርባሉ፡፡ባብዘኛው ግን ለኔ ለወገኔ ብለው በተነሳሽነት የሚሰሩ ናቸው፡፡ የድጋፍን ክትትል ሥራ እንደወትሮ በአካል በመንቀሳቀስ ማድረግ የማይደገፍ ከመሆኑ ጋር ቴክኖሎጂን በመጠቀም በዙም በቪዲዮ ኮንፈራንስና ባዘጋጀነው የግሩፕ ቴሌግራም አካውንት መረጃ በየቀኑ በመለዋወጥ እንገመግማለን፡፡

ቅድም እንዳሉት ከፌዴራል የተላከው አዲሱ የግዥ መመሪያ ምን አዎንታዊ አፈጻጸም ይዘው መጥተዋል?

የመንግስት ግዥ አፈጻጸምን በሚመለከት የክልሉ መንግስት የራሱ የሆነ በአዋጅ የተቋቋመ 146/2004 እና 28/2010 የግዥ መመሪያ አለ፡፡ ሆኖም ግን በግዥ አሁን የኮቪድ በሽታን ለመከላከል የነበረው የግዥ አፈጻጸም ረጅም ጊዜ ስለሚጠይቅ የቀድሞ የፋይናንስ ቢሮክራቲክ አሰራር ተከትሎ ጨረታ መጥቶ ይገዛ ቢባል የሰው ህይወት ማትረፍ ስለማይቻል የክልሉ መንግስት በካቢኔ አስወስነው ቀጥታ አስቸኳይ ግዥ በኮሚቴ እንዲፈጸም ሆኖ አፈጻጸም ወደፊት ኦዲት እንደሚደረግ ተገልፆ ለሁሉም የጤናና ለሚመለከታቸው መዋቅሮች እስከወረዳ ድረስ ተፈቅዶላቸው ሰርኩላር ተልከዋል፡፡ ይህ በክልሉ መንግስት የተወሰነ ውሳኔ ነው፡፡ አፈጻጸሙ እንዴት ነው የሚለው ክትትል ይደረጋል፡፡ መመሪያው ይበልጥ ትኩረት ያደረገው የተሰበሰበው ሀብት የግለሰብም ይሁን የመንግስት ብክነት እንዳይኖረው የመንግስትን የፋይናንስን ሥርዓት ተከትሎ እንዲሄድ በልዩ ልዩ ርዕስ ገቢ ሆኖ በአይበክስ ሲስተም እንዲመዘገብ፡፤ በቀጣይ ኦዲት እንደሚደረግና የመንግስትን የገቢ መመዝገቢያ አርቪ (ገቢ በሞዴል 19 እና ወጪ በሞዴል 22 )እንዲጠቀም ባጠቃላይ የመንግስትን አሰራር ሥርዓት እንዲከተል የሚያደርግ ሆኖ የተዘጋጀ ነው፡፡ ከዚያ አንጻር አንዳንድ ብዥታ ከመቅረፍ የተከደበትና ከፌዴራል እኛ ተቀብለን ያካተትናቸው በተለይ ደግሞ እነዚህ በቀላሉ ሊበላሹ የሚችሉትን የምግብ ቁሳቁሶችን እንደፓስታ ማኮሮኒ ዘይት ስኳር ጨው እና የመሳሰሉት በስጦታ የሚሰጡት በርካታ ንብረቶች ሳይበላሹ ለተጠቃሚ ተሰራጭተው ተመን ወጥቶላቸው በሪፖርት እንዲገለጹ በሀገር አቀፍ እይታ የተዘጋጀውን በኛ ልክ አሻሽለን ያወረድነው ነው፡፡ በዚህ አግባብ የግዥና የፋይናንስ አሰራርን የፈታንበት አግባብ ነው፡፡

በክትትልና ግምገማ ሂደት የታየ ክፍተትና የወሰዳችሁት የማስተካከያ እርምጃ ካለ?

አንደኛ ክትትልና ግምገማ ባደረግንባቸው በተለያዩ ዘዴዎች በቴልግራም በዙም ኮንፈራንስና በሌሎች ያገኘነውና ይስተካከል ያልነው ከአንዳንድ አካባቢና ሰዎች ሲነሳ የነበረው በእኛ አካባቢ በሽታው ባልተከሰተበት ሁኔታ ለምንድነው ሀብት የሚሰበሰበው? የተሰበሰበውም ሀብት ይከፋፈል፤ ይሰራጭ የሚል አንዳንድ አካባቢ እንደዚህ እያሉ ሀብትን ለማሰባሰብ የተሰማሩትን የማሸማቀቅ ሁኔታ የታየበትን በተቃራኒው ይህንን ሀብት ለራሳቸው ሊጠቀሙ ነው በማለት የተዛባ አስተሳሰብ የማራመድ ሁኔታ አንዱ መታረም የሚገባው እንደሆነ በመገንዘብ በአካል ታች ወርደን ሁሉ እንዲታረም ማስተካከያ አስተያየት የሰጠንበት፤ከዚሁ ጋር በተያያዘ አንድ በበሽታው እንደተያዘ የተጠረጠረ ግለሰብ በሞያሌ አቅጣጫ መጥቶ ሀላባ ላይ ሲያዝና ከእርሱ ጋር ግኑኝነት አላቸው የተባሉት በርካታ ቁጥር ያላቸው ወደ ለይቶ ማቆያ ሲገቡ ሁሉም መተረማመስ ሲጀምር፡ እኛ ስለበሽታው ያልነው ነገር እርግጠኛ መሆንን ያስረገጥንበት ሁኔታ መኖሩ፤ ከዚያ ውጭ የግኑኝነት አግባብና ወርደን ባየንበት ወቅት የታዘብነው የሀብት መለገስ የሀብታሞች ብቻ አድርጎ የማየት ሁሉም በአቅሙ ድርሻውን እስከማዕድ ማጋራት ድረስ መሄድ እንደሚገባው ግንዛቤ ያልተወሰደበት፤ በዚህም በተለያዩ የህብረትሰብ ክፍሎች በተለይ አርሶና አርብቶአደሮች አካባቢ በአቅማቸው ሊለግሱ የሚችሉ ግብዓት መኖሩን ተረድተው እንዲሳተፉ መቀስቀስ አስፈላጊ መሆኑን ያስረዳነበት ሁኔታ ነበር፡፡

አሁንም ከግንዛቤና ትግበራ አንጻር በተለይ ከማስክ አጠቃቀምና አካላዊ ሪቀትን በመጠበቅ ሰፊ ክፍተት እንዳለ እኔም ወደተለያዩ አካባቢዎች በተንቀሳቀስኩበት ጊዜ ያየሁት እናንተም ሳታዩ እንዳልቀራችሁ፤ ይህ ክፍተት ባስቸኳይ ሊስተካከል የሚገባ እንደሆነ፤ በርግጥ እዚህ ሀዋሳ አካባቢ የምናየው እስከነጉድለቱም ቢሆን የተሻለ እንደሆነ ጉድለቱ በፍጥነት ካልታረመ ዜጎቻችን ለከፋ ችግር ማጋለጥና የእኛም ልፋት በከንቱ እንዳይቀር መታረም እንደሚገባቸው በድጋፍና ክትትል ወቅት አስተያየት ሰጥተናል፡፡

ለኮቪድ-19 ምላሽ ለመስጠት በተደረገው ርብርብና ሂደት እንደመልካም ተሞክሮ ሊወሰድ የሚችል ጉዳዮች ካሉ?

እርስዎ ቅድሞ ለማንሳት እንደሞከሩት እንግዲህ አንደኛው አብሮነትን በጣም ያጠናከረ ነው፡፡ የኢትዮጵያዊነትን መተሳሰብ አብሮ እንብላ እንጠጣ የሚባለውን በተግባር ያረጋገጥንበት፡ ትላልቅ ህንጻዎችን ጭምር ለዚህ አገልግሎት እንድንጠቀም ለአብነት ከዲላ አንድ ባለሀብት ደውሎልኝ የእኔን ህንጻ ወስዳችሁ በበሽታው የተጠረጠሩትን ማቆያ አድርጉ ያለበትን ሳሰላስል ህብረተሰቡ ለወገን ያላቸውን ተቆርቋሪነትን የሚያሳይ ፤ ዜጎቼ ከተረፉ እኔ ነገ ሰርቼ አገኛለሁ የማለት መልካም ሃሳብ መሆኑ የአብሮነት ስሜት ያዳበረ መሆኑ፤ ሁለተኛው የቴክኖሎጂ አጠቃቀም ሥርዣታችን በጣም የተቀየረበት ነው፡፡ ብዙውን ጊዜ ከቴክኖሎጂ የራቁት ሰዎች ከቴክኖሎጂ ጋር እንዲቀራረቡ ያደረገ ክስተት መሆኑ፤ ነገሮችን ባጠረ ጊዜ ተደራሽ ማድረግ ብቻ ሳይሆን ሀብትንም በቁጠባ እንድንጠቀም አድርጎናል፡፡ ለተለያዩ ስብሰባዎች ከዞኖች እያንቀሳቀስን የምናወጣውን ገንዘብ ወጪ በማሰቀረት እሰከ ሁለት መቶ ሚሊየን ብር ወጪ አስቀርተናል፡፡ በተለያዩ መዋቅሮች በኩል በርዕሰ መስተዳድር በእርሻና ተፈጥሮ ሀብት ልማት ቢሮ እና በሌሎችም በኩል ተልዕኮን ለማስፈጸም የሚደረገው የስብሰባ ምልልስ ቀርቶ በቴክኖሎጂ በተደገፈ ስብሰባ ከቦታቸው ሳይንቀሳቀሱ በሚያደርጉት ውጤታማ ስብሰባ ማድረግ ተችሏል፡፡ እንዳንዱ እዚህ ድረስ መጥቶ በአካል ስብሰባ ውስጥ ሆኖ ግን በተግባር የፌስቡክ የሚጎረጉረው ሁሉ ዛሬ በቴክኖሎጂ በኩል በሚደረገው ቨርቸዋል ስብሰባ ሁሌንተናውን ሰጥቶ መከታተል መቻሉ በእኔ እይታ ሌላው ጠቃሚ ውጤት ነው፡፡ በዚህ ዓይነት ሂደት ብዙ ስብሰባዎችን አድርገናል፡ በተለይ ሰው ሰዓትን ከማክበር አንጻር በጊዜ የመገኘት ባሕል የዳበረበት ሁኔታ ነው የተፈጠረው፡፡ሌላው በስተመጨረሻ የማነሳው የግል ንጽህና መጠበቃችን ስርዓት መሻሻሉ ከዚህ በፊት በየጊዜው ሰውን የሚያስቸግሩ በሽታዎችን እንደታይፎይድ ታይፈስ እና አሜባ የመሳሰሉትን ታሚሜያለው የሚሉትን ከቤትም ጀምሮ ሰምቼ አላውቅም፡፡ ይህ የሚያመላክተው ባንድ ችግር ውስጥ ሆነን ሌላ አዎንታዊ ውጤት ማግኘታችን ነው፡፡ ይሁን እንጂ አሁንም በዙ ሪቀት መጓዝ እንደሚያሰፈልግ አውቀን እነዚህን በጎ ልምዶችን አጠናክረን መቀጠል ና ማስቀጠል አለብን እላለሁ፡፡

ቁልፍ መረጃ ሰጭ ዘጠኝ

የእርሻና ተፈጥሮ ሀብት ልማት ቢሮ

የናንተ ቢሮ የኮቪድ-19 መከላከልና ቁጥጥር ሂደት ያላችው አጠቃላይ ቁርኝት ምን ይመስላል?

- በማሰተባበር ረገድ ከሌሎች ጋር በአጋርነት ከመስራት አኳያ ተቋማችሁን ለተልዕኮ ብቁ አድርጎ በማዘጋጀት በጋራ ማቀድና መምራት በመሳሰሉት

ኮቪድ-19 በዓለም አቀፍ በሀገራችንና እንዲሁም ደግሞ በክልላችን እንደሚከሰት ከተገመተ ወዲህ በርካታ ሥራዎችን አቅደን እየሰራን እንዳለ ይታወቃል፡፡ በዚህም ሂደት በክልል ደረጃ ግብረ ሀይል በማቋቋም ይህንን በሽታ እንዴት መከላከልና መቆጣጥር እንደምንችል በማቀድ ወደስራ ገብተናል፡፡ በዚህ ግብረ ሀይል ውስጥ ከተካተቱት ተቋማት መካከል እንዱ የኛ (የእርሻና ተፈጥሮ ሀብት ልማት ቢሮ) ተቋም ነው፡፡ ከዚህ ጋር ተያይዞ ታሳቢ ያደረግነው በኮቪድ ምክንያት ለምግብ እጥረት የሚጋለጡ እና የአልባሳት እጥረት የሚያጋጥማቸው እንደሚኖሩ ነው፡፡ ሌላው የኮቪድ-19 በሽታ እየጨመረ ሲመጣ በግብርና ምርት ሂደትና ውጤት ላይ ጫና ያሳድራል፡፡ ይህም የሚገለጸው የግብርና ግብዓት ተደራሽ በማድረግ ሂደት ተፅዕኖ ያሳድራል፡፡ ሁለተኛው የሰው ሀይል ስምሪት ላይ እስከአርሶአደር ድረስ በሚፈለገው ልክ በማሰማራት ላይ አሉታዊ ተፅዕኖ ይኖራል፡፡ እነዚህን ሁሉ ለመቋቋም እንደቢሮአችንም እንደግብረሀይል ከባለድርሻ አካላት ጋር በመቀናጀት እየተሰራ ነው ያለው፡፡ አንዱ በክልላችን ከኮቪድ -19 ጋር ተያይዞ የምግብ እጥረት ለሚያጋጥማቸው በክልላችን አቅምና ከፌዴራል መንግስት በሚደረግ ድጋፍ እነዚህን የህብረተሰብ ክፍሎችን በመለየት የምግብ ድጋፍ ተደርገዋል፡፡ እንዲሁም በጎዳና ላይ ያሉትን ልጆች በማንሳት መጠለያና ምግብ እንዲያገኙ የመድረግ ስራ በተጨማሪ የክልላችን ህዝቦች ሥራ ወዳድ እንደመሆናቸው በተለያዩ የሀገራችን ክፍሎች ተበታትነው ያሉት አሁን በሽታው በፈጠረው ጫና ወደክልላችን ሲመለሱ ለነርሱም ምግብ መጠለያና አልባሳት ማቅረብ ስለሚያስፈልግ ከተለያዩ አጋሮች ያገኘናቸውን አልባሳትንም ድጋፍ አድርገናል ማለት ነው፡፡ ስለዚህ የምግብና አልባሳት አቅርቦትን ለሁሉም ሳይሆን እጅግ በጣም ተጋላጭ ለሆኑት ለይተን የማቅረብ ስራ ሰርተናል፡፡ የአካል ጉዳተኞችም በዚህ ማዕቀፍ እንዲደገፉ አድርገናል፤ ከዞኖች የምግብ እጥረት ድጋፍ በቀረበውና አቅማችን በፈቀደው ልክ ድጋፍ አድርገናል፡፡

ክልሉግብረ ሀይል አቋቁሞ ወደሥራ እንዲገባ የገፋፋው ወይንም ምክንያት የሆነው ነገር ምንድነው? መነሻ የሆነ ሀሳብ በተለያዩ ሰነዶች የተደገፈበትም ካለ? የበሽታውን ጫና የተረዳችሁበትስ አግባብስ ምን ይመስላል?

የኮቪድ -19 ክስተቱ ለክልላችን አስደኝጋጭ ሁኔታ ነው፤ ስለዚህ የመጀመሪያ መነሻ የሆነው ስለበሽታው ዓለምም መንስኤውና መከላከያውን በሚገባ ያላወቀው መሆኑ ነው፡፡በመሆኑም ይህንን ችግር እንደቀድሞ በአንድ ተቋም ጥረት ብቻ ምላሽ ማግኘት እንደማይቻልና ይልቁንም የሁሉንም ባለድርሻ አካላት ርብርብ እንደሚያስፈልግ ነው ግንዛቤ የወሰድነው፡፡ መንስኤው ችግሩ አዲስ ክስተት በመሆኑ ለጊዜው ምንም ዓይነት የሚያድን መድሃኒትም ሆነ የመከላከያ ክትባት ያልተገኘ በመሆኑ ምላሹ ደግሞ የችግሩን ስፋት፣ ግዝፈትና አጣዳፊነትን ያገናዘበና ምልዓተ ህዝብን ያሳተፈ መሆን እንዳለበት በተወሰደው ግንዛቤ ነው፡፡ ሁለተኛው የዓለም ጤና ድርጅት በሽታው ዓለም አቀፍ ወረርሽን መሆኑን በማወጁና በሽታውን ለመከላከል የሚያስችሉትን የተለያዩ አዳዲስ አሰራሮችን በጥናት አስደግፎ የሚያቀርባቸውን በማየት ለክልላችን በምን መልኩ እንጠቀማለን በማለት በጋራ በመወሰን መመሪያዎችንም እያወጣን መሄድ ያስፈልግ ነበር፤ ለዚሁም ለዚህ ችግር ይበልጥ ተጋላጭ የሚሆኑት አጥቢ እናቶች ድንገት በኮሮና ባይረስ ቢያዙ አስቸጋሪ ሁኔታ ሊገጥማቸው የሚችሉ አስቀድሞ እንደ አስም በሽታ እና ሌሎች በሽታዎች ያላቸውና በዕድሜ የገፉት ሰራተኞች ዕረፍት ወስደው እቤታቸው እንዲቆዩ ውሳኔ የተወሰነው በግብረ ሀይል ነው፡፡ ይህና የመሳሰሉት አሰራርን የሚደግፉ ሃሳቦች ማመንጨት ብቻ ሳይሆን በተግባረ እንዲውሉም ጭምር በግብረ ሀይል ተከናውነዋል፡፡እነዚህን ስራዎች የሰራነው በፌዴራል ደረጃ የሚወጣው አዋጅ ከወጣ ነው፡፡ ከዚያ በኋላ የፌዴራል አስቸኳይ ጊዜ አዋጅ ወጥቷል፡፡ ከዚህ ጋር ተያይዞ የተለያዩ መመሪያዎች መጥተዋል፡፡ይህ ከወጣ በኋላ እነዚህን መመሪያዎችንና ደንቦችን ተከታትሎ የማስፈጸምና ክፍተቶች ሲታዩ የማረም ስራዎች ስከናወኑ ነው የቆየው፡፡

እርሻና ተፈጥሮ ሀብት ልማት ቢሮ ይህንን ሥራ (የኮቪድ-19 መከላከልና ቁጥጥር) በሚያከናውንበት ጊዜ ከተለያዩ የራሳችሁ ተጠሪ ተቋማት ከፌዴራል ሚ/ር መስሪያ ቤትና ከፌዴራል የአደጋ ስጋት ሥራ አመራር ኮሚሽን ጋር ያላችሁ የስራ ቅንጅትና ቁርኝት ምን ይመስላል?

እንግዲህ ቅንጅታዊ ሥራ ከላይ እስከታች ድረስ በአንጻራዊነት ጥሩ ነው ልባል ይችላል፡፡ በተለይ እንደቢሮአችን ስንወስድ ቅድም ያልነው የምግብ አቅርቦትን በሚመለከት በክልላችን አቅም ብቻ የምናሟላው አይደለም፡፡ የኢፌዴሪ አደጋ ስጋት ስራ አመራር ድጋፍ የሚያስፈልግ ነው፡፡ የተለያዩ የምግብና የአልባሳትን ፍላጎታችን ለአደጋ ስጋት ስራ አመራር እያቀረብን እነርሱም ባላቸው አቅም ልክ እያገዙን በጋራ እየሰራን እንገኛለን፡፡ ሌላውና አንዱ ትልቁ ተፅዕኖ በግብርና ሥርዓታችን የሚያሳድረው ነው፡፡ ይህም እንደ ሀገርም እንደ ክልላችን በሎጅስትክስ አቅርቦት ላይ የሚያሳድረው ነው፡፡ በግብዓት እንደማዳበሪያ ዘር እና የተለያዩ የሰብል በሽታና ተባዮች መከላከያ ኬሚካሎች አቅርቦት ላይ ከፍተኛ ተጽእኖ ነው እያስከተለ ያለው፡፡ ለምሳሌ ለአብነት ብንወስድ ግብዓትን ለማቅረብ መጫን ማውረድ ያስፈልጋል፡፡ እነዚህ በመጫንና በማውረድ ሥራ ላይ የተሰማሩት ለኮቪድ የበለጠ ተጋላጭ የመሆን ሁኔታ ስለሚያጋጥማቸው በአቅርቦት ላይ አሉታዊ ተፅዕኖ አለው፡፡ ለምሳሌ ማዳበሪያ በጂቡቲ ወደብ ላይ ለማራገፍና ለመጫን የሰው ሀይል እጥረት አጋጥሞን ያውቃል፡፡ በኮቪድ በሽታ የተያዙም አሉ፤ ይህ በሌሎች ደግሞ የሚፈጥረው ድንጋጤ ክፍተኛ ነው፡፡ ይህም ላጭር ጊዜ አሉታዊ ተጽዕኖ ፈጥሮ ነበር ግን ከግብርና ሚኒስቴር ጋር በመነጋገር አማራጮችን በመፈለግ እንዴት መከላከል አለባቸው ፤ ተጽዕኖ ከፋ እንዳሆን መደረግ ስላለበት ከሚ/ር መስሪያ ቤት ጋር በመነጋገር ከወደብ ጀምሮ ማዕከላዊ መጋዘን በየፈዴረሽኖች ያሉትን መጋዘኖች እስከና እና እስከታች ድረስ ባሉት የማጓጓዝ መጫንና ማወረድ ሥራን በጋራ በመመካከር ችግሮችን በመፍታት በተሳለጠ ሁኔታ እየሰራን እንገኛለን፡፡ በዘር አቅርቦትም ተመሳሳይ ሁኔታ ነው ያጋጠመን፤ የዘር አቅርቦት የምናገኘው ከክልላችንና ከፌዴራል አቅራብ ተቋማት ነው፡፡ እነዚህንም ጭምር በተቀናጀ ሁኔታ እየሰራን ነው ያለነው፡፡ ሌላይኛው የሰራተኛ ነው፡፡ የልማት ጣቢያ ሰራተኞቻችን አርሶአደርን በአካል በማግኘት የማማከርና የመደገፍ ሥራ መሥራት ይጠበቅባቸዋል፡፡ ከዚህ በፊት ብዙ አርሶአደሮችን ባንድ ጊዜ የማግኘት ዕድልም ነበራቸው፤ እንደዚሁም ደግሞ ቤት ለቤት እየሄዱ፡፡ አሁን ግን ብዙ አርሶአደሮችን ባንድ ጊዜ ሰብስቦ ማግኘት አይችሉም፡፡ ስለዚህ ማድረግ የሚችሉት ቤት ለቤት በመሄድ ማነጋገር፤ ለዚሁም ደግሞ ራሳቸውን ጠብቆ መሆን ስላለበት ለዚህም ራሳቸውን እንዲከላከሉ የግል መከላከያ ቁሳቁሶችን እንደ የፊት መሸፈኛ ጭንብል፣ ሳኒታይዘርና የእጅ መታጠቢያ ሳሙና በክልላችን ላሉት ከሃያ ሺህ በላይ ለሚሆኑት አቅርበናል፡፡ ከታች አርሶአደሩን ከሚያገኙት ጀምሮ በሽታውን የሚከላከሉበትን መንገድን እንዲገነዘቡና የመከላከያ ቁሳቁሶችን እንዲያገኙ አድርገናል፡፡

ከዚህ በላይ በግብርና ግብዓት አቅርቦትና በሰው ሀይል ስምሪት በሚፈጠረው ተፅዕኖ ምክንያት የምርት ጉድለት በክልላችንም ሆነ በሀገር አቀፍ ደረጃ ይኖራል የሚል ግምት ይዘናል፡፡ በመሆኑም ለኮቪድ ምላሽ ዝግጅት በግብርና ሚ/ር ደረጃ ተዘጋጅቷል፡፡ እኛም በዚሁ ላይ ተስማምተናል፤ ሀገራዊ መድረክ ተፈጥሮ ጠቅላይ ሚ/ር በሰበሰቡት መድረክ እኛም ተገኝተን ድርሻችን ለመወጣት ተስማምተን መጥተናል፡፡ ከዚህ ተመልሰን እንደክልላችን የግብርና ኮቪድ ምላሽ ዕቅድ አዘጋጅተናል፡፡ ዝርዝር ዕቅድ አዘጋጅተን የክልሉ ምክትል ርዕሰመስተዳድር ባሉበት ለሁሉም ዘርፋችን መዋቅር ማለትም የዞን እርሻና ተፈጥሮ ሀብት ልማት መምሪያ የዞን ግብረ ሀይል ሰብሳቢ (የዞኑ ዋና አስተዳዳሪ) ሆነው በቪዲዮ ኮንፈራንስ በዝርዝር ዕቅድ ዙሪያ ሰፊ ውይይት በማድረግ የጋራ መግባባት ፈጥረናል፡፡ የዚህ ዕቅድ ቀጣይ በዞን በወረዳና ቀበሌ ደራጃ ተመሳሳይ መድረክ ተዘጋጅተው በመወያየት የጋራ መግባባት ተደርሰዋል፡፡ ማለትም ትስስሩ ከላይ ከጠቅላይ ሚኒስትር ና ሚኒስቴር መስሪያ ቤት ጀምሮ እስከቀበሌ ድረስ ተፈጥረዋል፡፡ ይህም የምርት ጉድለት በበሽታው ምክንያት እንዳፈጠር እንዴት ሊካካስ እንደሚቻል ዝረዝር ዕቅድ የተዘጋጀና ወደ ትግበራም የተገባበት ሁኔታ ነው ያለው፡፡ ስለዚህ ትስስሩ ይህን ይመስላል፡፡

የኮቪድን መከላከል ሥራ እንደቢሮ የሚከታተል በተለይ የተመደበ ሰራተኛ ስለመኖር ወይንም በቢሮ ሀላፊ ስለመመራት ጉዳይ፡

በዚህ ግቢና በዚህ ህንጻ ውስጥ ሁለት ቢሮዎችና በስሩ ተጠሪ የሆኑት ተቋማት ይገኛሉ፡፡ አዚህ ያሉት ሰራተኞችም ራሳቸውን ከኮቪድ መከላከል ይገባል፡፡ ሰራተኛው ራሱን እየተከላከለ ነው አርሶአደርን መደገፍ የሚችለው፤ስለዚህ ያንን የሚመራ ግብረ ሀይል ተቋቋሟል፤ እንደቢሮአችን ያ ግብረሀይል የበሽታ መከላከያ ሥርዓት ባግባቡ መከናወንን ይከታተላል፤ ለምሳሌ እዚህ ግቢ ማንም ማስክ ሳያደርግ መግባት እንደማይፈቀድ ማስታወቂያ በየቦታው እንደተለጠፈ ስትገቡ አይታችሁ ይሆናል፡ ይህ ተግባራዊ እንዲሆን በመስሪያ ቤት በኩል መቅረብ ያለባቸውን እንዲቀርብ ማድረግ መስሪያ ቤቱ እንከዋን ባይችል እያንዳንዱ ሰራተኛው በራሱ ገዝቶ እንዲያደርግ ከስምምነት ተደርሰዋል፡፡ እዚህ ላይ ጉድለት ካለ ይህ ግብረሀይል ይገመግማል ማለት ነው፡፡ ሁለተኛ ሳኒታይዘር ና የእጅ መታጠቢያ ሳሙና ማቅረብና ሰው እጁን ታጥቦ ስለመግባት ቁጥጥርና ክትትል ማድረግ የዚህ ግብረ ሀይልና በሥሩ የተቋቋሙት ንዑሳን ግብረ ሐይሎች ሃላፊነት ነው፡፡ ይህ ግብረ ሀይል ከሁለቱም ቢሮዎች ጋር በመመካከር ነው የሚሰራው፡፡

ይህ ሂደት በርካታ ወጪ የሚጠይቅ እንደሆነ ይታወቃልና ሀብት ከየት አገኛችሁ/ እንዴት እየተወጣችሁ ነው?

ከሀብት አንጻር እንደክልላችን በዚህ ዓመት የሀብት እጥረትም አለብን፤ባለፉት ሁለት ሶስት ዓመታት ከገጠሙን ክልላዊ ክስተቶች አንጻር በዚህ ዓመት ከሀብት አንጻር ትልቅ አሉታዊ ተጽዕኖ አሳድረዋል፡፡ ሆኖም ያለን ውስን ሀብትም ቢሆን ለሰው ልጅ ደህንነት ነው ቅድሚያ የሚሰጠው፤ መሆን ያለበትም፡፡ ከዚህ አንጻር የክልሉ ዐቢይ ግብረሀይል ለኮቪድ ምላሽ የሚሆን ሀብት ራሱን ችሎ ወደ ሰባ ሚሊየን የሚሆንን መድበዋል፡፡ ይህ እንግዲህ ከመጠባበቂያ አይደለም፤ የካፒታል ፕሮጄክቶችን ሥራም እንዲቆም ተደርጎ እና ሁለተኛ ደግሞ ከስልጠና በጀት ሙሉ በሙሉ ሳናቆም በፐርሰንት በመቀነስና ቅድሚያ የሚሰጣቸውን በመለየት አንዳንድ ስራዎችን ለሚቀጥለው ዓመት በማስተላለፍ ገንዘቡን ወደመጠባበቂያ በማዛወር ለከቮድ በመመደብ ነው፡፡

ወደቢሮአችን ስንመጣ ደግሞ የግል መከላከያ ቁሳቁሶችን ግዥ አንዳንድ ስራዎችን ለሚቀጥለው ዓመት በማሸጋገርና ከአንዳንዶች ደግሞ በመቀነስ በጀት በማዛወር ለኮቪድ ምላሽ በማዞር ነው፡፡ ሁለተኛ ደግሞ ከቢሮአችን ጋር በአጋሪነት ከሚሰሩት ተቋማትና አንዳንድ ፕሮጄክቶችና ፕሮግራሞች ለስልጠናና ግዥ ከሚሰጡን ድጋፎች ወደዚህ በማዞር ነው እየተወጣን ያለነው፡፡ ሌላው መንግስታዊ ያልሆኑትን ድርጅቶችን በማስተባበር ባጠቃላይ እስከ አስር ሚሊየን ደረስ ብር ወጪ አድርገን ነው የግል መከላከያ ቁሳቁሶች ግዥ ያካህድነው፡፡ ይህ አስር ሚሊየን እንደእርሻና ተፈጥሮ ሀብት ቢሮ ስሆን የእንስሳትና ዓሳ ሀብት ቢሮ ሲጨመር ሀብቱም ይጨምራል፡፡

ቅንጅታዊ ስራን ውጤታማ ለማድረግ እንደዐቢይ ግብረ ሀይልም ይሁን እንደ ንዑስ ግብረ ሐይል የክትትልና ድጋፍ እንዲሁም የግምገማ ስርዓት ምን ይመስላል?

ሀብት ማሳባሰብ የአንዱ ንዑስ ኮሚቴ ሥራ ነው፡፡ ለዚህም ክልላዊ ዕቅድ ተዘጋጅቶ ነው ወደሥራ የተገባው፡፡ ከዚህም ደግሞ ወደ ዞኖች ተሸንሽኖ የወረደው፤ ስለዚህ ሀብት በዞንና በልዩ ወረዳዎችም ጭምር በካሽና በዓይነት እንዲሰበሰብ የተደረገው፡፡ ሀብትን በማሰባሰብ አኳያ ከመዋቅር መዋቅር ወይንም ከዞን ወደ ዞን እንዲሁም ከልዩ ወረዳ ወደ ወረዳ ሥራውን ይዞ እየገመገሙ ከመምራት አንጻር እንዲሁም ህብረተሰቡን ባለቤት ከማድረግ አንጻር ሁሉንም መዋቅሮችን እኩል ያለማነቃነቅ ክፍተቶች ይታያሉ፡፡ ከዚያ ባለፈ ግን አንጻራዊ ንቅናቄ ጥሩ ነው፡፡ ከዚህ ውጭ ትኩረት የሚሻው ግን የተሰበሰበው ለታለመለት ዓላማ እየዋለ ነው የሚለውን ማረጋገጥ ይጠይቃል፡፡ እንግዲህ ሀብት በዓይነትም ይሁን በካሽ የተሰበሰበው በዞንና ልዩ ወረዳ ደረጃ ስለሆነ ይህንን ባግባቡ ሥራ ላይ አውለዋል ወይ የሚለውን በድፍረት ለመናገር በቅርበት ክትትል ማድረግ ያስፈልጋል፤ በዚህ ረገድ ክፍተት ያለ ይመስላል፡፡ በርግጥ ግምገማ ይደረጋል ግን በቂ አይደልም የሚል እምነት አለኝ፡፡

ይህንን ሥራ ለመገምገም በምን ያህል ጊዜ ውስጥ ትገናኙ ነበር? እንዲሁም በጋራ የታቀደው ሥራ ባይተገበር ተጠያቂነትን የማስከተል አግባብ ምን ይመስል ነበር?

በጅምር ሁኔታው አዲስና አስደንጋጭም ስለነበር በሳምንት ሶስት ጊዜ ነበር ፤ ተገቢም ነበር፤ በሂደት ወደ ሁለት ቀን በሳምንት እንገናኝ ነበር፤፤ እየቆዬ ሲመጣ መንጠባጠብ ታይተዋል፡፡ ያ ማለት ግን ትኩረት አለመስጠት ማለት አይቻልም ግን ሥራው ይበልጥ ተቋማዊ እየሆነ መምጣቱን በማየት የተደረገ ነበር ማለት ይቻላል፡፡ ከተጠያቂነት አንጻር ጉደለቱ ከፍተኛ ሆኖ ተጠያቂነትን የሚያስከትል ጉድለት በበኩሌ አላስተዋልኩትም፡፡ ምናልባት በዚያ ልክ አልሰራንም እንደሆነ አላወቅኩም፡፡ ይሁን እንጂ ጉድለት እንከዋን ቢታይም ተከታትሎ ተጠያቂ ከማድረግ አንጻር የተቀመጠ ግልጽ የሆነ ነገር የለም እውነቱን ለመናገር፡፡ ትኩረት ይፈልጋል በሚለው ቢወሰድ የተሻለ ይመስለኛል፡፡

ኮቪድን ለመከላከልና ለመቆጣጠር በምናደርገው ሂደት እንደመልካም አጋጣምና ተሞክሮ ሊጠቀስ የሚችል ሁኔታዎች ወይንም ክስተቶች ካሉ?

ሁሌም ተግዳሮት ወይንም ቻሌንጂ ሲመጣ ሌላ መልካም አጋጣምም ይዞ ይመጣል፤ ይህም ይህንን ቻሌንጂ ለመጋፈጥ የምንሄድባቸው መንገዶች አዳዲስ አቅሞችን እየፈጠሩልን ነው የሚሄዱት፡፡ እንግዲህ ይህንን ኮቪድ-19ን ከምንከላከልባቸው መንገዶች አንዱ ንጽህና መጠበቅ ነው፤አስቀድሞ ሰው በማወቅም ይሁን ባለማወቅ የእጅ ንጽህና የመጠበቅ ባህል አነስተኛ ነበር አንደ ሰፊ ማህበረሰብ ካየነው፤ ሆኖም ግን ይህ ችግር ከተከሰተ ጊዜ አንስቶ የእጅ መታጠብ ጉዳይ ሰፊ ቦታ ተሰጥቶ መመሪያም ተዘጋጅቶ ተከታታይ የግንዛቤ ማስጨበጫ መልዕክቶች መተላለፍ ከጀመረ ወዲህ ሰው የግል ንጽህና መጠበቅ ባህል ጨመሯል፡፡ በዚህም ምንም እንከዋን በጥናት የተረጋገጠ ባይሆንም በንጽህና ጉድለት የሚከሰቱ በሽታች እንደቀነሱ እንሰማለን፤ ይቀንሳል ብለንም እንገምታለን፤ ሳይንሳዊም ነው፡፡ ኮቪድ እንከዋን ቢጠፋም ይህን እጅ በተከታታይ መታጠብን ህብረተሰቡ ባህል ያደርጋል የሚል እሳቤ አለኝ ፤ ይህም አንዱ መልካም አጋጣሚ ነው እላለሁ፡፡ ሌላኛው ጥሩ ዕድል ነው ብለን የምንወስደው ከቴክኖሎጂ አጠቃቀም ጋር የተያያዘ ነው፡- ለምሳሌ ኢትዮጵያ ውስጥ ኢንቴርነት ከገባ ረጅም ጊዜ ያስቆጠረ ቢሆንም ይህንን ባግባቡ የምንጠቀም እጅግ ውስን ነበር፤ ምናልባት ፍላጎት ከማጣት ይሁን ካለመቻል አንጻር ግልጽ ባልሆነ ሁኔታ ማለት ነው፡፡ አሁን ከኮቪድ-19 የተነሳ በአንድ አዳራሽ ውስጥ ብዙ ሆነን መሰብሰብ አለመቻላችን ግዴታም ስለሆነ በቪዲዮ ኮንፈራንስ ትላልቅ ስብሰባዎችን እያደረግን መጥተናል፡፡ በዚህም በቴክኖሎጂ አጠቃቀም አንድ እርምጃ ወደፊት ሄደናል ማለት ነው፡፡ ስለዚህ ከቴክኖሎጂ ጋር እንዲንተዋወቅና ቴክኖሎጂን ወደፊት የተቋማችን የዕለት ተዕለት ስራ አካል እንዲናደርግ ዕውቀትና ክህሎትን በማግኘት የተቋማችን የአሰራር አካል እንዲሆን ዕድል የፈጠረ ነው ማለት ይቻላል፡፡ ይህ ደግሞ ሀብትን በቁጠባ እንዲንጠቀምና አንዳንድ አላስፈላጊ ወጪዎችን እንድንከላከልም ዕድል ፈጥሮልናል፤ ለምሳሌ ለአንዳንድ ስብሰባዎች ዞኖችንና ወረዳዎችን በመጥራት ብዙ ሚሊየን ብር የምናወጣበትን ስብሰባዎችን በቪዲዮ ኮንፈራንስ በማድረግ ወጪ እንድንቀንስ አድርጎናል፡፡ ሌላው ሀብት ሲያጥረን አስፈላጊ ፊት ለፊት እናደርግ የነበረውን ስብሰባን እንሰርዝ ነበር፤በዚህም ያቀድነውን ሳናሳካ እንተው ነበር፤ይህም በክትትልና ድጋፍ ሂደትም ሰፊ ክፍተት ፈጥሮብን ነበር፡ አሁን ግን ሀብት ሳናባክን ከዞኖችና ልዩ ወረዳዎች ጋር ባስፈለገን ጊዜ በዙምና ቪዲዮ ኮንፈራንስ ስብሰባ እናደርጋልን፡ መረጃ እንለዋወጣለን ማለት ነው፡፡ በዚህም ስራዎቻችን እየገመገምን ነው፤ ይህ ደግሞ አንድ መልካም አጋጣም ነው፡፡ ስለዚህ ሀብትን እንደንቆጥብ ከማድረግም በተጨማሪ ሥራችን በተቀላጠፈ ሁኔታ እንድናከናውን አማራጭ ሆኖናል፡፡ ሌላው ደግሞ የቴክኖሎጂ ማመንጨት ዕድል ነው፡፡ ሰው ወደፈጠራ የገባበት ሁኔታ አለ፡፡ ለአብነት ከንክኪ ነፃ የሆነ የእጅ መታጠቢያ ማሽን ተሰርቶ መቅረቡ አንዱ ማሳያ ነው፡፡ የተለያዩ የህክምና መገልገያ ቁሳቁሶችን እና መድሃኒትን ጭምር ለማምረት የሚደረገው ጥረት የዚህ ውጤት ነው፡፡ ፈጠራን እያበረታታ ነው ማለት ይቻላል፡፡

ቁልፍ መረጃ ሰጭ አስር

የደቡብ ሬዲዮና ቴሌቭዥን ድርጅት

የናንተ ድርጅት የኮቪድ-19 መከላከልና ቁጥጥር ሂደት ያላችው አጠቃላይ ቁርኝትና አስተዋጽኦ ምን ይመስላል?

- በማሰተባበር ረገድ ከሌሎች ጋር በአጋርነት ከመስራት አኳያ ተቋማችሁን ለተልዕኮ ብቁ አድርጎ በማዘጋጀት በጋራ ማቀድና መምራት በመሳሰሉት ረገድ፡-

ኮቪድ-19 በሽታ በዓለም መከሰቱ ከተሰማና በሀገራችንም እንደሚከሰት ግንዛቤ ከተወሰደ ጊዜ አንስቶ ከሀገር አቀፍ ጀምሮ እኛም እንደ ተቋም የኮሚቴ አባል ሆነን ከፌዴራል እስከታች ድረስ የሚዲያ ኮሚቴ አለ፤ በዚያ የሚዲያ ኮሚቴ ውስጥ የሚዲያና ቅስቄሳ እና የህዝብ ግኑኝነት ሥራ ጭምር ይዞ የሚሰራ አንድ ክንፍ ላይ ተደራጅተን ነው የምንቀሳቀሰው፡፡ በክልል ደረጃ በተቋቋመው ዋና/ዐቢይ ግብረ ሀይል አባል ሆነን ነው የምንቀሳቀሰው፡፡ እኛ ሥራው ከመጀምሪያ ጀምሮ ሳይቋረጥ የሚሄድ አሰራር ያለው ሆኖ ነው እየተንቀሳቀስን ያለው፤ ጠለቅ ያለውን ቀጥሎ ነው የሚገልጽላቸው፡፡ አጠቃላይ ሁኔታውን በሁለት ከፍዬ ሊግለጽላችው፤ አንደኛው ከፌዴራል ጀምሮ ያለው የሚዲያ ኮሚቴ በፌዴራል ደረጃ በጠቅላይ ሚኒስቴር ጽ/ቤት የፕረስ ሴክሪታሪ የሚመራ በሀገሪቱ ያሉት ሁሉም የመንግስትና ህዝብ ሚዲያዎች የምገናኙበት፤ ሁሉም ስል እነዚህ ዘጠኝ ማለትም በክልል ያሉትና በፌዴራል ደረጃ ያሉት ትላልቆች እንደ ኢብሲ፣ ዋልታ፣ ፋናና ኢዜአ ፕረስ የጨመረ እነዚህ በጋራ የምንገናኝበትና እነዚህ እየተገናኙ ሪፖርት የሚለዋወጡበትና ሥራው እየተገመገመ የሚሄድበት መስመር ነው፡፡ እዚያ ላይ በዋናነት እየሰራን ያለው ምንድነው፡- በሀገሪቱ ይህንን የመከላከል ሥራ እየሰራ ባለው አካል በአስተምሮህና መከላከል ረገድ መሰረታዊ አቅጣጫ ቢሆኑ በሚባሉ ጉዳዮች የተለያየና የተንጠባጠበ እንዳይሆንና ወጥ የሆነ የሚዲያና ኮሙኒኬሽን ሥራ መስራት እንዲቻል ነው ይህ አደረጃጀት የተፈጠረው፡፡ ይህ በቋሚነት እየተገመገም እኛም ሪፖርት እናቀርባለን፤ ይህም በሳምንት እየተገመገመ የሚሄድ ነው፡፡ ይህ ሂደት ግብረመልስም ያለው ሆኖ በተፈጠረው ግሩፕ በየቀኑ መረጃና ግብረ መልስ የምንለዋወጥበት ነው፡፡ ሌሎች ከኮቪድ ጋር በተያያዘ አዳዲስ ነገሮች የሀገር ውስጥም ይሁን የዓለም አቀፍ መረጃ ሁሉም የሚጠቀሙት፡ በደንብ ጎልቶ መውጣት ያለባቸው ካሉ ሁሉም መስራት ያለባቸው በማድረግ እርሱ ላይ በመንተራስ የሚሰራ ሥራ አለ፡፡

በኛ በኩልም በሀገሪቱ ወጥ በሆነ ሁኔታ እንዲሰራ በተባለው አግባብ እየሰራን እንገኛለን፡፡በክልል ደረጃ የዋናው ግብረ-ሀይል አባል ነን፤ ከዚያ ቀጥሎ ደግሞ የኛ የቴክኒክ ኮሚቴ አለ፤ያ የቴክኒክ ኮሚቴ ማለት የሚዲያ ኤዲቶሪያል ነው፡፡ እኔን ጨምሮ የይዘት ሀላፊዎችና ከታች ያሉ ዳይሬክተሮች አሉ፤ አጀንዳ አዘጋጅተው የሚሰራ አካል ነው የቴክኒክ ኮሚቴ፤ በዚህ አሰራር እኛ በዋናነት በሁለት ነገሮች ላይ ነው ትኩረት ሰጥተን የምንሰራው፤ አንደኛው የማስተማር ሥራ ነው፤ የማስተማር ሥራ በመሠረታዊነት በሁለት ተከፍሎ ነው የምንሰራው፤ አንደኛው ኮቪድ ከተከሰተ ጊዜ ጀምሮ ምን ማለት ነው እሱ? እንዴት መከላከል ይቻላል? ምን ዓይነት ባህሪይ አለው? ሰው ምን ዓይነት ጥንቃቄ ያድርግ? ወዘተ. የሚሉትን በስፋት የማስተማር ሥራ ነው፡፡ ይህን የማስተማር ሥራ በማዕከል ብቻ ሳይሆን በዐቢይ ኮሚቴ ከተወያየን በኋላ በቴክኒክ ኮሚቴ ደረጃ የክልሉን የመንግስት ኮሙኒኬሽን ቢሮ ጨምሮ ሳይንስና ኤንፎርሜሽን ቢሮ ያለበት የተወሰኑት ሌሎች ቢሮዎችም ያሉበት የዚህ ኮሚቴ አለ፤ ከዚያ ኮሚቴ ውስጥ ነው ራሳችንን የቴክኒክ ኮሚቴ አድርገን ወስደን ትኩረት አድርገን የምንስራው ማለት ነው፡፡

ስለዚህ ይህ የቴክኒክ ኮሚቴ ደግሞ አስር ቅርንጫፎች አሉት፡፡ ወደዚህ ቅርንጫፎች ዋንኛ መልእክቶች እንዲወርዱ እናደርጋለን፡፡በኤፍ ኤም ጣቢያዎች በ48 ቋንቋዎች ተተርጉሞ መልእክት እስከታች ህብረተሰብ ድረስ እንዲደርስ እናደርጋለን፡፡ የማስተማር ስራ በዋናነት የሚያተኩረው በመከላከል ላይ ነው፡፡ ባህሪይውን በማሳወቅና እንዴት መከላከል እንዳለበት የሚያደርግ እጅግ በርካታ ስራዎችን ሰርተናል፡፡ በጣም ስፋት ያለውን ሥራ ሰርተናል፤ ይህ ስፋት ያለው ሥራ የተቋሙን መደበኛ ዕቅድ እስከሚያንገዳግድ ድረስ ነው የገባንበት፤ እኛ ብዙ በመንግስት የምንደገፍ ቢሆንም የውስጥ ገቢ አሰባስበን የምንጠቀምበት ዕቅድ አለ፡- ከሰላሳ ሚሊየን ብር በላይ የመሰብሰብ ግዴታ አለብን፤ ያ ብር ደግሞ የተቋሙ በጀት አካል ሆኖ የተመደበ ነው፡፡ ሆኖም ግን በጣም በርከት ያሉ ሥራዎችን በነጻ ነው የምንስራው፤ ይህ የማህበራዊ ሀላፊነትን የምንወጣበት ግዴታም ስለሆነ ገንዘብ ካላመጣችሁ ብሎ ሥራ ማቆም አይቻልም፡፡ እንዲሁም አልፈን መከላከልን መሠረት ያደረገ የማስተማር ሥራ በስፋት አከናውነናል፡፡ በማዕከል ካሉት ቢሮዎች ጋር በተቀናጀ መንገድ የማስተማር ሥራ አከናውነናል፡፡ በማስተማር ሥራ ሂደት ተቀናጅተው ከሚሰሩት ቢሮዎች በዋናነት የጤና ቢሮ ስሆን እነርሱም በዋናነት ትክክለኛ መረጃ በመስጠት ለምሳሌ በጤና ቢሮ ሀላፊና በህብረተሰብ ጤና ኢንስቲትዩት ዳይሬክተር ውጭ በበሽታ የተያዙትን የመግለጽ ሀላፊነት እንደሌለ ብቻ ሳይሆን ክልክል ነው፤ ይህም በወጣው መመሪያ መሰረት ስለሆነ መረጃን በምንፈልግበት ጊዜ የጤና ቢሮ ሃላፊ ከየትም ቢሆን በስልክ ጭምር ገብቶ መረጃ ይሰጣል፡፡ እዚሁ ከሆነ ደግሞ ስቱዲዮ ይገባና መረጃ ይሰጣል፡፡ አቶ አቅናው ማለት ነው፤ እርሱ ከሌለ ደግሞ ዶ/ር ማሙሽ ይሰጣል፡፡ መግለጫ ብቻ ሳይሆን እኛጋ መደበኛ ፕሮግራሞች በስፋት ነበሩ፡፡ ቀጥታ ከህዝብ ጋር የሚደረጉ ውይይቶች ሲኖሩ እነዚህ ሰዎች እየመጡ ለሚነሱ ጥያቄዎች ምላሽ ይሰጣሉ፤ አንዳንድ ውይይቶች እስከሁለት ሰዓት ድረስ የሚቆዩ ናቸው፡፡ በዚህ ሂደት ማብራሪያ ይሰጣሉ፤ ብዥታ ካለም እንዲጠራ ያደርጋሉ፡፡ ስለዚህ ከነርሱ ጋር በቅንጅት ሰርተናል፡፡

ሁለተኛ ሌሎች ሰክተር መስሪያ ቤቶች ለምሳሌ ንግድና የገበያ ልማት ቢሮ ይህ ቢሮ ከገበያ አስተዳደር አቅርቦት ጋር ሰፊ ችግሮች ስለነበሩበት ከኛ ጋር ተቀናጅተው ሰርተዋል፤በራሳቸው የሚያዘጋጃቸውን መልእክቶችንም ጭምር ያለክፍያ እንስተላልፍላቸው ነበር፤ መዚቃዎች መነባንቦች ህዝቡ እንዲጠነቀቅ የሚያደርጉ የተለያዩ መልእክቶችን ፤ በተመሳሳይ ባህልና ቱሪዝም ቢሮ ሴቶችና ህጻናት ቢሮ ርዕሰ መስተዳድር እንዲሁም፡፡ ርዕሰ መስተዳድር ለምሳሌ ሀገር ውስጥ ያሉትን የክልሉ ተወላጅ የሆኑትን ታዋቅ ግለሰቦች፣ አርትስቶች፣ አትሌቶች የተለያዩ አካላትን በነርሱ በኩል ተደራሽ አደርጎ እኛ ቀረጻውን በመከናወን መልዕክት እንዲያስተላልፉ አድርገናል፡፡ ከሌሎች ተቋማት ጭምር እነርሱ ወደእኛ የመጡ አሉ፤ እኛ ደግሞ ወደእነርሱ ሄደን የሰራነው አለ፡፡ ከጊዜ አንጻር ይህንን ሥራ በየቀኑ የምንሰራው ስለሆነ ስፋት ያለው ነው፡፡

የክልል ዐቢይ ኮሚቴ እና የፌዴራል ቴክኒክ ኮሚቴ ጋር በጋራ እንደምትሰሩ ባለን መረጃ መሰረት የሁለቱ እቅዶች የመናበብ ሁኔታ ምን ይመስላል? አንዱ ሌላውን ከመደገፍና ከማገዝ አኳያ ምናልባት የማይናበቡ ሆኖስ ከሆነ እንደት አጣጣማችሁ?

እስካሁን የገለጽኩት አንደኛው ሥራችን ነው፤ ይህም የማስተማር የማንቃት ነው፡፡ ሁለተኛው ደግሞ የማጋለጥ ሥራዎችን ናቸው፡፡ በሽታውን ለመከላከል ይረዳ ዘንድ የአስቸኳይ ጊዜ አዋጅ ወጥተዋል፤ በዚህ አዋጅ በርካታ የበሽታ መተላለፍን ሊያባብሱ የሚችሉ ተግባራትን ክልከላ ያደረጉት ሁኔታዎች ይገኛሉ፡- ሰው በበዛበት ቦታዎች መደረግ የሌባቸውም የተባሉትን ከማድረግ ጀምሮ ቁሳቁሶችን መሰወር ዋጋ መጨመር የመሳሰሉት በርካታ ድርጊቶች ስለነበሩ እነዚህ ላይ በስፋት ሰርተናል፡፡ የማጋለጥ ስራዎችን በስፋት ሰርተናል፤ ከዚህ ጋር ተያይዞ ደግሞ ከፖሊስ ኮሚሽንና የክልሉ ሰላምና ጸጥታ ቢሮ በጣም አግዞናል፤ ተቀናጅተን በርካታ ስራዎችን አከናውነናል፡፡ በነዚህ በሁለቱ ጉዳዮች ሰፋፊ ስራዎችን ሰርተናል፡፡ ዕቅዱ ከፌዴራል የተጀመረ ላይ የራሳችንን አቅደን የምንሰራበት የፌዴራል መንግስት በዋናነት የመከላከሉ ሥራ በሚፈለገው አቅጣጫ ይዘን እንዲንሄድ ነው ያገዘን፡፡ ለምሳሌ አማራ ክልል ሌላ ጉዳይ እየሰራ እኛጋ ደግሞ ሌላ ነገር የሚሰራ ከሆነ ወረርሽኝ ስለሆነ ወጥ የሆነ እልባት አያገኝም፤ከማዕከል ከጤና ሚኒስቴር ከዓለም የጤና ድርጅት የሚወርዱ መረጃዎች ወጥ ሆነው እንዲተገበሩ አግዞናል ማለት ነው፡፡ ይህ ደግሞ መረጃ ብቻ ሳይሆን የተግባቦት አካሄድንም ይጨምራል ማለት ነው፤ ስለዚህ ከዚያ አንጻር ከክልል የሚመጣውና ከፌዴራል የሚመጣ አይጣረዝም፡፡ ክልል ላይ የምኖረው ይበልጥ ሰብሰብ ያለ ይሆናል፡፡ ውስን አካባቢዎች ይህ ዞን ያ ወረዳ ያ የከተማ አስተዳደር እየተባለ እያንዳንዱ አካባቢ እየተያዘ የሚገመገምበት ሁኔታ ነው በክልል ያለው፡፡ ኳራንታይን አካባቢ እየተነሳ ምን ዓይነት የሚዲያ ሽፋን መሰጠት እንደላበት ነው የሚገመገመው፡፡ ከፌዴራል ጋር ያለው ደግሞ እንደሀገር የተያዘው አቅጣጫ የሚሸራረፍ ነገር እንዳኖር የሚያደርግ ስለሆነ አይጣረዝም፡፡ የተናበቡ በመሆናቸው እስካሁን ያጋጠመን ችግር አልነበረም፡፡

የተቋማችሁን ሰራተኞችና ደንበኞቻችሁን ከበሽታው ለመከላከል ምን ዓይነት ዝግጅት አድርጋችኋል?

በሀገራችንም ይሁን በዓለም አቀፍ ደረጃ ለኮቪድ ተጋላጭ ከሆኑት ሰራተኞች አንዱ የኛ ነው፡ እንዲሁም ከጤና ባለሙያዎች ባልተናነሰ ሁኔታ ችግሮች ባሉበት እየገቡ ዘገባ ይሰራሉ፤ መረጃ ተደራሽ ለማድረግ ይሯሯጣሉ፤ ስለዚህ ምንድነው ያደረግነው፡ ከንጽህና አጠባበቅ አንጻር መጀመሪያ ከኛ ተቋም ነው የተጀመረው፡፡ ሰራተኞች ወደተቋም ሲገቡ በር ላይ እጃቸውን ታጥበው እንዲገቡ በማድረግና ይህንንም ለሌሎች በማሳየትና በኮሚቴ ስብሰባዎችም እነዚህን በማንሳት በተቋማት በዚህ መንገድ መሄድ ያዋጣል በሚል ነው የጀመሪነው፡፡ ከዚያ በኋላ ሀኛ የተሸለ የሰሩት ብዙ ተቋማት አሉ፡፡ ስለዚህ በኛ በኩል ይህን ለማድረግ ሞክረናል፤ ለሰራተኞቻችን ማስክና ሳኒታይዘር የመሳሰሉትን ነገሮች በተቻለ መጠን ለማድረስ ጥረት አድርገናል፤ በዚህ ረገድ በጣም ያገዘን የኢትዮጵያ ቀይ መስቀል ማህበር የደቡብ ቅርንጫፍ ነው፡፡ ይህ ማህበር አንድ አራት አምስት ዙር ይሆናል ችግር ባጋጠመን ቁጥር ማስክና ሌሎችንም ግብዓቶችን በመስጠት አግዘውናል፡፡ ሌላው ደግሞ የሰው ሀይል በጣም ቀንሰናል፤ የግድ በስራ ባህሪይ ክፍት ማድረግ የማንችላቸውን አስቀርተን ሌሎችን ደግሞ በፈረቃ እንዲሰሩ በማድረግ ከ40 በመቶ በላይ ሰራተኛ ቀንሰናል፡፡ ይህም ሰራተኞቻን እንዳይጎዱብን በማሰብ ነው፡ በተለይ በዕድሜ የገፉት ተዛማጅ በሽታ ያለባቸው እረፍት እንዲወስዱ አድርገን ሌሎች ደግሞ በፈረቃ እንዲሰሩ ነው ያደረግነው፡፡

ግን በቂ አይደልም፤ በየጊዜው አካባቢው ዲስኢንፈክት እንዲደረግ እንፈልግ ነበር፤ግን ያ ሁሌ አይሳካም፤ ችግር ይገጥማል፤ ስለዚህ አብዘኛው በማሽን ላይ የሚቀመጡ ሰራተኞች አልኮልና ሳኒታይዘር እንዲጠቀሙ እያደረግን ነው፡፡ ባብዘኛው ጥሩ ነገር አለ ግን አልፎ አልፎ መዘናጋት ውጭ እንደለ ሁሉ እዚህም ይስተዋላል፡፡ በተለይ ከቅርብ ጊዜ ወዲህ ሰውም ስለሚተዋወቅ ነው መሰለኝ ጥንቃቄውን ማላላት ይታያል፡፡

የታቀደው ዕቅድ ወደታለመለት ግብ እያመራ ስለመሆኑ ማረጋገጫና የክትትልና ግምገማ ሥርዓት በዐቢይና ንዑስ ኮሚቴ ደረጃ ምን ይመስላል?

ዐቢይ ኮሚቴ ቋሚ የሆነ የክትትልና ግምገማ ሥርዓት ነበር፤ አንዳንዴ በሳምንት ሶስቴ አንዳንድ ደግሞ ሁለቴ፤ እንዲሁም ደግሞ ቢያንስ አንዴ በዙምና ቪዲዮ ኮንፈራንስ ይደረግ ነበር፡፡ እኛም አስቸኳይ ነገር አጋጥሞን በፈቃድ ካልሆነ በስተቀር እንሳተፍ ነበር፡፡ የግምገማ ስርዓቱ ጥሩ ነበር፤ ሁሉም እንዲያወራ አይጠበቅም፤ ዋና ዋና ጉዳዮች ይነሳሉ፡ከፍተቶች ካሉ ተገምግመው ቀጣይ አቅጣጫ ተቀምጦ ሁሉም ለቀጣይ ተልዕኮ ይመለስ ነበር፤ እኛጋ ደግሞ መደበኛ አሰራር አለ፤ የኮቪድን አጀንዳ የመደበኛ ግምገማ አካል ነው ያደረግነው፡፡ በየቀኑ ብሪፍንግ አለ፤ ኢዲቶሪያል አለ፤ አሁን ከበሽታው ስጋት አንጻር ብሪፍንግ ቀንሰናል፤ ይሂን እንጂ ተግባሩ በበላይ ባሉት አመራር ደረጃ እንዲገመገም እያደረግን ነው፡፡ ይህ ማለት የግምገማ ስርዓት መኖሩን ነው የሚያረጋግጠው፡፡ ሪፖርት በራሳችን እየገመገምን ወደላይም ለክልልም እንልካለን፡፡ባብዘኛው ለሪፖርት ሶፍት ኮፒ ነው የምንጠቀመው፤ ምክንያቱም ከንኪክ ውጭ በ ኢሜይልና በቴሌግራም በመሳሰሉት ነው የምንልከው፡፡ የነበረው የክትትልና ድጋፍ ሂደት ብዙም ችግር ያለነበረው ነው፤ ሆኖም ግን አልፎ አልፎ መቆራረጦች ነበሩበት፡፡

ከቅርብ ጊዜ ወዲህ ግን መዘናጋቶች አሉ፤ በተለይ ኮቪድ በኢኮኖሚ ላይ ተጽዕኖ ፈጥሮአል ስለተባለ የመከላከል ስራው ከሌሎች እንቅስቃሴዎች ጎን ለጎን መሄድ አለበት ስለተባለ አስቀድሞ ክርር አድርገን ይዘን ስለነበረ ከዚያ ጋር ሲነጻጸር የተወሰነ መላላት እንደተከሰተ እናስተውላለን፡፡በዚህ ረገድ የክትትልና ድጋፍም ሥራ በተወሰነ ደረጃ ረገብ እንዳለ ተስተውሏል፡፡ ሆኖም ግን መሰረታዊ ሥራ የሚጎዳ እንዳልሆነ ነው የተመለከትነው፡፡

የኮቪዲን በሽታ ለመከላከልና ለመቆጣጠር ባደረጋችሁት ሂደት እንደመልካም አጋጣሚ የምታነሱት ወይንም የተሻለ ተሞክሮ ነው የምትሉት ነገር ካለ ብትገልጹ?

ቅድም የኮቪድን ምላሽ መሰረት አደርገን በሰራናቸው የማስተማርና የማጋለጥ ስራዎች ሂደት ያየናቸውና የተገነዘብናቸው ለሀገርም የተሻለ ተሞክሮ ይሆናሉ የሚለው የህብረተሰብ መደጋገፍ ነው፡፡ በጣም በሚገርም ሁኔታ እኛም በዚህ ላይ በስፋት ሰርተናል፡፡ በሚገርም ሁኔታ የቤት ኪራይ ከሶሰት ወር ላላነሰ ጊዜ ሙሉ በሙሉ የተው በግማሽ የቀነሱ መኖራቸው ፤ ሌላው ደግሞ የተለያዩ ድጋፎችን ምግብና አልባሳት እንዲሁም ቁሳቁሶችን በማቅረብና ከመንግስት አካላት ውጭ በግለሰብ ደረጃ ሁሉ እየዞሩ የሚያስተምሩ ነበሩ፤ በዚህም የእርስ በርስ መደጋገፍና መተሳሰብ በአዎንታዊ ጎኑ ጨምረዋል ማለት ይቻላል፡፡ በተቃራኒው ደግሞ ሰዎች በተቸገሩት ጊዜ ዋጋ የሚጨምሩትም ዕቃ የሚያሸሹ ሁለቱንም ጫፎች አይተናል፤ ግን ቀላል በማይባል ደረጃ ደሀዎች እንዲረዱ ሰርተናል እኛ፡፡ ሚዲያ ላይ ያልወጡ ግን በርካታ ድጋፍ ያደረጉት የሀይማኖት ተቋማት አሉ፡፡ከእስልምና ከኦርቶዶክስ እና ከፕሮቴስታንት ድርጅቶች በርካታ በጎ ሥራ ያደረጉትን አይተናል፡፡ ይህ ጥሩ እሴት ነው፤ያኛው ደግሞ የሚወገዝ ድርጊት ነው፡፡ ሁለተኛው አዎንታዊ ጎኑ ከቅንጅታዊ አሰራር አንጻር ነው፤ ለምሳሌ እኛ ከሌሎች ክልሎች ሁሉ እንደዋወል ነበር፤ በጋራ እንሰራ ነበር፤ ፕሮግራም አውጥተን ተንቀሳቅሰን ልምድ ልውውጥ እንደርግ ነበር፤ በኦሮሚያ ከኦብን ጋር በመተባበር ሻሸመኔ ሄደን ቅስቀሳ አድርገናል፡፡ አጀንዳው የሁሉም ነው የሚለውን ለማሰተላለፍ ነው፡፡
