# Supplementary material for: Exploring the patterns of multisectoral approach in fighting COVID-19 Pandemic in SNNPR, Ethiopia: A qualitative case study approach
Source: PLoS One. 2022 Feb 25;17(2):e0263667. doi: 10.1371/journal.pone.0263667 (PMC8880945; doi:10.1371/journal.pone.0263667)
Supplement: S3 File — (DOCX) [file pone.0263667.s003.docx]

**Title: Exploring the patterns of multisectoral approach in fighting COVID-19 pandemic in SNNPR, Ethiopia: A qualitative case study approach.**

Abraham A Ali^1^ PhD, [alanoabraham@yahoo.com](mailto:alanoabraham@yahoo.com)

Akmel M Usman^1^ PhD, [abukelsuma09@gmail.com](mailto:abukelsuma09@gmail.com)

Fekadeselassie B Badebo ^1^ MSc, [fekade93@yahoo.com](mailto:fekade93@yahoo.com)

Solomon H Tilahun^1^ MA, sohadi2005@yahoo.com

**^1^ Policy Study and Research Institute, SNNPR**

**Transcription of the Key Informant Interview (English version)**

1. **KII Zone-one**

Key informant I.

Date of the interview: June 10, 2020

Place of the Interview: Office of the Special Assistant to the Chief Administrator

Time of the interview: 10:00- 10:45 AM

Moderator: Abraham Alano, PhD, PI

Note taker: Samuel Sadihun

What is the relationship between the zonal administration office in terms of cholera prevention and control?

COVID-19 is known to be a national and international epidemic in response to the consensus of others. Since the outbreak, we have been active as a zonal administration, especially in the area of ​​prevention.

When we look at this in detail, there is a large committee in the zone. The main committee is made up of 11 subcommittees. A total of 18 structures consist of four cities and 14 woredas and all departments in the zone. Each sub-committee is planning and reviewing the implementation of the plan every three days.

For example, these sub-committees:

- Media and Communication,

- Health,

- Gathering riots,

- Business sector,

- Silence,

- Sanitation Sub-Committee,

- Agricultural development

- Government Service Subcommittee,

- Sub-committee on street children

- Education task force,

- It is explained that the work of education is a sub-committee that deals with the prevention and control of COVID-19 disease.

These subcommittees are implementing their plans.

In addition, we present to the main committee every three days the evaluation of the level of work to be done in each city and woreda. Efforts are under way to prevent the zonal administrator from chairing the main committee.

These subcommittees are implementing their plans.

In addition, we review the progress of the work being done in each city and woreda every three days.

In this process, since the zone is accountable to the state, what is the relationship and working relationship with the state?

This main committee is in direct contact with the region. The zone has prepared the plan and presented it to the region.

The zonal administrator participates in the similar arrangements in the region. We then reviewed the direction to the subcommittees and assessed that we were working on prevention.

How is the COVID-19 disease pressure and risk level in the zone?

It is known that Zone-one, especially Arbaminch town, is a city that has another chance of spreading the disease as there are people coming in from two directions. For example, we are working closely with the security forces as the smuggling is one of the main threats posed by smugglers from Moyale to the southeast via Moyale.

Secondly, zone-one is unique in that in the highlands of our zone, almost every household has migrated from Addis Ababa and the livelihoods of those affected by the disease are declining. We have heard that the disease is spreading in all sub-cities of Addis Ababa. So there are a lot of people who are coming back. There is also a risk that the disease could spread to other parts of the world.

One of the suspects is still being treated at a health center in Darramalo Woreda. But there are many doubts and the result is negative. Since blood tests have been introduced in our area, we suspect that many people might be infected. We still believe that there many infected people in our city, even though we have not enough testing.

In this regard, a home-based survey has been launched in Arba Minch to help better assess the spread of the disease. Temperature measurement was conducted in each house and the health of the entire family was measured by measuring the temperature; the same thing is happening in every district. However, as a zonal administration, we have serious concerns.

As mentioned above, the COVID-19 response task force has been established; what led you to form a task force at this stage?

The main reason is that as the disease is a global epidemic, it is inevitable that we too will be affected, and in terms of the economic and social interaction of the zone and the zonal capital city, it is important to prevent it from running after the outbreak.

As mentioned above, the COVID-19 response task force has been established; the main reason is that as the disease is a global epidemic, it is inevitable that we too will be affected, and in terms of the economic and social interaction of the zone and the zonal capital city, it is important to prevent it from running after the outbreak.

In connection with this, the security committee is working with the media to prevent those who have or suspect that they have such a disease, so that they are aware of the transmission routes and the issues that need to be addressed, and the other fundraising committee considering the potential resources in the event of a crisis. The presence of mobilization, another part of the prevention work, is to prevent people from leaving their homes unless there is a compelling reason to do so, which can be used to support vulnerable people, such as pick-ups, street vendors, who sell wood and manage the disease.

To raise money and to work together to solve the problem; these task forces have been set up, for example, to ensure that inflation does not occur and that the people are not exposed to unnecessary pressures and that inflation does not occur and that prices are stable.

The goal of agriculture is to enable farmers to stay healthy and engage in the production of food. These task forces or sub-committees will carry out defensive work in connection with COVID- 19;

For example, a task force set up in government-related work is a committee that examines how employees can provide services on their own, as it is run by every sector, especially the public service. The above-mentioned sub-committees also have their own missions. This is a challenge that cannot be overcome by one sector alone. All stakeholders are participating.

What is the document that or as the basis for this work as the head of the zonal administration?

Since the onset of the disease, the federal government has developed its own plan at the national level and has set the direction for the state, and the state has donated its share to the zones in the same way. The zone itself has been transformed into districts. Each woreda and city administration has changed its situation to kebeles. Each woreda and city administration is planning according to its own situation (manpower, other inputs).

As a result, employers at all levels of the federal government are planning to cooperate with each other.

The administration has its own plan to guide this work; performance is reviewed every three days.

What is the nature of coordination?

How frequently do you meet?

For example, in connection with the Hygiene and Sanitation Sub-Committee, there is the use of sanitizers, as well as general sanitation, to maintain the physical distance and hygiene of hairdressers. Another is that this is being done in collaboration with the Department of Education and schools, as schools are currently closed and are being used for retention of suspects (quarantine).

All sub-committees work in coordination with each other.

For example, the Security Sub-Committee works in collaboration with the Transport Subcommittee. If there is a problem with the use of transport, a security committee will work to stabilize the area.

In general, each subcommittee meets every three days to review progress. It also notifies the state of the result. This is a clear procedure.

- What about understanding the overall goal of this movement?

It all started with the realization that the importance of preventing and controlling the disease from the beginning is to control the disease in a short period of time and to avoid the threat of the disease.

- What evidence do you have that the activities of the movement so far are moving towards the desired goal?

There are some issues that we consider to be defensive. That is to say, the precautionary measures in the first place are now being reduced as the disease progresses. The fact is, we have to be more careful. This is a sign of adaptation and, on the other hand, a way to make a living at home without having to worry about malnutrition. This has caused many to think less and less. This has just been reviewed.

You have two responsibilities

- Assessing a common goal by leading a joint task force.

As a member of the task force of each institution we have seen so far, it is being reviewed daily and every day, and the sub-committees are doing better than the gaps I mentioned above.

However, when we look at the performance of the districts, there is a tendency to do things differently. ‘Burabuure's (patchy) performance in the first place has been improved by the main committee.

Do you have any challenges in coordinating and leading multiple institutions in this disease response process?

Institutional motivation, in terms of travel together

Although the sub-committees are organized in the context of the prevention and control of the disease, some committees, such as the fundraising committee, have some shortcomings.

This situation is slowly changing. There were gaps in the collection of resources, especially in the woreda area, and there were gaps in how some woredas would be able to prevent or cope with the epidemic, especially in quarantine areas. Schools are said to be closed, and if there is a sudden suspicion of what to eat, what to drink, and what to do in terms of sanitation, we have gone to the so-called places to move from the inconvenient to the ideal. There were limitations

However, despite these limitations, for example, work has been done to organize and equip these centers to provide appropriate services to patients.

So, despite the limitations, it has been achieved. The zone is budgeting for this.

Is there a good experience with this new phenomenon? If so, what?

This is indeed a new phenomenon. It is a new phenomenon, especially in terms of quick awareness, with the participation of innovator and other stakeholders.

KI 2. Zonal Deputy Head for the Department of Women, Children and Youth Affairs

Date of the interview: June 10, 2020

Place of the Interview: Deputy Head’s Office

Time of the interview: 11:15-12:05

Moderator: Abraham Alano, PhD, PI

Note taker: Samuel Sadihun

1. **What is the nature of acquaintance of your institution against the fight COVID-19?**

It is a must for us to work in close collaboration with various sectors and stakeholders. This sector is responsible for more than 70 percent of the population (women children and youth). We are worried that the disease impact exceptionally harms these groups of population. Therefore, we strongly engaged in the efforts to tackle the pandemic.

**How do you describe the level of preparedness of your organization to respond to the COVID-19 prevention and control? How do you relate with organization up and down and also laterally?**

Our sectorial based vertical and horizontal integration is functional. We have very good up and down ward integration. . The regional bureau has sent us the structure which helps to act the issue of COVID. We in turn cascaded the same to the districts and then to the kebele. Horizontally, we have established a youth volunteer taskforce with other sectors for which our sector acts as a secretary.

WE work in close collaboration with many sectors at zonal level. The same is true both upper and lower levels. In the regard to the prevention and control of this disease, we jointly move out to the community to give information at various gathering places. This includes governmental sectors, university, colleges and civic organizations and religious institutions.

For example we work with many civic organizations and association in the fight against this pandemic. These are youth associations, federations’ league, women federation, league and association. They are the backbone of the volunteer services by disseminating information, supporting those people who are at most needs and distributing resources to them.

**How do you ensure that the MSA undertaking sounds strong or functional?**

We have strong collaboration which can be evidenced by the involvement of our top leaders in various taskforces. For instance, the head of the department is a chairperson for logistic committee and I am a deputy chair for the youth volunteer groups. We closely follow the work by conducting regular meetings every week.

*How you describe the functionality of MSA meeting its purpose?*

The challenges related with the intervention of the disease prevention and control in the joint undertakings are:- lack of uniformity in the desired behavioral level at the urban and rural dwellers, lack of personal protective materials for the general public and the youth volunteer group are some to be mentioned.

**Lessons learned and beneficial aspects obtained in the due process of COVID-19 prevention and control.**

One of the opportunities COVID-19 comes with is its ability to jointly mobilize various stakeholders together, which was not the case before. I feel that it has reduced both political and religious differences and brought various people to stand together to fight a common problem.

**KI 3. Zonal Government communication Department**

Date of the interview: June 10, 2020

Place of the Interview: Head’sOffice

Time of the interview: 1:30-2:20 PM

Moderator: Abraham Alano, PhD, PI

Note taker: Samuel Sadihun

1. What is the nature of acquaintance of your institution against the fight COVID-19?

We are the part and parcel of the zonal main and sub taskforces. Among the 10 sub taskforces established in the zone for this purpose one is ours; the media and communication sub-taskforce, which our department is chairing. This sub-taskforce is mandated to work closely with all other sub-taskforces and main taskforce by documenting all information and also broadcasting to the public what each taskforce is doing. Moreover, we are continuously indulged in information dissemination with the health departments and others who are working in this direction.

What it looks like the multisectoral collaborative undertaking in the fight against COVID-19?

In a nutshell, I can say we are working better by collaborating with all stakeholders in our zone. Our department is responsible for communication affairs in which it takes lion share in information dissemination. The department of health and regional communication bureau are among the top we work together. We do so by sharing information on daily basis with upper and lower levels.

What guided or initiated the collaborative intervention against the prevention and control of COVID-19?

We noted the disease is a borderless in terms of nations, societies and demographic variables. No country is spared from the disease be it rich or poor. Thus, we transmit information to the people with the recognition of this issue in order to mobilize people and sectors to involve in the prevention and control of the disease.

The disease prevention and control efforts mainly based on the regional manual and the main taskforce adopted plan. On the top of these documents, we also get information from the national and global media about the magnitude and challenges the disease causes. It was terrifying to see while human dead bodies buried like as that of the warfront mass decree. We first never noticed that problem is coming to us. But, not far enough, the problem is affecting us.

KI 4. Zonal Education Department

Date of the interview: June 11, 2020

Place of the Interview: Head’s Office

Time of the interview: 9:15- 10:10

Moderator: Abraham Alano, PhD, PI

Note taker: Samuel Sadihun

1. What is the nature of acquaintance of your institution against the fight COVID-19?

In my feeling, education sector is one of the sectors badly threatened by the disease. Schools are closed and shifted to quarantine and isolation centers. We are worried how to withstand the burden the disease could cause in the entire school communities.

There is clear structure indicating the organization of multisectoral actions. Regional government has established taskforces jointly and in sector specific. When our sector is concerned, the taskforce is structured from region to the school level encompassing all stakeholders.

In witness on the functionality of the multisectoral actions, we work in close collaboration with the health sectors and other stakeholders from governmental and non-governmental organizations. We move in a taskforce to support and supervise how things are being carried out.

How do you describe the need for MSA for the actions related to COVID-19prevention and control?

Look; the disease nature is terrifying. Leave alone us, the poor guys. This disease puzzled the global nation’s categories as most developed ones. Therefore, we have realized that unless we move together, we cannot manage this deadly pandemic disease. This is how we have established a joint action points together.

Issue of having joint plan in the stride to prevent and control the disease through joint undertakings:

Another dimension that symbolizes the need for integrated actions is the presence of multiple taskforces. The main taskforce is mandated to indicate where the virus positive people shall stay and they indicate the school. However, schools are under the education department and its is thus out mandate to furnish the school for the purpose.

How you ensure whether the actions are taking you to the set goal accomplishment?

We follow the intervention of the plan whether it was intervened as per the plan or any gaps identified. As I have already informed above about the two mandates given for our sector at this time; one is availing the school compound for the quarantine and isolation and ensuring the continuity of the teaching learning process via the virtual means. Hence, we have developed the checklist to check whether the actions were conducted based on the plan. By doing so, we see the actual intervention and if any gap identified, take corrective actions.

What lessons learned or any beneficial experiences observed during the response against the COVID-19?

Despite its numerous burdens the COVID-19 disease put on us, there are also some positive contribution we gained in the due process of its prevention and control. As an education sector, it gave us to look to our radio and television based educational systems carefully. We have checked the functionality and coverage of our radio based education. It has helped us to improve our technology use and efficiency on resource use.

Covid-19 disease occurrence has contributed significantly to improve the multisectoral collaboration. A lot can be mentioned in this regard. For example; this is a disease, means an enemy. It is clear that to fight such debilitating enemy, we shall stand together. One of the best examples in strengthening the collaboration is its improvement in the quality of information. Formerly we have discrepant information of the orphaned children attending school. The date we have and that at the labour and social affairs were not matching. But, since we have started to work together, we have matching information and eased our action.

KI 5. Zonal Finance Department

Date of the interview: June 11, 2020

Place of the Interview: Head’s Office

Time of the interview: 11:00- 11: 50 Am

Moderator: Abraham Alano, PhD, PI

Note taker: Samuel Sadihun

1. What is the nature of acquaintance of your institution against the fight COVID-19?

We are part of the main taskforce and also chairing one of the many sub-taskforces related to resource mobilization. We have also established a designated disease prevention and control team in our institution. Our efforts are cascaded to the district level following our sectoral structure. Using all these structures, we mobilize resources to aid the disease prevention and control tasks.

We have recognized that our department has numerous customers thus interacting with many people in a day base. Therefore, the risk of disease acquisition is well noticed and we have established a team that takes the responsibility to disseminate information and avail all necessary preventive materials in the department compound and all zonal, woreda level sector offices.

In the due process of working together with various stakeholders, we have established both vertical and horizontal links. The vertical link is the regional finance bureau and regional sub-taskforce for resource mobilization.

How do you describe the need for MSA for the actions related to COVID-19prevention and control?

The indication for the multispectral action in our area related to the fight of this pandemic are the establishment of various taskforce and committees, platforms for joining planning intervention, monitoring and follow-up. We have regular meeting sessions both at the main and sub taskforce levels. However, the MSA approach is not uniformly functioning in all hierarchies. As has been seen at some districts level, there have been staggering and gaps in the joint undertakings.

How you ensure the actions are taking towards goal accomplishment?

I have faced surprising issue ones up on a time. Some professionals requested me to pay periderm beyond the guideline. Youth have asked me to distribute the collected money for the disease issue for them as they feel that the disease is an artifact simply created for the political purpose.

Lessons learned and beneficial aspects observed during the fight against the COVID-19

The contribution of COVID-19 for collaborative efforts are numerous.. One to be noted is in relation to the information flow and access. For example a lady from Addis Aba came to Daramalo was said to have a COVID. All who heard this information including farmer rushed to communicate the issue to the responsible authorities.

KI 6. Labour and Social Affairs Department

Date of the interview: June 11, 2020

Place of the Interview: Head’s Office

Time of the interview: 2:00-3:00 PM

Moderator: Abraham Alano, PhD, PI

Note taker: Samuel Sadihun

1. What is the nature of acquaintance of your institution against the fight COVID-19?

We, the labour and social affairs department own the issue of Covid-19 as our prim responsibility. Our stakeholders are numerous. The structure we have established for joint actions include health department, food security division, and various religious institutions, community organizations non-governmental organizations higher education institutions (colleges and university). We began our duties first developing a joint plan at main taskforce level and shared our sectoral and sub-task force plan. And, part of the plan is cascaded to the lower level structures.

What has initiated the MSA for the disease prevention and control?

The reason why multisectoral approach is necessary for the fight against Covid-19 is clear for us, labour and social affairs sector. The reason being; our sector deals with the most vulnerable group of people who are at highest risk for the disease exposure. We made a base on our existing sectoral policy and the directives forwarded from the regional and zonal governments.

How to ensure the goal accomplishment?

We have been doing the monitoring and follow-up process by gathering together very frequently. At the early stage, we used to meet every other day and seriously evaluate the performance. We encourage the success to continue and suggest ways to improve drawbacks. However, this enthusiasm over the time started to lag and we are not in a similar manner this time.

With regard to the responsibilities and accountabilities related to the COVID-19 prevention and control in aggregate approach, these have been indicated in our joint plan. The plan acts as a binding document for all participating stakeholders to execute its share.

Lesson learned and any beneficial effects observed in the due processes of the disease prevention and control

One of the outstanding contributions of the occurrence of covid-19 is its capacity to consolidate the joint efforts by minimizing divisive attitude. I saw great shift in this regard. Stakeholders put the human being at center and honored the need. It created better means to shrink the polarized thinking and brought in to the center, all stands together despite the differences to fight the pandemic.

KI 7. Zonal Health Department

Date of the interview: June 12,2020

Place of the Interview: Head’s Office

Time of the interview: 6:00-7:00 PM

Moderator: Abraham Alano, PhD, PI

Note taker: Samuel Sadihun

1. What is the nature of acquaintance of your institution against the fight COVID-19?

Being the health department, our acquaintance with the disease prevention and control is obvious. By the time when the COVID—19 was declared a global pandemic, we did not wait the organized multisectoral arrangement. We started to prepare ourselves how to deal with the problem. Later, the federal, regional and zonal administration has established structures that would better respond to the pandemic. Based on the designated approach, our sector is functions as a secretary for the zonal main taskforce and I chair the the disease prevention and treatment sub-task force at the zonal level. There are plenty members under this sub-taskforces composed of health related sectors, NGOs, training institutions etc…

What it looks like the MSA intervention and what initiated it for this purpose?

The burden of the disease is beyond imagination and we all were frustrated. When we heard the first case in our region, we all started to shiver. The problem is now knocking every door. The burden in our zone can be sited multiple. However, I frankly point out that our effort is not comparable with the dearth of the disease, which has to be improved.

In my view, the current strength of the multisectoral approach across the region is not in congruence to the level of the problem. I can say that we had better commitment and actions at early stage but now for unclear reasons, the efforts are shrinking.

As a health sector, we made our basis to organize ourselves for the prevention and control of the pandemic on the directives given from the minister of health and the regional health bureau. We made a clear differentiation and integration of efforts to comprehensively address the issue of our routine health programs and the prevention and control of the new pandemic. Emergency operation centers are organized across the health sectors from the federal level to the district to handle this matter.

The collaborative undertakings within the health sector efforts and other stakeholders as focused on the joint efforts evidenced by the strong functionality of the EOC. The EOC is the center piece of the actions related to the disease prevention and control. On daily basis, EOC follow the undertakings. Starting from the community surveillance to the treatment center actions, all are being assessed, discussed and ways forward enacted.

How you ensure that the current intervention moves you toward the desired direction?

As the health sector, we have established a strong follow-up and monitoring mechanism. The EOC on a daily base follow each activity carried out by designated taskforces and conduct meeting on a daily base. For your surprise, at this time they are on meeting evaluating the overall daily undertakings. Every day the EOC share information with me via the telegram. Look, they send their daily meeting outcome to me. They outlined issue that needs my attention. Once I receive from them, I share in my part with the Zonal Chief Administrator. By doing so, we solve the ongoing gaps and forward remedies to be carried out.

What lessons learned and beneficial aspects observed?

The occurrence of this disease has contributed for the strength of the health system in general and the disease surveillance system in particular. This has given us added capacity to prevent and control diseases such as measles, malaria. The hand washing practice is another value helped not only to prevent covid-19 but also other communicable disease.

The battle with covid-19 has created remarkable opportunities for the health sector. It has given opportunity to strengthen the health information system. Moreover, the virtual meeting we set to follow the efforts of the disease prevention and control enhanced our means of using technology. These in turn helped us to easily access information from various institutions vertically and horizontally.

**Key interview center two**

KI. 1. The Ethiopian Red Cross Society, Southern Branch

Date of the interview:

Place of the Interview: Head’s Office

Time of the interview:

Moderator: Abraham Alano, PhD, PI

Note taker: Mr. Solomon Hail, MA.

1. What is the nature of acquaintance of your institution against the fight COVID-19?

Responding to the public emergencies like covid-19 is one of the main responsibilities of the Ethiopian Red Cross Society. The day we heard about the occurrence of the pandemic in the glob and Ethiopia launched it as area of concern, we set our usual readiness plan and engaged collaborating with the sectors mandated to tackle the problem.

We joyfully joined the main taskforce when the regional government called us to take part in this arena. They invited us to take a lead position in organizing the volunteer services, which is the central issue for the Red Cross society. With enduring experiences and enthusiasm, we joined the taskforce and acting together to solve the alarming problem. The volunteer services we chair include institutions and organizations such as women federation, teachers’ association, youth federation, sport commission and 12 other sectors together. This platform has brought 368000 volunteer s and deployed across the region to disseminate information and support those who are at most in need.

What it looks like the MSA intervention and what initiated it for this purpose?

Our collaborative intervention looks like this. We gather together weekly on Friday via video conference and share information. We evaluate our performances, the success and drawbacks. We act most issues at our level and present the most pressing and strategic issues to the main taskforce. And, we act according to the directives given by the main taskforces. By doing so one learns from other and enriches our efforts to prevent and control the disease.

The base for our action is the joint plan developed at the main taskforce at the regional government level. We took our share as the sub-taskforce for the intervention of volunteer service and resource mobilization. We have cascaded part of the plan to the lower hierarchy to implement accordingly. These efforts have got appreciation for the supervisory team from the federal inspection team. Thus, we are courageous to strengthen the efforts we have started until the disease remains not a problem for our society. And, this is the sign to what extent we are effectuating our activities in a collaborative way.

How you ensure that the current intervention moves you toward the desired direction?

We have an established follow-up and monitoring and evaluation approach. We do these in three ways: video conference, telegram channel and conducting in person visit to the sites. Every taskforce members when went out for the field work, they immediately share the finding to me at the common telegram channel. On daily base, we do this and develop a common understanding what is going on out in the field.

On the top of our designated activities, we share information with other sub-task force when issues come to our attention and need their actions. For example, if any breach of the state of emergency happens on the site where we do our actions, we directly present the matter to the peace and security taskforce in order to act and correct the incident. By doing so we ensure the horizontal collaboration and witness the goal of the disease is a joint goal accomplishment.

Lessons learned and beneficial aspects observed:

Let me keep aside the burden of covid-19 pandemic and only focus on the opportunities we have been gaining. The occurrence of this disease has improved the personal hygiene, mainly the hand hygiene. I have one friend by profession a pediatrician. Let me share what he said to me in one occasion. He said to me that “ you disappeared, what has happened?...He meant that the frequency of visit to his clinic had reduced. I somehow started to lough and in my inner said, o! yea, it was the virus and bacteria contamination that force to visit to your clinic frequently. As now we stick we stick to the proper hand hygiene, no as such diarrhea, colds and other communicable disease affecting us. Thus, I took a lesson to strengthen this practice even for the future.

Another remarkable observation with the positive sides of the pandemic is the enrichment of the collaborative actions. The experience in the sub=taskforce which I chair, I see a tremendous commitment of all who participate in this team, they are fast and energetic. When I call a meeting, they spear instantly. I guess, your observation and experiences in other sub taskforces remain the same. Moreover, it has created exceptional opportunity to know each other. Formerly, we barely know the roles of each other. But, as we began to work together, we got chance to see what each does. For us it has created to promote the actions of our institution as well. I pledge for myself to further strength this experience for our other issues after the control of the disease. We have many assignments to accomplish through joint actions.

COVID-19 has also come with the inertia of innovation. It forced people to innovate. This time, my organization engaged in the production of sanitizer and face masks and working strongly to produce medical gloves. We export some sanitizers to Kenya. This also helps our revenue generation and solidarity with the neighbor country. What surprises me is that the brain and hands which now producing these items had been with us earlier. But, when the forcing situation comes, we have started to struggle and engaged in productive actions due to covid-19.

KI 2. Health Bureau

Date of the interview:

Place of the Interview: Head’s Office

Time of the interview:

Moderator: Abraham Alano, PhD, PI

Note taker: Mr. Solomon Hail, MA.

1. What is the nature of acquaintance of your institution against the fight COVID-19?

As soon as we heard that covid-19 is the global pandemic, the regional government established a taskforce to respond to the pandemic even before the federal minister of health passes directives. At the moment, the main taskforce was supposed to be led by the regional president and the technic committee to be chaired by the bureau of health. However, eventually we have seen the need to modify the technic committee as the challenges of the disease is so deep and wide that requires the expansion of involving stakeholder. As the result nine more sub-taskforces were added to the efforts.

About the conformity of regional and federal level action plans:

Our plan to the prevention and control of the disease made both global and national sources of information. As it was indicated that 80% of the exposed people either remain asymptomatic or show mid symptoms, the 15 %need hospitalization and of which 5% will need critical care and 2 % will need artificial ventilation. By taking these evidences, we began to prepare ourselves in order to establish various centers for quarantine, isolation and treatment. We have developed a regional main plan that guides all other sub=taskforces plan. All other sectors and sub-taskforce developed their plan in consultation with the main plan. The plan is the guiding document for the entire activities.

How to ensure the goal accomplishment?

At this point in time I do never dare that we have successfully accomplishing our duties, but we are doing important efforts to tackle the problems. There are many drawbacks on the way. Some social occasions are propagating disease spread. I fear, the second disease wave in the region would be alarming.

The evaluation at the lower hierarchy is well articulated and we do share the same. The overall disease prevention and control efforts at this time look sluggish unlike the previous time. We were meeting three times in a week earlier but this time not ones in a week. As a result, the regional president has called a meeting both the regional and zonal taskforce members on the way how to revitalize the efforts. He well stressed on the overall weakness being manifested at every stages. Finally he put ways forward to strength the efforts.

Lessons learned and beneficial aspects observed

The occurrence of this disease can be seen as an opportunity to the health sector so because it has forced the higher level policy makers to give due attention to the sector and identify the critical shortages. Now, everyone started to consider how much the health issue is universal one. From our side also it has forced us to critically evaluate our overall preparedness to withstand the challenges. We saw the weakness in our previous approach where little attention was given to the diagnostic technologies.

Another area the disease occurrence helped to improve is the local budget allocation. Most health budgets were dependent on foreign aid but when this disease happened globally, the rich countries stranded on their home issue and little attention was given for developing countries like Ethiopia. This has forced the local government to allocate budget for the pandemic control by shifting priorities.

KI 3. Bureau of Women Children and Youth Affairs

Date of the interview:

Place of the Interview: Head’s Office

Time of the interview:

Moderator: Abraham Alano, PhD, PI

Note taker: Mr. Solomon Hail, MA.

1. What is the nature of acquaintance of your institution against the fight COVID-19?

I consider the joint efforts to tackle the covid-19 pandemic from the wide spectrum of the society, women, children and youth. These portions of the society composes of more than 70% of the total population and highly exposed to the disease, Therefore, the efforts to control the disease necessitate the joint efforts of multiple sector engagement. It is well accepted that the desired result cannot be attained if we move alone as the customary sectoral approach. The width and depth of the disease related impacts requires the must multisectoral approach.

In the stride to prevent and control the covid-19 pandemic, our sector follows two approaches. At the regional level our engagement starts at the main taskforce that led by the regional president. We act as a member of the main task force and chair the logistic committee. The other one is we work in close collaboration with the federal mister. We consolidated the overall actions at our sector level by establishing sectoral based main and sub-committees that share the plans both from the federal and regional governments. We customized the plan to our context and act accordingly. These approaches cascaded to the lower level until to the women development armies.

We have a platform to follow the activities intervention at the zonal and special woreda levels both at the main and sub taskforces. To this effect, we have set office telegram page via which all posts their daily report. We in turn evaluate the actions based on the report and provide feed backs. By doing so, we ensure the multisectoral action for the prevention and control of the disease.

How do you describe the functionalities of MSA in the stride for the prevention and control of COVID-19?

One peculiar nature our structure is we have a women development armies that extend to the community and household levels beyond the kebele. The presence of this structure helps us to transmit information to the most vulnerable groups in the society.

The women development armies and related structure work very hard to materialize the COVID -19 prevention and control by providing support for those who are economically and socially vulnerable. They respond to the call of the prime minister, food sharing ‘maeid magarat’. They collect money from those who are better of when compared to their counterpart. The contribution was in terms of finance, in kinds such as food item and the motto of one birr for one needy woman. Through these approached they have collected to about 29 million birr amount both in cash and kind.

Resource mobilization for the covid-19 prevention in our region follows the three approaches: 1) cloth and food items are collected and distributed through the accident prevention preparedness commission; 2) sanitizers and related hygiene supplies through the Ethiopian Red Cross Society and the medical supplies including drugs are through the health system. The distributions of materials are based on the decision of the main taskforces based on the clear criteria. The overall actions base on the collaborative undertakings.

The overall collaborative actions evidenced by the join interventions both multisectorally and sectorally. This is further evidenced by the monitoring and follow-up conducted on the daily basis at the sectoral and sub task forces and three times a week at the main task forces.

In the efforts to tackle the covid-19 pandemic, we work closely with our federal minister, sharing plan and receiving various support. We do also communicate twice in a week, thereby give performance report and receive feedback from them. They have visited out action in person and gave material support for the people in need women and children.

Lessons learned and beneficial aspects observed:

The multispectral collaboration in relation to the fight against covid-19 has developed remarkable experiences in joint efforts. I recall the challenges related with multisectoral actions before COVID-19. However, when covid-19 came into appearance, all sectora moved together, lend hands mobilized resources by prioritizing the neediest people. I witness that this is a special occasion where in my experience I saw how all governmental, non-governmental civic and philanthropic organization team up.

Our sector collaborates with various sectors including media sector. Surprisingly, in the endeavor for the disease fight, all put their efforts without reservation. We moved together to provide information to guide the disease prevention and control to various group of societies. Our efforts extended to all vulnerable groups such as street children, elders, and sick people.

What aspects need improvement?

Despite continuous provision of health information to prevent and control the disease, the level of public awareness and their preparedness to cope with the problem remains rudimentary. People are in seriously taking the matter. Yet, they stand together in crowded manner in social setting. Hand washing is not practices in the way it has to be. Public ceremonies are still in place. They worship together. These all show how discrepantly people are acting in the contrary to the established disease prevention and control ways.

The efforts first started with great worries and enthusiasm but eventually most people including top leaders started to walk back. In our region, other competing agenda such as regionals structural request out compute the current public issue. Negligence was rampantly exhibited by most portion of the society.

The bottle neck related with the prevention and control disease occurrence limiting public movement. As the result, some people forced to stay home. The elongated home stay created situation that force for domestic violence. Personal assaults, abuses and disagreements happened. Many conflict incidents happened disrupting family relations. I have evidence such as in 15 zones and districts there had 108 domestic violence documented 68 abductions also occurred.

**KI 8. : Finance Bureau**

What is your office's overall connection to the COVID-19 prevention and control process?

• Collaborate with others in the field of coordination, such as planning and directing your organization to be qualified for the mission.

Since the outbreak of COVID-19 in the country on March 3, it has been set up as a major committee of the regional government. Our office is a member of the main committee and under the direction of the sub-committee fundraising.

We have organized a technical committee comprising of various experts from our office and other institutions to form a fundraising committee.

In this regard, 324 million birr has been collected so far in the form of cash and materials. Accounts for this service have been opened in each structure as far as the district. Cash collected in the opened account is credited. This bank account has been opened by the government and the private bank at the local banks. During this fundraising, there was a great deal of motivation and cooperation from the people; We are amazed at the fact that the one who has the money is delivering the other kind of cow calf and all the milk and cows without hesitation. I believe that the response of the community to the food crisis may have been experienced not only by the country but also by African countries.

Therefore, the values ​​we have seen in this process of accumulating wealth should be enhanced in the future. As it is known that our region shares borders with two African countries, we are working with security and health institutions to ensure that the people who cross the border have access to quarantine, medical and food services. For example, if we do not raise awareness in advance to raise wealth, wealth cannot be given to anyone. Only an intelligent person can give wealth, and on the other hand, if the society is not aware and does not try to defend itself, it is useless for us to accumulate wealth a thousand times.

Under our committee there is a sub-committee that does good work other than fundraising. This is done by educating and educating the public about the disease. We are working together in this way.

What motivated you to do this COVID-19 work this way?

One of the reasons is that after the outbreak of this epidemic at the national and national level, a committee should be formed to work with the federal government / prime minister to organize the regional and sub-national structures.

Therefore, the direction is from the federal and state governments. It is also a decision of the regional government that we must save our citizens. The second is that we, as a fundraising committee, have prepared the Federal Ministry of Finance Directive No. 63/2012 E.C., and we have prepared Directive No. 31/2012 E.C., following that directive. The third source is the various state of emergency.

What these proclamations should be implemented: What needs to be done for citizens to implement these mandatory conditions: There is one who has no power, Unemployed: How can these locks be saved? Can the government alone do all this? This is not possible; What if it is not possible? It is on the basis of these three principles that we have to mobilize the people.

How do you assess the current and current impact of COVID-19?

The pressure is too heavy; Now that we are in charge of the economy, the disease has spread in our region and people are shocked by the lack of economic interaction, which has had a huge impact on the revenue collection process. The economic crisis is worrying in the direction of prevention and control and the restriction of activities due to the threat posed by the disease.

Another threat is social unrest; Out of the 25 structures we have, more than 18 structures are failing to pay their monthly salaries. I mention this where the budget declared by the budget body cannot be brought to the treasury; more than 400 million birr tax has been canceled in Hawassa town since the government imposed tax breaks in some sectors. As a result, it has not been able to pay its salary since May.

The pressure is even greater on the larger economic zones and cities. There is a lot of pressure in the way we are evaluating, but there is a lot of distraction. There is also a great deal of cognitive impairment. So we are working on the idea that a very serious situation could arise.

As an office, what are you doing to protect your own employees and customers from COVID-19?

According to the office, we are working to prevent the disease from the door. First, our staff is divided into two groups, with older and more preoccupied health workers working from home and working in shifts in the morning and afternoon so that others do not have to work harder and have more physical distance between them. We bought a sanitizer and a mask for all employees so that they could wash their hands when they came into the office.

We have purchased a touch-free hand washing machine from the College of Technical and Vocational Training and placed it at the entrance to our campus. We are not only providing water and showers to the staff and clients, but we are also monitoring the situation. As our office is known to have a large number of customers, we were forced to wear a mask when they came to the first office; We are using various communication methods such as e-mail communication, video conferencing, video conferencing, and physical communication.

Are you assigned a specialist at the bureau level to coordinate this internal defense and control work?

Yes, there is a person assigned to this, The head of the bureau, Ato Zerfu Atanafu, is in charge of this. We are in charge of human resource management. In addition to being organized in such a way, we, as the head of the office, are jointly evaluating their direction and directing them.

In terms of coordinating coordination, what is the tendency to co-lead, respond, and revise plans?

As you know, in our country's committee work, there is a tendency to go out quickly and sometimes to drag. The current movement is not at the beginning; It's a hot spot where we meet three times a week and meet from the bottom line as well as a video conference on Thursdays and Thursdays. The same is true of the sectors; But now there is also a slowdown in current national affairs

We have no problem planning and combining that with the underlying structure. But in terms of implementation, there are foot drags in terms of monitoring and evaluation. Sometimes there is a tendency to move forward and sometimes backward,We have an organized document about the plan. When we do this, we also work with other sectors. For example, in collaboration with the Bureau of Urban and Housing Development, the Bureau of Health, in collaboration with the Federal Ministry of Health, we have developed a hard copy and soft copy for GPS prevention and control work.

- What does the monitoring and evaluation system look like on both sides?

Monitoring and evaluation work is underway. Regarding the evaluation of the main committee's work, the regional office of the Prosperity Party will also monitor the performance of the management in a checklist. According to the fundraising committee, we are monitoring the situation to see if the money has been misappropriated.

This is not only for us, but also for the implementation of the state of emergency by the Federal Task Force. Therefore, I can assure you that our fundraising system has witnessed this and that it can be documented. How much money went into the account opened; How much property was collected, And exactly how many people received proper support? We will monitor the preparation of the checklist.

In some places, we have seen shortcomings. When we ask where the money and property are, we collect it, but they report it as if we have distributed it. Most of the time, they are motivated by me. While it is not recommended to do support monitoring as usual, we will be using technology to review and exchange information on video conferencing and group telegram accounts on a daily basis.

What is the positive impact of the new procurement directive from the Federal Government?

Regarding the implementation of public procurement, the regional government has its own Proclamation No. 146/2004 and 28/2010. However, due to the long-term procurement process for the prevention of CVD disease, it is not possible to save lives if the bidding process is not carried out following the previous financial bureaucratic process. .

This is a decision made by the regional government. The performance will be monitored. The directive emphasizes that the collected assets should be registered with the IEBX system in order to comply with the government's financial system so as not to be wasted by individuals or the government. It is designed to be followed by a general audit and follow the government's procedures for the use of the Government Revenue Arvey (Revenue Model 19 and Expenditure Model 22).

In this regard, we have revised a national view to ensure that some of these perishable food items, such as pasta, macaroni, oil, sugar, salt, etc., are distributed and distributed to consumers without damage. This is how we challenge the procurement and financial system.

Are there any gaps in the monitoring and evaluation process and are you correcting them?

First, through the various methods we have monitored and evaluated, what we have found and corrected by the Telegram, the zoom Conference, and others. And the wealth that was gathered will be divided, Realizing that they are using this wealth for their own benefit, we have suggested that it be corrected.

In connection with this, when a suspect came to Moyale and a large number of alleged accomplices entered the detention center and everyone started to panic, we were reassured that we were sure of what we had to say about the disease; Beyond that, when we look at the relationship, we see that the donation we have seen is only for the rich, and it is not understood that everyone should go as far as to share their share of the table. In this regard, it is important to understand that there is an input that can be given to different sections of the society, especially farmers and pastoralists.

As you can see, there is still a wide gap in terms of awareness and application, especially in terms of use of masks and protection of physical distance. If this gap needs to be fixed urgently; In fact, what we see here in Hawassa is that even if the problem is not rectified soon, we have suggested during our support and monitoring that our citizens should be exposed to more problems and our efforts should be corrected in vain.

Are there any issues that could be considered a good experience in the process of responding to CV-19?

As you mentioned earlier, one of the things you can do is keep up the pressure. An investor from Dilla, for example, called me to take my building and keep the suspects in check. As I reflect on the situation, it shows the community's concern for others; It is a good idea to say that if my citizens survive, I will work tomorrow. The second is that our use of technology has changed dramatically. It is often the case that people who are not tech-savvy come close to technology; Not only does it make things easier but it also makes us more resourceful. We have saved up to two hundred million birr for the various meetings we have been spending from the zones.

The meeting was held in the presence of the President, the Office of Agriculture and Natural Resources Development and others. Another important result, in my view, is that everyone who has come here in person, but in practice, has been able to keep up with Facebook's virtual meeting today.

The meeting was held in the presence of the President, the Office of Agriculture and Natural Resources Development and others. Another important result, in my view, is that everyone who has come here in person, but in practice, has been able to keep up with Facebook's virtual meeting today.

We've had a lot of meetings in this process, especially in terms of respecting time. This indicates that we are in a difficult situation and that we are experiencing another positive outcome. However, I say that we still need to go a long way in realizing that we need to go the extra mile.

KI 9. Bureau of Agriculture and Natural Resources Development

What is your office's overall connection to the COVID-19 prevention and control process?

• Collaborate with others in terms of coordination:

It is known that we are planning to do a lot of work since it is expected to take place in our country and in our region. In the process, we have set up a task force at the regional level to plan how to prevent and control the disease. One of the institutions included in this task force is our (Bureau of Agriculture and Natural Resources Development).

n connection with this, we assume that there will be food shortages and clothing shortages due to covid. Another increase in CVD-19 disease is affecting agricultural production processes and outcomes. This affects the process of making agricultural inputs accessible. Second, there is a negative impact on the deployment of manpower to farmers.

It is working with stakeholders as a task force to address all of this. One is to provide food aid to those in need in our region who are facing food insecurity in our region and with the support of the federal government, these communities have been identified as food aid.

In addition to providing shelter and food for the children on the streets, the people of our region, who are scattered in different parts of the country, are now in need of food and shelter.

So we have been working to identify the most vulnerable, not just for everyone, but for the most vulnerable. We also support people with disabilities in this framework; We did our best to provide food assistance from the zones.

What motivated or motivated the region to form a task force? Is there an original idea supported by various documents? How do you understand the severity of the disease?

The CV-19 incident is a shock to our region; Therefore, the first reason is that he does not know the cause and prevention of the disease in the world.

| The reason is that the problem is a new phenomenon and no cure or vaccine has been found for the time being, and the response should be to take into account the scale, magnitude and urgency of the problem and to involve the entire population. The World Health Organization (WHO) has declared that the disease is a global epidemic, and we need to work together to develop guidelines for the prevention of the disease. | The reason is that the problem is a new phenomenon and no cure or vaccine has been found for the time being, and the response should be to take into account the scale, magnitude and urgency of the problem and to involve the entire population. The World Health Organization (WHO) has declared that the disease is a global epidemic, and we need to work together to develop guidelines for the prevention of the disease. |
| --- | --- |

The decision to allow older mothers with asthma and other illnesses, such as asthma and other illnesses, to take a break and stay at home, which could make them more susceptible to coronary heart disease.

This and similar activities have been carried out not only to generate ideas but also to put them into practice. We have done this work only if a proclamation is issued at the federal level. After that, a federal state of emergency was declared. In connection with this, various directives have come in. Since this was published, I have been following these guidelines and regulations and correcting any gaps.

What is your coordination with the Federal Ministry and the Federal Disaster Risk Management Commission?

Coordinating work, then, is relatively good from top to bottom. Especially when it comes to our office, our priorities are not limited to our local food supply. FDRE disaster risk management support is needed. We are working together to provide our diverse food and clothing needs to disaster management. The other big impact is our agricultural system. This affects the supply of logistics as a country and as a region.

It is having a significant impact on the supply of inputs such as fertilizer seeds and various pesticides. For example, you need to download and install to provide input. These downloads have a negative impact on supply, as they are more vulnerable to copycat. For example, we have experienced a shortage of manpower to unload and load fertilizer at the port of Djibouti. There are also cases of cochlear implants; This is a great shock to others.

This has had a negative impact in the short term, but they have to talk to the Ministry of Agriculture about how to prevent it. We need to work together to solve problems by consulting with the Ministry to ensure that the impact is not too severe. The same is true of seed supply; Seed supply comes from our state and federal agencies. We are also working on these in a coordinated manner. The other is the employee. Our development station staff is expected to consult and support the farmer in person.

In the past, they had access to many farmers at the same time. They also go from house to house. But now you can't get many farmers at once. So what you can do is go from house to house and talk to them: To this end, we have provided personal protective equipment, such as masks, sanitizers, and hand sanitizers, to more than 20,000 people in our area. From the bottom line, we have helped farmers understand how to prevent the disease and provide them with protective equipment.

Moreover, due to the impact of agricultural inputs and manpower, production deficits are expected to remain at the regional and national levels. Therefore, preparation for the CVD response has been prepared at the level of the Ministry of Agriculture. We agree on that, At the forum convened by the Prime Minister, we agreed to play our part. Returning here, we have prepared a response plan for our region. Prepare a detailed plan and become the Zonal Task Force (Zonal Chief Administrator) with the structure of all our sectors, including the zonal agriculture and natural resource development department.

At the video conference, we discussed the details of the detailed plan and reached a consensus. The next stage of this plan was discussed at the zonal, woreda and kebele level. That is to say, the link was created from the top of the Prime Minister's Office to the Kebele. A detailed plan for how to compensate for the productive defects caused by the disease has been developed and implemented. So the connection looks like this.

Crowd protection work as an office, especially in the case of the appointment of a staff member or the conduct of an office manager.

There are two offices in this compound and this building and the institutions under it. Employees here also need to protect themselves from copyright. The worker is defending himself and can support the farmer, so a task force has been set up to lead that. According to our office, that task force monitors the proper functioning of the immune system; For example, you may have seen a notice posted here stating that no one is allowed to enter the premises without a mask.

If there is a defect here, this task force will review it. Second, the provision of sanitizers and hand sanitizers and the monitoring and supervision of hand washing are the responsibility of this task force and its sub-task forces. This task force works in consultation with both offices.

This process is known to be costly and where do you get the money from?

We also have a shortage of resources this year in terms of resources. But even the limited resources we have are a priority for human well-being; it should be. In this regard, the region's main task force has allocated about 70 million ETB for COVID-19's response. This, then, is not a backup; Capital projects have been halted, and secondly, they have been reduced by a percentage of the training budget and transferred to a reserve fund by allocating some work to next year.

When we come to our office, we are buying personal protective equipment, shifting some of the work for the next year and reducing the budget for some, in response to COVID-19. Second, we are moving away from the support of training and procurement of our projects and programs and programs in partnership with our office. We have also mobilized non-governmental organizations to purchase personal protective equipment at a total cost of up to ten million birr. This is ten million as the Bureau of Agriculture and Natural Resources, and with the addition of the Bureau of Livestock and Fisheries.

What does monitoring and support and evaluation systems look like as a main task force or sub-task force to make coordination work more effective?

Fundraising is the responsibility of a subcommittee. A regional plan has been prepared and implemented. And from this he descended to the zones, therefore, the wealth was collected in cash and in kind, even in the zonal and special districts. There are gaps in terms of mobilization of resources, from zonal to zonal, from zonal to zonal, from special woreda to woreda, in terms of conducting and evaluating the work, and in terms of community ownership. Beyond that, relative mobility is good.

Other than that, it requires that the collection be used for its intended purpose. Since resources are collected in kind and in cash at the zonal and special woreda levels, it is necessary to closely monitor whether they have used this properly. There seems to be a gap in this. Of course, I think it will be evaluated, but it is not enough.

How often did you meet to review this work? And what was the imposition of accountability if the joint plan was not implemented? It was three times a week because it was new and shocking. It was appropriate; In the process, we met about two days a week. As I waited, I noticed dripping. That is not to say that we did not pay attention, but we did see that the work was becoming more institutional. In terms of accountability, I did not realize the seriousness of the problem and the lack of accountability. I don't know if we probably didn't work there. However, in spite of its shortcomings, there is no clear line of reasoning. I think it's better to take it for granted.

It was three times a week because it was new and shocking. It was appropriate; In the process, we met about two days a week. As I waited, I noticed dripping. That is not to say that we did not pay attention, but we did see that the work was becoming more institutional. In terms of accountability, I did not realize the seriousness of the problem and the lack of accountability. I don't know if we probably didn't work there. However, in spite of its shortcomings, there is no clear line of reasoning. I think it's better to take it for granted.

What if there are situations or events that can be cited as opportunities and experiences in the process of preventing and controlling CVD?

Whenever there is a challenge or challenge, there is another opportunity; this means that the ways in which we are going to face this challenge are creating new potential for us.

One of the ways we can prevent this Covenant-19 is to keep it clean; However, since the advent of handwashing, a wide range of personal hygiene practices have been introduced and a series of awareness-raising messages have been issued. Although this has not been confirmed by research, we find that diseases caused by poor hygiene have decreased; We expect it to decrease, It is also scientific. I think the community will make it a tradition to wash this hand regularly even if Kovid is gone. I say this is a good opportunity.

Another good opportunity is related to the use of technology. For example, if the Internet has been around for a long time, we have used it very limited. Probably a factor as to why they're doing so poorly. We are now holding video conferences because we are not allowed to gather in one hall because of Covid-19. This means that we are one step closer to using technology. Therefore, it can be said that it has created an opportunity for him to become acquainted with technology and to make technology part of the day-to-day operations of our institution.

This allows us to use our resources more efficiently and to avoid some unnecessary expenses. For example, calling some zones and woredas for some meetings cost us millions of dollars by video conferencing. When we were short of resources, we canceled our important face-to-face meetings, leaving us without success, which left a huge gap in the monitoring and support process.

With this we are reviewing our work: This is a good opportunity. Thus, in addition to saving money, we also have the option of working efficiently. Another is the opportunity to generate technology. There are times when a person is creative. For example, the introduction of touch-free hand washing machines is one example. As a result, efforts are being made to produce a variety of medical supplies and medicines. It can be said to encourage creativity.

Key informant ten

Southern Radio and Television Agency

What is your organization's overall connection and contribution to the CVD-19 prevention and control process?

• Collaborate with others in the field of coordination.

We have been a member of the committee from the national level to the media, from the national level to the time when it has been reported that there is an outbreak of CVD-19 disease in the world and in our country. In that media committee, we are organized on one wing, which also includes media mobilization and public relations work. We operate as a member of the main task force established at the regional level. We have been working continuously since the beginning; He explains the following in depተ

I would describe the whole situation in two parts: One is the media committee from the federal level, which is headed by the press secretary of the prime minister's office at the federal level, where all government and public media in the country meet. All in all, these nine, the big ones at the state and federal level, such as IBC, Walta, Fana and ESA Press, are the line through which we meet and exchange information and evaluate the work. What we are doing here is primarily to make sure that the body that is doing this defense work in the country does not become divided on issues that are considered to be the basic direction in terms of education and defense, and to create a consistent media and communication work.

This is being reviewed regularly and we will report back; This is being reviewed weekly. This process is also a feedback loop where we exchange information and feedback every day with the created group. Other new developments related to Covid, both domestic and international, are widely used and should be highlighted.

For our part, we are working in a manner that is consistent with the country. We are a member of the main task force at the regional level. Then there is our technical committee, which is a media editorial. There are content managers and directors below, including me; The Technical Committee is the body that prepares the agenda; In this process, we focus on two main things: One is teaching; Teaching is basically divided into two parts: What has he meant since the first Kovid happened? How can it be prevented? What kind of character does it have? What precautions should one take? Inter alia. It is a work of teaching.

After discussing this teaching work not only at the center but also at the main committee, there is a committee at the technical committee level, including the regional government communication bureau, as well as some other offices with the science and information bureau. It is from that committee that we take ourselves as a technical committee and work.

So this technical committee has ten branches. We will send the main messages to these branches. We will translate the message on FM stations in 48 languages ​​and reach the community. Teaching is primarily about prevention. We have done a lot of work to inform the behavior and how to prevent it. We have done a great deal of work; We are engaged in this large-scale work to the extent that it undermines the institution's standard plan; Although we have a lot of support from the government, there is a plan to collect internal revenue - we have a duty to collect more than thirty million birr; That money is allocated as part of the institution's budget.

However, we do a lot of free work; This is a social responsibility and you can't stop working if you don't bring money. We have also done extensive teaching work on prevention. We worked in coordination with the branch offices. I am one of the bureaucrats who are involved in the teaching process, and they are primarily responsible for providing accurate information. This is in accordance with the instructions, and when we need information, the head of the health office can come in from anywhere, even by phone.

If he is here, he will enter the studio and provide information. It means Mr. If he is not, Dr. Mamush will give. Not only descriptions but also standard programs were widely available to us. In direct public discussions, these people respond to incoming questions; Some conversations last up to two hours. They explain this process: If there is confusion, they call it quits. So we worked in coordination with them.

Second, other sector offices, such as the Bureau of Trade and Market Development, have worked closely with us because of the wide range of problems with the supply of market management; Singing hymns contain various messages that alert the public: Head of the Bureau of Culture and Tourism Women and Children Bureau as well.

For example, we have made the headlines, for example, well-known individuals, artists, and athletes from the region, and we have made it possible for them to convey a message through the recording. They came to us from other institutions, We went to them and did something. In terms of time, we do this work on a daily basis because it is extensive.

Based on our information that you are working with the State Executive Committee and the Federal Technical Committee, what is the status of the two plans? How do you feel if you are unreadable in terms of supporting and helping each other?

One of the tasks I have described so far is: This is to enable teaching. The second is exposure. A state of emergency has been declared to help prevent the disease; There are circumstances in this Proclamation that prohibit many activities that may aggravate the spread of the disease.

We have worked extensively on these issues, as there have been a number of actions, including the disappearance of goods and the increase in the price of goods. We have done extensive exposure work; In connection with this, we have been greatly assisted by the Police Commission and the Regional Peace and Security Bureau; We did a lot of work together. We have done extensive work on these two issues. The plan, which began with the federal government, helped us move the federal government in the right direction.

For example, if the Amhara region is doing something else and we are doing something else, it will not be a consistent solution because it is an epidemic; This includes not only information but also communication; Therefore, there is no contradiction between what comes from the state and what comes from the federal. The more I live in the region, the more organized I am. Limited Areas This zone is called Woreda Ya Urban Administration and each area is under review. What kind of media coverage should be provided in the quarantine area?

The one with the federal government will not be compromised because the direction of the country will be compromised. We have never had a problem with reading.

How do you prepare to protect your employees and clients?

One of the most vulnerable workers in CVD, both in our country and internationally, is ours. They run to make information accessible; So what we did was start with our institution in terms of hygiene. We started by saying that when employees enter an institution, they should wash their hands at the door and show it to others and take them to committee meetings. Since then, many institutions have done better.

So we tried to do this, We strive to provide as much as possible to our staff, such as masks and sensors; The southern branch of the Ethiopian Red Cross Society has been a great help in this regard. This association will be one or four rounds. Every time we have a problem, they help us by providing masks and other inputs. Another is that we have greatly reduced manpower; We have reduced staffing by more than 40 percent by eliminating the need to be open-minded and allowing others to work in shifts. This is so that we do not harm the workers.

But it is not enough; We always wanted the area to be disinfected, but that didn't always work out; There is a problem: So we are making most of the workers on the machine use alcohol and sanitizer. There are usually good things to do, but there are exceptions to this rule. I think it's easy to be cautious, especially since people have recently become acquainted.

What is the status of the plan and the monitoring and evaluation system at the level of the main and sub-committees?

The main committee was a permanent monitoring and evaluation system; Sometimes three times a week and some twice; There was also at least one video conference.

We were involved in an emergency, except when we had to. The review system was good; Not everyone is expected to talk; Major issues will be raised. There is a standard for us: We have included the CVD agenda as part of a regular review. There is freezing every day, Editorial We are now reducing the risk of the disease; However, we are reviewing the performance at the highest leadership level. This means that there is an evaluation system. We evaluate the report ourselves and send it to the region. We usually use a soft copy of the report; This is because we send emails and telegrams without any contact.

The monitoring and support process was relatively smooth; However, there were occasional cuts.

But lately, there has been a flurry of activity, In particular, we have already argued that the defense has been linked to other economic activities, and that there has been a slight weakening. However, we have seen that basic work is not harmful.

What, if anything, do you think would be a good opportunity for you to prevent or control colic?

What we have seen and realized in the process of educating and exposing ourselves based on the pre-Covenan response is the support of the community that will be a better experience for the country. Surprisingly, we have worked extensively on this. Surprisingly, the rent has been completely reduced by at least three months; Another was providing food, clothing, and supplies in various forms, as well as teaching on a case-by-case basis.

s a result, mutual support and concern have increased. On the contrary, we have seen both extremes in the face of adversity. But we have worked hard to help the poor. There are many religious organizations that have not been published in the media. We have seen a lot of good work from Islamic, Orthodox and Protestant organizations. That is a good value, and it is a bad thing.

The second positive aspect is in terms of coordination; For example, we were in all other regions; We worked together; We set up a program and exchanged experiences; In Oromia, we went to Shashemene to mobilize with Obn. The agenda is to convey that it belongs to everyone.

1. Zone-two

**Interviewer ­­­ ………………………………..Yoseph Watte & Semagegn Dansa**

**Interviewee ……………………………….. department Head of City Development and Construction**

**Place of Interview …………………………Office of the head of the department**

**Date:**

**Time- Started ..................................................................…...3:30**

**Ended ………………………………………………………..4:30**

**Consent for Audio Record ……………………..…………. Confirmed**

**Questions**

1. ***What is the link between your institution and COVID-19 Prevention and controlling?***

- ***In coordinating, supporting and supervising higher and lower tiers; with other departments; and/or your direct engagements in Prevention and control of the pandemic.***
- ***Establishing responsive institutions***
- ***Institutional capacity to work with other competent departments and institutions***

According to Ato Hanjalo, Cities and towns are more exposed to the pandemic since the COVID-19 transmits in social contacts and related factors. Therefore, the department feels and acts preventing and controlling the pandemic is primary responsibility of the sector. For this reason, the department of City Development and Construction is member of Zonal Grand Committee (ዋናዉ ግብረ-ሃይል), Main Committee (አብይ ኮሚቴ), Sub-Committee (ንዑስ ኮሚቴ ) and Technical Committee (ቴክኒክ ኮሚቴ ). This structure enables different sectors to work in collaboration with other. Accordingly, there are eight towns in the zone. The structures are devolved into Woreda and town administrations are chaired by Chief Administrator and mayor respectively. This makes the department to work in coordination with other parallel sectors, and supporting and supervising lower tiers. According to Hanjelo, responsive institutions are also formed in all towns (six town) with clear duties and accountability structure. Accordingly, the target is to bring attitudinal changes in the society in line to the directives of Federal government.

1. ***How you label the participation of your institution in responding to the COVID-19?***

- ***In differentiating the extent and character of the pandemic***
- ***Taking primary responsibility (Who initiated Prevention and control of COVID-19?)***
- ***In supporting those who are taking primary responsibility in prevention and controlling***
- ***What kind of approach could be effective in prevention and controlling of COVID-19?***
- ***How do you see the manner of engagement of different stakeholders?***
  - ***Its necessity***

The head of the department labels the participation of the sector as strong and warm in prevention and controlling. Accordingly, this is not only because it is the command from higher level government structure but also because of the nature of the towns/cities where social contact, movement and interactions are favoring the expansion of the pandemic. Yet, there are limitations in understanding the extent and coverage of the pandemic because of limited testing capacity. Regarding characters of the pandemic, thanks to information from Ministry of Health, means of transmission and symptoms of the disease are clearly known and communicated to everybody through different technologies.

According to the informant, the initiation of prevention and controlling came from regional government in line with directions from federal government. He explained that the sector supports and collaboratively working with other Departments such as Heath and Peace and Security which are providing technical reports for implementation of State of Emergency. All sectors are working in an integrated manner with higher, lower and parallel sectors to control and prevent the pandemic. It is highly cooperative engagement among the stakeholders.

1. ***What is the role of your institution in prevention and controlling of COVID-19?***

The head of the department said that working strongly to transform the ways of life is crucial since our usual way of life is favoring the transmission and expansion of the pandemic. For this purpose, according to him, they are using some influential individuals such Wolaita Dicha FC Players, university students, Business men, elders and religious leaders. Moreover, as he said that, the department has made frequent spray of chemicals in different places of the town and mobilizing the residents to keep clean their compound and the surroundings.

1. ***If you are participating in prevention and control of COVID-19, how do you do so? What kind of principle do you use?***

- ***If you have any supportive document***

He revealed that the department is participating in line to the guideline set by the regional governments and some contextualized implementation strategies at zonal administration level. With this fact, to protect some vulnerable peoples the sector congregated such as street children, from different places in Soddo town and trained on how to prevent and control the pandemic. Eventually, the department returned them to their families with all necessary sanitation materials and with some amount of food items.

According to the Wolaita Zone, City Development and Construction Department, the leading principle in COVID-19 Prevention and Controlling is making cities/towns neat and clean than ever. To put this into ground, the guideline from the regional government directs ways of organization, resources mobilization and utilization of those resources. There are also supportive documents while the sector carries the mentioned duties. But, the head of the department also revealed that there are limitations in implementation of these directives of National State of Emergency statements.

1. ***How do you assess your institutions response against the Pandemic?***

- ***If any responsible person assigned for the purpose***
- ***If any institutional plan cascaded from common plan***
- ***If any allocation of resource necessary***
- ***If any supervisor and support***

As the head explained, the department takes COVID-19 Prevention and Controlling as the primary duty to save the life of the people. For this purpose, peoples are assigned at Grand committee, Main committee, sub-committee and technical committees. Plans from Federal and regional government are cascaded for concurrent use. Resources are mobilized and collected in kind as well as in cash. The department head substantiated that in a day, 4.5 million birr was collected in Soddo town. This is to say that public engagement to tackle the pandemic is so warm. These activities are carried with due supervision and support.

1. ***If you are working with other institutions in collaboration, they how do work in such relation? What is the guideline?***

- ***If any supportive document to woke together***
  - ***Implementation of Commonly deigned plan***
- ***Goal clarity and common understandings of goals***
- ***If you are working together, how often you meet and at what level you cooperate***
- ***What is responsibility of your department in the group work? At what level you participate?***

As the head, because of the nature of the pandemic, the sector is working in collaboration with other sectors as mentioned in the above. This is partly the direction from federal and regional government on how taskforces to be organized and to function collaboratively. Thus, they have shared plans to work vigorously towards creating goal clarity and common understanding. For this end, they meet every three days and discuss on the activities. As it is indicated, the head of the department is member of grand committee and chairing the sub-committees in the department.

1. ***What evidence do you have that the approach so far used is leading to the targeted goal?***

- ***Support and supervision***

The interviewee said he has no evidence that the approach is leading to the intended goal., though the public has information on the means of transmission and its eventual effect, it found to be reluctant to implement the principles in the day to day life, according to the informant. Thus, actions and public behaviors are favoring the expansion of the pandemic regardless of support and supervisions.

1. ***If you have joint meetings and how often do you take such meetings***

- ***If you use technological inputs***

The joint meeting with other departments is taken at every five day. And if any urgency is there, it can take at any time. To take meetings and for interpersonal communication technologies are used such as zoom and telegram, said the department head.

**Interviewers ………………………….Yoseph Watte & Semagegn Dansa**

**Interviewee …………………………… Department of Health**

**Place of Interview ……………………..Office of the head of the department**

**Date: ……………………………………….**

**Time- Started …………………………… 12:30 (evening)**

**Ended……………………………..1:30 (evening)**

**Consent for Recording…………………...Confirmed**

**Questions**

1. ***What the link between your institution and COVID-19 Prevention and controlling is look like?***

- ***In coordinating, supporting and supervising higher and lower tiers; with other departments; and/or your direct engagements in Prevention and control of the pandemic.***
- ***Establishing responsive institutions***
- ***Institutional capacity to work with other competent departments and institutions***

According to the head of the department, prevention and controlling of COVID-19 is principally the duty of the health department. But, because of the pandemic nature of the disease, it gets the attention of everyone. According to the informant, following the first incidence of it in Ethiopia, which was happened March 13, 2020 ( መጋቢት 4፣2012 ዓ.ም), in the next day which was March 14, 2020 ( መጋቢት 5፣2012 ዓ.ም), rigorous meeting was held at zonal level lunching a taskforce to act against the expansion of the pandemic.

To be responsive to the problem, the health department professional are divided into two service sectors namely pandemic control and regular duties, according to the informant,. The latter groups are to sustain provisions of regular health services as mandatory and overlooking it may result in big health crises. Moreover, as he stated, the pandemic control section composed of different sub-sections such as incident management, operational section, planning section, logistics section, lesion office, and surveillance office and so on. He depicted that, some of the members in these committees are experienced individuals who have participated in Ebola control in West Africa. The committees are working in collaboration with Wolaita Soddo University.

According to the head of the department, situations are evaluated by these technical committees and forwarded to main committee which in turn forwards to grand committee. Grand committee discusses on the issues presented by health department and passes decisions. These all indicates according to Ato Desalegn that there are coordinated and collaborative efforts among different parallel departments and supervision and control with higher and lower institutions.

1. ***How you label the participation of your institution in responding to the COVID-19?***

- ***In differentiating the extent and character of the pandemic***
- ***Taking primary responsibility (Who initiated Prevention and control of COVID-19?)***
- ***In supporting those who are taking primary responsibility in prevention and controlling***
- ***What kind of approach could be effective in prevention and control of COVID-19?***
- ***How do you see the manner of engagement of different stakeholders?***
  - ***Its necessity***

Ato Dessalegn, the head of the department, labels the participation of his institution as strong as enough since the professionals are dedicated for the purpose. Accordingly, the sector differentiates the extent of the pandemic and its nature which enables to use appropriate strategy to act on it. Therefore, the department of Health takes primary responsibility in bringing issues into attention of the grant committee. The department head also said that abiding to the directives of Ministry of Health and State of Emergency in general are the best approaches to prevent and control the transmission of the COVID-19 pandemic. To accomplish this goal, all stakeholders are warmly participating and engaging at different levels.

1. ***What is the role of your institution in prevention and controlling of COVID-19?***

The role the department is paramount in both clinical and non-clinical activities in giving professional services to the public say Ato Dessalegn. For example, hiring/ employing additional medical doctors, making door to door body temperature screening by using health extension workers which covered 91% of the total population of the zone. Accordingly, in the campaign, 41 individuals found with COVID-19 symptoms. These individuals were hosted at different identified schools until tested in laboratory. Luckily, all of them found negative to the pandemic. Side by side, as the head indicated resource mobilization, developing laboratory equipments, establishing quarantines and employing additional professions in the sector was accomplished. Moreover, health professionals were lodged at six different entry tolls namely *Goja* from Dawro, *Abala Abaya* from Arbaminch, *Buge* from Shashemene, *Asho* from Gofa, *Achura* from Hassana and *Beleila* from Hawassa for temperature screening. But, the interviewers intervened on the moment reacted that they have not encountered such screening at entry points. Then the head of department admitted the possibility of such absences and took the comment as input for future action.

1. ***If you are participating in prevention and controlling of COVID-19, how do you do so? What kind of principle do you use?***

- ***If you have any supportive document***

According to Dessalegn, Yes staffs of the department are participating at individual level and collectively to work bilaterally and multilaterally with different relevant stakeholders such as participating in grand committee to mobilizing the whole community in resource generating and to awareness creation among the public.

According to the head, the main principle for prevention and controlling of the pandemic is adherence to principles of World Health Organization which are cascaded down through Federal Ministry of Health and declared through state of emergency. There are supportive documents for this endeavor and ‘concept notes’ designed in the department to contextualize the directives.

1. ***How do you assess your institutions response against the Pandemic?***

- ***If any responsible person assigned for the purpose***
- ***If any institutional plan cascaded from common plan***
- ***If any allocation of resource necessary***
- ***If any supervisor and support***

According to Dessalegn, the head of the health department, health sector professionals consider themselves as state soldiers who are prepared to maintain territorial integrity and political independence. They are ready to scarify their life to sustain the lives of the generation. To supervise the achievements, therefore, there are focal person assigned for the purpose. Institutional plans were cascaded down from shared goals. Resources are also allocated for the prevention and treatment purpose as already stated in the above. Supervisions and supports are also going on as the head described.

1. ***If you are working with other institutions in collaboration, then how do work in such relation? What is the guideline?***

- ***If any supportive document to woke together***
  - ***Implementation of Commonly deigned plan***
- ***Goal clarity and common understandings***
- ***If you are working together, how often you meet and at what level you cooperate***
- ***What is responsibility of your department in the group work? At what level you participate?***

Accordingly, the health sector is working with other institutions collaboratively. Especially, the role Wolaita Soddo University is vital. The cascaded directives favor such collaboration for resource mobilization and working for common goal. The youth club, Red-cross, businessmen and Soddo University are very close in supporting departmental activities. There is goal clarity and common understanding among sectors. There are documents encouraging working together for plans designed across sectors. For the success of this, according to the head, joint meetings are taken at every three days.

1. ***What evidence do you have that the approach used so far is leading to the targeted goal?***

- ***Support and supervision***

According to the informant, the approach presented by Ministry of Health and enforced by State of Emergency are important and feasible to achieve the targeted goal. However, practically, there is big limitation in the implementation of the principle, according to the head. For example, there is limitation to transport samples and getting medical dresses which are pertinent to COVID-19. But, support and supervision in this regard is working with full efforts.

1. ***If you have joint meetings and how often do you take such meetings***

- ***If any use technological inputs***

According to the informant, joint meeting are held at every three days or at least once in a week to discuss the progress and to take the necessary action. On the moment, he revealed successes of new thinking and innovations to overcome challenges. For example, IC mechanical ventilator innovated by Wolaita Soddo University student which is important to fill the shortage of ventilator. In addition to this, sterilizer was invented by high school student to deter the transmission of the virus from customers. As he stated, papers, shoe and phones are sterilized before used in offices.

Another technological input probably in facing the pandemic is the way of teaching the public about the transmission and consequence of the pandemic. For this intention, as the head said, in Wolaita zone there are Wolaita language based songs which are highly articulated and touching the emotions of the public are produced and on broadcast in the society.

**Interviewer …………………………………Yoseph Watte & Semagegn Dansa**

**Interviewee …………………………….......... department Head of Finance**

**Place of Interview ………………………….Office of the head of department**

**Date:…………………………………………….**

**Time- Started ……………………………………………….8:00**

**Ended……………………………………………………...…..9:00**

**Consent for interviewee ……………………………………Confirmed**

**Questions**

1. ***What is the link between your institution and COVID-19 Prevention and Control Mechanisms?***

- ***In coordinating, supporting and supervising higher and lower tiers; parallel departments; or your direct engagements in Prevention and control.***
- ***Establishing responsive institutions***
- ***Institutional capacity to work with other computing departments and institutions***

The finance department which is crucial for logistic mobilization declares that it is working in coordination with other parallel institutions; and supporting and supervising and lower and higher institutions in prevention of the pandemic. The sector mobilizes resources and apportions them to relevant bodies. According to the head, 132 million birr was planned to be mobilized at zonal level for the prevention and controlling the pandemic. Of these, 18 million birr is already collected in coordinated efforts of different sectors. Such resources are deposed only in COVID-19 account which exists in all 22 Woredas of the Wolaita Zone. The account is controlled by Finance and Health departments concurrently. In addition to this, the finance department subsidized 3 million birr from its treasury for this purpose. For any possible purchasing, the finance purchasing section already secured permission from its regional Bureau to conduct direct purchasing, according to the informant. To ban any possible corruption, the collected money deposited in code-19 whose owner is the Finance department and moves to code-22 with the consent of Health department.

As the head’s statement, they have not yet used the cash deposit while donations in a kind are used at different instances. Accordingly, these all are to indicate that responsive institutions are established with adequate institutional capacity to working with other competent departments and institutions. For actual implementation of these resources committees and subcommittees are organized.

1. ***How you label the participation of your institution in responding to the COVID-19?***

- ***In differentiating the extent and character of the pandemic***
- ***Taking primary responsibility (Who initiated Prevention and control of COVID-19?)***
- ***In supporting those who are taking primary responsibility in prevention and control***
- ***What kind of approach could be effective in prevention and control of COVID-19?***
- ***Who do you see the manner of engagement of different stakeholders?***
  - ***Its necessity***

Ato Tektle said that, his department’s participation in COVID-19 Prevention and Controlling is strong since the nature of the pandemic seeks huge resources and logistics that force the institution to be more responsible. Though the initial concern came from Federal government and followed by regional government, the zonal government mobilized the public and all its sectors in the day next to the day of the identification of infected person at Addis Ababa. With this case, our sector started to involve in major tasks of resource mobilization to actualize the endeavor.

The head of the department indicated that they are using the approach proposed by federal government (keeping social distance, putting masks, washing hands frequently and using sanitizer and other alcoholic elements and the like). For this end, there is wide engagement from different stakeholders such as Non-Governmental Organizations and Business men, which is crucial, in mobilization of resources in supporting government efforts. But, still to date, there is dearth in implementation of the directions set forth.

In the meantime, he indicated the intention to have a separate budget code in next year (2013 E.C) for COVID-19 from government treasury. Accordingly, this enables to respond to the problem effectively where stakeholders’ participation swings. And if the pandemic is controlled in advance, the budget could be shifted for other public purpose.

1. ***What is the contribution of your institution in prevention and controlling of COVID-19?***

Our department acts as one the actors in Prevention and Controlling COVID-19. It is primarily responsible in resource mobilization and distributing such resources to respective offices, the head said. Since, the pandemic is exponentially expanding; it is costly and needs more money and resource to tackle it. Therefore, according to the head, the department is strongly engaged in resource mobilization.

1. ***If you are participating in prevention and control of COVID-19, how do you do so? What kind of principle do you use?***

- ***If you have any supportive document***

Our department is robustly participating in prevention and control of the COVID-19. It is done in all possible directions with stretched efforts in having bilateral as well as multilateral relations as per the direction of the Federal government and also some informal communications at lower, higher and parallel sectors. These participations are minutes that are documents to confirm the statement.

The main principle of the Finance department, as the head said is collective engagement to control and prevent the pandemic. According to him, human survival comes first to any other activities and that all mankind regardless of any differences should work in together to achieve the goal. To achieve these objectives, there are supportive documents as substantiated by hard copy provided herewith.

1. ***How do you assess your institutions response against the Pandemic?***

- ***If any responsible person assigned for the purpose***
- ***If any institutional plan cascaded from common plan***
- ***If any allocation of resource necessary***
- ***If any supervisor and support***

According to Ato Teketel, ways of dealing with to the problem is adequate as resource mobilization duty is significantly going on to increase deposited cashes in the COVID-19 account. For this purpose, peoples from the department are assigned at Grand committee, Main committee, sub-committee and technical committees. Guidelines/Plans from Federal and regional governments are cascaded for concurrent use. Resources are mobilized and collected in kind as well as in cash. These activities are carried with due supervision and support.

1. ***If you are working with other institutions in collaboration, they how do work in such relation? What is the guideline?***

- ***If any supportive document to woke together***
  - ***Implementation of Commonly deigned plan***
- ***Goal clarity and common understandings of goals***
- ***If you are working together, how often you meet and at what level you cooperate***
- ***What is responsibility of your department in the group work? At what level you participate?***

The Finance department head stated in the interview that they work in collaboration with other institutions. In this regard the role of Wolaita Soddo University is paramount. Through its community service, it is serving as quarantine center for those who are infected and even for suspected ones providing all necessary food items and clinical services. Street children are also supported by the university. The guideline is partly the university`s legislation which encourages to give community service and the directives set by federal and regional governments; as the head said. Thus, they have shared plans to work vigorously towards creating goal clarity and common understanding. For this end, as the informant, they meet every three days and discuss on the activities. The head of the department is member of grand committee and chairing the committee in the department.

1. ***What evidence do you have that the approach so far used is leading to the targeted goal?***

- ***Support and supervision***

According to the informant, there is no evidence that the current approach is directing to prevent and control the COVID-19. This is because that the approach is not effectively implemented by the Peace and Security department of the zone. Though the informant saying so, as interviewee’s observation, heads of departments they themselves were not implementing the directions set in the state of emergency. They do not put on face masks and hosting large number of customers in their office at a time. At Soddo town there were public demonstrations where no one was intervening to stop. Actually, support and supervisions are there at all places though they seem for sake of reporting.

1. ***If you have joint meetings and how often do you take such meetings***

- ***If you use technological inputs***

The head of the department indicated that frequent meetings are held at zonal and sector level. As per the guideline, in every three days there joint meetings. But, in the finance sector, there is no new technological input innovated to cope up our strategies to the problem occurred.

**Interviewer ……………………………….Yoseph Watte & Semagegn Dansa**

**Interviewee ………………………………… department of Transport and road development**

**Place of Interview ………………………….Office of the head of department**

**Date: …………………………………………………..**

**Time- Started …………………………………………………. 8:30**

**Ended……………………………………………………9:30**

**Consent for Audio Record…………………………………… Confirmed**

**Questions**

1. ***What is the link between your institution and COVID-19 Prevention and controlling?***

- ***In coordinating, supporting and supervising higher and lower tiers; with other departments; and/or your direct engagements in Prevention and control of the pandemic.***
- ***Establishing responsive institutions***
- ***Institutional capacity to work with other competent departments and institutions***

The head of Transport departments, Ato Samuel, explained that the transport sector works in collaboration with other parallel, higher and lower sectors in the zone and beyond it. The department makes appropriate support and supervision to the prevention and controlling of the pandemic. Since the major way of the transmission of the pandemic is social contact, issues around transportations are series.

Accordingly, bus stations have their own mini-media to teach and aware the public about the COVID-19 Pandemic. At entry of stations, everyone has compulsory body temperature screening. With this effect, the department works to implement national direction of boarding 50% of expected number travelers. All travelers also must put on either surgical or at least community masks. In the meantime, according to the informant, accidental and regular controls and visits are taken over transportations. This is taken in collaboration to police force and roads security sectors.

The head of the department also indicated that, for transports coming into the zone, there are six tolls at entry points. According to the informant, this is for two reasons. One is to block illegal transportations which are boarding outside bus stations. This is because, a one who comes outside bus station, his/her temperature screening is not done and other necessary procedures cannot be fulfilled. The second reason is for temperature screening. For this purpose, health professionals are lodged at six different entry tolls namely *Goja* from Dawro, *Abala Abaya* from Arbaminch, *Buge* from Shashemene, *Asho* from Gofa, *Achura* from Hassana and *Beleila* from Hawassa doing so. Hence, there is no entry and exit for public transport before and after 6:00 am and 6:00 pm respectively.

1. ***How you label the participation of your institution in responding to the COVID-19?***

- ***In differentiating the extent and character of the pandemic***
- ***Taking primary responsibility (Who initiated Prevention and control of COVID-19?)***
- ***In supporting those who are taking primary responsibility in prevention and control***
- ***What kind of approach could be effective in prevention and control of COVID-19?***
- ***How do you see the manner of engagement of different stakeholders?***
  - ***Its necessity***

As it is informed, the nature and character of the pandemic necessarily invites the transport department to strongly participate in Prevention and Controlling. Most stakeholders have differentiated the extent and character of the pandemic. For this purpose, initiation came from Federal and regional governments and enforced by Zonal governments. Following that, the sector is taking primary role and supporting others who play significant role in prevention and controlling of the pandemic.

The informant of interview describes that transport by the nature is hard to manage. This is because of it complicated nature such as the two legged motor cycle transport from town to the rural areas are carries more people beyond the standard and moving in different unmarked paths. Even in public transports while a person pays double of the normal rate, there is a trend of carrying of more peoples. This is related to corrupt actions of some traffic policies and irresponsibility of transporters for their life and for the life of others.

Like all other departments, the department of transport also tries to apply the deterrence approach which complements national directives which are declared with the state of emergency. For implementation of these, it tries to invite all relevant bodies especially business men are engaged in the transport sector.

1. ***What is the contribution of your institution in prevention and controlling of COVID-19?***

The contribution of the transport sector is vital since the pandemic moves with human and other non-entities and not in air. Therefore, making the transportation services and related activities safe and free from COVID -19 related infections are going on at every possible means. That is why checkpoints at entry of the zone are established. The support of other sectors especially Health and the police force is also important.

1. ***If you are participating in prevention and control of COVID-19, how do you do so? What kind of principle do you use?***

- ***If you have any supportive document***

According to the interviewee, the Deterrence approach in collaboration with other sectors is the primary principle of the department to save lives of the generation. As details mentioned in the above, the department is taking all possible action on transports before entering into the zone and within the zone. As already indicated prevention and controlling is taken collaboratively to achieve the intended goal. To so, there are documents (hard and soft copies) that govern the intervention strategy and tactic.

1. ***How do you assess your institutions response against the Pandemic?***

- ***If any responsible person assigned for the purpose***
- ***If any institutional plan cascaded from common plan***
- ***If any allocation of resource necessary***
- ***If any supervisor and support***

As the informant, it is not successful even though the public has awareness about prevention and controlling techniques. The public has not made it as their way of life. It became chasing transportations at night and days. The lack of consistency from local government over implementation is found to be another challenge. But, responsible persons are assigned from the department for the purpose. Institutional plans are cascaded from general directives and necessary resources are allocated for the implementation. In the resource mobilization, the role of youth associations, Red-cross, businessmen and Wolaita Soddo University was paramount, as the head of the department. This all is done with supervisions and support.

1. ***If you are working with other institutions in collaboration, they how do work in such relation? What is the guideline?***

- ***If any supportive document to woke together***
  - ***Implementation of Commonly deigned plan***
- ***Goal clarity and common understandings***
- ***If you are working together, how often you meet and at what level you cooperate***
- ***What is responsibility of your department in the group work? At what level you participate?***

As the informant said clearly, the prevention and controlling of the pandemic is by nature needs coordinated and collaborative efforts. For this reason, the department of Transport is part of Grand committee, Main committee, sub-committee and technical committee. Its activities are also carried with close collaboration of Health and Peace and Security departments. In order to so, there are supportive documents for the implementation of cross-sector plans. According to the informant, through series of discussions, goal clarity and common understanding is established. For the collaborative engagements, meetings made in every 15 days at sub-committee level and at every week at technique committee level. In all committees, the department has membership roles.

1. ***What evidence do you have that the approach so far used is leading to the targeted goal?***

- ***Support and supervision***

According to Ato Samuel, regarding goal of Prevention and Controlling of the pandemic, we have no evidence that we are on right truck though things look silent in the zone and infected ones recovered. He even said that things look silent may be because of limited testing capacity. In all these cases, supports and supervisions are there.

1. **If you have joint meetings and how often do you take such meetings**

- **If you use technological inputs**

For the collaborative engagements meetings made in every 15 days at sub-committee level and at every week at technique committee level. Regarding the usage of technological inputs, there are practices such as body temperature screening, putting on masks and using sanitizers in transportations.

**Interviewer ………………………Yoseph Watte & Semagegn Dansa**

**Interviewee ……………………… department of Peace and Security**

**Place of Interview ………………..Office of the head of department**

**Date: ………………………………**

**Time Started …………………………………8:30**

**Ended …………………………………9:30**

**Consent for Recording ………………………..Confirmed**

**Questions**

1. ***What is the link between your institution and COVID-19 Prevention and controlling?***

- ***In coordinating, supporting and supervising higher and lower tiers; with other departments; and/or your direct engagements in Prevention and control of the pandemic.***
- ***Establishing responsive institutions***
- ***Institutional capacity to work with other competent departments and institutions***

Ato Tessema, the head of the department of peace and security said that the sector has primary responsibility for the implementation of directives set by government in prevention and controlling the COVID-19 pandemic. It is the state of emergency which is declared and that makes police and security sector to work day and night on the issue. Accordingly, the informant said that department is working in coordination with parallel institutions and supporting and supervising higher and lower tiers in prevention and controlling the COVID-19 pandemic. According to Ato Tessema, institutions are formed to adequately respond to deter the possible expansion of the pandemic. The institutional capacity to work with other departments and institutions is well enough. This is mainly based on the structures and directives from the Federal and regional governments with some little contextualization to the local context.

According to the informant, most human actions are not still inline to the state of emergency. For example, religious leaders are still continued to take their usual activities. Markets are also gathering as usual as it has been in past. The command post has not divided markets into different possible places. In all these cases, the directive of the federal government was not implemented. The head admits that this absent mindedness my end up bad consequences; but economic and related problems in the area put pressure to tolerate the action.

1. ***How you label the participation of your institution in responding to the COVID-19?***

- ***In differentiating the extent and character of the pandemic***
- ***Taking primary responsibility (Who initiated Prevention and control of COVID-19?)***
- ***In supporting those who are taking primary responsibility in prevention and control***
- ***What kind of approach could be effective in prevention and control of COVID-19?***
- ***How do you see the manner of engagement of different stakeholders?***
  - ***Its necessity***

The head of the department labels the participation of his institution as crucial in prevention and controlling the pandemic. All workers in the sector are informed to differentiate the extent and character of the pandemic and to act accordingly. Thanks to ministry of health and media, the level of awareness both by the workers in different sector and by the broader society well entrenched. Accordingly, the department of Peace and security takes primary responsibility in implementing state of emergency which is initiated by the Federal government. Therefore, as the head indicated the sector plays decisive role working with lower sectors of Woreda up to Kebele. The sector has especial collaboration with departments of transport, health and justice.

The current approach of the Federal government that is keeping social distance, washing hands, using hand sanitizers and transportation rules are irreplaceable approaches to tackle the problem of COVID-19, the informant said. Therefore, the sector feels responsible to put these things implemented. But, as the head indicated challenges from local communities are creating political and security crises. For example, when motor cycles were banned to give riding service, it also became a challenge to peace and security aspect of the zone. Robbery, theft and killings of someone to take money and such criminal actions became prevalent. Therefore, the local government passed decision to tolerate some aspects of the state of emergency and now motor cycles are working as usual. It is not only motor cycles but pool and coffee houses are also backed the previous status.

But, the interviewee challenged the arguments of the head in saying that ‘why the motor ride service givers are not at least putting on face mask? Why not you oblige pool houses to put on face masks and to clean sticks with sanitizer at every interval before transferred to another? Why not you enforce using face mask by everybody? For these all questions surprisingly, the respondent said that it is not to take serious actions against the people.

1. ***What is the role of your institution in prevention and control of COVID-19?***

The role of the department is leading other sectors to the goal, bringing issues to the grand and main committee and implementing the state of emergency in all sectors in the zone, the head said. Accordingly, actions were taken in transportation and one instance Ato Tessema indicated was one minibus was fined 11,000 birr for carrying 11 extra peoples of expected 50%. Therefore, according to my informant there are attempts to implement state of emergency with some ups and downs.

1. ***If you are participating in prevention and controlling of COVID-19, how do you do so? What kind of principle do you use?***

- ***If you have any supportive document***

The department takes direct measure and authorized to do by the federal government. Using police force, it strews people gathered in some places. At four entry directions in to the zone, namely *Buge, Dimtu, Abala Abaya* and *Achura*, the security forces checking and controlling exit and entry from and to the zone. This responsibility is discharged not only at day time but also at night since illegal transporters should not enter without temperature screening. Moreover, it forces individuals to put on face masks and controls transportations as per the rule of the federal government. Night clubs, pool houses, coffee houses and bars and mead houses are supervised by peoples assigned from peace and security sectors. But, the reality is that these things are highly tolerated.

The principle the peace and security uses for prevention and controlling the pandemic is implementing the state of emergency and mobilizing the collective effort for that endeavor. To so also, there are national plans which reaching to local level of government.

1. ***How do you assess your institutions response against the Pandemic?***

- ***If any responsible person assigned for the purpose***
- ***If any institutional plan cascaded from common plan***
- ***If any allocation of resource necessary***
- ***If any supervisor and support***

As the informant indicated, the institutional response against the pandemic is lenient and not serious enough. This is repeatedly indicated by the head of the department that enabling individuals to work and to sustain their life. He also he said that the zone is the one with immense number of unemployed youths, roughly about 900,000. But, at least to make some supervision, there is responsible person assigned for the purpose in line with institutional plans cascaded from the common plan. Necessary resources are also allocated with required supervisor and support.

1. ***If you are working with other institutions in collaboration, they how do work in such relation? What is the guideline?***

- ***If any supportive document to woke together***
  - ***Implementation of Commonly deigned plan***
- ***Goal clarity and common understandings***
- ***If you are working together, how often you meet and at what level you cooperate***
- ***What is responsibility of your department in the group work? At what level you participate?***

The collaboration and cooperation with other institutions contributes a lot for the prevention and controlling of the COVID-19 pandemic. In this regard, the role of Wolaita Soddo University is considerable, according to the informant. They provide containment and quarantine centers, food for peoples hosted there and clinical services by relevant professionals. Moreover, religious leaders, Red-cross, youth and Soddo Agriculture College are providing all necessary services and working with peace and security department to prevent and control the pandemic. For this end, there are directives cascaded from federal up to Kebele administration in the implementation of the state of emergency.

1. ***What evidence do you have that the approach so far used is leading to the targeted goal?***

- ***Support and supervision***

Ato Tessmea do not agree on the way they were moving so far as approach to tackle the pandemic. He said that, our enforcement measures have to implement in line to the directives set by the federal government to save the life of the generation. It is mandatory for implementing the state of emergency and taking serious measures as stated in the directive.

1. ***If you have joint meetings and how often do you take such meetings***

- ***If you use technological inputs***

The department head of peace and security, Ato Tessema said that his sector is strongly working to achieve the goal in collaboration with Transport, Health and Justice. These four sectors taking meetings every day or at least frequent calls each other to share ideas. For this end there are practices of using new technologies such as launching mini-medias at all 22 woreda bus stations to teach about the pandemic. Moreover, using temperature screening at entry of the zone and each bus station is another technological input.

**Interviewer ………………………… Yoseph Watte & Semagegn Dansa**

**Interviewee ………………………….. department of Government Communications**

**Place of Interview ……………………..Office of the head of the department**

**Date: ………………………………………**

**Time- Started …………………………… 5:30**

**Ended ………………………..……6:30**

**Consent for Recording ………………….. Confirmed**

**Questions**

1. ***What is the link between your institution and COVID-19 Prevention and controlling?***

- ***In coordinating, supporting and supervising higher and lower tiers; with other departments; and/or your direct engagements in Prevention and control of the pandemic.***
- ***Establishing responsive institutions***
- ***Institutional capacity to work with other competent departments and institutions***

According to Wondimu Woldie, his department is one of the important sectors in COVID-19 prevention and controlling. Since media is a crucial institution in reaching to the broader society, the way it is managed and administered maters the success or failure of the intervention. Therefore, according to the informant, the institution works strongly in awareness creation on prevention and controlling; and for mobilization of different sectors in fund raising. For this purpose, the government communication department works in collaboration with higher and parallel sectors and supervising the lower tiers. In this regard, according to the informant, the department is member of grand committee and main committee and chairing committees within the institution.

All journalists are assigned to work due commitment in awareness creation to save the lives of the generation as the informant said. Accordingly, both *Fana* and *Wogeta* Radios are working in and outside the studio. For example, moving to market places where usually large numbers of peoples are gathered from the zone and surrounding areas of zones and distant areas. Thus broadcasting in market places for example on Monday-Gesuba, on Tuesday-Boditti and Areka, on Thursday-Humbo and on Saturday-Soddo are important days of marketing to exploit the opportunity of accessing such number of people at a day. According to him, government communication department feels responsive sector in tackling the pandemic having institutional capacity to work with other competent departments and institutions.

1. ***How you label the participation of your institution in responding to the COVID-19?***

- ***In differentiating the extent and character of the pandemic***
- ***Taking primary responsibility (Who initiated Prevention and control of COVID-19?)***
- ***In supporting those who are taking primary responsibility in prevention and control***
- ***What kind of approach could be effective in prevention and control of COVID-19?***
- ***How do you see the manner of engagement of different stakeholders?***
  - ***Its necessity***

As the vice head states, the participation of government communication in responding COVID-19 is average. He put in this label when the interviewer raised some instances that Medias should have been worked. For example, media should have brought the concentration at market into the attention of the local government and could have pushed for further action. Not only that the concentration at religious centers, peoples gathering at hotels, work places and social gathering scuh as Idir and equb should be broadcasted and should have got government attention. In relation to this, the v/head also revealed one fact that delegates from federal government visited the zone and evaluated the performance of different sectors in COVID-19 Prevention and Controlling. According to him, the eventual result found to low. This indicates that the zonal administration and its sectors are not at level of protecting their people from the pandemic.

Yet, as it is responded by the informant, *Fana* and *Wogeta* are trying to reach the public specially on changing attitudes. In all these, there is deep engagement and collaboration with road transport, peace and security, health and child and women department.

1. ***What is the contribution of your institution in prevention and control of COVID-19?***

The vice head of government communication indicates his department is trying to reach broader society in teaching the ways of transmission and prevention of the pandemic.

1. ***If you are participating in prevention and control of COVID-19, how do you do so? What kind of principle do you use?***

- ***If you have any supportive document***

The v/head of the department indicated that his department is participating in every sector in broadcasting what sectors and departments are doing. Therefore, accordingly, it is not independent functioning of the sector only but it also works in strong collaboration with all departments in the zone. For example, the government communication department is part of the grand, main committee and technical committees of the zone. This implies it works in partnership with other sectors.

According to Wondimu, the government communication uses the principle that ‘information is power’. He said that in breaking information gap and introducing directives of the federal government which is adopted from World Health Organization is crucial. They peoples are oriented and resultant change in attitude matters in confronting the COVID-19 pandemic. To carry out these actions, there are supportive documents in the department.

1. ***How do you assess your institutions response against the Pandemic?***

- ***If any responsible person assigned for the purpose***
- ***If any institutional plan cascaded from common plan***
- ***If any allocation of resource necessary***
- ***If any supervisor and support***

The institutional response of the government communication department is adequate since it trys to reach to the every corner of the society. Accordingly, in rural areas local languages are also used to make it more clearly for the public. With this regard, there is focal person assigned and there are institutional plans cascaded from federal and regional directives. Resources are mobilized and supervision and supports are taken.

1. ***If you are working with other institutions in collaboration, they how do work in such relation? What is the guideline?***

- ***If any supportive document to woke together***
  - ***Implementation of Commonly deigned plan***
- ***Goal clarity and common understandings***
- ***If you are working together, how often you meet and at what level you cooperate***
- ***What is responsibility of your department in the group work? At what level you participate?***

As my informant, the government communication department by its nature is the mouth of government sectors in transmitting information on what each sector is doing and plans to do. Therefore, it not only working in collaboration but it is geminated to parallel, lower and higher tiers of departments. It is partly based on media policy and partly on directives set with state of emergency. There are supportive documents indicating so and also there are cross-sector designed plans. As the v/head, there is clarity of goals and also common understanding. For this regard, they are taking meetings every three days and when urgency happens, it can take at any time.

1. ***What evidence do you have that the approach so far used is leading to the targeted goal?***

- ***Support and supervision***

As the v/head indicated, there is no evidence implying for reaching to the targeted goal. In the zone, there is no effective implementation of the state of emergency and that directives of the federal government are not implemented.

1. ***If you have joint meetings and how often do you take such meetings***

- ***If you use technological inputs***

Joint meetings are held once a week and now with other political issues such schedules are extended to 15 and more. It is not like what it has been at the beginning. Now there is absent mindedness both on government as well as on the public side.

**Interviewer ………………………… Yoseph Watte & Semagegn Dansa**

**Interviewee ………………………….. department of**

**Education**

**Place of Interview ……………………..Office of the head of the department**

**Date: ………………………………………**

**Time- Started …………………………… 2:30**

**Ended ………………………..……3:30**

**Consent for Recording ………………….. Confirmed**

**Questions**

1. ***What is the link between your institution and COVID-19 Prevention and controlling?***

- ***In coordinating, supporting and supervising higher and lower tiers; with other departments; and/or your direct engagements in Prevention and control of the pandemic.***
- ***Establishing responsive institutions***
- ***Institutional capacity to work with other competent departments and institutions***

The department of education is the one which is highly affected by the incidence of the COVID-19 pandemic. Ranging from kindergartens to preparatory levels has huge number of population and thus schools are places where there are greater human contacts. According to Dr. Teferi, usually classes are hosting more students than the expected standard. Therefore, what happens at school has direct effect in the community since students are there almost from every family. That is why government closed schools immediately after the happening of the pandemic in the country.

According to him, his department works in collaboration with other departments. For example, in coordination with health and peace and security department, some selected schools are prepared for containment and quarantine services.

In the meantime, as the head said that based on the directions of Minister of Education, the department tries to make students close to their exercise and text books. Teachers are advised to prepare assignments and to give to their students. This is in order to make students to read text books at their hand and to spend more time at home than outside. But, according to the head, the reality is different when supervision is taken. For example, while schools are closed to ensure social distance, however, most students are outside their home playing each other. This is missing the point of the state of emergency.

1. ***How you label the participation of your institution in responding to the COVID-19?***

- ***In differentiating the extent and character of the pandemic***
- ***Taking primary responsibility (Who initiated Prevention and control of COVID-19?)***
- ***In supporting those who are taking primary responsibility in prevention and control***
- ***What kind of approach could be effective in prevention and control of COVID-19?***
- ***How do you see the manner of engagement of different stakeholders?***
  - ***Its necessity***

According to Dr. Teferi, the participation and engagement of education department is so strong. In order to make his idea clear, he has listed some the activities such as closing schools, enforcing private schools to retain their employees and making some selected schools ready for containment and quarantine purpose. To achieve these, the department is differentiating the extent and character of the pandemic.

The primary initiation has come from Grand committee and then being implemented throughout the zone based on the national directions, as the informant. The department is member of the grand committee and supporting it in all directions. Hence according to the head, directions set by the national government are irreplaceable to ban the expansion of the pandemic. For this purpose, the department is working in collaboration with youth associations, department of health, transport and peace and security. For such coordinated efforts, the nationally designed action plans which are cascaded into regions and zones are very important.

1. ***What is the rolr of your institution in prevention and control of COVID-19?***

Dr. Teferi limited the role of his department into three major parts. The first is making institutions/schools ready to contain the suspected ones and to use some selected schools for quarantine purpose. The second one is using studios of ‘Education by Radio’ for the awareness creation using local language. The other role of the department is public mobilizations for resource generation. Resource mobilization is vital since dealing with COVID-19 is costly engagement.

1. ***If you are participating in prevention and control of COVID-19, how do you do so? What kind of principle do you use?***

- ***If you have any supportive document***

According to information of Education department head, his sector is participating in COVID-19 prevention and controlling. Some of the activities are taken by the institution by itself and the others are done in collaboration with other sectors. These are taken as the directions already designed at central government. The principle that the education sector uses is mass mobilization to deter the expansion of the pandemic.. Some of the activities are also included in minutes.

1. ***How do you assess your institutions response against the Pandemic?***

- ***If any responsible person assigned for the purpose***
- ***If any institutional plan cascaded from common plan***
- ***If any allocation of resource necessary***
- ***If any supervisor and support***

The head of the department explains the level of participation of his institutions as strong as possible. Accordingly, it tries all the best to intervene in issues under its jurisdiction. Moreover, it is involving with full engagement with other sectors. According to the head, at this time no schools and that the only attention of the department is on prevention and controlling of the pandemic. For this purpose, there are assigned persons from the sector. There are also joint plans cascaded from the national plan and mobilizing resources for tackling the pandemic. Follow-up and supports are complementing these efforts.

1. ***If you are working with other institutions in collaboration, they how do work in such relation? What is the guideline?***

- ***If any supportive document to woke together***
  - ***Implementation of Commonly deigned plan***
- ***Goal clarity and common understandings***
- ***If you are working together, how often you meet and at what level you cooperate***
- ***What is responsibility of your department in the group work? At what level you participate?***

1. ***What evidence do you have that the approach so far used is leading to the targeted goal?***

- ***Support and supervision***

1. ***If you have joint meetings and how often do you take such meetings***

- ***If you use technological inputs***

The head of education department, Dr. Teferi said that their sector is working with other institutions in collaboration. To do so, there are guidelines and directives set by federal and regional governments. Common understanding and goal clarity over joint/aggregate plans are created. Thus is the result of frequent meetings that are held every five days. This helped to stand together for common goal.

At grand and main committee, the department has member status. At sector level, the head chairs every activities of the institution. This is supported by supervisions. These meetings however were not supported by technological inputs. But, for prevention and controlling the pandemic, innovative actions were taken by some high school students. For example, sterilizer was invented by high school student to deter the transmission of the virus from customers. As the head stated, papers, shoes and phones of customers are sterilized before used in offices.
